# The effects of MicroRNA transfections on global patterns of gene expression in ovarian cancer cells are functionally coordinated

**DOI:** 10.1186/1755-8794-5-33

**Published:** 2012-08-01

**Authors:** Shubin W Shahab, Lilya V Matyunina, Christopher G Hill, Lijuan Wang, Roman Mezencev, L DeEtte Walker, John F McDonald

**Affiliations:** 1 School of Biology, Georgia Institute of Technology, Atlanta, GA, 30332, USA; 2 Parker H. Petit Institute for Bioengineering and Bioscience, Georgia Institute of Technology, Atlanta, GA, 30332, USA; 3 Ovarian Cancer Institute, Atlanta, GA, 30342, USA

## Abstract

**Background:**

MicroRNAs (miRNAs) are a class of small RNAs that have been linked to a number of diseases including cancer. The potential application of miRNAs in the diagnostics and therapeutics of ovarian and other cancers is an area of intense interest. A current challenge is the inability to accurately predict the functional consequences of exogenous modulations in the levels of potentially therapeutic miRNAs.

**Methods:**

In an initial effort to systematically address this issue, we conducted miRNA transfection experiments using two miRNAs (miR-7, miR-128). We monitored the consequent changes in global patterns of gene expression by microarray and quantitative (real-time) polymerase chain reaction. Network analysis of the expression data was used to predict the consequence of each transfection on cellular function and these predictions were experimentally tested.

**Results:**

While ~20% of the changes in expression patterns of hundreds to thousands of genes could be attributed to direct miRNA-mRNA interactions, the majority of the changes are indirect, involving the downstream consequences of miRNA-mediated changes in regulatory gene expression. The changes in gene expression induced by individual miRNAs are functionally coordinated but distinct between the two miRNAs. MiR-7 transfection into ovarian cancer cells induces changes in cell adhesion and other developmental networks previously associated with epithelial-mesenchymal transitions (EMT) and other processes linked with metastasis. In contrast, miR-128 transfection induces changes in cell cycle control and other processes commonly linked with cellular replication.

**Conclusions:**

The functionally coordinated patterns of gene expression displayed by different families of miRNAs have the potential to provide clinicians with a strategy to treat cancers from a systems rather than a single gene perspective.

## Background

MicroRNAs (miRNAs) are a class of small (18–24 nucleotides) regulatory RNAs that are crucial in many cellular processes including development, angiogenesis, cell cycle and cellular migration [1]. These small RNAs are encoded in the genomes of both unicellular [2] and multicellular organisms and typically repress gene expression at the post-transcriptional level [3]. In humans, miRNAs have been shown to repress translation (primarily through degradation of target mRNAs) by interacting with 3’untranslated regions (UTR) in a sequence specific manner [4,5]. In rare instances, miRNAs have also been reported to increase translation [6] and/or transcription [7] of target genes.

There is a large and growing body of evidence that many diseases, including cancer, are associated with changes in cellular miRNA levels [8]. In cancer, levels of specific miRNAs have been reported to be significantly down- or up-regulated in various cancer types indicating that these regulatory RNAs may be operationally defined as either tumor suppressor genes or oncogenes depending upon the cellular context [9]. Based on these findings, the clinical potential of miRNAs as cancer biomarkers and/or therapeutic agents is being actively pursued [10].

A continuing challenge to the effective use of miRNAs in cancer therapy is our limited ability to accurately predict the molecular consequences of exogenous perturbations in cellular levels of miRNAs [11]. The difficulty in anticipating the full molecular consequences of miRNA therapy may be due, in part, to the limitations of *in silico* target prediction algorithms [12] and to the fact that miRNAs can directly and/or indirectly modulate expression levels of multiple genes in addition to the intended target(s) [13].

In an effort to better understand the range of molecular changes potentially associated with miRNA therapy for ovarian cancer, we conducted a series of controlled experiments in which two miRNAs previously implicated in ovarian cancer onset/progression [14] were individually transfected into a well-defined ovarian cancer (HEY) cell line [15], and the consequence on global patterns of gene expression monitored by microarray (Affymetrix HG-U133 Plus 2.0) and quantitative (real-time) polymerase chain reaction (qPCR). Functional predictions derived from the gene expression analyses were experimentally validated. Our results imply that miRNAs may be clinically useful in a systems approach to cancer therapy.

## Results

### Ectopic expression of miR-7 or miR-128 significantly down-regulates EGFR in HEY cells

**Figure 1:**
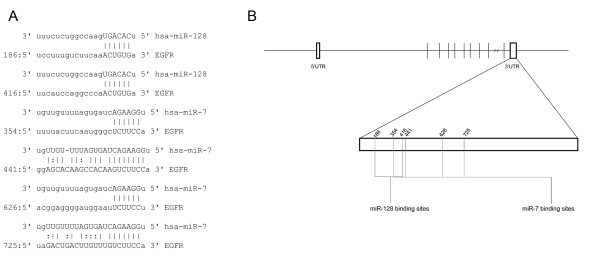
**Predicted miR-7 and miR-128 target sites (miRanda) on *EGFR *(epidermal growth factor receptor gene) 3’UTR (untranslated region).** (**A**) Nucleotide sequences of miR-128 and miR-7 “seed” regions and complementary “target” sequences on the *EGFR* UTR. Vertical lines ( | ) denote Watson-Crick base pairing, while the colon (:) sign denotes wobble between G and U on opposite strands. (**B**) Schematic illustration of the relative location of each target site on the EGFR gene.

**Figure 2:**
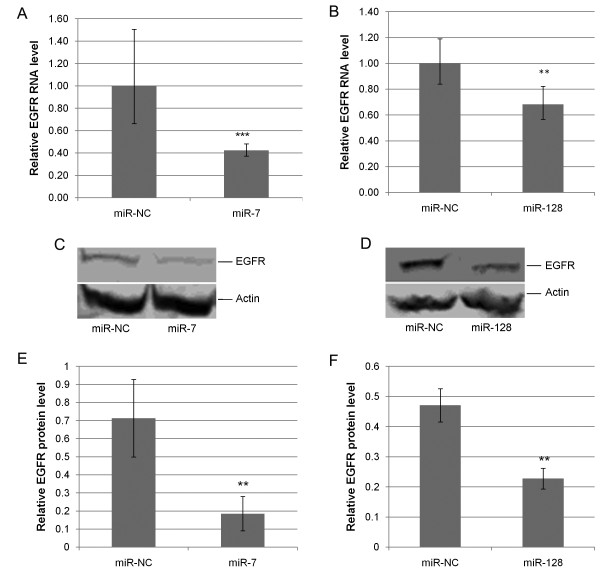
**miRNAs miR-7 and miR-128 down-regulate EGFR in HEY cells.** (**A** &**B**) Histograms showing the average of independent biological repicates of qPCR determined EGFR RNA levels following transfection of miR-7 or miR-NC (**A**), and miR-128 or miR-NC (**B**) in HEY cells using GAPDH as the endogenous control. Statistical analysis for qPCR was carried out using randomization (REST 2008 software) [42] with at least 1000 iterations [Note that the REST Software determines gene 95% confidence intervals for gene expression ratios using a bootstrapping technique without normality or symmetrical distribution assumptions. Thus, the error bars will not necessarily be equal in the plus and minus directions] (*** p <0.001, ** p <0.05); (**C** &**D)** Representative western blots of EGFR protein levels after transfection of miR-7 and miR-NC (**C**) or miR-128 and miR-NC (**D**); (**E** &**F**) Histograms showing average densitometric analyses of independent biological replicates of immunoblots measuring protein levels of EGFR following transfection of miR-7 and miR-NC (**E**) or, miR-128 and miR-NC (**F**). Statistical analysis of immunoblots was carried out using a 2-tailed t-test (** p <0.01).

To assess the biological effectiveness of our transfections, we monitored changes in levels of EGFR, a previously validated target of miR-7 and miR-128 [16,17]. These previous findings are consistent with the fact that the 3’UTR of *EGFR* contains up to four predicted binding sites for miR-7 and two predicted binding sites for miR-128 (Figure 1). Since we previously demonstrated that EGFR is highly expressed in HEY cells [15], we expected levels of EGFR mRNA and protein to be significantly reduced after a successful miR-7 or miR-128 transfection (Additional file 1: Figure S1). Consistent with this expectation, our results demonstrate that over-expression of either miR-7 or miR-128 in HEY cells results in significant down-regulation of EGFR expression (Figure 2).

### miR-7 or miR-128 transfection induces changes in expression of hundreds to thousands of genes

To study the effect of over-expression of miR-7 or miR-128 on global patterns of gene expression, we isolated total RNA from cells 48 hours after transfection and conducted microarray analysis (HG-U133 Plus 2.0). The results indicate that transfection of miR-7 induced significant changes in the expression of 754 genes (fold change ≥1.4, FDR ≤5%; Additional file 1: Table S1) and that miR-128 transfection induced significant changes in the expression of 2338 genes (Additional file 1: Table S2), both relative to controls. The number of genes differentially expressed in both experiments was 286 (overlap of the two experiments). For miR-7, the majority of the differentially expressed genes (DEGs) were down-regulated (599, ~80%), while for cells transfected with miR-128, most (1624, ~70%) of the DEGs were up-regulated.

### Less than 1% of the miR-7 or miR-128 induced changes in gene expression are the consequence of down-regulation of EGFR

To eliminate the possibility that miRNA induced changes in levels of RNA might simply be explained by the down-regulation of EGFR, we monitored the effect of EGFR siRNA transfection on global changes in levels of RNA and contrasted the results with expression patterns observed after miR-7 or miR-128 expression.

After verifying knock-down of EGFR by siRNA transfection (Additional file 1: Figure S2), we monitored global patterns of gene expression by microarray. Employing identical criteria to that used for the detection of differentially expressed genes after the miRNA transfections (fold change ≥1.4, FDR ≤5%), we found that only eight genes (in addition to *EGFR*) displayed a significant change in levels of RNA (Additional file 1: Table S3). (Note that the majority of regulatory effects associated with EGFR knockdown are expected to operate on the post-transcriptional level and will not be detected by microarray analyses). Only two of these genes (including *EGFR*) are altered in all three transfections. Three of the genes differentially expressed after siRNA transfection are also differentially expressed after miR-7 transfection, only five of the nine are also differentially expressed after miR-128 transfection, and only three were differentially expressed only in the siRNA transfection. These findings indicate that the vast majority of changes in levels of mRNA observed after miR-7 or miR-128 transfection are not merely the consequence of knock-down in EGFR expression.

### Less than 20% of the genes differentially expressed after miR-7 or miR-128 transfection are predicted targets of these miRNAs

A single miRNA can potentially target hundreds of genes [18,19]. To explore the possibility that the global changes in expression patterns observed after miR-7 or miR-128 transfection were the result of direct targeting, we first computed the percentage of the down-regulated genes potentially regulated by miR-7 or miR-128 as predicted by three major target prediction algorithms (miRanda, TargetScan, and PicTar). The results indicate that on average < 20% of the down-regulated genes differentially expressed after miR-7 or miR-128 transfection are direct regulatory targets of these miRNAs (Table 1). For example, of the 599 genes down-regulated after miR-7 transfection, only 194 or 32.4% are predicted to be targets of this miRNA by miRanda. The percentages are lower using targets predicted by the PicTar (6.5%) and TargetScan (10.2%) algorithms.

**Table 1:** Fraction of down-regulated, up-regulated and total differentially expressed genes predicted to be targets of miRNAs

	**miR-7**				**miR-128**			
	**M**	**TS**	**PT**	**AVG**	**M**	**TS**	**PT**	**AVG**
**Down (%)**	32.4	10.2	6.5	16.4	36.0	14.7	9.1	19.9
**Up (%)**	12.3	1.9	0.6	4.9	18.8	3.8	2.6	8.4
**All (%)**	28.2	8.5	5.3	14.0	24.0	7.1	4.6	11.9

Significantly down-regulated (Down), up-regulated (Up) or total differentially expressed genes (All) after miR-7 or miR-128 transfections in HEY cells were searched for targets of the respective miRNAs using miRanda (M), TargetScan (TS) and PicTar (PT) algorithms. The fraction (%) of targets within each group and the average (AVG) of all 3 algorithms are reported in this table.

Although increased levels of miRNAs are generally expected to down-regulate the expression of target genes, there have also been reports of the expression of target genes being increased after miRNA transfection [7]. If we include all genes that are either down-regulated or up-regulated after miR-7 or miR-128 expression, the percentage of predicted targets remains, on average, < 20% (range: 4.6 to 28.2%) (Table 1). We conclude that less than one-fifth of the genes differentially expressed after miR-7 or miR-128 transfection are likely the result of regulatory effects directly exerted by these miRNAs.

### The effect of miR-128 transfection on patterns of gene expression may be partially explained by the de-repression of endogenous miRNA targets

It has been previously proposed that transfection of exogenous miRNA molecules may interfere with endogenous miRNA targeting of mRNAs due to competition for a limited number of RNA-Induced Silencing Complexes (RISC) [20]. According to this model, if endogenous miRNAs are out competed for RISCs by exogenous miRNAs, the targets of the endogenous miRNAs may be expected to be up-regulated. In a pre-transfected cell, endogenous miRNAs with higher expression values are predicted to have a greater likelihood of being in the RISC and, thus, more likely to be affected by introduction of exogenous miRNAs [20,21].

As mentioned above, a majority (~70%) of the 2338 genes displaying significant changes in expression after miR-128 transfection were up-regulated (Additional file 1: Table S2). We have previously shown that our transfection protocol results in a significant increase in levels of the transfected miRNA (~ 130X) [22]. To determine if the RISC competition model might help explain some of the effects of miR-128 transfection, we sought to determine if the target sequences of miRNAs most highly expressed in pre-transfected HEY cells were significantly enriched in genes up-regulated after miR-128 transfection.

We employed a previously established gene set enrichment analysis algorithm (Genomica; [23]) to identify miRNA target sites enriched in mRNAs up-regulated after miR-128 transfection. The results indicate that target sites of 78 miRNAs are significantly enriched among the 1624 genes up-regulated after miR-128 expression (Additional file 1: Table S4).

**Figure 3:**
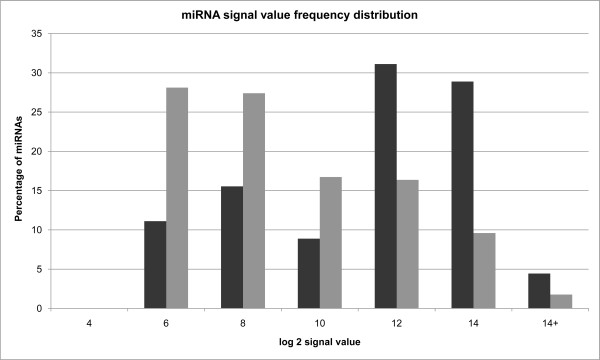
**Endogenous miRNAs that target up-regulated genes are expressed at relatively higher levels in pre-transfected cells.** Frequency distribution of log_2_ signal values for all 281 miRNAs expressed in HEY cells (grey) compared with signal value distribution for 45 miRNAs expressed among those targeting up-regulated genes after miR-128 transfection (black). The bar chart shows that the 45 miRNAs targeting up-regulated genes have a greater frequency of being expressed at log_2_ signal value >10, while most miRNAs frequently have log_2_ (signal value) ≤10 (Mann–Whitney p-value <0.0001). Only miRNAs with at least one “TRUE” detection (Additional file 1 Table S5) call across all samples and with a ‘hsa’- prefix in probeset names were used for plotting the distribution of signal values. Frequency of miRNAs having signal values between log_2_ 4–14 was calculated and plotted using the ‘data analysis’ package in Microsoft Excel.

To test whether these 78 miRNAs were expressed at higher levels in pre-transfected HEY cells relative to all expressed miRNAs, we measured levels of human miRNAs in pre-transfected HEY cells by microarray (Affymetrix GeneChip miRNA array; Additional file 1: Table S5). Out of 847 human miRNAs present on the chip, 281 were found to be expressed in pre-transfected HEY cells, and out of the 78 miRNAs with target sites enriched among up-regulated genes after miR-128 transfection, 45 were expressed in pre-transfected HEY cells. Figure 3 displays a histogram of log_2_ signal values for all expressed miRNAs (281) and for the subset of miRNAs (45) with target sites significantly enriched in genes/mRNAs up-regulated after miR-128 transfection. The results demonstrate that miRNAs with binding sites significantly enriched in up-regulated genes display a significantly greater fraction of signal values above log_2_ (10) relative to all miRNAs (Mann–Whitney p-value <0.0001) indicating a generally higher level of expression in pre-transfected HEY cells. While these findings are consistent with the hypothesis that at least some of the increased level of gene expression observed after miR-128 transfection may be the indirect effect of the displacement of endogenous miRNAs from the RISC, they do not exclude alternative hypotheses (*e.g.*, see below). In addition, the RISC displacement model does not explain the 714 genes down regulated after miR-128 transfection. Nor does it explain the vast majority of the genes differentially expressed after miR-7 transfection.

As reported above, only ~20% of differentially expressed genes were up-regulated after miR-7 transfection. When we employed the Genomica gene set enrichment analysis algorithm [23] to identify miRNA target sites enriched in mRNAs up-regulated after miR-7 transfection only 2 miRNAs were found to be significantly enriched among the up-regulated genes (Additional file 1: Table S6). Thus, the RISC displacement model does not explain the up-regulation of genes after miR-7 transfection or the fact that the vast majority of genes were down-regulated after miR-7 transfection. While the reason for this discrepancy is currently unknown, we note that there are several previous reports of relatively little up-regulation of genes after microRNA transfections [4,13,24,25]. Perhaps other indirect effects of miRNA transfections (*e.g*., see below) may override or mask the consequence of the displacement of endogenous miRNAs from RISC or possible variability in the binding affinity of different miRNAs for RISC [26] may modulate the relative effectiveness of miRNA displacement.

### miR-7 or miR-128 transfection may trigger cascades of indirect regulatory changes in gene expression

GeneGo functional and binding interaction network analysis was used to identify genes with the most significant number of interactions (“hub genes”) among genes differentially expressed after miR-7 or miR-128 transfection. miRNA induced changes in the expression level of hub genes are expected to induce subsequent changes in the expression of genes regulated by these hub genes. If these regulated genes include other hub genes, the regulatory “ripple effect” can be significant.

**Figure 4:**
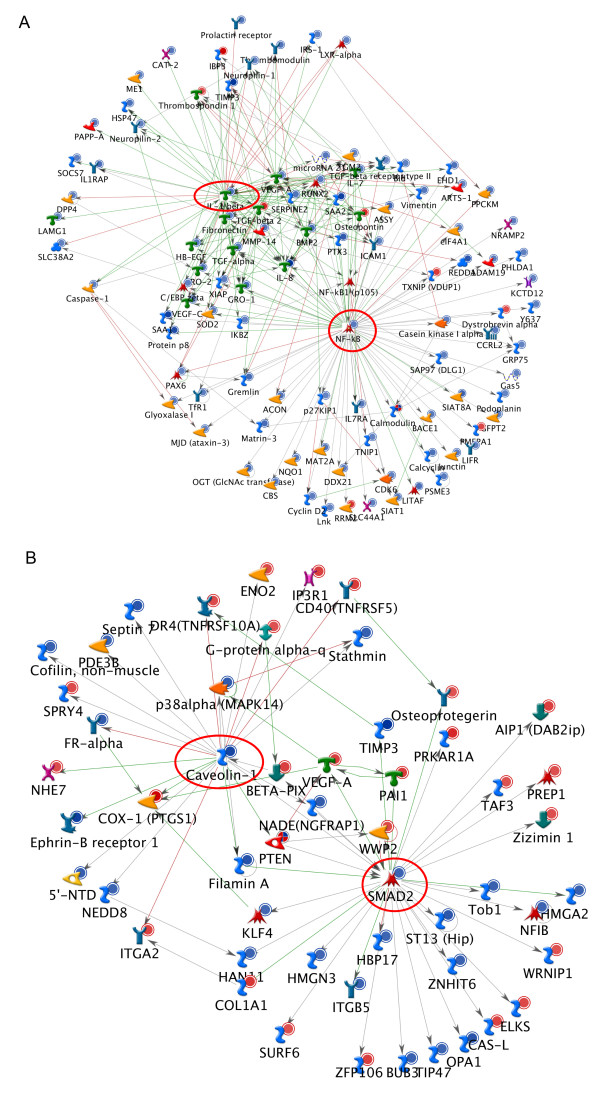
**Examples of how miRNA regulation of hub genes indirectly affect the expression of large numbers of downstream genes.** (**A**) Among genes differentially expressed after miR-7 transfection, *RELA/NF-κB* (circled in red) acts as a hub gene, and is found to target many of the differentially expressed genes. One of these, IL1-beta (circled in red) is significantly down-regulated and targets additional differentially expressed genes, thus, amplifying the effect of NF-κB down-regulation. (**B**) Caveolin-1 (circled in red) is a significantly down-regulated hub gene and a predicted target of miR-128. One of CAV1’s many direct downstream target genes is SMAD2 (also circled in red; regulation shown by arrow/edge pointing from Caveolin-1 to SMAD2), which itself acts as another hub gene. Several downstream targets of SMAD2 are also differentially expressed following miR-128 transfection. Thus by targeting the hub gene CAV1, miR-128 can regulate the non-target hub gene SMAD2 and trigger a ripple effect on the expression of down-stream genes. Key: blue filled circle - significantly down-regulated gene as determined by microarray analysis; red filled circle – significantly up-regulated gene as determined by microarray analysis; green edges: established activating interaction; red edges: established inhibitory interaction; grey edges: predicted interaction of unknown significance.

Functional network analysis identified 10 hub genes among those differentially expressed after miR-7 transfection and 61 among genes differentially expressed after miR-128 transfection (FDR <0.05). Many of these hub genes are predicted to be direct targets of miR-7 or miR-128 regulation (Additional file 1: Table S7 and Additional file 1: Table S8). For example, the gene encoding the RELA component of the NF-κB regulatory protein is a predicted target of miR-7 and was significantly down-regulated after miR-7 transfection (Additional file 1: Table S1). Consistent with the hypothesis that down-stream effects of RELA may be contributing to indirect changes in expression levels, we found that a number of the genes differentially expressed after miR-7 transfection are documented targets of NF-κB regulation. Among these is the cytokine IL-1 beta [27] that, in turn, is predicted to regulate genes that were also differentially expressed after miR-7 transfection (Figure 4A, Additional file 1: Table S9).

Similar changes in patterns of gene expression were observed after miR-128 transfection. For example, Caveolin-1 (CAV1) is a negative regulator of the Ras-p42/44 MAP kinase cascade [28]. Levels of CAV1 were significantly reduced after miR-128 transfection and down-stream targets of CAV1 were among those genes significantly differentially expressed (Additional file 1: Table S10 and Additional file 1: Table S2). Among the CAV1 regulated genes displaying significant changes in expression after miR-128 transfection is another hub gene, the transcription factor SMAD2 [29]. Genes known to be trans-regulated by SMAD2 were also differentially expressed after miR-128 transfection (Figure 4B, Additional file 1: Table S10). Collectively, our results are consistent with the hypothesis that a major fraction of the changes in expression induced after miR-7 or miR-128 transfection are likely the indirect or down-stream consequences of changes in hub gene expression.

### miR-7 and miR-128 transfection modulates changes in the expression of genes involved in distinct developmental and cell cycle related pathways

Our results indicate that the direct and indirect effects of miR-7 and miR-128 transfection on global patterns of gene expression are quite distinct. Only 286 genes were in common among the 754 genes differentially expressed after miR-7 transfection and the 2338 genes differentially expressed after miR-128 transfection. To evaluate the potential functional significance of the genes uniquely differentially expressed after either miR-7 or miR-128 transfection, we subjected the gene expression data to GeneGo pathway enrichment analysis.

**Figure 5:**
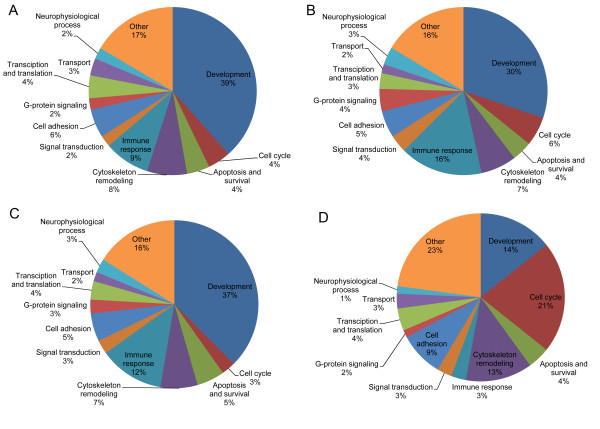
**Genes differentially expressed after miR-7 or miR-128 transfection display distinct pathway signatures.** The majority of pathways enriched (FDR <0.05) among (**A**) differentially expressed genes after miR-7 transfection, (**B**) differentially expressed genes after miR-128 transfection are related to development. Cell cycle, cytoskeleton remodeling, apoptosis and signal transduction pathways are also enriched and may signify cancer specific processes. Distribution of groups of pathways enriched among (**C**) down-regulated genes after miR-7 transfection or (**D**) down-regulated genes after miR-128 transfection indicate that pathways involved in development are the largest fraction of enriched pathways for miR-7 while genes down-regulated by miR-128 are enriched for pathways involved in cell cycle regulation revealing a distinct pathway signature for each miRNA.

The results of our functional pathway analysis indicate that genes differentially expressed after miR-7 transfection are significantly over-represented in 91 functional pathways (Figure 5A, Additional file 1: Table S11). The 20 most significantly over-represented pathways are listed in Table 2A and include cell adhesion and other pathways associated with epithelial-mesenchymal transition (EMT). We found that genes differentially expressed after miR-128 transfection are over-represented in 231 pathways (Figure 5B, Additional file 1: Table S12). The 20 most significantly over-represented pathways listed in Table 2B include a variety of pathways associated with cell cycle control.

**Table 2:** Twenty most significantly enriched (FDR < 0.05) GeneGo pathways among differentially expressed genes after miR-7 (A) or miR-128 (B) transfection into HEY cells

**A.**	
**Pathways enriched among miR-7 modulated genes**	**p-value**
Cell adhesion_Chemokines and adhesion	1.31E-07
Cell cycle_Regulation of G1/S transition (part 1)	8.68E-07
Cell adhesion_Ephrin signaling	3.96E-06
Development_EGFR signaling pathway	1.02E-05
Development_ERBB-family signaling	1.13E-05
Development_WNT signaling pathway. Part 1. Degradation of beta-catenin in the absence WNT signaling	1.42E-05
Development_VEGF-family signaling	1.68E-05
**Cytoskeleton remodeling_Cytoskeleton remodeling**	3.13E-05
**Neurophysiological process_Receptor-mediated axon growth repulsion**	3.43E-05
Proteolysis_Putative ubiquitin pathway	3.44E-05
Development_TGF-beta-dependent induction of EMT via RhoA, PI3K and ILK.	4.05E-05
Cell adhesion_Plasmin signaling	4.85E-05
Development_TGF-beta-dependent induction of EMT via SMADs	4.85E-05
Transport_RAB5A regulation pathway	5.75E-05
**Cytoskeleton remodeling_TGF, WNT and cytoskeletal remodeling**	7.29E-05
Cell cycle_Regulation of G1/S transition (part 2)	7.30E-05
Apoptosis and survival_HTR1A signaling	7.54E-05
Development_Regulation of epithelial-to-mesenchymal transition (EMT)	7.67E-05
Translation _Regulation of EIF2 activity	1.01E-04
Cell adhesion_ECM remodeling	1.01E-04
**B.**	
**Pathways enriched among miR-128 modulated genes**	**p-value**
Cell cycle_The metaphase checkpoint	1.01E-11
**Cytoskeleton remodeling_TGF, WNT and cytoskeletal remodeling**	1.29E-09
Cell cycle_Role of Nek in cell cycle regulation	1.77E-09
Transport_Clathrin-coated vesicle cycle	4.15E-09
Cell cycle_Initiation of mitosis	4.37E-09
Immune response_Histamine H1 receptor signaling in immune response	1.78E-07
Cell cycle_Role of APC in cell cycle regulation	1.79E-07
Cell cycle_Spindle assembly and chromosome separation	2.75E-07
**Cytoskeleton remodeling_Cytoskeleton remodeling**	2.98E-07
Proteolysis_Role of Parkin in the Ubiquitin-Proteasomal Pathway	3.68E-07
Cytoskeleton remodeling_Neurofilaments	6.14E-07
Cell cycle_Chromosome condensation in prometaphase	8.18E-07
Immune response_Gastrin in inflammatory response	1.85E-06
**Neurophysiological process_Receptor-mediated axon growth repulsion**	2.69E-06
Translation_Non-genomic (rapid) action of Androgen Receptor	3.55E-06
Development_PIP3 signaling in cardiac myocytes	4.80E-06
Cytoskeleton remodeling_Role of Activin A in cytoskeleton remodeling	5.34E-06
Apoptosis and survival_Apoptotic Activin A signaling	5.76E-06
Apoptosis and survival_BAD phosphorylation	6.54E-06
Immune response_Fc epsilon RI pathway	7.40E-06

The pathways that are enriched among differentially expressed genes after both miR-7 and miR-128 transfection are highlighted in bold.

Of the 252 pathways (not additive due to overlap) significantly over-represented among genes differentially expressed after miR-7 or miR-128 transfection, only 71 pathways were found to overlap (including the EGFR pathway). Among the 20 most significantly enriched pathways only 3 overlapped. Among genes down-regulated following miR-7 transfection most pathways are involved in development (cell adhesion, various signaling pathways, *etc.*), while among those down-regulated following miR-128 transfection most pathways are involved in cell cycle regulation (Figure 5C and 5D, Additional file 1: Table S13 and Additional file 1: Table S14) [Note a similar analysis of up-regulated genes revealed only 1 pathway enriched for miR-7 and 70 pathways enriched for miR-128 (Additional file 1: Table S15 and Additional file 1: 1S16)].

### Experimental assays of cell adhesion and cell cycle control in miR-7 and miR-128 transfected cells are consistent with predictions from the gene expression analyses

In an effort to experimentally test the validity of the computationally predicted consequences of miRNA transfection on cell function, we conducted assays to monitor the relative changes in cell adhesion and in cell cycle control in HEY cells transfected with miR-7 and miR-128, respectively.

**Figure 6:**
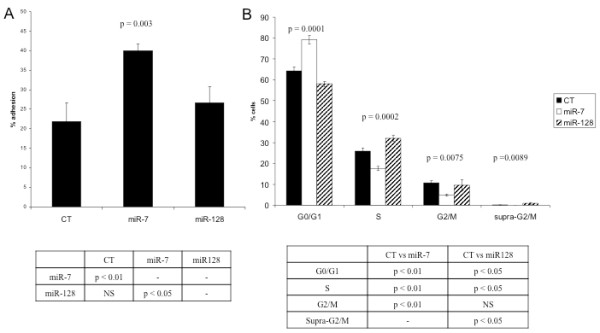
**Experimental validation that miR-7 transfection induces changes in cell adhesion while miR-128 transfection induces changes in cell cycle control.** (**A**) Percent adhesion for miR-7, miR-128 and negative control (CT) transfected Hey cells to basement membrane extract (BME). Cells were labeled with 2 μM Calcein AM for 1 hour and 20,000 labeled cells were seeded in BME coated 96-well plate. Each assay was carried out in triplicate for an adhesion period of 1 hour and 30 minutes. The results demonstrate a significant increase in cell adhesiveness in miR-7 transfected cells relative to negative controls. No significant difference in adhesiveness was observed between miR-128 transfected cells relative to negative controls. Each column represents the mean of three individual experiments. Error Bars = standard deviation. (**B**) Distribution of miR-7, miR-128 and negative control (CT) transfected Hey cells in specific phases of the cell cycle. MiR-7-transfected cells display a significant increase in the proportion of cells in G0/G1 and a significant decrease in the proportion of cells in the S and G2/M stages of the cell cycle relative to controls. Cells transfected with miR-128 display a significant decrease in the proportion of cells in the G0/G1 and an increase in the proportion of cells in S phase of cell cycle relative to controls. (P-values were determined by ANOVA for each phase of cell cycle for 3 separate transfections. Error bars = standard deviation).

The results of the cell adhesion assays are presented in Figure 6A and demonstrate a significant increase in cell adhesiveness in miR-7 transfected cells relative to negative controls. In contrast, no significant difference in adhesiveness was observed between miR-128 transfected cells relative to negative controls. These findings are consistent with the computational prediction that genes differentially expressed after miR-7 transfection will be most significantly enriched for genes involved in cell adhesion (p = 1.31×10^-7^, Table 2A). Also consistent with the results, genes differentially expressed after miR-128 transfection were not computationally predicted to be enriched for genes involved in cell adhesion (Table 2B).

Flow cytometry was used to monitor the proportion of cells in various stages of the cell cycle. The results presented in Figure 6B and Additional file 1: Figure S3 demonstrate that significant differences were detected among miR-7, miR-128 and control miRNA-transfected cells for each stage of the cell cycle. miR-7-transfected cells displayed a significant increase in the proportion of cells in G0/G1 and a significant decrease in the proportion of cells in the S and G2/M stages of the cell cycle relative to controls. This finding is consistent with the computational prediction that genes differentially expressed after miR-7 transfection are enriched for genes involved in “Cell cycle_Regulation of G1/S transition” (p = 8.68×10^-7^, Table 2A). In contrast, cells transfected with miR-128 displayed a significant decrease in the proportion of cells in the G0/G1 and an increase in the proportion of cells in S phase of cell cycle relative to controls. A higher proportion of miR-128 transfected cells in the supra-G2/M phase of the cell cycle may be indicative of incomplete chromosomal segregation due to alterations at the mitotic/metaphase checkpoint. These observations are consistent with the prediction that genes differentially expressed after miR-128 transfection are enriched for genes involved in “Cell cycle: The metaphase checkpoint” (p = 1.01×10^-11^, Table 2B) and “Cell cycle_Spindle assembly and chromosome separation” (p = 2.75×10^-7^, Table 2B).

In general, our experimental studies are consistent with the computational analysis of our microarray data indicating that miR-7 and miR-128 transfection modulates changes in the expression of genes predominately involved in distinct regulatory pathways.

## Discussion

Although the role of miRNAs and other non-coding RNAs in the regulation of cellular function is only beginning to be understood, the diagnostic and therapeutic potential of these regulatory RNAs is already widely recognized and accepted [30]. Indeed, a number of pre-clinical trials of miRNAs are currently in progress [10]. The initial results of these studies appear to be generally supportive of the potential utility of miRNAs in the early diagnosis of cancer [31,32], but significant questions remain with respect to their potential therapeutic value.

One of the challenges to the therapeutic use of miRNAs is technical in nature and deals with the problems of packaging and delivering miRNAs to cancer cells. Significant progress has been made in this area in recent years and all indications are that these technical challenges are not insurmountable [33,34]. The second challenge is more scientific in nature and centers around our current inability to precisely predict the molecular consequences of modulating miRNA levels on cell function. While the exogenous administration of miRNAs (or “antagomirs”) have been shown to be capable of effectively regulating targeted oncogenes and tumor suppressor genes in specific cellular contexts [30], the indirect effects of perturbing miRNA levels remains difficult to predict. Since this molecular unpredictability could translate into clinically unanticipated side-effects, it may significantly mitigate the intended therapeutic benefits [11]. It is generally agreed that increased therapeutic predictability of miRNAs and other regulatory RNAs will require a better understanding of the processes underlying the molecular function of these molecules *in vivo*[11].

In an initial effort to systematically dissect the molecular consequences of exogenously modulating levels of miRNA in ovarian cancer cells, we transfected two distinct human miRNAs, miR-7 and miR-128, into a well-characterized ovarian cancer cell line and monitored the consequent changes on endogenous levels of gene expression. These specific miRNAs were chosen because modulations in their levels of expression have both been previously correlated with ovarian cancer onset/progression [14].

We found that transfection of miR-7 and miR-128 resulted in significant changes in expression of hundreds to thousands of genes. Consistent with the fact that miR-7 is predicted to target fewer genes than miR-128 (using miRanda, TargetScan or PicTar), we found that the number of genes differentially expressed after miR-7 transfection was substantially less (approximately one-third) than that after miR-128 transfection. To determine if the large number of genes differentially expressed after the miRNA transfections were likely due to the direct regulatory action of the miRNAs, we computed the proportions of differentially expressed genes that are direct targets of miR-7 or miR-128 regulation as predicted by three different target prediction algorithms. On average, the proportion of differentially expressed genes predicted to be directly regulated by either miRNA was less than one-fifth indicating that most of the changes in gene expression induced by the miRNA transfections are indirect.

One recently described model to account for indirect effects of miRNA transfection postulates that transfected miRNAs may out-compete endogenous miRNAs for available RISCs [20] and consequently lead to up-regulation of targets of these endogenous miRNAs. This model may be relevant with respect to our miR-128 transfection experiment where the majority (70%) of differentially expressed genes displayed a significant increase in gene expression.

Consistent with the predictions of the RISC-competition model, we found that ~60% of the mRNAs enriched for binding sites of those miRNAs most highly expressed in pre-transfected HEY cells were significantly over-represented among mRNAs up-regulated after miR-128 transfection. While these results do not exclude alternative explanations, they are consistent with the hypothesis that at least some of the genes displaying increased expression after miR-128 transfection may be explained by the RISC-competition model. The model does not explain the >700 genes down regulated after miR-128 transfection or the fact that the vast majority of genes differentially expressed after miR-7 transfection were down-regulated. It is also not immediately apparent why the changes in global patterns of gene expression induced by the displacement of endogenous miRNAs should be functionally coordinated.

Perhaps the most commonly observed mechanism underlying coordinated changes in global patterns of gene expression involves the modulation of centralized or “hub” regulatory genes [35]. Hub genes have the potential to exert control on suites of downstream genes thereby inducing cascades of regulatory changes in patterns of gene expression [36]. Network analyses revealed that a substantial number of genes differentially expressed after miR-7 or miR-128 transfection are regulatory hubs capable of controlling many of the down-stream genes that were differentially expressed after miR-7 and miR-128 transfection. These findings indicate that miRNAs can both directly and indirectly induce changes in cellular regulatory networks.

In general, our experiments demonstrate that exogenous expression of miRNAs in ovarian cancer cells induces regulatory changes far in excess of those on the predicted targets. From the clinical perspective, this begs the question as to whether miRNAs are appropriate agents for ovarian cancer therapy. Clearly, if the clinical intent is to precisely silence a specific gene and if the collateral indirect effects induced by miRNAs are counter-therapeutic, the answer would be “no”. However, it is important to keep in mind that miRNAs have been evolving as essential components of the eukaryotic regulatory system for millions of years. Thus, the network of regulatory effects exerted by miRNAs is unlikely to be random and indeed may eventually provide clinicians with a strategy to treat ovarian cancer cells from a systems rather than a single gene perspective.

Our pathway enrichment analysis of the pathways most significantly affected by miR-7 and miR-128 transfection predicts a significant difference in the functional consequence of exogenous perturbations in levels of these two miRNAs. The pathways most significantly affected by miR-7 transfection are predicted to be involved with G1/S-phase cell cycle control as well as cell adhesion and other developmental networks previously associated with epithelial-mesenchymal transitions (EMT) and other processes linked with metastasis [37]. In contrast, the pathways most significantly affected by miR-128 transfection are more focused on mitotic-phase cell cycle control and other processes commonly linked with cellular replication [38]. Experimental analyses of cell adhesion and cell cycle control of cells transfected by miR-7 and miR-128 respectively were found to be remarkably consistent with these computational predictions. Although both sets of functions are generally characteristic of ovarian cancer cells, particular pathways may be relatively more important in particular tumors.

The therapeutic impact of individual miRNA treatments will ultimately be evaluated at the clinical level. However, the more we can learn about the processes underlying functional specialization of different families of miRNAs in experimentally controlled conditions, the better positioned we will be to interpret the significance of changes of these regulatory RNAs in individual tumors and to rationally select specific miRNAs for possible use in targeted cancer therapy.

## Conclusions

In summary, we have determined that the transfection of miRNAs into ovarian cancer cells results in changes in global patterns of gene expression. We have additionally shown that these changes in the expression are not random but can be explained, in large measure, by molecular models derived from our current understanding of miRNA function. Clearly, additional studies will be required to further evaluate the validity of these and other explanatory models in various classes of ovarian and other types of cancer cells and other families of miRNAs. Nevertheless, the results of our present studies indicate that while the molecular regulatory mechanisms underlying miRNA functions *in vivo* are extremely complex, they are not intractable. Validated models that can be used today to retrospectively explain the impact of exogenously expressed miRNAs, may be used in the future to prospectively design optimal system-wide strategies for the effective treatment of ovarian and other types of cancer.

## Methods

### Cell culture and siRNA/miRNA transfection

HEY cells were provided (July, 2005) by Gordon B. Mills (University of Texas, M. D. Anderson Cancer Center), freshly recovered from liquid nitrogen (<6 months) and were cultured according to methods described in [15]. Briefly, the cells (1.5 × 10^5^/well) were seeded on six-well plates in RPMI 1640 (Mediatech, Manassas, VA) supplemented with 10% v/v heat-inactivated fetal calf serum (Invitrogen, Carlsbad, CA), 2 mM L-glutamine (Mediatech), 10 mM HEPES buffer (Mediatech), penicillin (100 U/mL), and streptomycin (100 μg/mL) and allowed to adhere overnight at 37°C in a 5% CO_2_ atmosphere. The following day after washing with PBS and replacing the growth medium with reduced-serum Opti-MEM (Invitrogen), cells were transfected with hsa-miR-7 miRIDIAN mimic (cat # C-300546), hsa-miR-128 miRIDIAN mimic (cat # C-300597), miRIDIAN miRNA mimic negative control #1 (*C. elegans* miRNA cel-miR-67, with minimal sequence identity in humans, cat # CN-001000)), ON-TARGET *plus* siRNA EGFR (cat # L-003114), ON-TARGET *plus* siRNA RELA (cat # L-003533) or ON-TARGET *plus* non-targeting siRNA negative control (siNC) (cat # D-001810) (Thermo Fisher Scientific, Lafayette, CO) using Lipofectamine 2000 (Invitrogen) according to the manufacturer's instructions at a final concentration of 25nM (or left untransfected for naïve cells). All transfections were carried out in duplicates or triplicates. The cells were incubated with the transfection medium for 4 hours, washed and allowed to grow in growth medium (RPMI 1640) for 44 hours before RNA/protein isolation. Transfection efficiency was estimated from the relative knock-down of EGFR for miR-7 and the EGFR siRNA, and from the knock-down of BMI1 for miR-128 based on the manufacturer’s recommendations for siRNA/miRNA (Thermo Fisher Scientific).

### RNA isolation and whole genome microarray

Total RNA was isolated using the RNeasy Mini RNA isolation kit (QIAGEN, Valencia, CA). The integrity of the RNA was verified using an Agilent 2100 Bioanalyzer (1.8-2.0; Agilent Technologies, Palo Alto, CA). mRNAs were converted to double stranded (ds)-cDNA and amplified using Applause 3’-Amp System (NuGen, San Carlos, CA). This cDNA was fragmented and biotin labeled using the Encode Biotin Module (NuGen), hybridized to Affymetrix HG-U133 Plus 2.0 oligonucleotide arrays and analyzed with a GeneChip Scanner 3000 (Affymetrix, Santa Clara, CA).

### RNA isolation and miRNA microarray

Total RNA was isolated from two independent samples of pre-transfected (naïve) HEY cells using the mirVana miRNA isolation kit according to the manufacturer's instructions (Applied Biosystems, Foster City, CA). The quantity and size of small RNAs was verified using an Agilent 2100 Bioanalyzer (Agilent Technologies). Small RNAs were labeled with Genisphere FlashTag HSR Biotin RNA labeling kit (Genisphere, Hatfield, PA) followed by hybridization with GeneChip miRNA Array chips (Affymetrix) according to the manufacturer's instructions. The chips were washed and then scanned with a GeneChip Scanner 3000 (Affymetrix). Microarray analyses were performed on 3 independent replicates of negative control transfected cell samples (miR-NC), 3 independent replicates of miR-7 transfected cell samples and 2 independent replicates of miR-128 transfected cell samples. Raw data in the form of CEL files were produced by the Affymetrix GeneChip Operating System (GCOS) software.

### Quantitative (real-time) PCR (qPCR)

Total RNA (1–5 μg) extracted from cells was converted to cDNA using the Superscript III First Strand Synthesis System (Invitrogen). cDNA was then purified using the Qiagen PCR purification kit following the manufacturer’s instructions. qPCR experiments were carried out for the *EGFR, BMI1* and *GAPDH* genes using iQ SYBR Green Supermix (Bio-Rad, Hercules, CA). The sequence specific primers used for qPCR are as described in [39] for *EGFR* and in [40] for *GAPDH*. The sequences of the *BMI1* primers were as follows, BMI1-forward: ACTTCATTGATGCCACAACC, BMI1-reverse: CAGAAGGATGAGCTGCATAA, and were obtained from the qPCR primer database RTPrimerDB [41]. All reactions were optimized with non-template controls, and -RT (no reverse transcriptase) controls prior to experiment.

All qPCR reactions were carried out using at least 3 technical replicates and 3 biological replicates on the CFX96 Real Time PCR detection system (Bio-Rad). Expression values were normalized to the endogenous control GAPDH. Relative fold change of target RNA level between transfection groups was determined by the ΔΔCt method. Statistical significance was determined using the pair-wise fixed reallocation randomization test in the Relative Expression Software Tool (REST 2008; [42]).

### Cell cycle assays

Hey cells were transfected with miR-7, miR-128 or negative control miRNA in 3 independent transfection experiments, and the distribution of the transfected cells in various phases of the cell cycle was determined by flow cytometry based on cellular DNA content as previously described [43]. Equal numbers of transfected cells were used for DNA staining and FACS analysis (2 x10^6^ cells for each independent experiment). DNA content histograms were deconvoluted using the Watson Pragmatic model implemented in FlowJo 7.6 software (Tree Star Inc., Ashland, OR). Each assay was performed 3 times with independently transfected cells. Statistical significance of differences among proportions of cells in specific phases of the cell cycle was tested by ANOVA followed by Tukey’s Multiple Comparison post-test (p < 0.05).

### Cell adhesion assay

Cell adhesion assays were performed using the CultreCoat BME 96 Well Cell Adhesion Assay Kit (Trevigen, Gaithersburg, MD) according to the manufacturer's instructions. Briefly, basement membrane extract (BME) coated 96-well plates were rehydrated for two hours. Hey cells transfected with miR-7 or with negative control miRNA were labeled with 2 μM Calcein AM for 1 hour and then harvested and washed once with PBS. Cells were suspended in adhesion buffer to a density of 200,000 cells/ml. 100 μl of suspended cells were added to triplicate wells and incubated at 37°C for 1 hour and 30 minutes. Fluorescence was read at 485 nm excitation/520 emission (Total RFU). Cells were washed twice with wash buffer and fluorescence read again (RFU after wash). Percent adhesion was computed as (RFU after wash - background) / (Total RFU - background) × 100.

### Immunoblotting

Immunoblotting for EGFR and β-Actin was performed as described previously [44] with the following modifications. The blots were incubated for 2 hours with goat anti-rabbit IgG (1:2000) linked to fluorescein (FITC), or for 1 hour with donkey antimouse IgG (1:5000) linked to phycoerythrin (PE) secondary antibodies at room temperature (Southern Biotech, Birmingham, AL; primary anti-EGFR antibodies are described in [44]). Bands were visualized using a Typhoon 9400 Imager (GE Healthcare, Piscataway, NJ) followed by densitometric analysis using the ImageQuant TL Software (GE Healthcare).

### Microarray data analysis

miRNA microarray data were analyzed using miRNA QC Tool software (Version 1.0.33.0; Affymetrix). Probesets with “FALSE” call in all samples were removed prior to statistical analysis.

mRNA microarray data were analyzed using the Expression Console software (Affymetrix) and Bioconductor tools [45] written in the R statistical programming language (http://www.r-project.org). Normalization was performed using MAS 5.0, PLIER (Expression Console) and GCRMA (R). The log_2_ transformed expression values from MAS 5.0 were analyzed for Affymetrix “Present/Absent” calls using Spotfire DecisionSite for Microarray Analysis (DSMA). Probesets with “Absent” call in all samples were removed from analysis. Average probeset intensities for each group was calculated based on the log_2_ transformed values from PLIER and then filtered with DSMA to include only those probesets with a fold change ≥ 1.4. The false discovery rate (FDR) for each probeset was calculated from the log_2_-transformed values after GCRMA normalization using the SAM algorithm [46]. Differentially expressed probesets were identified using a threshold 5% FDR correction, a fold change ≥ 1.4 and at least “Present/Marginal” call in one sample. These three different filtering approaches were used based on previous recommendations [47] and the combination of all three was used to achieve the most stringent filtering.

All microarray data are MIAME compliant and have been submitted to GEO under the accession nos. GSE29126 and GSE27431.

### Pathway enrichment analysis, identification of hub genes and network building

After identification of DEGs, Pathway Maps analyses were carried out using GeneGo (http://www.GeneGo.com/) gene ontology software. Hub genes were identified using the GeneGo interactome analyses with the ‘Significant interactions within sets’ algorithm. In all cases significance was based on FDR ≤ 0.05. Networks were built using functional and binding interactions unless transcriptional regulation is specified.

### miRNA target download

The miRNA targets predictions based on miRanda, TargetScan and PicTar were downloaded from http://www.microrna.org (August 2010 release) [48,49], http://www.targetscan.org[19] and from http://www.pictar.org[18] respectively. “Good” mirSVR score refers to miRNA targets with < −0.1 score, and “non-good” mirSVR score refers to targets with > −0.1 score obtained from the support vector regression algorithm mirSVR, available with miRanda predictions from http://www.microrna.org.

### miRNA target enrichment analysis among up-regulated genes

Gene set enrichment analysis for predicted miRNA targets among up-regulated genes was carried out using the web interface of Genomica (http://genomica.weizmann.ac.il/, accessed July 7, 2010) [23] using default settings. Probeset IDs of up-regulated genes were uploaded onto the Genomica server for analysis and enrichment was performed against the ‘Human MicroRNA RNA’ gene set with FDR ≤ 0.05. When only a miRNA family is identified (without specifying the identity of the paralog) all members of the family were considered (*e.g*., hsa-miR-146a and hsa-miR-146b were both considered when Genomica identified hsa-miR-146).

## Competing interests

The authors declare that they have no competing interests.

## Authors’ contributions

SWS and JFM conceived of the study, participated in its design and coordination, analysis and interpretation of the data, and drafted the manuscript. LVM carried out the microarray experiments. CGH assisted in analysis of the microarray data. LW conducted and analyzed the results of the cell adhesion experiments. RM conducted and analyzed the results of the cell cycle experiments. LDW assisted in coordination of the study and contributed to the drafting of the manuscript. All authors read and approved the final manuscript.

## Pre-publication history

The pre-publication history for this paper can be accessed here:

http://www.biomedcentral.com/1755-8794/5/33/prepub

## Supplementary Material

Additional file 1 **Figure S1.** Confirmation of successful miR-128 transfection into HEY cells. **Figure S2.** Confirmation of EGFR down-regulation by siRNA. **Figure S3.** Deconvolution of DNA content histograms for Hey cells .**Table S1.** Differentially expressed genes in miR-7 transfected HEY cells. **Table S2.** Differentially expressed genes in miR-128 transfected HEY cells. **Table S3.** Differentially expressed genes in siRNA transfected HEY cells. **Table S4.** miRNA target enrichment analysis for up-regulated genes after miR-128 transfection. **Table S5.** Microarray expression levels of pre-transfected HEY cell miRNAs. **Table S6.** miRNA target enrichment analysis for up-regulated genes following miR-7 transfection. **Table S7.** Hub genes and their targets affected by miR-7 in HEY cells. **Table S8.** Hub genes and their targets affected by miR-128 in HEY cells. **Table S9.** Differentially expressed genes that are targeted by NF-κB or IL-1 Beta. **Table S10.** Direct downstream targets of Caveolin-1 and SMAD2. **Table S11.** GeneGo pathway maps following miR-7 transfection into HEY cells. **Table S12.** GeneGo pathway maps following miR-128 transfection into HEY cells. **Table S13.** GeneGo pathway maps for down-regulated genes following miR-7 transfection into HEY cells. **Table S14.** GeneGo pathway maps for down-regulated genes following miR-128 transfection into HEY cells. **Table S15.** GeneGo pathway maps for up-regulated genes following miR-7 transfection into HEY cells. **Table S16.** GeneGo pathway maps for up-regulated genes following miR-128 transfection into HEY cells. Click here for file
