# Supplementary material for: The effects of MicroRNA transfections on global patterns of gene expression in ovarian cancer cells are functionally coordinated
Source: BMC Med Genomics. 2012 Aug 1;5:33. doi: 10.1186/1755-8794-5-33 (PMC3481362; doi:10.1186/1755-8794-5-33)
Supplement: Additional file 1 — Figure S1. Confirmation of successful miR-128 transfection into HEY cells. Figure S2. Confirmation of EGFR down-regulation by siRNA. Figure S3. Deconvolution of DNA content histograms for Hey cells .Table S1. Differentially expressed genes in miR-7 transfected HEY cells. Table S2. Differentially expressed genes in miR-128 transfected HEY cells. Table S3. Differentially expressed genes in siRNA transfected HEY cells. Table S4. miRNA target enrichment analysis for up-regulated genes after miR-128 transfection. Table S5. Microarray expression levels of pre-transfected HEY cell miRNAs. Table S6. miRNA target enrichment analysis for up-regulated genes following miR-7 transfection. Table S7. Hub genes and their targets affected by miR-7 in HEY cells. Table S8. Hub genes and their targets affected by miR-128 in HEY cells. Table S9. Differentially expressed genes that are targeted by NF-κB or IL-1 Beta. Table S10. Direct downstream targets of Caveolin-1 and SMAD2. Table S11. GeneGo pathway maps following miR-7 transfection into HEY cells. Table S12. GeneGo pathway maps following miR-128 transfection into HEY cells. Table S13. GeneGo pathway maps for down-regulated genes following miR-7 transfection into HEY cells. Table S14. GeneGo pathway maps for down-regulated genes following miR-128 transfection into HEY cells. Table S15. GeneGo pathway maps for up-regulated genes following miR-7 transfection into HEY cells. Table S16. GeneGo pathway maps for up-regulated genes following miR-128 transfection into HEY cells. [file 1755-8794-5-33-S1.pdf]

Effects of MicroRNA Transfections on Global Patterns of Gene Expression are Functionally Coordinated

Shubin W. Shahab[1,2], Lilya V. Matyunina[1,2,3], Christopher G. Hill[1,2], Lijuan Wang[1,2], Roman Mezencev[1,2], L. DeEtte Walker[1,2,3] and John F. McDonald[1,2,3]

[1]School of Biology, Georgia Institute of Technology, Atlanta, GA, 30332, USA, [2]Parker H. Petit Institute for Bioengineering and Bioscience, Georgia Institute of Technology, Atlanta, GA, 30332, USA, [3]Ovarian Cancer Institute, Atlanta, GA, 30342, USA.

Supplementary Information

Table of Content

Supplementary Figures **Page**

Figure 1. Confirmation of successful miR-128 transfection into HEY cells..........................3

Figure 2. Confirmation of EGFR down-regulation by siRNA..4

Figure 3. Deconvolution of DNA content histograms for Hey cells......................................5

Supplementary Tables

Table 1. Differentially expressed genes in miR-7 transfected HEY cells..............................6

Table 2. Differentially expressed genes in miR-128 transfected HEY cells..........................41

Table 3. Differentially expressed genes in siRNA transfected HEY cells.............................107

Table 4. miRNA target enrichment analysis for up-regulated genes after miR-128 transfection...108

Table 5. Microarray expression levels of pre-transfected HEY cell miRNAs......................117

Table 6. miRNA target enrichment analysis for up-regulated genes following miR-7 transfection...139

Table 7. Hub genes and their targets affected by miR-7 in HEY cells................................140

Table 8. Hub genes and their targets affected by miR-128 in HEY cells............................151

Table 9. Differentially expressed genes that are targeted by NF-κB or IL-1 Beta..............250

Table 10. Direct downstream targets of Caveolin-1 and SMAD2.....................................251

Table 11. GeneGo pathway maps following miR-7 transfection into HEY cells................252

Table 12. GeneGo pathway maps following miR-128 transfection into HEY cells............255

Table 13. GeneGo pathway maps for down-regulated genes following miR-7 transfection into HEY cells...262

Table 14. GeneGo pathway maps for down-regulated genes following miR-128 transfection into HEY cells...266

Table 15. GeneGo pathway maps for up-regulated genes following miR-7 transfection into HEY cells...268

Table 16. GeneGo pathway maps for up-regulated genes following miR-128 transfection into HEY cells...269

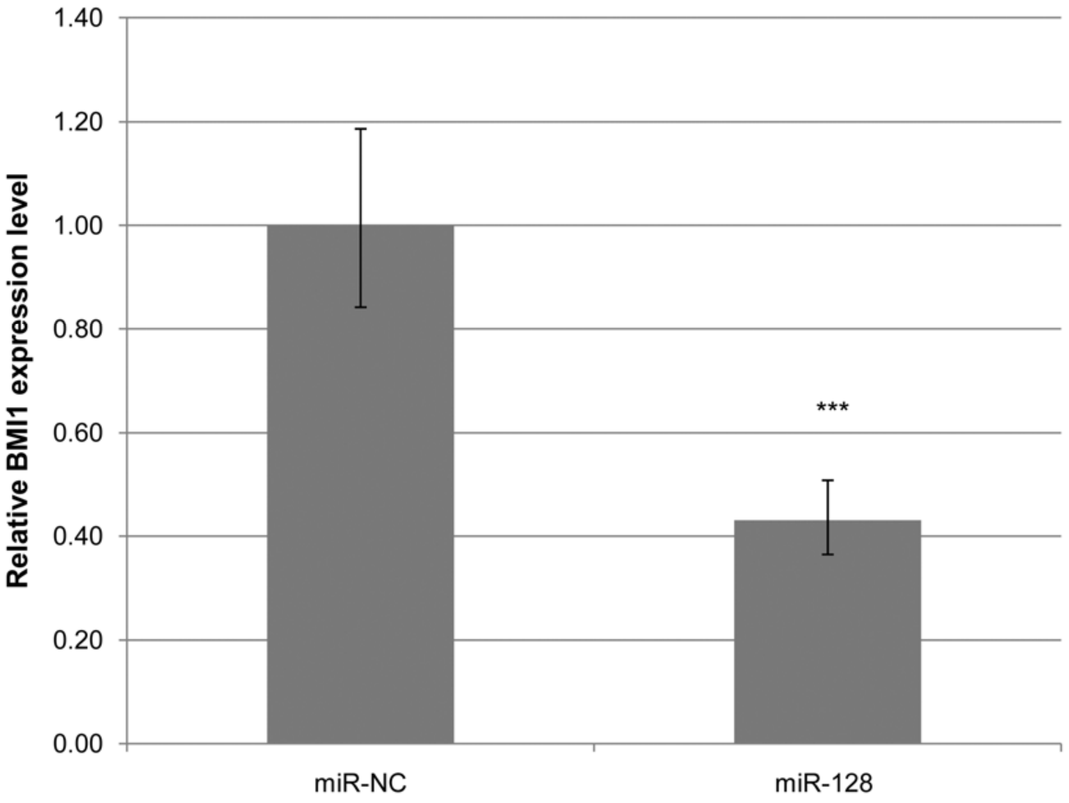

Supplementary Figure 1. Confirmation of successful miR-128 transfection into HEY cells. Successful transfection of miR-128 was confirmed by assaying the relative down-regulation of its validated target BMI1. Here we confirmed successful transfection of the miRNA into HEY cells by measuring BMI1 RNA level using qPCR following transfection of either miR-NC or miR-128 into HEY cells. Transfection of miR-128 down-regulated BMI1 by ~60 % relative to miR-NC. *** $p < 0.005$.

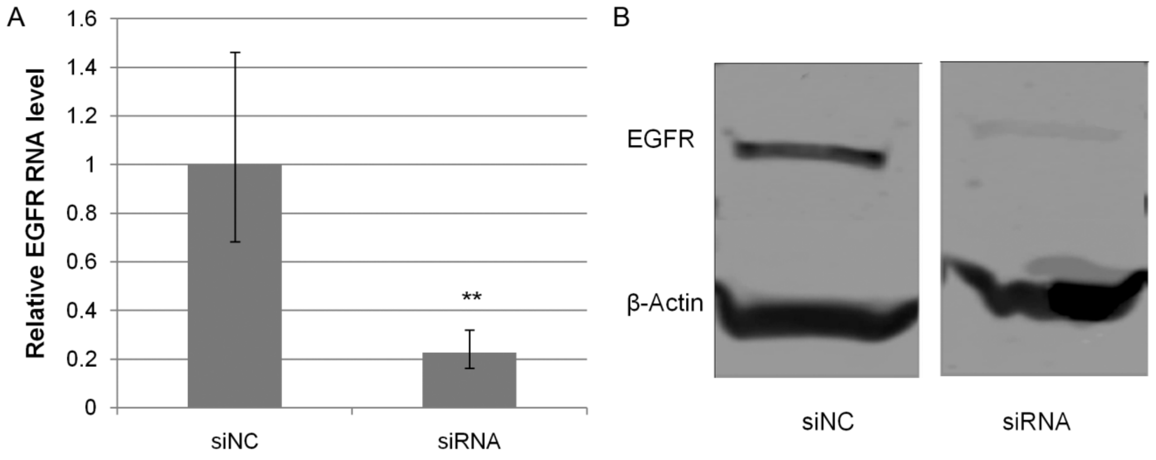

Supplementary Figure 2. Confirmation of EGFR down-regulation by siRNA. (A) Relative EGFR RNA levels determined by qPCR following transfection of EGFR siRNA (siRNA) or negative control siRNA (siNC) in HEY cells using GAPDH as endogenous control. Error bars show standard deviations from the mean based on RNA levels of 3 independent biological replicates. Statistical analysis was done using randomization with the help of REST 2008 software with at least 1000 iterations. ** $p < 0.05$. (B) Representative image of Western blot analysis of EGFR protein levels after transfection with EGFR siRNA or negative control siRNA (siNC). β-Actin is used as loading control for the immunoblot.

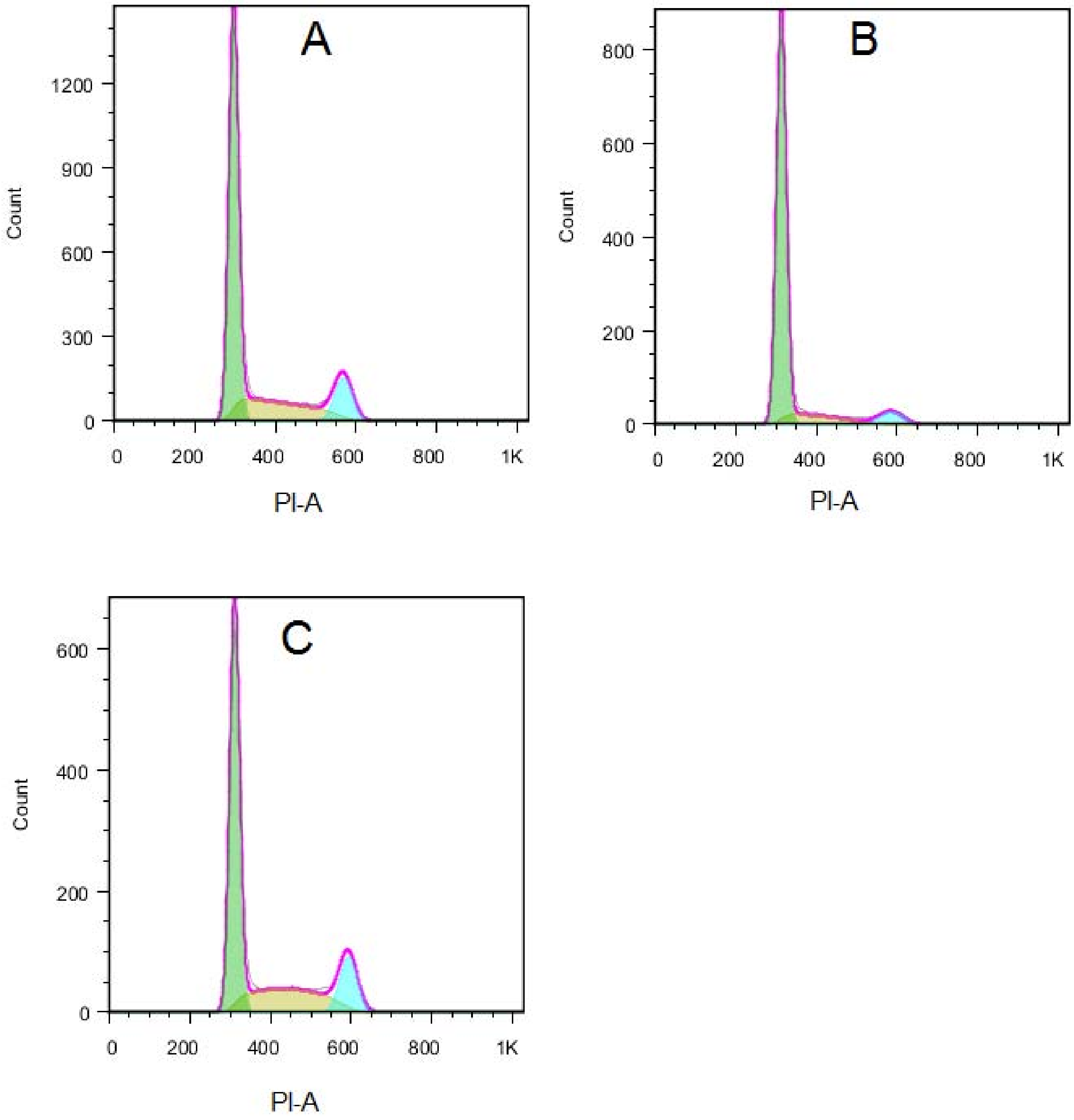

Supplementary Figure 3. Deconvolution of DNA content histograms for Hey cells transfected with (A) negative control miRNA, (B) miR-7, and (C) miR-128 by the Watson Pragmatic model. Green, yellow and cyan areas correspond to cells in G0/G1, S and G2/M phases of the cell cycle, respectively. PI-A: fluorescence intensity of propidium iodide (CV<6% for all G0/G1 peaks).

Supplementary Table 1. Differentially expressed genes in miR-7 transfected HEY cells. Genes differentially expressed (fold change ≥1.4, FDR ≤5%) in HEY ovarian cancer cells transfected with miR-7 compared to HEY cells transfected with negative control miRNA (miR-NC). 'Probeset ID' refers to Affymetrix HG-U133 Plus 2.0 probeset identifier. 'Gene Symbol' shows the official gene symbol for the corresponding Probeset ID. 'miR7-miR-NC' refers to the difference between average log_2 signal values (*i.e.* the log_2 of the ratio of signal values) of the miR-7 transfected group and the miR-NC transfected group. 'q-value (%)' shows the false discovery rate calculated using the SAM algorithm. miRanda predicted targets of miR-7 are listed in the column titled 'miR7tgts_M', TargetScan predicted targets of miR-7 are listed under the heading miR-7tgts_TS, and predicted PicTar targets of miR-7 are listed under the heading miR-7tgts_PT.

Probeset ID	Gene Symbol	miR7-miRNC	q-value(%)	miR-7 tgts_M	miR-7 tgts_TS	miR-7 tgts_PT
235348_at	ABHD13	-0.934249333	3.671565177	ABHD13		
207268_x_at	ABI2	0.503142	4.235240967		ABI2	
225098_at	ABI2	0.589023333	3.075356134		ABI2	
231321_s_at	ACER3	-0.942571333	0.587554882	ACER3		
222687_s_at	ACER3	-0.742688333	1.259835547	ACER3		
222688_at	ACER3	-0.648128667	1.259835547	ACER3		
227776_at	ACER3	-1.250583667	0	ACER3		
200793_s_at	ACO2	-1.451693667	1.164891419	ACO2		
201963_at	ACSL1	-0.630158	3.121720166			
209765_at	ADAM19	-1.124628667	1.79815454			
220866_at	ADAMTS6	-0.849758333	1.79815454			
237217_at	ADAMTSL1	-1.236878333	3.671565177			
224371_at	ADAMTSL1	-1.150468	2.485389855			

**Probeset ID**	**Gene Symbol**	**miR7-miRNC**	**q-value(%)**	**miR-7 tgts_M**	**miR-7 tgts_TS**	**miR-7 tgts_PT**
227113_at	ADHFE1	0.555978	1.79815454			
212500_at	ADO	-1.253696	0			
212502_at	ADO	-0.924597333	0.884241011			
225108_at	AGPS	-0.914143333	3.267378371	AGPS		
238855_at	AHNAK	-1.076481667	4.235240967	AHNAK		
226665_at	AHSA2	-0.623571333	3.121720166			
232810_at	AIG1	-1.203369	0.587554882	AIG1		
230520_at	AIG1	-0.606927	4.522982336	AIG1		
225342_at	AK3L1	-0.991323333	2.485389855			
204347_at	AK3L1	-0.664979333	4.235240967			
222862_s_at	AK5	0.533551	1.79815454			
210517_s_at	AKAP12	0.697011667	1.79815454			
1556841_a_at	ALDH1L2	-1.716318	0			
231202_at	ALDH1L2	-1.628134667	0			
1559393_at	ALDH1L2	-1.517756667	0			
204174_at	ALOX5AP	-0.74708	4.235240967			
202920_at	ANK2	-1.192487	0	ANK2		
202921_s_at	ANK2	-0.666925333	1.79815454	ANK2		
1554471_a_at	ANKRD13C	-0.912919333	1.79815454			
223418_x_at	ANKRD13C	-0.702445667	4.522982336			
229307_at	ANKRD28	-1.261974667	0	ANKRD28		
226025_at	ANKRD28	-0.9043	2.345471612	ANKRD28		
213035_at	ANKRD28	-0.779184	0.587554882	ANKRD28		
225735_at	ANKRD50	-0.929331333	1.202805955			
227660_at	ANTXR1	-0.839906667	3.121720166			
213454_at	APITD1	0.858506667	1.336284421			
211404_s_at	APLP2	-0.681913333	3.671565177	APLP2		
221087_s_at	APOL3	-1.624035333	0.884241011			

**Probeset ID**	**Gene Symbol**	**miR7-miRNC**	**q-value(%)**	**miR-7 tgts_M**	**miR-7 tgts_TS**	**miR-7 tgts_PT**
1557236_at	APOL6	-1.268496	0.587554882			
219716_at	APOL6	-0.982219333	1.259835547			
201096_s_at	ARF4	-0.6237	3.121720166	ARF4		ARF4
225171_at	ARHGAP18	-0.913321667	3.267378371			
225166_at	ARHGAP18	-0.798673667	1.79815454			
1560821_at	ARHGAP22	-0.719731	2.485389855			
211716_x_at	ARHGDIA	0.612537333	2.485389855			
202206_at	ARL4C	-0.804041333	1.259835547		ARL4C	ARL4C
219637_at	ARMC9	1.366374667	1.79815454			
206129_s_at	ARSB	-1.019458333	1.259835547	ARSB		
205047_s_at	ASNS	-1.40403	0			
225008_at	ASPH	-0.920992	4.235240967	ASPH		
218857_s_at	ASRGL1	1.82704	1.79815454			
207076_s_at	ASS1	-1.785531333	2.652723034			
222709_at	ATG7	-0.915890667	1.164891419			
209281_s_at	ATP2B1	-0.509994	4.235240967			
212136_at	ATP2B4	-1.032477667	4.235240967	ATP2B4		
212135_s_at	ATP2B4	-0.897951667	4.235240967	ATP2B4		
226302_at	ATP8B1	-0.588692	2.345471612			
205416_s_at	ATXN3	-0.548166667	4.235240967			
212475_at	AVL9	-0.728865667	2.345471612			
212471_at	AVL9	-0.684653333	1.164891419			
203188_at	B3GNT1	0.594666333	1.336284421			
206233_at	B4GALT6	0.565187	3.671565177			
206232_s_at	B4GALT6	0.875351667	2.485389855			
224335_s_at	BACE1	-1.073999	4.235240967	BACE1	BACE1	BACE1
228189_at	BAG4	-0.792539333	1.259835547			
227896_at	BCCIP	-0.838288	2.652723034	BCCIP		

Probeset ID	Gene Symbol	miR7-miRNC	q-value(%)	miR-7 tgts_M	miR-7 tgts_TS	miR-7 tgts_PT
218264_at	BCCIP	-0.762422333	4.235240967	BCCIP		
227143_s_at	BID	-0.692035	3.121720166			
205289_at	BMP2	-1.512856667	3.121720166			
209308_s_at	BNIP2	-0.643826667	2.345471612			
231960_at	BRWD1	-0.595151	4.235240967	BRWD1	BRWD1	
207485_x_at	BTN3A1	-1.359866333	0			
209846_s_at	BTN3A2	-1.026154333	0			
212613_at	BTN3A2	-1.009746	1.259835547			
204820_s_at	BTN3A2 /// BTN3A3	-0.925859	2.345471612			
38241_at	BTN3A3	-1.032930333	3.671565177			
225192_at	C10orf46	0.861095333	2.485389855			
227257_s_at	C10orf46	1.360522333	2.652723034			
52164_at	C11orf24	-1.032017667	1.164891419	C11orf24		
221534_at	C11orf68	-1.249860667	4.522982336	C11orf68		
204521_at	C12orf24	0.734646333	2.652723034			
229778_at	C12orf39	-1.621159333	4.522982336			
1553987_at	C12orf47	-0.709361	3.671565177			
1560426_at	C12orf55	-0.722036667	4.235240967			
219563_at	C14orf139	-0.858455	4.235240967			
212736_at	C16orf45	1.336602667	0			
230721_at	C16orf52	-0.570776333	2.485389855			
229071_at	C17orf100	0.913176333	3.075356134			
228452_at	C17orf39	-1.333441667	0.884241011	C17orf39		
220058_at	C17orf39	-0.676747	4.522982336	C17orf39		
238166_s_at	C17orf51	-0.807785333	4.235240967			
228146_at	C17orf51	-0.684836	4.235240967			
212055_at	C18orf10	-0.600639333	4.235240967	C18orf10		
1553338_at	C1orf55	-0.724618333	4.235240967	C1orf55		

Probeset ID	Gene Symbol	miR7-miRNC	q-value(%)	miR-7 tgts_M	miR-7 tgts_TS	miR-7 tgts_PT
224693_at	C20orf108	0.707014333	0.967238362			
224690_at	C20orf108	0.830417667	0.967238362			
1554818_s_at	C20orf12	0.634711333	3.671565177			
225313_at	C20orf177	-1.010335667	1.336284421	C20orf177		
1553311_at	C20orf197	-1.755140333	4.235240967			
223951_at	C21orf116	-0.715518667	3.121720166			
1559901_s_at	C21orf34	-0.874869333	1.259835547	C21orf34		
242565_x_at	C21orf57	1.240048333	0.488023728			
36552_at	C2CD3	0.561834667	1.79815454			
1553158_at	C3orf34	0.535698667	3.671565177			
1562953_s_at	C4orf12	-0.748492333	0			
227856_at	C4orf32	-0.756391333	4.235240967			
203738_at	C5orf22	-1.322204333	0	C5orf22	C5orf22	
1552660_a_at	C5orf22	-1.218008333	0	C5orf22	C5orf22	
219029_at	C5orf28	-0.755382333	1.259835547			
238635_at	C5orf28	-1.090266667	0.884241011			
220324_at	C6orf155	-0.708793333	1.259835547			
210410_s_at	C6orf26 /// MSH5	0.691512	3.075356134			
227000_at	C7orf41	0.663013667	2.345471612			
235866_at	C9orf85	0.911754333	3.267378371			
213688_at	CALM1	0.710425	1.336284421			
200623_s_at	CALM3	-1.321739	1.164891419	CALM3		
200622_x_at	CALM3	-1.054822333	4.522982336	CALM3		
200757_s_at	CALU	-0.83004	1.336284421	CALU	CALU	CALU
200756_x_at	CALU	-0.7102	2.485389855	CALU	CALU	CALU
200755_s_at	CALU	-0.592016667	3.267378371	CALU	CALU	CALU
214845_s_at	CALU	-0.566416667	4.235240967	CALU	CALU	CALU
224994_at	CAMK2D	-1.020761	0	CAMK2D	CAMK2D	

**Probeset ID**	**Gene Symbol**	**miR7-miRNC**	**q-value(%)**	**miR-7 tgts_M**	**miR-7 tgts_TS**	**miR-7 tgts_PT**
225019_at	CAMK2D	-0.86719	4.522982336	CAMK2D	CAMK2D	
210787_s_at	CAMKK2	-1.347015333	0	CAMKK2		
208838_at	CAND1	-0.800467	1.336284421	CAND1		CAND1
239771_at	CAND1	-0.884525333	1.259835547	CAND1		CAND1
213547_at	CAND2	-0.896886667	4.522982336			
208374_s_at	CAPZA1	-0.964162333	1.164891419	CAPZA1		CAPZA1
204950_at	CARD8	-0.792443333	4.235240967			
1554479_a_at	CARD8	-0.820563667	3.671565177			
206011_at	CASP1	-0.887094667	3.121720166	CASP1		
212097_at	CAV1	-0.892259333	2.485389855	CAV1		
225231_at	CBL	-0.769676	0.884241011			
243475_at	CBL	-0.742550667	4.522982336			
212816_s_at	CBS	-1.027109667	0.884241011	CBS		
1553972_a_at	CBS	-0.907156667	0.587554882	CBS		
1555920_at	CBX3	-0.713528	1.336284421			
1560599_a_at	CCDC123	0.677689	2.652723034			
1554512_a_at	CCDC123	0.976173	3.075356134			
232489_at	CCDC76	-0.913534333	2.485389855	CCDC76	CCDC76	CCDC76
218175_at	CCDC92	1.032029333	4.235240967			
205899_at	CCNA1	1.717766333	0	CCNA1		
200952_s_at	CCND2	-0.905452333	0			
211434_s_at	CCRL2	-1.093904667	4.235240967			
229900_at	CD109	-0.85771	3.121720166			
226545_at	CD109	-0.784628	2.652723034			
210743_s_at	CDC14A	0.848836333	1.336284421	CDC14A		
209288_s_at	CDC42EP3	-0.704293333	4.235240967			
224428_s_at	CDCA7	-0.766242333	2.485389855			
234932_s_at	CDCP1	-0.815387667	1.164891419			

Probeset ID	Gene Symbol	miR7-miRNC	q-value(%)	miR-7 tgts_M	miR-7 tgts_TS	miR-7 tgts_PT
224851_at	CDK6	-0.772117333	1.259835547			
209112_at	CDKN1B	-0.569535667	3.267378371			
212864_at	CDS2	0.750268	0.884241011			
226449_at	CEP120	-0.726862667	2.485389855			
224352_s_at	CFL2	-0.791701	0			
219270_at	CHAC1	-1.550980333	2.485389855	CHAC1		
228999_at	CHD2	-0.948332333	1.164891419			
226350_at	CHML	-0.694081667	4.235240967			
226314_at	CHST14	1.39496	0			
200998_s_at	CKAP4	-1.215276667	1.259835547	CKAP4	CKAP4	CKAP4
200999_s_at	CKAP4	-1.190297667	1.164891419	CKAP4	CKAP4	CKAP4
212306_at	CLASP2	-0.683096333	4.235240967	CLASP2	CLASP2	
211709_s_at	CLEC11A	-1.119912667	0.884241011			
201560_at	CLIC4	-0.584176667	3.671565177	CLIC4		
226017_at	CMTM7	-0.783440333	1.259835547			
202163_s_at	CNOT8	-1.345856333	0	CNOT8	CNOT8	CNOT8
202164_s_at	CNOT8	-1.266676667	1.202805955	CNOT8	CNOT8	CNOT8
202162_s_at	CNOT8	-0.735841	1.164891419	CNOT8	CNOT8	CNOT8
226751_at	CNRIP1	-1.187306333	0			
203073_at	COG2	-0.737239333	3.121720166			
211343_s_at	COL13A1	-1.633290333	0	COL13A1		
225293_at	COL27A1	-0.730513	4.235240967			
221729_at	COL5A2	-0.991497	1.202805955	COL5A2		
226934_at	CPSF6	-0.928021667	3.121720166			
201990_s_at	CREBL2	0.731282333	4.235240967			
209967_s_at	CREM	-0.603597333	3.267378371			
207630_s_at	CREM	-0.521365	2.652723034			
226656_at	CRTAP	-1.402645333	1.202805955			

Probeset ID	Gene Symbol	miR7-miRNC	q-value(%)	miR-7 tgts_M	miR-7 tgts_TS	miR-7 tgts_PT
1555889_a_at	CRTAP	-1.377131667	0			
227138_at	CRTAP	-1.153451333	4.522982336			
201380_at	CRTAP	-1.061394667	0			
1554464_a_at	CRTAP	-0.80016	2.485389855			
240221_at	CSNK1A1	-0.942355667	3.121720166			
229665_at	CSTF3	-0.793617	1.259835547			
208735_s_at	CTDSP2	-1.067301333	1.336284421		CTDSP2	
201904_s_at	CTDSPL	-1.043472667	1.336284421	CTDSPL		
206085_s_at	CTH	-0.906001667	1.164891419			
219080_s_at	CTPS2	-0.933936	3.267378371			
201370_s_at	CUL3	-0.823882667	4.522982336			
232466_at	CUL4A	-0.778637667	1.79815454			
203531_at	CUL5	-0.710311333	3.267378371	CUL5	CUL5	
203533_s_at	CUL5	-0.548173667	3.671565177	CUL5	CUL5	
204470_at	CXCL1	-1.523311333	0			
209774_x_at	CXCL2	-1.359100333	0	CXCL2		
219179_at	DACT1	-1.111032333	1.164891419			
200794_x_at	DAZAP2	-0.855724	3.121720166	DAZAP2		
218094_s_at	DBNDD2 /// SYS1 /// SYS1-DBNDD2	0.898224	2.652723034			
228106_at	DCAF16	-0.531252	4.522982336			
220172_at	DCAF17	-1.034532667	0.587554882			
224730_at	DCAF7	-1.500802	3.121720166	DCAF7		
222925_at	DCDC2	-0.855561	2.485389855			
204296_at	DCTN1	-0.958904	3.671565177			
243711_at	DDAH1	-0.60428	3.671565177			
209383_at	DDIT3 /// NR1H3	-0.761831	4.235240967			
202887_s_at	DDIT4	-2.187503667	0.884241011	DDIT4	DDIT4	DDIT4

Probeset ID	Gene Symbol	miR7-miRNC	q-value(%)	miR-7 tgts_M	miR-7 tgts_TS	miR-7 tgts_PT
205168_at	DDR2	-0.720583333	2.345471612			
227561_at	DDR2	-0.714136667	4.522982336			
225442_at	DDR2	-0.649201667	4.522982336			
224654_at	DDX21	-0.88558	4.235240967			
219641_at	DET1	-1.007442667	4.522982336	DET1		
202533_s_at	DHFR	0.634782667	4.522982336			
202532_s_at	DHFR	0.658043333	3.075356134			
1558342_x_at	DIXDC1	0.857792667	0			
214724_at	DIXDC1	0.886852	3.671565177			
1558340_at	DIXDC1	0.898065667	0			
220572_at	DKFZp547G183	-0.935759667	1.79815454			
230229_at	DLG1	-0.849842	1.79815454			
243938_x_at	DNAH5	1.165058667	0.884241011			
212817_at	DNAJB5	0.804281333	2.345471612			
205003_at	DOCK4	-0.844925667	2.652723034			
209691_s_at	DOK4	0.713829667	0.967238362			
203716_s_at	DPP4	-1.017578667	4.522982336			
201430_s_at	DPYSL3	0.933444333	0	DPYSL3		
225228_at	DRAM2	-0.821912	4.522982336			
203635_at	DSCR3	-0.654742667	4.522982336			
211493_x_at	DTNA	0.596403333	2.345471612	DTNA		
225415_at	DTX3L	-0.741281333	3.267378371			
219469_at	DYNC2H1	0.692893	2.652723034	DYNC2H1		
1561939_at	DYNC2H1	0.752620667	2.485389855	DYNC2H1		
225494_at	DYNLL2	1.000013	0.967238362			
241713_s_at	DYX1C1	1.064675	4.235240967			
211607_x_at	EGFR	-0.616986667	4.522982336	EGFR	EGFR	
209037_s_at	EHD1	-1.432172	4.522982336	EHD1	EHD1	

Probeset ID	Gene Symbol	miR7-miRNC	q-value(%)	miR-7 tgts_M	miR-7 tgts_TS	miR-7 tgts_PT
233660_at	EHD4	-1.571905	0			
1556607_at	EHD4	-1.166487333	3.267378371			
209536_s_at	EHD4	-0.87014	3.671565177			
228967_at	EIF1	-0.686682333	2.652723034			
201017_at	EIF1AX	-0.548122	4.235240967			
222576_s_at	EIF2C1	-0.564634333	4.235240967		EIF2C1	EIF2C1
224936_at	EIF2S3	-0.578356667	3.121720166	EIF2S3		
224935_at	EIF2S3	-0.542473333	3.121720166	EIF2S3		
214805_at	EIF4A1	-0.843098	3.121720166			
1555996_s_at	EIF4A2	-0.665303333	1.259835547			
31845_at	ELF4	0.839981	2.345471612			
203490_at	ELF4	0.652643667	2.652723034			
226099_at	ELL2	-0.859892667	3.121720166			
226982_at	ELL2	-0.576571667	4.522982336			
213895_at	EMP1	-1.271489667	1.336284421			
225078_at	EMP2	0.671409667	2.345471612			
212336_at	EPB41L1	0.729266333	3.075356134			
206114_at	EPHA4	-0.785929333	0	EPHA4		
210753_s_at	EPHB1	-1.588063	0	EPHB1		
230425_at	EPHB1	-1.49045	0	EPHB1		
211898_s_at	EPHB1	-1.189079667	3.121720166	EPHB1		
210385_s_at	ERAP1	-0.614447	3.267378371			
212580_at	ERAP1	-0.578651	4.522982336			
228131_at	ERCC1	-1.06454	1.79815454			
213365_at	ERI2	-0.641929333	3.267378371	ERI2		
221543_s_at	ERLIN2	-1.380031667	0	ERLIN2		
208958_at	ERP44	-0.761450333	0	ERP44		
224657_at	ERRFI1	-0.968132333	1.79815454			

Probeset ID	Gene Symbol	miR7-miRNC	q-value(%)	miR-7 tgts_M	miR-7 tgts_TS	miR-7 tgts_PT
201574_at	ETF1	0.560095333	3.267378371			
204774_at	EVI2A	-0.838887	4.235240967			
204603_at	EXO1	0.909993333	0.884241011			
214507_s_at	EXOSC2	-0.854102	2.485389855	EXOSC2	EXOSC2	
209527_at	EXOSC2	-0.607609333	4.522982336	EXOSC2	EXOSC2	
217490_at	EXOSC6	-0.613708667	1.336284421			
209537_at	EXTL2	-0.739207667	1.259835547			
203358_s_at	EZH2	0.515519667	3.267378371			
226905_at	FAM101B	-0.633648333	3.121720166			
223059_s_at	FAM107B	-0.674381	2.652723034		FAM17B	
223058_at	FAM107B	-0.669111	3.121720166		FAM17B	
1557129_a_at	FAM111B	1.175139333	3.671565177			
1557128_at	FAM111B	1.204887667	1.202805955			
1569024_at	FAM13A	-1.238234	0	FAM13A		
1569025_s_at	FAM13A	-0.939806667	0	FAM13A		
1557385_at	FAM161A	0.797169667	2.345471612			
212017_at	FAM168B	-0.856380333	3.121720166	FAM168B	FAM168B	
226752_at	FAM174A	-0.581110667	3.121720166			
243011_at	FAM55C	0.819736667	1.79815454			
235030_at	FAM55C	0.897649667	0.967238362			
243606_at	FAM55C	0.958509333	1.336284421			
203184_at	FBN2	-0.808324	4.522982336			
241736_at	FBXW2	-0.537070667	3.671565177		FBXW2	
229419_at	FBXW7	-0.681468	4.235240967			
1552721_a_at	FGF1	-1.803147667	0			
208240_s_at	FGF1	-1.712672667	0			
205117_at	FGF1	-1.349569	0.587554882			
210973_s_at	FGFR1	-0.907155667	4.522982336			

**Probeset ID**	**Gene Symbol**	**miR7-miRNC**	**q-value(%)**	**miR-7 tgts_M**	**miR-7 tgts_TS**	**miR-7 tgts_PT**
222956_at	FIGN	0.852729	0.967238362			
1558308_at	FLJ33297	-0.848847333	4.522982336			
1556033_at	FLJ39739	-1.138749	0			
232249_at	FMNL3	-0.743451667	3.671565177			
215245_x_at	FMR1	-0.516229667	4.522982336			
212464_s_at	FN1	-0.655773333	2.652723034			
211719_x_at	FN1	-0.63965	3.267378371			
216442_x_at	FN1	-0.62945	2.652723034			
210495_x_at	FN1	-0.62431	3.121720166			
213056_at	FRMD4B	-1.004611667	3.121720166	FRMD4B		
225464_at	FRMD6	-0.607033333	4.235240967			
208782_at	FSTL1	-0.821716667	1.79815454	FSTL1		
214211_at	FTH1	-0.831765333	3.267378371			
222811_at	FTSJD1	-0.750995	3.671565177			
214094_at	FUBP1	-1.357703	0			
214093_s_at	FUBP1	-0.576453667	1.336284421			
208841_s_at	G3BP2	-0.847398	4.235240967	G3BP2	G3BP2	G3BP2
221759_at	G6PC3	0.65483	3.267378371			
227428_at	GABPA	-0.638312333	4.235240967			
218313_s_at	GALNT7	-0.890303667	2.652723034	GALNT7		GALNT7
227517_s_at	GAS5	-0.730173333	1.79815454			
1560402_at	GAS5	-0.588273333	3.267378371			
225430_at	GATC	0.508744	4.522982336			
231577_s_at	GBP1	-1.154603	0.587554882			
202269_x_at	GBP1	-1.026829667	1.164891419			
223434_at	GBP3	-0.62089	4.235240967			
203765_at	GCA	-1.18384	4.235240967			
219821_s_at	GFOD1	-0.636643333	3.121720166			

**Probeset ID**	**Gene Symbol**	**miR7-miRNC**	**q-value(%)**	**miR-7 tgts_M**	**miR-7 tgts_TS**	**miR-7 tgts_PT**
205100_at	GFPT2	-0.669665667	1.336284421			
243502_at	GJC1	-0.612254	1.164891419	GJC1	GJC1	
228776_at	GJC1	-0.573069667	4.235240967	GJC1	GJC1	
214430_at	GLA	0.652549	2.652723034			
200681_at	GLO1	-0.909495667	3.671565177	GLO1		
221447_s_at	GLT8D2	-1.130248667	0			
235371_at	GLT8D4	-0.742504667	0			
215001_s_at	GLUL	-0.864536	2.652723034			
221628_s_at	GLYR1	0.671594667	1.202805955			
235233_s_at	GMEB1	-0.760359667	3.671565177			
235232_at	GMEB1	-1.000150333	3.267378371			
225420_at	GPAM	-0.886147	3.267378371	GPAM		
225424_at	GPAM	-0.793781	2.652723034	GPAM		
230369_at	GPR161	0.716365333	2.652723034			
209409_at	GRB10	-0.864713	2.345471612			
218469_at	GREM1	-1.651188	0	GREM1		
218468_s_at	GREM1	-1.632119333	0	GREM1		
226881_at	GRPEL2	-0.671769667	1.164891419			
225276_at	GSPT1	-1.136835333	0.884241011	GSPT1		
219770_at	GTDC1	0.549109	4.235240967			
221892_at	H6PD	-0.959188667	0.587554882			
226160_at	H6PD	-0.917473333	3.267378371			
222685_at	HAUS6	-0.853378667	1.164891419			
203821_at	HBEGF	-1.063623	2.652723034			
38037_at	HBEGF	-1.058676	3.121720166			
1568746_a_at	hCG_1654703	-2.137198	0			
238422_at	hCG_1811732	0.977125	1.233540637			
225012_at	HDLBP	-1.070912667	0	HDLBP	HDLBP	

Probeset ID	Gene Symbol	miR7-miRNC	q-value(%)	miR-7 tgts_M	miR-7 tgts_TS	miR-7 tgts_PT
233642_s_at	HEATR5B	-0.623326667	4.235240967	HEATR5B		
44783_s_at	HEY1	-1.550026667	0			
218839_at	HEY1	-1.070523	1.164891419			
1553402_a_at	HFE	-0.741104667	2.652723034			
223073_at	HIATL1	-1.112165	3.671565177	HIATL1		
212291_at	HIPK1	0.617595333	1.79815454	HIPK1		
202934_at	HK2	-1.224419	2.345471612	HK2		
209068_at	HNRPDL	-0.863323667	0			
205601_s_at	HOXB5	-0.758956	3.121720166	HOXB5		HOXB5
202557_at	HSPA13	-0.740381667	3.267378371			
200690_at	HSPA9	-0.594157	0.587554882			
203960_s_at	HSPB11	0.656703333	2.345471612			
219998_at	HSPC159	0.844315667	1.336284421			
215485_s_at	ICAM1	-0.851862333	3.121720166			
204569_at	ICK	-0.701909	4.522982336			
225615_at	IFFO2	0.825340333	1.202805955			
214453_s_at	IFI44	-1.689571	2.652723034			
222519_s_at	IFT57	0.664064333	0.967238362			
212143_s_at	IGFBP3	1.917721667	0.967238362			
39402_at	IL1B	-1.452849	0			
205067_at	IL1B	-1.467005333	0			
205227_at	IL1RAP	-1.550110667	0			
206693_at	IL7	-0.983392667	1.79815454			
205798_at	IL7R	-1.179825333	0			
211506_s_at	IL8	-1.644915	0			
202859_x_at	IL8	-2.790871667	1.79815454			
214493_s_at	INADL	0.605393	2.652723034			
213643_s_at	INPP5B	-0.884047	1.259835547	INPP5B		

Probeset ID	Gene Symbol	miR7-miRNC	q-value(%)	miR-7 tgts_M	miR-7 tgts_TS	miR-7 tgts_PT
235283_at	INTS6	-0.628273667	3.121720166			
200994_at	IPO7	-1.064822667	0	IPO7		
200995_at	IPO7	-0.993921667	3.121720166	IPO7		
200993_at	IPO7	-0.699516667	3.121720166	IPO7		
1554739_at	IPP	-0.561747333	4.522982336			
215130_s_at	IQCK	1.093483	0			
213392_at	IQCK	1.144875667	2.345471612			
204686_at	IRS1	-0.880808667	3.267378371	IRS1	IRS1	
235057_at	ITCH	-0.649007667	4.235240967	ITCH		ITCH
227792_at	ITPRIPL2	-1.271248667	1.79815454			
1568619_s_at	ITPRIPL2	-0.888665333	3.267378371			
225142_at	JHDM1D	-1.039806333	0.587554882		JHDM1D	
218418_s_at	KANK2	1.068694667	0.884241011			
212447_at	KBTBD2	-0.692199	2.485389855	KBTBD2	KBTBD2	
214595_at	KCNG1	-0.991521667	2.652723034			
212188_at	KCTD12	-0.78182	4.235240967			
218310_at	KCTD7 /// RABGEF1	-0.653316333	3.671565177			
201549_x_at	KDM5B	-0.662096333	3.267378371	KDM5B		
211202_s_at	KDM5B	-0.729085	0	KDM5B		
231031_at	KGFLP2	-1.521108	0			
212056_at	KIAA0182	0.584176667	2.652723034	KIAA0182	KIAA182	
202181_at	KIAA0247	-1.090836	3.267378371	KIAA0247	KIAA247	
212314_at	KIAA0746	-1.868745	0			
212311_at	KIAA0746	-1.410239	0			
230232_at	KIAA0746	-1.249311333	0.587554882			
1559601_at	KIAA2018	-0.937686	0.587554882		KIAA218	
227433_at	KIAA2018	-0.873415333	1.164891419		KIAA218	
224662_at	KIF5B	-0.59615	3.121720166	KIF5B		

**Probeset ID**	**Gene Symbol**	**miR7-miRNC**	**q-value(%)**	**miR-7 tgts_M**	**miR-7 tgts_TS**	**miR-7 tgts_PT**
220825_s_at	KIRREL	-0.772344	1.202805955			
225140_at	KLF3	-0.638833667	3.121720166			
225133_at	KLF3	-0.60824	4.235240967			
221841_s_at	KLF4	-1.294014667	0	KLF4	KLF4	KLF4
220266_s_at	KLF4	-1.064316333	1.259835547	KLF4	KLF4	KLF4
209255_at	KLHDC10	-0.670668333	2.345471612			
212882_at	KLHL18	-0.910570333	0.587554882			
218587_s_at	KTELC1	0.506082667	3.267378371			
209270_at	LAMB3	-0.580993333	4.522982336			
200771_at	LAMC1	-0.73233	2.652723034			
202267_at	LAMC2	-1.270863333	0			
223380_s_at	LATS2	0.597402667	1.336284421			
218604_at	LEMD3	-0.729039667	3.121720166	LEMD3	LEMD3	LEMD3
207170_s_at	LETMD1	-0.70346	3.671565177			
227771_at	LIFR	-0.661857	3.671565177			
218850_s_at	LIMD1	-0.927072333	2.652723034	LIMD1		
200706_s_at	LITAF	-1.526414	0			
200704_at	LITAF	-1.270465	0			
202674_s_at	LMO7	-0.662218	2.485389855			
227578_at	LOC100128191	0.956306667	1.336284421			
227406_at	LOC100129387	-0.579716333	3.671565177			
1558782_a_at	LOC100130557	-0.834155	3.267378371			
226526_s_at	LOC100130905	-1.078553	1.202805955			
227074_at	LOC100131564	-1.027196667	1.164891419			
1557783_at	LOC100133991	0.606983667	3.671565177			
229685_at	LOC100134937	0.952088667	2.485389855			
225785_at	LOC100289942 /// REEP3	-1.107265333	1.259835547			

**Probeset ID**	**Gene Symbol**	**miR7-miRNC**	**q-value(%)**	**miR-7 tgts_M**	**miR-7 tgts_TS**	**miR-7 tgts_PT**
243092_at	LOC100290367	-1.005412	4.522982336			
227136_s_at	LOC100292024	1.33364	3.075356134			
235191_at	LOC148189	-0.972376667	3.121720166			
230502_s_at	LOC149832	0.674781333	4.522982336			
1555847_a_at	LOC284454	-1.289552667	0			
233737_s_at	LOC284561	-1.723565667	0			
232689_at	LOC284561	-1.700749	0			
1556896_at	LOC284751	0.507893333	0.488023728			
235606_at	LOC344595	0.935697667	1.233540637	LOC344595		
239466_at	LOC344595	1.020400667	0.488023728	LOC344595		
235205_at	LOC346887	-0.706917667	2.652723034			
232001_at	LOC439949	-0.600382333	1.202805955			
1555858_at	LOC440944	-0.768911667	3.671565177			
227474_at	LOC654433	-0.820439	2.485389855	LOC654433		
228425_at	LOC654433	-0.713548333	4.522982336	LOC654433		
231247_s_at	LOC727820	-0.788250667	1.259835547			
227384_s_at	LOC727820	-0.773393667	1.259835547			
227383_at	LOC727820	-0.714555333	2.345471612			
1568763_s_at	LOC728613 /// PDCD6	-0.589180667	3.267378371			
214291_at	LOC729046 /// RPL17	-0.963140667	2.652723034			
231808_at	LOC729082	-0.778646	0	LOC729082		
228808_s_at	LOXL2	-1.743451667	0.587554882			
220816_at	LPAR3	-1.267253667	4.522982336			
213615_at	LPCAT3	-0.756959333	2.652723034			
1557360_at	LRPPRC	-1.338582667	3.121720166			
229231_at	LRRC37B	0.550703	4.522982336			
201932_at	LRRC41	-0.829756667	3.267378371			
218364_at	LRRFIP2	-0.764148333	4.235240967			

Probeset ID	Gene Symbol	miR7-miRNC	q-value(%)	miR-7 tgts_M	miR-7 tgts_TS	miR-7 tgts_PT
220099_s_at	LUC7L2	-0.918849333	0	LUC7L2		
212449_s_at	LYPLA1	-0.816624667	2.345471612	LYPLA1		
203897_at	LYRM1	-1.038915	2.485389855			
221311_x_at	LYRM2	0.715663	3.075356134			
226321_at	LYSMD3	-0.725042	4.235240967		LYSMD3	
226538_at	MAN2A1	-0.907624667	3.121720166			
205105_at	MAN2A1	-0.645298	3.671565177			
235103_at	MAN2A1	-0.593864333	3.121720166			
205447_s_at	MAP3K12	1.114845	0.884241011			
218582_at	MARCH5	-0.620779667	1.79815454	MARCH5		
215512_at	MARCH6	-0.647366333	1.202805955			
221047_s_at	MARK1	0.551930667	3.671565177			
200768_s_at	MAT2A	-1.299810333	1.202805955			
242260_at	MATR3	-1.638601	4.235240967			
1564907_s_at	MATR3 /// SNHG4	-0.883998333	0.884241011			
228938_at	MBP	0.907891667	0.967238362			
209087_x_at	MCAM	0.934544667	3.075356134			
211340_s_at	MCAM	0.990582333	1.79815454			
204058_at	ME1	-0.905962	4.522982336			
1555300_a_at	MED12L	0.951143	1.233540637			
215897_at	MED25	1.190249667	0.488023728			
214831_at	MED28	-0.624068333	3.671565177			
212830_at	MEGF9	-0.770316667	1.259835547	MEGF9	MEGF9	
212831_at	MEGF9	-0.678113	3.121720166	MEGF9	MEGF9	
225955_at	METRNL	-1.328199333	1.259835547			
1552312_a_at	MFAP3	-0.755696	0			
203102_s_at	MGAT2	-0.766612	3.121720166	MGAT2		
223974_at	MGC11082	-0.718998	2.485389855			

Probeset ID	Gene Symbol	miR7-miRNC	q-value(%)	miR-7 tgts_M	miR-7 tgts_TS	miR-7 tgts_PT
1560019_at	MGC11082	-0.811882667	3.121720166			
228235_at	MGC16121	-1.903165333	0			
229784_at	MGC16121	-1.791946333	0			
228040_at	MGC21881	0.621571667	2.652723034			
200899_s_at	MGEA5	-0.601610667	3.121720166			
211026_s_at	MGLL	-1.341348	3.121720166			
225102_at	MGLL	-1.248998	0			
55081_at	MICALL1	-0.718769667	3.671565177	MICALL1		
229437_at	MIR155HG	-0.842209667	1.164891419			
232291_at	MIR17HG	-1.230599333	0			
233636_at	MIR17HG	-1.048502667	1.164891419			
224917_at	MIR21	-1.244892333	0			
1570202_a_at	MKL2	0.802457667	3.267378371			
239468_at	MKX	-1.029428667	3.121720166			
215904_at	MLLT4	0.683659333	3.671565177			
202827_s_at	MMP14	-1.209085333	2.345471612			
214812_s_at	MOBKL1B	-0.998477667	3.121720166			
226790_at	MORN2	0.653373667	4.522982336	MORN2		
217942_at	MRPS35	0.568166667	1.79815454			
201761_at	MTHFD2	-1.131021667	2.485389855			
203433_at	MTHFS	0.531792333	3.267378371			
216095_x_at	MTMR1	-0.567174	2.345471612	MTMR1		
210360_s_at	MTSS1	-1.153246667	0			
203036_s_at	MTSS1	-1.005807	1.336284421			
210359_at	MTSS1	-0.963849667	0			
202364_at	MXI1	-0.726356333	1.79815454			
227342_s_at	MYEOV	-0.914498667	3.121720166			
223130_s_at	MYLIP	-1.184893	0.587554882			

Probeset ID	Gene Symbol	miR7-miRNC	q-value(%)	miR-7 tgts_M	miR-7 tgts_TS	miR-7 tgts_PT
212365_at	MYO1B	-0.52789	3.121720166			
212364_at	MYO1B	-0.679782667	3.121720166			
222018_at	NACA /// NACAP1	-0.767312667	2.485389855			
229757_at	NAF1	-0.778863333	3.121720166	NAF1		
202944_at	NAGA	-0.536860333	3.671565177			
1556121_at	NAP1L1	-0.577026333	3.671565177			
225448_at	NAPG	-1.056599333	0	NAPG		
219217_at	NARS2	0.632544333	3.075356134			
224771_at	NAV1	0.766265333	3.075356134	NAV1		
225847_at	NCEH1	-0.900574	4.235240967			NCEH1
211063_s_at	NCK1	-0.580914667	3.267378371	NCK1		
203245_s_at	NCRNA00094	0.847156333	4.522982336			
220399_at	NCRNA00115	-0.713692333	3.267378371			
227249_at	NDE1	-0.628478333	1.336284421			
200632_s_at	NDRG1	-1.709222667	3.121720166			
209159_s_at	NDRG4	1.357064333	0			
217773_s_at	NDUFA4	-1.048018333	0.884241011	NDUFA4	NDUFA4	
240391_at	NDUFB2	-0.695622667	3.671565177			
243357_at	NEGR1	1.012096667	3.267378371			
1553194_at	NEGR1	1.428915667	2.345471612			
1555082_a_at	NEK11	1.192521667	1.233540637			
226806_s_at	NFIA	-1.207506	3.267378371			
224976_at	NFIA	-0.881994	0.587554882			
223217_s_at	NFKBIZ	-0.640763	3.121720166			
227040_at	NHLRC3	-0.755338667	2.652723034			
202007_at	NID1	-0.71996	2.652723034	NID1		
234299_s_at	NIN	-0.552763667	2.652723034	NIN		
205851_at	NME6	-0.759152667	1.79815454			

**Probeset ID**	**Gene Symbol**	**miR7-miRNC**	**q-value(%)**	**miR-7 tgts_M**	**miR-7 tgts_TS**	**miR-7 tgts_PT**
209755_at	NMNAT2	-1.098375667	0	NMNAT2		
202443_x_at	NOTCH2	-0.711596667	4.522982336	NOTCH2		
212377_s_at	NOTCH2	-0.672263333	3.267378371	NOTCH2		
213462_at	NPAS2	-0.853028	3.121720166			
201468_s_at	NQO1	-1.087051667	2.652723034	NQO1		
212298_at	NRP1	-0.53694	3.121720166	NRP1		
1555468_at	NRP2	-0.857637333	0.587554882			
227486_at	NT5E	-0.52182	4.522982336			
224581_s_at	NUCKS1	-0.687386667	3.121720166	NUCKS1		
201270_x_at	NUDCD3	-1.075437667	4.235240967	NUDCD3		
224938_at	NUFIP2	-0.937140667	3.267378371			
209230_s_at	NUPR1	-2.358642667	0			
209628_at	NXT2	-1.030856	0.884241011	NXT2	NXT2	NXT2
220594_at	OGT	-1.670188	0	OGT	OGT	OGT
209240_at	OGT	-1.176701333	0	OGT	OGT	OGT
207563_s_at	OGT	-1.083406667	0.587554882	OGT	OGT	OGT
212307_s_at	OGT	-1.08238	1.259835547	OGT	OGT	OGT
207564_x_at	OGT	-0.88482	3.121720166	OGT	OGT	OGT
229787_s_at	OGT	-0.730873667	3.121720166	OGT	OGT	OGT
214306_at	OPA1	-0.575459333	3.267378371			
208158_s_at	OSBPL1A	-0.891697333	3.121720166			
226507_at	PAK1	-1.119439333	0.587554882	PAK1		
209615_s_at	PAK1	-0.928915	1.164891419	PAK1		
214607_at	PAK3	1.307822333	0.488023728			
201981_at	PAPPA	-1.232375	0		PAPPA	PAPPA
224941_at	PAPPA	-1.065846333	3.121720166		PAPPA	PAPPA
228237_at	PAPPA2	-1.675025	4.235240967			
203060_s_at	PAPSS2	-0.640308	1.164891419			

Probeset ID	Gene Symbol	miR7-miRNC	q-value(%)	miR-7 tgts_M	miR-7 tgts_TS	miR-7 tgts_PT
219033_at	PARP8	-0.525855667	3.121720166			
235795_at	PAX6	-0.648552	4.522982336	PAX6	PAX6	
214528_s_at	PAX8	-0.565310667	1.79815454	PAX8		
224152_s_at	PBRM1	0.790726667	4.522982336			
223899_at	PBRM1	0.955391333	0.967238362			
223900_s_at	PBRM1	1.002770667	1.79815454			
212151_at	PBX1	1.134571667	0.884241011	PBX1		
202847_at	PCK2	-1.310621	0			
224399_at	PDCD1LG2	-0.930666	3.121720166			
204134_at	PDE2A	-1.22719	4.522982336			
204491_at	PDE4D	-0.903938	4.522982336	PDE4D	PDE4D	PDE4D
210836_x_at	PDE4D	-0.838599333	1.202805955	PDE4D	PDE4D	PDE4D
210837_s_at	PDE4D	-0.675686333	4.522982336	PDE4D	PDE4D	PDE4D
211840_s_at	PDE4D	-0.515351	4.235240967	PDE4D	PDE4D	PDE4D
210305_at	PDE4DIP	0.973508333	2.652723034			
1553175_s_at	PDE5A	-0.853903	3.267378371			
221994_at	PDLIM5	-0.558823	4.235240967	PDLIM5		
208233_at	PDPN	-0.569332333	1.259835547			
1555131_a_at	PER3	0.623034667	1.233540637			
226823_at	PHACTR4	-0.588172333	2.652723034	PHACTR4	PHACTR4	
238131_at	PHC2	-0.950204333	3.267378371			
1554472_a_at	PHF20L1	-0.628621333	2.485389855	PHF20L1		
201397_at	PHGDH	-0.909055667	1.164891419			
217996_at	PHLDA1	-0.831013333	4.522982336			
215285_s_at	PHTF1	0.824159333	4.235240967			
210191_s_at	PHTF1	0.94547	0			
212511_at	PICALM	-0.863357667	2.652723034	PICALM		
213070_at	PIK3C2A	-0.788261333	4.235240967			

**Probeset ID**	**Gene Symbol**	**miR7-miRNC**	**q-value(%)**	**miR-7 tgts_M**	**miR-7 tgts_TS**	**miR-7 tgts_PT**
212688_at	PIK3CB	-0.875460333	2.485389855			
217620_s_at	PIK3CB	-0.765261333	3.121720166			
203879_at	PIK3CD	-1.244866667	0	PIK3CD	PIK3CD	
206369_s_at	PIK3CG	-0.690495667	3.671565177			
226864_at	PKIA	0.932049333	0.884241011			
235110_at	PLA2G16	1.19783	0.967238362	PLA2G16		
216971_s_at	PLEC1	-1.295646	3.121720166			PLEC1
200827_at	PLOD1	-1.450998	3.671565177	PLOD1		
202620_s_at	PLOD2	-0.78531	3.671565177			
213030_s_at	PLXNA2	-1.456791333	0.587554882			
227032_at	PLXNA2	-0.717524333	2.652723034			
207290_at	PLXNA2	-0.689534667	3.121720166			
222449_at	PMEPA1	0.777452333	3.075356134			
212037_at	PNN	-0.968072667	1.259835547			
1567213_at	PNN	-0.69586	4.235240967			
1567214_a_at	PNN	-0.626216667	3.121720166			
233030_at	PNPLA3	-0.722142667	1.336284421			
204835_at	POLA1	0.607854667	2.345471612			
208828_at	POLE3	-1.165725	1.79815454	POLE3		
225844_at	POLE4	-1.224073333	0	POLE4	POLE4	POLE4
1553587_a_at	POLE4	-1.04295	0.884241011	POLE4	POLE4	POLE4
231041_at	POLR1E	-0.630361	3.121720166			
222402_at	POMP	-0.744544667	4.235240967			
212226_s_at	PPAP2B	-1.558204667	2.652723034			
212230_at	PPAP2B	-1.658872333	0			
227025_at	PPHLN1	-0.838864667	2.345471612			
227728_at	PPM1A	-0.552715	4.235240967			
1552670_a_at	PPP1R3B	0.921723667	0.884241011			

**Probeset ID**	**Gene Symbol**	**miR7-miRNC**	**q-value(%)**	**miR-7 tgts_M**	**miR-7 tgts_TS**	**miR-7 tgts_PT**
202886_s_at	PPP2R1B	-0.509599333	3.267378371	PPP2R1B		
225066_at	PPP2R2D	-1.361326	0.587554882			
201877_s_at	PPP2R5C	-0.705693333	3.671565177			
225426_at	PPP6C	0.759816333	0.488023728			
212527_at	PPPDE2	-0.706798	1.79815454			
230708_at	PRICKLE1	-1.076491	0.587554882			
226065_at	PRICKLE1	-0.801137	2.485389855			
225984_at	PRKAA1	-0.626598333	3.121720166			
240349_at	PRKAA2	0.572097667	2.485389855			
218236_s_at	PRKD3	-0.64267	2.652723034	PRKD3		
209323_at	PRKRIR	-0.724386	4.522982336			
210476_s_at	PRLR	-1.336604	0			
211917_s_at	PRLR	-1.330297667	1.79815454			
1554867_a_at	PRR16	0.915686333	0.884241011			
205618_at	PRRG1	-1.043651333	2.652723034	PRRG1		
229073_at	PRTG	-0.840633	4.235240967			
220892_s_at	PSAT1	-1.427263333	3.267378371			
223062_s_at	PSAT1	-1.25579	0.587554882			
208257_x_at	PSG1	0.767003	3.267378371			
200988_s_at	PSME3	-1.050206	0	PSME3	PSME3	PSME3
226110_at	PTAR1	-0.970493333	0.884241011		PTAR1	
235484_at	PTAR1	-0.800085667	1.259835547		PTAR1	
203555_at	PTPN18	0.539818333	3.267378371			
205712_at	PTPRD	-0.670422667	4.235240967	PTPRD		
206157_at	PTX3	-2.092085667	0			
201607_at	PWP1	-0.603019667	1.259835547			
225796_at	PXK	-1.049763333	0			
215520_at	PYGO1	-0.831292667	1.79815454			

**Probeset ID**	**Gene Symbol**	**miR7-miRNC**	**q-value(%)**	**miR-7 tgts_M**	**miR-7 tgts_TS**	**miR-7 tgts_PT**
214541_s_at	QKI	-1.009615	1.164891419			
212636_at	QKI	-0.785281	3.121720166			
212265_at	QKI	-0.680450333	4.235240967			
219178_at	QTRTD1	-0.873926667	0	QTRTD1	QTRTD1	
203831_at	R3HDM2	-0.847478	3.267378371	R3HDM2		
34478_at	RAB11B	0.535157667	4.522982336			
210879_s_at	RAB11FIP5	-1.132019333	3.267378371	RAB11FIP5	RAB11FIP5	
209514_s_at	RAB27A	0.587926	2.652723034			
210951_x_at	RAB27A	0.595969333	3.075356134			
227123_at	RAB3B	0.734701	4.522982336			
228432_at	RAB3IP	0.603784	4.235240967			
213704_at	RABGGTB	-1.683975333	0			
209181_s_at	RABGGTB	-0.92422	1.164891419			
209180_at	RABGGTB	-0.73812	3.121720166			
204478_s_at	RABIF	0.607254	2.345471612			
210255_at	RAD51L1	0.715874	2.652723034			
201244_s_at	RAF1	-0.749153333	1.202805955	RAF1	RAF1	RAF1
221809_at	RANBP10	0.749035333	1.336284421	RANBP10		
53987_at	RANBP10	0.867540333	2.345471612	RANBP10		
208530_s_at	RARB	0.729352	2.485389855			
210550_s_at	RASGRF1	1.056664667	0			
236613_at	RBM25	-0.948649333	1.164891419			
226272_at	RCAN3	0.641638667	1.79815454			
230202_at	RELA	-0.884386333	4.235240967	RELA		
201783_s_at	RELA	-1.084247667	0.884241011	RELA		
1552651_a_at	RFFL	-0.864844333	4.235240967	RFFL		
228980_at	RFFL	-0.758182667	0	RFFL		
203823_at	RGS3	-0.966549	2.652723034			

Probeset ID	Gene Symbol	miR7-miRNC	q-value(%)	miR-7 tgts_M	miR-7 tgts_TS	miR-7 tgts_PT
1554897_s_at	RHBDL2	-0.890074667	3.121720166			
35685_at	RING1	0.577007	2.485389855			
208371_s_at	RING1	0.692315	2.485389855			
211678_s_at	RNF114	-1.054624667	1.164891419	RNF114		RNF114
200867_at	RNF114	-0.963122	0	RNF114		RNF114
225414_at	RNF149	-0.831801333	3.267378371			
218528_s_at	RNF38	-0.731113	3.671565177	RNF38		
209111_at	RNF5	-0.983915667	4.522982336			
227268_at	RNFT1	-0.944572667	1.259835547			
226877_at	RPL32P3	-0.834382667	4.235240967			
213459_at	RPL37A	-0.727465	3.671565177			
214097_at	RPS21	-0.715312333	1.259835547			
227722_at	RPS23	0.953195667	2.485389855			
1555878_at	RPS24	-0.808988333	3.121720166	RPS24		
201890_at	RRM2	1.162881	0			
225773_at	RSPRY1	-1.128661667	0.587554882			
225774_at	RSPRY1	-0.801426333	3.121720166			
201980_s_at	RSU1	-0.855856667	1.164891419	RSU1		
238550_at	RUFY2	-0.959559333	1.336284421			
232231_at	RUNX2	-1.122809	3.121720166			
218377_s_at	RWDD2B	0.552812333	3.267378371			
202853_s_at	RYK	-1.072854	1.259835547	RYK		
214172_x_at	RYK	-1.063804667	3.121720166	RYK		
228923_at	S100A6	-1.461995667	3.671565177			
214456_x_at	SAA1 /// SAA2	-2.610745667	1.202805955			
208607_s_at	SAA1 /// SAA2	-2.395396333	2.345471612			
202797_at	SACM1L	-0.876779	2.485389855	SACM1L		
234491_s_at	SAV1	-0.890352	1.259835547	SAV1		

**Probeset ID**	**Gene Symbol**	**miR7-miRNC**	**q-value(%)**	**miR-7 tgts_M**	**miR-7 tgts_TS**	**miR-7 tgts_PT**
222573_s_at	SAV1	-0.619433333	3.267378371	SAV1		
224983_at	SCARB2	-1.611795	2.652723034	SCARB2		
201646_at	SCARB2	-1.117837667	0	SCARB2		
201647_s_at	SCARB2	-1.013853333	0.587554882	SCARB2		
228930_at	SCARNA15	-1.030062	1.164891419			
212900_at	SEC24A	-0.960971	3.121720166	SEC24A		
230215_at	SEC61A2	-0.504135667	3.267378371			
230265_at	SEL1L	-0.666486667	3.671565177	SEL1L		
229427_at	SEMA5A	-1.243511333	2.485389855			
212698_s_at	SEPT10	-0.742979667	4.522982336	SEPT10		
212190_at	SERPINE2	-1.344465333	0			
227487_s_at	SERPINE2	-0.952465667	1.259835547			
207714_s_at	SERPINH1	-1.461419	0	SERPINH1		
202657_s_at	SERTAD2	-0.799201	1.79815454			
202656_s_at	SERTAD2	-0.660977333	4.522982336			
235684_s_at	SESN3	1.015411667	1.336284421			
235683_at	SESN3	1.016083333	1.336284421			
220200_s_at	SETD8	-1.073348667	3.121720166	SETD8	SETD8	SETD8
210172_at	SF1	-0.555828	3.121720166			
213370_s_at	SFMBT1	0.738469667	2.652723034			
202037_s_at	SFRP1	-0.597836333	1.202805955			
212322_at	SGPL1	-1.100033333	1.259835547			
212321_at	SGPL1	-0.979887667	1.259835547			
208381_s_at	SGPL1	-0.903334667	0.587554882			
203320_at	SH2B3	-0.698961667	3.121720166			
209371_s_at	SH3BP2	-0.881833667	2.485389855			
217257_at	SH3BP2	-0.690330333	3.121720166			
225162_at	SH3D19	-0.722483667	3.121720166			

**Probeset ID**	**Gene Symbol**	**miR7-miRNC**	**q-value(%)**	**miR-7 tgts_M**	**miR-7 tgts_TS**	**miR-7 tgts_PT**
220563_s_at	SHANK1	-1.360337	1.164891419			
213308_at	SHANK2	-1.913073333	0	SHANK2	SHANK2	SHANK2
214095_at	SHMT2	-0.629882667	3.267378371			
214096_s_at	SHMT2	-0.613566667	4.522982336			
210135_s_at	SHOX2	-0.721773333	0.884241011			
215856_at	SIGLEC15	0.800166667	2.345471612			
206934_at	SIRPB1	-1.561482333	0	SIRPB1		
229796_at	SIX4	-0.973481333	1.79815454			
200711_s_at	SKP1	-0.957986667	1.259835547	SKP1	SKP1	SKP1
224844_at	SLAIN2	-0.751258333	1.259835547	SLAIN2		
203124_s_at	SLC11A2	-0.941557333	1.164891419			
209681_at	SLC19A2	-0.682479333	2.485389855			
1569054_at	SLC1A3	-1.199367333	0			
209610_s_at	SLC1A4	-1.000477667	1.259835547			
212811_x_at	SLC1A4	-0.916372333	1.336284421			
212810_s_at	SLC1A4	-1.918650667	0			
201920_at	SLC20A1	-1.074550667	1.164891419			
230494_at	SLC20A1	-0.985977	1.164891419			
218653_at	SLC25A15	-1.189347667	1.259835547	SLC25A15	SLC25A15	
222705_s_at	SLC25A15	-0.809075333	2.652723034	SLC25A15	SLC25A15	
226217_at	SLC30A7	0.937776333	3.267378371			
218519_at	SLC35A5	-0.697916	4.522982336	SLC35A5		
209711_at	SLC35D1	-0.770836333	3.267378371			
240335_at	SLC35F5	-1.441292	0			
222982_x_at	SLC38A2	-0.710633333	3.121720166	SLC38A2	SLC38A2	SLC38A2
227046_at	SLC39A11	-1.651654667	0	SLC39A11		
224596_at	SLC44A1	-0.877535333	4.522982336			
224595_at	SLC44A1	-0.869039333	2.652723034			

**Probeset ID**	**Gene Symbol**	**miR7-miRNC**	**q-value(%)**	**miR-7 tgts_M**	**miR-7 tgts_TS**	**miR-7 tgts_PT**
227620_at	SLC44A1	-0.753985333	3.121720166			
1552779_a_at	SLC44A5	1.104580667	1.233540637			
234976_x_at	SLC4A5	-1.197857667	2.485389855			
209884_s_at	SLC4A7	-0.865813	1.336284421	SLC4A7		SLC4A7
1554113_a_at	SLC4A8	1.238314333	1.79815454			
212944_at	SLC5A3	-0.852218667	1.336284421		SLC5A3	
240419_at	SLC6A15	-0.632446	4.235240967	SLC6A15		
217678_at	SLC7A11	-0.918763333	2.652723034			
207626_s_at	SLC7A2	-0.961542667	4.522982336	SLC7A2		
225516_at	SLC7A2	-1.160175	1.202805955	SLC7A2		
209679_s_at	SMAGP	-1.26137	3.121720166	SMAGP		
201589_at	SMC1A	0.622566	4.522982336	SMC1A	SMC1A	
204240_s_at	SMC2	0.813537667	1.336284421	SMC2		
212569_at	SMCHD1	-0.730611333	2.485389855			
212579_at	SMCHD1	-0.689383667	2.485389855			
224842_at	SMG1	-0.639944	4.522982336	SMG1	SMG1	
222597_at	SNAP29	-0.920544333	1.336284421			
237833_s_at	SNCAIP	0.719812667	0.967238362			
224610_at	SNHG1	-0.855842667	4.522982336			
238691_at	SNHG10	-0.872975	4.235240967			
215011_at	SNHG3	-1.181054333	3.267378371			
1564911_at	SNHG4	-1.700191667	4.235240967			
225220_at	SNHG8	-1.486193333	4.522982336			
215224_at	SNORA21	-0.948382	2.485389855			
227655_at	SNORD123	-0.562317333	1.336284421			
242146_at	SNRPA1	-0.854699667	3.121720166			
231723_at	SNX12	-0.765689	4.522982336			
227031_at	SNX13	-0.525005333	3.267378371			

Probeset ID	Gene Symbol	miR7-miRNC	q-value(%)	miR-7 tgts_M	miR-7 tgts_TS	miR-7 tgts_PT
226178_at	SOCS4	-0.751036333	1.336284421			
226572_at	SOCS7	-0.792216	3.267378371	SOCS7		
221477_s_at	SOD2	-1.007411	1.336284421			
222513_s_at	SORBS1	-0.869642333	2.485389855	SORBS1		
212870_at	SOS2	-0.672741333	2.485389855			
223980_s_at	SP110	-0.779145333	4.235240967			
222154_s_at	SPATS2L	0.80286	3.267378371	SPATS2L		
1555882_at	SPIN3	-0.618602333	2.652723034			
224995_at	SPIRE1	-1.154054	4.522982336	SPIRE1		
209436_at	SPON1	-0.856228667	4.235240967			
209875_s_at	SPP1	1.132586	2.652723034			
1568574_x_at	SPP1	1.345295667	0.967238362			
226837_at	SPRED1	-0.651784333	4.522982336			
204011_at	SPRY2	-0.815825	1.79815454			
208920_at	SRI	-0.904760333	2.652723034			
229744_at	SSFA2	-0.813521333	2.652723034			
225033_at	ST3GAL1	-0.661016667	2.345471612			
214971_s_at	ST6GAL1	-1.215393333	0.587554882			
201998_at	ST6GAL1	-1.131418	4.235240967			
214970_s_at	ST6GAL1	-1.091385333	3.121720166			
1569788_at	ST8SIA1	-1.829504	0.884241011			
215505_s_at	STRN3	-0.510956333	3.121720166	STRN3	STRN3	STRN3
224700_at	STT3B	-0.806815333	3.267378371			
221727_at	SUB1	-0.928042	1.336284421			
233827_s_at	SUPT16H	0.601263333	3.671565177			
232387_at	SYNRG	0.608817667	2.345471612			
227891_s_at	TAF15	-0.954017333	3.671565177	TAF15		
234168_at	TAF15	-0.758936667	1.79815454	TAF15		

Probeset ID	Gene Symbol	miR7-miRNC	q-value(%)	miR-7 tgts_M	miR-7 tgts_TS	miR-7 tgts_PT
235020_at	TAF4B	-0.548757667	4.522982336	TAF4B		
225973_at	TAP2	-0.700565667	1.259835547			
227407_at	TAPT1	-0.857053333	4.522982336			
240206_at	TARS	-1.103299333	4.235240967			
211052_s_at	TBCD	-0.815536	4.235240967	TBCD		
233633_at	TBL1XR1	-0.842313667	3.267378371			
229706_at	TCERG1	-0.708525333	4.522982336	TCERG1		TCERG1
223274_at	TCF19	0.708475333	2.345471612			
212386_at	TCF4	-0.594426333	3.267378371	TCF4	TCF4	
212761_at	TCF7L2	-0.678958667	4.522982336			
232258_at	TCTE3	-1.536918333	3.121720166			
1557945_at	TCTE3	-1.719009	0			
242939_at	TFDP1	-0.541832333	4.522982336	TFDP1		
213258_at	TFPI	-1.07711	3.267378371	TFPI		
209676_at	TFPI	-1.040796667	3.671565177	TFPI		
208691_at	TFRC	-1.004519667	2.652723034	TFRC	TFRC	TFRC
207332_s_at	TFRC	-0.745543333	3.267378371	TFRC	TFRC	TFRC
211258_s_at	TGFA	-1.06985	0	TGFA		
205016_at	TGFA	-1.210024333	1.259835547	TGFA		
220407_s_at	TGFB2	1.448298	0	TGFB2		
208944_at	TGFBR2	-0.54384	2.485389855			
218724_s_at	TGIF2	0.577946667	2.652723034			
201042_at	TGM2	-0.633246667	4.522982336			
203888_at	THBD	-1.395412333	1.164891419			
201109_s_at	THBS1	0.632441333	4.235240967			THBS1
226117_at	TIFA	-0.918779	0.587554882			
1553099_at	TIGD1	-0.731421667	3.267378371			
217612_at	TIMM50	-1.267004667	4.522982336	TIMM50		

Probeset ID	Gene Symbol	miR7-miRNC	q-value(%)	miR-7 tgts_M	miR-7 tgts_TS	miR-7 tgts_PT
201149_s_at	TIMP3	-2.195775667	0			
201150_s_at	TIMP3	-2.729882667	0			
201147_s_at	TIMP3	-1.849861667	0			
212665_at	TIPARP	-0.60784	4.235240967			
203220_s_at	TLE1	0.634178667	3.075356134			
203222_s_at	TLE1	0.750021667	2.652723034			
204872_at	TLE4	-0.619784667	4.522982336			
215033_at	TM4SF1	-1.542192667	3.121720166	TM4SF1		
224755_at	TM9SF3	-0.719110333	2.485389855	TM9SF3		
212352_s_at	TMED10	-0.73499	3.121720166	TMED10		
200929_at	TMED10	-0.514016667	4.235240967	TMED10		
239627_at	TMED9	-1.077871333	4.235240967	TMED9	TMED9	TMED9
220169_at	TMEM156	-0.901984	3.671565177			
241844_x_at	TMEM156	-0.864624333	1.336284421			
213338_at	TMEM158	-1.41653	0			
212164_at	TMEM183A	-0.942255333	3.121720166			
212623_at	TMEM41B	-0.702701	4.235240967			
219410_at	TMEM45A	-1.440164333	0			
234672_s_at	TMEM48	-0.936111667	1.202805955			
231697_s_at	TMEM49	-0.974991667	0			
227936_at	TMEM68	-0.733142	2.485389855	TMEM68		
207196_s_at	TNIP1	-0.600812667	4.235240967			
225765_at	TNPO1	-0.519644667	3.121720166			
235602_at	TP53INP1	0.626663667	3.671565177			
202479_s_at	TRIB2	-0.628485667	1.259835547	TRIB2		
202478_at	TRIB2	-1.429087667	0.587554882	TRIB2		
203148_s_at	TRIM14	-0.906431333	1.79815454			
210995_s_at	TRIM23	0.865646333	0.488023728	TRIM23		

Probeset ID	Gene Symbol	miR7-miRNC	q-value(%)	miR-7 tgts_M	miR-7 tgts_TS	miR-7 tgts_PT
210994_x_at	TRIM23	0.954428	0.967238362	TRIM23		
224806_at	TRIM25	-0.984163333	3.267378371			
221897_at	TRIM52	-0.694048333	2.485389855	TRIM52		
223132_s_at	TRIM8	-1.142103667	2.345471612			
223131_s_at	TRIM8	-1.016276	3.267378371			
221012_s_at	TRIM8	-0.985976667	0			
221229_s_at	TRMT61B	-0.721347333	3.267378371			
213027_at	TROVE2	-0.796343	0			
212839_s_at	TROVE2	-0.63592	4.235240967			
209109_s_at	TSPAN6	0.649963333	3.267378371			
221493_at	TSPYL1	-0.700732667	4.522982336	TSPYL1		
225180_at	TTC14	-0.759574	0.587554882			
213174_at	TTC9	0.5964	1.336284421			
230891_at	TUBE1	-1.557673667	0	TUBE1		
226181_at	TUBE1	-1.349856667	0	TUBE1		
201010_s_at	TXNIP	1.039912333	3.671565177			
221490_at	UBAP1	-0.831219333	1.202805955			
46270_at	UBAP1	-0.669415	1.164891419			
211764_s_at	UBE2D1	-0.601746667	3.267378371			
217823_s_at	UBE2J1	-0.560476667	4.522982336	UBE2J1		
222656_at	UBE2W	-0.774379667	1.202805955			
227413_at	UBLCP1	-1.130022333	3.121720166	UBLCP1	UBLCP1	UBLCP1
212934_at	UBXN2B	-0.829024333	3.671565177	UBXN2B		
233245_at	UHRF1BP1	-1.043952667	2.485389855	UHRF1BP1		
236272_at	UNC119B	0.857203	2.652723034			
229672_at	UQCC	-0.854922333	0.884241011			
241755_at	UQCRC2	-0.590119333	3.671565177			
209475_at	USP15	-0.565263667	1.164891419			

Probeset ID	Gene Symbol	miR7-miRNC	q-value(%)	miR-7 tgts_M	miR-7 tgts_TS	miR-7 tgts_PT
203869_at	USP46	-0.576425	4.522982336			
219675_s_at	UXS1	0.543583333	2.652723034			
204254_s_at	VDR	0.971318	2.345471612			
211527_x_at	VEGFA	-0.811529	3.267378371			
209946_at	VEGFC	-1.068400667	1.259835547			
212399_s_at	VGLL4	-0.720984333	2.485389855	VGLL4		
1555938_x_at	VIM	-1.249948667	1.259835547			
209822_s_at	VLDLR	-1.197247	0.587554882			
200628_s_at	WARS	-0.873239	4.235240967			
200629_at	WARS	-0.768625333	1.79815454			
204042_at	WASF3	-1.427599333	0	WASF3	WASF3	
213836_s_at	WIPI1	-0.650241667	3.267378371			
226182_s_at	WISP3	-0.759793333	1.259835547			
212637_s_at	WWP1	-0.746545333	4.235240967		WWP1	WWP1
212638_s_at	WWP1	-0.610333333	3.267378371		WWP1	WWP1
228363_at	XIAP	-0.937778667	1.336284421		XIAP	
235222_x_at	XIAP	-0.797038667	4.522982336		XIAP	
212048_s_at	YARS	-1.029342333	1.259835547			
224953_at	YIPF5	-0.674397	3.267378371	YIPF5		
215150_at	YOD1	-0.689149667	1.164891419	YOD1		
218647_s_at	YRDC	-0.704336333	2.652723034			
222430_s_at	YTHDF2	0.542036	2.485389855			
204799_at	ZBED4	-0.687751667	0.587554882			
225629_s_at	ZBTB4	-0.763684333	4.522982336	ZBTB4		ZBTB4
231899_at	ZC3H12C	-0.920977333	0			
212402_at	ZC3H13	-0.535861	4.235240967	ZC3H13		
222451_s_at	ZDHHC9	-1.396425667	0.587554882	ZDHHC9		
228317_at	ZFAND5	-0.825508333	3.121720166			

**Probeset ID**	**Gene Symbol**	**miR7-miRNC**	**q-value(%)**	**miR-7 tgts_M**	**miR-7 tgts_TS**	**miR-7 tgts_PT**
213065_at	ZFC3H1	-0.924743	4.522982336	ZFC3H1		
1559270_at	ZFHX4	0.522322	3.267378371	ZFHX4		
201368_at	ZFP36L2	-0.638609333	4.235240967			
226581_at	ZFYVE20	-0.653507	1.164891419	ZFYVE20		
210282_at	ZMYM2	-1.003531667	3.267378371	ZMYM2		
228157_at	ZNF207	-0.942706	3.121720166	ZNF207		
231848_x_at	ZNF207	-0.904013333	1.164891419	ZNF207		
1556035_s_at	ZNF207	-0.877426333	2.345471612	ZNF207		
219826_at	ZNF419	-0.830149333	3.267378371			
205964_at	ZNF426	-0.674046333	1.336284421			
219711_at	ZNF586	-0.710133	3.267378371			
241499_at	ZNF621	-0.679997333	3.671565177			
227122_at	ZNF791	-0.595340333	3.121720166			

Supplementary Table 2. Differentially expressed genes in miR-128 transfected HEY cells. Differentially expressed genes (fold change ≥1.4, FDR ≤5%) in miR-128 transfected cells, compared to miR-NC transfected cells. 'Probeset ID' refers to Affymetrix HG-U133 Plus 2.0 probeset identifier. 'Gene Symbol' shows the official gene symbol for the corresponding Probeset ID. 'miR128-miR-NC' refers to the difference between average log_2 signal values of the miR-128 transfected group and the miR-NC transfected group. 'q-value (%)' shows the false discovery rate calculated using the SAM algorithm. miRanda predicted targets of miR-128 are listed in the column titled 'miR-128tgts_M', TargetScan predicted targets of miR-128 are listed under the heading miR-128tgts_TS, and PicTar predicted targets of miR-128 are listed under the heading miR-128tgts_PT.

Probe Set ID	Gene Symbol	miR-128-miRNC	q value (%)	miR-128 tgts_M	miR-128 tgts_TS	miR-128 tgts_PT
203504_s_at	ABCA1	-1.2890325	0	ABCA1	ABCA1	ABCA1
203505_at	ABCA1	-1.448058167	0	ABCA1	ABCA1	ABCA1
216066_at	ABCA1	-1.554447667	3.701646186	ABCA1	ABCA1	ABCA1
223320_s_at	ABCB10	-1.301568833	0.393887585	ABCB10		
208561_at	ABCC9	1.917778	0			
208562_s_at	ABCC9	1.1849265	3.029425085			
202850_at	ABCD3	0.540919167	3.701646186	ABCD3		
213935_at	ABHD5	0.5595905	3.701646186			
221679_s_at	ABHD6	0.820912167	0.796301331			
205730_s_at	ABLIM3	0.510903333	0.550502435			
213497_at	ABTB2	0.636957333	0.230227638			
205412_at	ACAT1	-0.527562	3.701646186			
202323_s_at	ACBD3	0.555013333	1.123589586			
202324_s_at	ACBD3	0.6097835	0.550502435			
222688_at	ACER3	0.579175	1.123589586			
231321_s_at	ACER3	0.53604	0.137080273			
205942_s_at	ACSM3	1.900536833	3.701646186			
219616_at	ACSS3	1.1664155	0			
229222_at	ACSS3	0.7010045	3.701646186			
200974_at	ACTA2	0.57388	1.123589586		ACTA2	
211995_x_at	ACTG1	-0.520146667	2.499099453			
212988_x_at	ACTG1	-0.519393333	3.029425085			
205209_at	ACVR1B	0.717875333	3.029425085			
208218_s_at	ACVR1B	0.814223833	0			
208223_s_at	ACVR1B	0.746774833	3.701646186			
213198_at	ACVR1B	0.981891	0			
1559548_at	ACVR2B	2.1022055	0			
220028_at	ACVR2B	1.981403667	0			

**Probe Set ID**	**Gene Symbol**	**miR-128-miRNC**	**q value (%)**	**miR-128 tgts_M**	**miR-128 tgts_TS**	**miR-128 tgts_PT**
203504_s_at	ABCA1	-1.2890325	0	ABCA1	ABCA1	ABCA1
236126_at	ACVR2B	2.224874	0			
205260_s_at	ACYP1	0.625475333	1.455154512			
216705_s_at	ADA	1.012514833	0.230227638			
244463_at	ADAM23	0.594664167	3.701646186			
220866_at	ADAMTS6	-0.664578667	0	ADAMTS6		
1554697_at	ADAMTS9	0.718192333	3.029425085	ADAMTS9		
226426_at	ADNP	-0.5487835	1.455154512	ADNP		
205891_at	ADORA2B	-0.877400167	0	ADORA2B	ADORA2B	ADORA2B
206170_at	ADRB2	-1.272666833	0	ADRB2		
204184_s_at	ADRBK2	0.509439	0.796301331			
227310_at	ADSS	0.691361667	0.796301331			
201792_at	AEBP1	0.817765	0.137080273			
1565034_s_at	AFF3 /// MLL	0.6259095	0.796301331			
217939_s_at	AFTPH	0.910508333	0			
222472_at	AFTPH	0.767316333	3.701646186			
218096_at	AGPAT5	0.612708667	3.701646186			
232007_at	AGPAT5	1.3494965	0			
204500_s_at	AGTPBP1	0.513328333	3.029425085			
226115_at	AHCTF1	-0.654191667	0.393887585			
215672_s_at	AHCYL2	0.682438833	2.499099453	AHCYL2		
211986_at	AHNAK	-0.626413333	0.550502435			
212992_at	AHNAK2	-0.7592465	1.123589586			
205996_s_at	AK2	-1.22597	0	AK2	AK2	AK2
212173_at	AK2	-0.58158	2.499099453	AK2	AK2	AK2
212174_at	AK2	-1.338656	0	AK2	AK2	AK2
212175_s_at	AK2	-1.071963333	0	AK2	AK2	AK2
204347_at	AK3L1	0.985481667	0.230227638			
1553502_a_at	AKAP2 /// PALM2 /// PALM2-AKAP2	1.255804167	0			
1564002_a_at	AKD1	0.571346333	1.810509752			
225701_at	AKNA	0.607305	1.810509752			
201272_at	AKR1B1	-0.803764833	1.123589586			
202139_at	AKR7A2	-0.605714667	3.701646186	AKR7A2		
212607_at	AKT3	0.596703333	1.455154512	AKT3		
212609_s_at	AKT3	0.668008	1.123589586	AKT3		
219393_s_at	AKT3	0.532755	1.810509752	AKT3		
222880_at	AKT3	0.707581667	0.550502435	AKT3		
218373_at	AKTIP	0.7816065	0.796301331			
223894_s_at	AKTIP	0.710029333	0.05396551			
203180_at	ALDH1A3	1.718034333	0			
1559393_at	ALDH1L2	0.636991667	1.123589586	ALDH1L2		
202053_s_at	ALDH3A2	0.51599	1.810509752	ALDH3A2		
205640_at	ALDH3B1	-1.051461167	0.137080273			
219015_s_at	ALG13	0.5435515	0.393887585			
219649_at	ALG6	0.634426	1.123589586	ALG6		
203545_at	ALG8	0.662311667	1.123589586			
225625_at	ALKBH2	0.714164	3.029425085			

Probe Set ID	Gene Symbol	miR-128-miRNC	q value (%)	miR-128 tgts_M	miR-128 tgts_TS	miR-128 tgts_PT
203504_s_at	ABCA1	-1.2890325	0	ABCA1	ABCA1	ABCA1
204174_at	ALOX5AP	1.664466333	0			
209424_s_at	AMACR	0.610616333	1.810509752			
222108_at	AMIGO2	-0.744633	1.123589586			
1553219_a_at	AMMECR1	1.631660167	0			
204976_s_at	AMMECR1	1.336621667	0.05396551			
226421_at	AMMECR1	1.7951395	0			
225450_at	AMOTL1	0.516324833	0.393887585			
203002_at	AMOTL2	-0.73718	0.550502435			
207992_s_at	AMPD3	0.621892167	1.123589586			
239651_at	ANAPC5	1.174939833	0			
225521_at	ANAPC7	-0.8982025	1.455154512			
211148_s_at	ANGPT2	1.234888833	3.701646186	ANGPT2		
205391_x_at	ANK1	1.516891167	3.701646186		ANK1	ANK1
208353_x_at	ANK1	1.352047667	1.455154512		ANK1	ANK1
206385_s_at	ANK3	1.148116333	0.137080273	ANK3		
207950_s_at	ANK3	0.72788	0	ANK3		
209442_x_at	ANK3	0.648911333	1.455154512	ANK3		
223092_at	ANKH	0.74677	0			
223094_s_at	ANKH	0.520049	1.810509752			
210486_at	ANKMY1	0.740091167	0.393887585			
223251_s_at	ANKRD10	-0.700433333	0	ANKRD10		
212289_at	ANKRD12	0.593848667	3.029425085			
238642_at	ANKRD13D	0.846839333	0.05396551	ANKRD13D		
1561079_at	ANKRD28	-0.902397	0.137080273	ANKRD28		
213035_at	ANKRD28	-0.878198167	0	ANKRD28		
226025_at	ANKRD28	-0.84847	0.230227638	ANKRD28		
229307_at	ANKRD28	-1.210447	0.05396551	ANKRD28		
238332_at	ANKRD29	0.54824	3.029425085			
223542_at	ANKRD32	-0.656516667	0.796301331			
227337_at	ANKRD37	1.047029833	0.550502435			
212731_at	ANKRD46	1.246949667	0			
222951_s_at	ANKRD5	1.621154	0			
219496_at	ANKRD57	0.923220667	0.393887585			
227034_at	ANKRD57	0.519715833	3.701646186			
204672_s_at	ANKRD6	0.849068	0.137080273			
212747_at	ANKS1A	0.973421667	0.05396551	ANKS1A	ANKS1A	
218910_at	ANO10	0.694600167	3.701646186	ANO10		
202888_s_at	ANPEP	-0.6315255	1.123589586			
227660_at	ANTXR1	-0.533176667	2.499099453			
1553150_at	AOF1	1.299071333	0			
227021_at	AOF1	1.090397833	0			
225754_at	AP1G1	0.746894	1.810509752	AP1G1		
203300_x_at	AP1S2	0.565851667	0.550502435	AP1S2		
231714_s_at	AP4B1	0.564187833	1.455154512	AP4B1		
231962_at	AP4B1	0.8295375	2.499099453	AP4B1		
236221_at	AP4B1	1.221504833	0	AP4B1		
220229_s_at	AP4E1	0.5321625	3.029425085			
228164_at	AP4E1	0.664016333	1.455154512			
206679_at	APBA1	1.432571167	0			
228101_at	APBA1	1.238016	0.550502435			

Probe Set ID	Gene Symbol	miR-128-miRNC	q value (%)	miR-128 tgts_M	miR-128 tgts_TS	miR-128 tgts_PT
203504_s_at	ABCA1	-1.2890325	0	ABCA1	ABCA1	ABCA1
235548_at	APCDD1L	0.534763833	2.499099453	APCDD1L		
221087_s_at	APOL3	1.027558667	0			
1557116_at	APOL6	0.610134167	3.701646186			
219716_at	APOL6	0.747502667	1.810509752			
221031_s_at	APOLD1	-0.518254	3.029425085	APOLD1	APOLD1	
214102_at	ARAP2	-0.677203833	0.05396551			
200011_s_at	ARF3	-0.646139167	0.393887585		ARF3	
207606_s_at	ARHGAP12	-0.575668333	0.796301331	ARHGAP12	ARHGAP12	ARHGAP12
1560821_at	ARHGAP22	-0.579054333	0.550502435			
205068_s_at	ARHGAP26	1.3401365	0			
215955_x_at	ARHGAP26	1.16964	0.393887585			
226906_s_at	ARHGAP9	0.923947167	0.796301331			
215139_at	ARHGEF10	1.1584695	3.701646186	ARHGEF10		
213039_at	ARHGEF18	0.505324	2.499099453			
204765_at	ARHGEF5	1.196906833	0.05396551			
235412_at	ARHGEF7	1.077317167	0			
223111_x_at	ARID4B	0.616460333	2.499099453	ARID4B		ARID4B
238068_at	ARIH2	-0.617525167	0.230227638			
229028_s_at	ARL17	-0.648820167	1.810509752			
202092_s_at	ARL2BP	-0.546442	0	ARL2BP		
202206_at	ARL4C	-0.802072167	0.550502435			
202207_at	ARL4C	-0.666493333	0.393887585			
202208_s_at	ARL4C	-0.696847667	0.796301331			
213759_at	ARL4C	-1.044259333	0.550502435			
218150_at	ARL5A	0.694795	3.701646186			
242727_at	ARL5B	0.866140667	2.499099453			
223735_at	ARL6	0.721361667	1.455154512	ARL6		
211935_at	ARL6IP1	-0.603885	1.123589586			
217852_s_at	ARL8B	0.589111667	1.455154512	ARL8B		ARL8B
1555279_at	ARMC8	-0.803865	0	ARMC8	ARMC8	ARMC8
1555281_x_at	ARMC8	-0.822949667	0	ARMC8	ARMC8	ARMC8
219094_at	ARMC8	-1.360451	0	ARMC8	ARMC8	ARMC8
219636_s_at	ARMC9	0.514447	3.701646186			
227444_at	ARMCX4	-0.502335833	1.455154512	ARMCX4		
1563101_at	ARNTL2	0.613378333	2.499099453			
220658_s_at	ARNTL2	0.712288333	0			
223586_at	ARNTL2	1.000428	0			
224204_x_at	ARNTL2	0.839825	0			
223665_at	ARPM1	0.734451167	0.05396551			
218832_x_at	ARRB1	-0.625153833	3.701646186			
223695_s_at	ARSD	0.894769	0.796301331			
223696_at	ARSD	0.9920135	3.701646186			
239147_at	ARSK	0.819457167	0			
205808_at	ASPH	-0.622569833	1.123589586	ASPH		
209135_at	ASPH	-1.100363333	0	ASPH		
210896_s_at	ASPH	-1.195448333	0	ASPH		
225008_at	ASPH	-1.059891333	0.230227638	ASPH		
214993_at	ASPHD1	0.507827333	3.029425085			
219918_s_at	ASPM	-0.541938833	0.550502435			
232238_at	ASPM	-0.788898333	0.796301331			

Probe Set ID	Gene Symbol	miR-128-miRNC	q value (%)	miR-128 tgts_M	miR-128 tgts_TS	miR-128 tgts_PT
203504_s_at	ABCA1	-1.2890325	0	ABCA1	ABCA1	ABCA1
209693_at	ASTN2	0.520120167	3.701646186			
224850_at	ATAD1	0.522581667	3.701646186			
232908_at	ATAD2B	1.053269667	3.701646186			
1558233_s_at	ATF1	0.804484167	0.796301331			
1565269_s_at	ATF1	0.675098333	0.550502435			
202672_s_at	ATF3	0.566412667	0.230227638	ATF3		
203952_at	ATF6	0.509225	1.455154512	ATF6		
206684_s_at	ATF7	1.0602145	0			
228830_s_at	ATF7	0.931	0.05396551			
235160_at	ATF7	0.933074667	0			
244587_at	ATF7	1.034226	0.137080273			
232612_s_at	ATG16L1	0.9980395	0.550502435			
229389_at	ATG16L2	0.894641833	0.550502435	ATG16L2		
1559485_at	ATG2B	0.5678935	0.796301331			
223340_at	ATL1	-0.5054	3.701646186			
1570352_at	ATM	1.043292667	3.029425085			
201855_s_at	ATMIN	1.086113333	0.05396551			
213238_at	ATP10D	0.541074833	0.550502435			
212362_at	ATP2A2	0.501221833	2.499099453			
205410_s_at	ATP2B4	0.973074667	0.230227638			
212135_s_at	ATP2B4	0.764658833	0.393887585			
212136_at	ATP2B4	0.782377833	0.796301331			
211137_s_at	ATP2C1	-0.645667833	1.455154512			
236171_at	ATP2C2	-0.567798167	3.029425085			
214132_at	ATP5C1	1.110065333	0			
208745_at	ATP5L	-0.765475833	0.137080273			
221504_s_at	ATP6V1H	-0.509387333	3.029425085	ATP6V1H		
219659_at	ATP8A2	1.64259	0			
219660_s_at	ATP8A2	1.137993333	3.701646186			
212062_at	ATP9A	0.500751667	2.499099453		ATP9A	
203231_s_at	ATXN1	1.9079905	0			
203232_s_at	ATXN1	1.1217165	0			
208832_at	ATXN10	-1.053695167	0.137080273	ATXN10	ATXN1	ATXN10
208833_s_at	ATXN10	-0.74774	0.05396551	ATXN10	ATXN1	ATXN10
226095_s_at	ATXN1L	0.626971	0.550502435			
227373_at	ATXN1L	0.761206167	3.701646186			
205415_s_at	ATXN3	0.561828333	0.550502435			
204516_at	ATXN7	0.650414	1.810509752	ATXN7		
204092_s_at	AURKA	-0.934966667	0.137080273			
208079_s_at	AURKA	-0.736003333	0.393887585			
208080_at	AURKA	-0.852462667	1.455154512			
218580_x_at	AURKAIP1	-0.582230667	3.701646186			
225552_x_at	AURKAIP1	-0.6224585	3.701646186			
225555_x_at	AURKAIP1	-0.705009	1.810509752			
219366_at	AVEN	0.7664865	0.05396551			
227100_at	B3GALTL	-0.753492167	1.455154512			
222870_s_at	B3GNT2	0.768843	1.455154512			
1554835_a_at	B3GNT5	1.126444667	0			
225612_s_at	B3GNT5	0.635103	2.499099453			
201882_x_at	B4GALT1	-0.963641	1.123589586	B4GALT1		

Probe Set ID	Gene Symbol	miR-128-miRNC	q value (%)	miR-128 tgts_M	miR-128 tgts_TS	miR-128 tgts_PT
203504_s_at	ABCA1	-1.2890325	0	ABCA1	ABCA1	ABCA1
201883_s_at	B4GALT1	-1.288009833	0	B4GALT1		
211631_x_at	B4GALT1	-0.56589	1.123589586	B4GALT1		
216627_s_at	B4GALT1	-0.718119333	0.550502435	B4GALT1		
229403_at	B4GALT1	-0.728685	1.455154512	B4GALT1		
206232_s_at	B4GALT6	1.256677167	0.137080273			
206233_at	B4GALT6	1.1835125	1.123589586			
235333_at	B4GALT6	1.098508167	0			
234315_at	BACH1	-0.8152985	0			
1558662_s_at	BANK1	1.502779833	0			
222915_s_at	BANK1	1.58909	0			
212384_at	BAT1	0.575168167	1.455154512			
201255_x_at	BAT3	0.645265	1.123589586			
210208_x_at	BAT3	0.584468333	1.455154512			
213318_s_at	BAT3	0.584495	1.455154512			
209958_s_at	BBS9	0.567023	0.796301331			
37549_g_at	BBS9	0.6530195	1.123589586			
205084_at	BCAP29	0.597771	0.550502435			
218264_at	BCCIP	-0.582171833	0.796301331			
219497_s_at	BCL11A	1.159932667	0	BCL11A		BCL11A
219498_s_at	BCL11A	1.105578	1.810509752	BCL11A		BCL11A
222891_s_at	BCL11A	0.7592285	1.810509752	BCL11A		BCL11A
222895_s_at	BCL11B	1.708575	1.123589586			
206665_s_at	BCL2L1	-0.661481667	3.701646186			
212312_at	BCL2L1	-1.257424667	2.499099453			
217955_at	BCL2L13	0.560507667	0.05396551			
209311_at	BCL2L2	0.950831667	0			
203796_s_at	BCL7A	1.629843	0.550502435			
1560683_at	BCL8	0.896146333	3.701646186			
228065_at	BCL9L	-0.698508667	1.810509752			
206382_s_at	BDNF	-0.839541667	0			
239367_at	BDNF	-0.846116333	1.455154512			
1567359_at	BDNFOS	-1.722383667	0.393887585			
227920_at	BEND3	0.6663145	0.393887585			
218056_at	BFAR	0.716290667	1.455154512			
1556051_a_at	BICD1	0.878193333	0.393887585			
210538_s_at	BIRC3	1.860895667	0			
202094_at	BIRC5	-1.040222667	1.455154512	BIRC5		
202095_s_at	BIRC5	-0.947665833	1.810509752	BIRC5		
221478_at	BNIP3L	0.625123	2.499099453			
203502_at	BPGM	0.786627833	0.796301331			
213473_at	BRAP	0.5462015	3.701646186	BRAP		
202227_s_at	BRD8	-0.914730167	0			
223376_s_at	BRI3	-0.562231167	1.810509752			
221703_at	BRIP1	-0.708904167	2.499099453			
219280_at	BRWD1	0.541428667	0.796301331			
1553252_a_at	BRWD3	0.854608333	0.05396551			
244738_at	BRWD3	1.146628333	0.137080273			
205715_at	BST1	-0.529531667	2.499099453			
225389_at	BTBD6	0.626390167	1.123589586			
1556000_s_at	BTBD7	0.847543333	0			

Probe Set ID	Gene Symbol	miR-128-miRNC	q value (%)	miR-128 tgts_M	miR-128 tgts_TS	miR-128 tgts_PT
203504_s_at	ABCA1	-1.2890325	0	ABCA1	ABCA1	ABCA1
224943_at	BTBD7	1.1045675	0.796301331			
224945_at	BTBD7	1.1410855	0.393887585			
1569693_at	BTBD8	0.644544	3.701646186			
238675_x_at	BTF3L4	0.562466667	2.499099453			
207485_x_at	BTN3A1	0.697536167	1.455154512			
209770_at	BTN3A1	1.152715333	0			
209846_s_at	BTN3A2	0.825626667	0.393887585	BTN3A2		
212613_at	BTN3A2	0.9276005	0.05396551	BTN3A2		
204820_s_at	BTN3A2 /// BTN3A3	0.7487675	0			
204821_at	BTN3A3	0.638410667	1.123589586			
204901_at	BTRC	0.819848667	1.123589586			
216091_s_at	BTRC	0.682468333	0.550502435			
216277_at	BUB1	-0.521303333	2.499099453	BUB1		
201458_s_at	BUB3	-0.687018333	1.123589586	BUB3		
209974_s_at	BUB3	-0.72436	0.393887585	BUB3		
223853_at	BVES	0.747241333	0.550502435	BVES		
234243_at	BXDC5	-1.09763	3.701646186			
234244_at	BXDC5	-1.845528167	3.029425085			
224664_at	C10orf104	0.599195833	3.029425085			
223703_at	C10orf11	0.529778333	2.499099453			
229399_at	C10orf118	0.583668333	1.455154512			
219601_s_at	C10orf12	0.527707167	1.123589586			
202808_at	C10orf26	-0.915817333	0	C10orf26	C1orf26	
225192_at	C10orf46	0.756811	0.393887585			
227257_s_at	C10orf46	1.122039	0.05396551			
230051_at	C10orf47	-0.709367167	1.455154512	C10orf47		
224435_at	C10orf58	0.622309	1.123589586	C10orf58		
228155_at	C10orf58	0.514093167	0.796301331	C10orf58		
232230_at	C10orf75	0.5606105	0.796301331			
238794_at	C10orf78	0.742217667	1.810509752			
219240_s_at	C10orf88	0.590501333	0.796301331			
222852_at	C10orf88	0.590055833	1.810509752			
220987_s_at	C11orf17 /// NUAK2	0.99237	1.123589586			
52164_at	C11orf24	0.604069	3.701646186			
228331_at	C11orf31	1.236574833	3.029425085			
235486_at	C11orf41	-0.9285525	0	C11orf41	C11orf41	
1553787_at	C11orf45	-0.855273833	0	C11orf45		
221652_s_at	C12orf11	0.56542	0.796301331			
224759_s_at	C12orf23	0.682026	1.810509752	C12orf23		
204521_at	C12orf24	0.6092735	0.230227638			
229018_at	C12orf26	0.74395	0.550502435			
227245_at	C12orf30	0.560622	1.810509752			
229778_at	C12orf39	1.055458333	0.393887585			
225772_s_at	C12orf62	-0.8025565	1.123589586			
223476_s_at	C12orf65	0.613259167	1.455154512			
1553141_at	C13orf31	0.920970333	0.393887585	C13orf31		
1553142_at	C13orf31	1.221167833	0.137080273	C13orf31		
228937_at	C13orf31	0.897141333	0.550502435	C13orf31		

Probe Set ID	Gene Symbol	miR-128-miRNC	q value (%)	miR-128 tgts_M	miR-128 tgts_TS	miR-128 tgts_PT
203504_s_at	ABCA1	-1.2890325	0	ABCA1	ABCA1	ABCA1
219757_s_at	C14orf101	0.502255167	2.499099453			
226630_at	C14orf106	-0.519193667	2.499099453			
241816_at	C14orf106	-0.6827525	1.455154512			
219720_s_at	C14orf118	0.733957667	0.393887585			
229520_s_at	C14orf118	0.6133365	3.701646186			
219563_at	C14orf139	1.3673325	0			
223273_at	C14orf142	-0.552032	1.455154512			
232635_at	C14orf145	-0.949005167	0.230227638	C14orf145		
225514_at	C14orf21	0.573385167	3.029425085			
223474_at	C14orf4	0.861723167	3.701646186	C14orf4		
235430_at	C14orf43	0.676577333	3.701646186			
228666_at	C15orf38	-0.782378167	1.810509752	C15orf38		
1552310_at	C15orf40	-0.603085	0.393887585			
232506_s_at	C15orf41	0.574771667	0.137080273			
1560814_a_at	C15orf57	0.735607	0			
230281_at	C16orf46	1.070141	0.230227638	C16orf46		
225088_at	C16orf63	0.676668667	0.393887585			
1568954_s_at	C16orf72	0.575273167	1.123589586			
228373_at	C16orf72	0.873286333	0			
213235_at	C16orf88	0.641108833	3.029425085			
229071_at	C17orf100	0.562540833	3.701646186			
220058_at	C17orf39	0.862032333	0			
223401_at	C17orf48	0.5420905	3.029425085	C17orf48		
224574_at	C17orf49	0.519571667	3.701646186			
238166_s_at	C17orf51	1.198373	0			
238167_at	C17orf51	0.568287	0.393887585			
218464_s_at	C17orf63	0.601171333	0.550502435		C17orf63	
222641_s_at	C17orf63	0.759881667	0		C17orf63	
219435_at	C17orf68	0.824358	1.810509752			
222779_s_at	C17orf85	-0.583936167	0.230227638	C17orf85	C17orf85	C17orf85
224957_at	C18orf32	1.209317167	0.137080273			
1553652_a_at	C18orf54	-0.602908667	0.550502435			
229442_at	C18orf54	-0.709159167	1.810509752			
244324_at	C18orf54	-0.857066167	0.796301331			
221764_at	C19orf22	0.773414167	2.499099453			
223631_s_at	C19orf33	-0.643989333	3.701646186			
221988_at	C19orf42	1.399458667	0			
1552862_at	C1orf104	0.8347065	0			
230256_at	C1orf104	0.874281667	0.796301331			
204699_s_at	C1orf107	0.648977833	1.123589586	C1orf107		
204700_x_at	C1orf107	1.031022667	0.550502435	C1orf107		
1555112_a_at	C1orf114	1.3301925	0.550502435			
223124_s_at	C1orf128	-0.534750167	3.029425085	C1orf128	C1orf128	
212002_at	C1orf144	-1.119380667	0.550502435	C1orf144	C1orf144	C1orf144
212003_at	C1orf144	-1.2811905	0.137080273	C1orf144	C1orf144	C1orf144
212004_at	C1orf144	-0.997545	0	C1orf144	C1orf144	C1orf144
212005_at	C1orf144	-1.264864667	0.05396551	C1orf144	C1orf144	C1orf144
233750_s_at	C1orf25	0.883453667	0.137080273			
221222_s_at	C1orf56	1.109096	0			
209006_s_at	C1orf63	0.622700167	1.123589586			

Probe Set ID	Gene Symbol	miR-128-miRNC	q value (%)	miR-128 tgts_M	miR-128 tgts_TS	miR-128 tgts_PT
203504_s_at	ABCA1	-1.2890325	0	ABCA1	ABCA1	ABCA1
1554660_a_at	C1orf71	0.677947	1.810509752			
1554661_s_at	C1orf71	0.704310667	0.230227638			
225551_at	C1orf71	0.532476333	0.796301331			
218448_at	C20orf11	0.526951667	3.029425085			
1554818_s_at	C20orf12	0.945855833	0.393887585			
223951_at	C21orf116	0.747951333	0.796301331			
1552604_at	C21orf74	-0.7464275	3.029425085			
1552605_s_at	C21orf74	-0.623754167	1.455154512			
220941_s_at	C21orf91	1.208649333	0			
226109_at	C21orf91	1.383326333	0			
224695_at	C2orf29	-0.573965	1.123589586			
219128_at	C2orf42	1.1022195	0			
231252_at	C2orf67	-0.865234833	3.701646186			
238768_at	C2orf68	0.833830833	0			
1558094_s_at	C3orf19	0.838474667	0			
226891_at	C3orf21	-0.602704	0.230227638			
1553158_at	C3orf34	0.600208667	2.499099453			
219474_at	C3orf52	1.236992833	0.230227638			
241817_at	C3orf62	0.819931	0.796301331			
1562953_s_at	C4orf12	0.6713295	2.499099453			
231010_at	C4orf16	1.007322333	0			
242753_x_at	C4orf16	0.998847333	0			
219450_at	C4orf19	0.559093167	1.810509752			
228859_at	C4orf21	-0.596592167	2.499099453			
231204_at	C4orf21	-0.5022575	3.029425085			
219747_at	C4orf31	2.971016	0			
238015_at	C4orf46	-0.973276	0			
238411_x_at	C5orf13	1.474635	0	C5orf13	C5orf13	C5orf13
1553108_at	C5orf24	0.893259333	1.123589586			
229886_at	C5orf34	-0.675788833	3.701646186			
1554229_at	C5orf41	0.699487833	0		C5orf41	C5orf41
225956_at	C5orf41	0.541032667	3.701646186		C5orf41	C5orf41
225957_at	C5orf41	1.0642765	0.550502435		C5orf41	C5orf41
238476_at	C5orf41	1.0436475	2.499099453		C5orf41	C5orf41
226977_at	C5orf53	0.889788167	0			
241874_at	C5orf53	0.535202833	0.796301331			
229070_at	C6orf105	0.520293333	1.455154512			
205457_at	C6orf106	0.641718333	1.810509752			
217924_at	C6orf106	1.241839833	0			
217925_s_at	C6orf106	1.006578	0			
1554486_a_at	C6orf114	-1.103231667	2.499099453			
1559051_s_at	C6orf150	0.797299667	0.550502435			
220324_at	C6orf155	-1.008844167	2.499099453			
1553726_s_at	C6orf170	1.414555833	3.701646186			
223576_at	C6orf203	0.76587	0.137080273			
229762_at	C7orf38	1.205118333	0			
218008_at	C7orf42	-0.926695833	0	C7orf42	C7orf42	
224688_at	C7orf42	-0.703197333	0.137080273	C7orf42	C7orf42	
206497_at	C7orf44	0.5493235	3.029425085			
209446_s_at	C7orf44	0.778491667	0			

Probe Set ID	Gene Symbol	miR-128-miRNC	q value (%)	miR-128 tgts_M	miR-128 tgts_TS	miR-128 tgts_PT
203504_s_at	ABCA1	-1.2890325	0	ABCA1	ABCA1	ABCA1
228728_at	C7orf58	-0.621921167	1.810509752	C7orf58		
244569_at	C8orf37	0.738647667	3.701646186			
1556571_at	C8orf38	-0.704999167	2.499099453	C8orf38		
220038_at	C8orf44 /// SGK3	0.957291167	1.123589586			
224158_s_at	C8orf83	0.662110667	3.029425085			
223007_s_at	C9orf5	0.648513333	1.123589586	C9orf5		
227831_at	C9orf5	0.570615	3.029425085	C9orf5		
218998_at	C9orf6	-0.565850833	1.810509752	C9orf6		
1554708_s_at	C9orf68	0.965647333	0.05396551			
221865_at	C9orf91	1.075579333	0			
219147_s_at	C9orf95	1.043053667	0			
231270_at	CA13	1.047719333	0.796301331			
235899_at	CA13	0.757779333	3.029425085			
239106_at	CA5BP	0.933861333	0.393887585			
243173_at	CABP7	-0.579264333	3.701646186			
207050_at	CACNA2D1	0.778586167	0.137080273			
34726_at	CACNB3	0.703218	1.455154512			
207693_at	CACNB4	0.913894833	0.230227638			
209002_s_at	CALCOCO1	0.517841	1.455154512			
206331_at	CALCRL	2.013195	0			
210815_s_at	CALCRL	2.137966833	0			
201615_x_at	CALD1	0.59426	0.550502435	CALD1		
211984_at	CALM1	0.533213333	1.810509752			
211985_s_at	CALM1	0.683415	0.393887585			
213688_at	CALM1	0.746340333	0.550502435			
200623_s_at	CALM3	-0.905597667	0.230227638			
224994_at	CAMK2D	-0.583965667	0.05396551	CAMK2D		
210787_s_at	CAMKK2	0.512802667	1.455154512	CAMKK2		
212763_at	CAMSAP1L1	0.617231667	0.230227638			
217196_s_at	CAMSAP1L1	0.819017333	0.796301331			
208838_at	CAND1	-0.686739	1.123589586	CAND1		
208839_s_at	CAND1	-0.807773667	0.393887585	CAND1		
238034_at	CANX	0.628186167	2.499099453	CANX		
212551_at	CAP2	0.73764	0.230227638	CAP2		
1555608_at	CAPRIN2	0.775828667	0.137080273			
218456_at	CAPRIN2	0.748045	0.230227638			
208374_s_at	CAPZA1	-0.918563333	0.05396551	CAPZA1		CAPZA1
1552701_a_at	CARD16	1.142377167	0.05396551			
1552703_s_at	CARD16 /// CASP1	1.065073333	0			
1554479_a_at	CARD8	0.781267667	1.455154512			
204950_at	CARD8	0.8491945	0			
240983_s_at	CARS	0.636835333	1.810509752			
207842_s_at	CASC3	-0.696716167	0.796301331	CASC3	CASC3	CASC3
211208_s_at	CASK	-0.549386667	1.810509752			
1552689_at	CASKIN1	-0.819886	0.550502435			
206011_at	CASP1	0.951063167	0.550502435	CASP1		

Probe Set ID	Gene Symbol	miR-128-miRNC	q value (%)	miR-128 tgts_M	miR-128 tgts_TS	miR-128 tgts_PT
203504_s_at	ABCA1	-1.2890325	0	ABCA1	ABCA1	ABCA1
209970_x_at	CASP1	0.789382333	0.05396551	CASP1		
211366_x_at	CASP1	0.7275155	0	CASP1		
211367_s_at	CASP1	0.798197333	0.137080273	CASP1		
211368_s_at	CASP1	0.885092333	0	CASP1		
213596_at	CASP4	0.646818	0.393887585			
207181_s_at	CASP7	0.81774	0.550502435			
203065_s_at	CAV1	-0.94296	0	CAV1		
212097_at	CAV1	-1.287760333	0	CAV1		
202370_s_at	CBFB	-0.690856667	1.123589586	CBFB		CBFB
209682_at	CBLB	0.526430333	3.029425085			
209715_at	CBX5	-0.504221667	1.455154512			
212126_at	CBX5	-0.609386833	0.796301331			
48117_at	CCDC101	0.652898833	3.701646186			
225320_at	CCDC109A	0.676608	2.499099453	CCDC109A		
225644_at	CCDC117	0.5578445	2.499099453			
235330_at	CCDC117	0.53485	1.123589586			
1553542_at	CCDC125	0.627834333	0.796301331	CCDC125		
229082_at	CCDC125	0.853334	0.550502435	CCDC125		
1554217_a_at	CCDC132	0.593985	1.455154512			
1561054_a_at	CCDC14	0.602107333	1.810509752			
226723_at	CCDC23	0.6289405	1.123589586			
218655_s_at	CCDC49	0.560745	0.230227638			
226713_at	CCDC50	-1.389393833	0			
228693_at	CCDC50	-1.229145	0			
235051_at	CCDC50	-0.969500667	0			
236831_at	CCDC50	-0.647403167	3.701646186			
218026_at	CCDC56	-0.561048333	2.499099453			
1553102_a_at	CCDC69	1.311803	0			
212886_at	CCDC69	1.512974333	0			
1553214_a_at	CCDC7	1.025811167	0.230227638	CCDC7		
221791_s_at	CCDC72	-0.502466667	1.123589586	CCDC72		
1559893_at	CCDC75	0.530986167	1.810509752			
228495_at	CCDC75	0.519269333	2.499099453			
232489_at	CCDC76	0.545562	3.029425085			
204609_at	CCDC85B	-1.131246167	0.796301331			
222809_x_at	CCDC85C	-0.563827333	3.029425085			
238759_at	CCDC88A	0.828843833	3.029425085	CCDC88A	CCDC88A	CCDC88A
215343_at	CCDC88C	1.09639	0.796301331	CCDC88C	CCDC88C	
209689_at	CCDC93	0.888924333	0.550502435			
221685_s_at	CCDC99	-0.614070333	2.499099453			
205899_at	CCNA1	1.912486833	0			
213226_at	CCNA2	-0.578099167	0.393887585			
200952_s_at	CCND2	0.715329	0.137080273			
200953_s_at	CCND2	0.812153167	1.123589586			
223084_s_at	CCNDBP1	0.821	0			
204826_at	CCNF	-0.712188833	3.029425085	CCNF		
227299_at	CCNI	0.559256667	1.810509752			
206967_at	CCNT1	0.656729333	0.796301331			
220671_at	CCRN4L	0.7249915	0.393887585			
226545_at	CD109	-0.933740833	0			

Probe Set ID	Gene Symbol	miR-128-miRNC	q value (%)	miR-128 tgts_M	miR-128 tgts_TS	miR-128 tgts_PT
203504_s_at	ABCA1	-1.2890325	0	ABCA1	ABCA1	ABCA1
229900_at	CD109	-0.724272	0.796301331			
239719_at	CD109	-0.633131667	1.810509752			
223834_at	CD274	1.149527333	0.05396551	CD274		
205264_at	CD3EAP	1.272541667	0			
215346_at	CD40	0.643827333	1.810509752	CD40		
222292_at	CD40	1.172888167	0	CD40		
205173_x_at	CD58	0.549958333	0.796301331			
211744_s_at	CD58	0.6218485	0.230227638			
216322_at	CD58	0.527794333	3.029425085			
216942_s_at	CD58	0.524745167	3.029425085			
204440_at	CD83	1.138902667	3.701646186			
233825_s_at	CD99L2	-0.684472333	0			
210440_s_at	CDC14A	1.028181333	0			
210743_s_at	CDC14A	1.399892833	0			
203213_at	CDC2	-0.8156515	0.550502435			
203214_x_at	CDC2	-0.697675333	1.123589586			
210559_s_at	CDC2	-0.7739345	0.230227638			
201853_s_at	CDC25B	-0.987032833	0			
205167_s_at	CDC25C	-0.916745667	0.393887585			
216914_at	CDC25C	-0.9046735	0.230227638			
217010_s_at	CDC25C	-0.828959	1.810509752			
212897_at	CDC2L6	0.567536	0.393887585			
219343_at	CDC37L1	0.8256225	0.230227638			
228561_at	CDC37L1	0.8667225	0			
209286_at	CDC42EP3	-0.570335	1.123589586			
209287_s_at	CDC42EP3	-0.72657	0.05396551			
209288_s_at	CDC42EP3	-0.543801667	3.701646186			
1552612_at	CDC42SE2	0.734813333	0.393887585			
1552613_s_at	CDC42SE2	0.684968	1.455154512			
221436_s_at	CDCA3	-0.531626833	3.701646186			
224753_at	CDCA5	-0.7588185	3.029425085			
221520_s_at	CDCA8	-1.217812833	0.393887585			
213348_at	CDKN1C	-1.656151167	0.230227638	CDKN1C		
218929_at	CDKN2AIP	0.532022833	3.701646186			
207276_at	CDR1	1.220619833	1.810509752			
228482_at	CDRT4 /// FAM18B2	0.708701833	3.029425085			
213003_s_at	CEBPD	-1.084828167	0.393887585			
204962_s_at	CENPA	-0.728420833	0			
210821_x_at	CENPA	-0.7493255	1.810509752			
204739_at	CENPC1	-0.6884365	1.810509752			
205046_at	CENPE	-0.695516167	0			
207828_s_at	CENPF	-0.51944	2.499099453			
209172_s_at	CENPF	-0.871221833	0			
207590_s_at	CENPI	-0.676614833	0.393887585			
214804_at	CENPI	-0.857710833	0.550502435			
242916_at	CEP110	0.780606667	1.455154512			
239413_at	CEP152	-0.875257	0.05396551			
1558953_s_at	CEP164	0.64779	0.393887585			
218542_at	CEP55	-0.767978333	0.796301331	CEP55		

Probe Set ID	Gene Symbol	miR-128-miRNC	q value (%)	miR-128 tgts_M	miR-128 tgts_TS	miR-128 tgts_PT
203504_s_at	ABCA1	-1.2890325	0	ABCA1	ABCA1	ABCA1
200021_at	CFL1	-0.766976667	1.810509752			
224352_s_at	CFL2	-0.605789167	1.123589586			
215105_at	CG030	-0.500110667	1.810509752			
203975_s_at	CHAF1A	-0.848992167	1.123589586			
229808_at	CHAF1A	-0.658164	2.499099453			
218642_s_at	CHCHD7	-0.712799667	0			
220619_at	CHD7	-0.764704	1.455154512			
1559481_at	CHIC1	0.828066833	3.701646186			
210069_at	CHKB-CPT1B /// CPT1B	1.214557	0			
218178_s_at	CHMP1B	-0.521118333	1.455154512			
218571_s_at	CHMP4A	0.535305	3.701646186			
226803_at	CHMP4C	1.292847167	0			
215916_at	CHRNE	0.5258635	3.701646186			
226368_at	CHST11	0.64679	1.455154512	CHST11		
226314_at	CHST14	0.833012167	1.455154512			
208252_s_at	CHST3	0.522639667	3.029425085			
200810_s_at	CIRBP	-1.456406333	0.230227638	CIRBP		
200811_at	CIRBP	-1.447496833	0.137080273	CIRBP		
212801_at	CIT	-0.835518667	1.123589586			CIT
207980_s_at	CITED2	-1.2153855	0	CITED2	CITED2	CITED2
209357_at	CITED2	-0.826916167	0.05396551	CITED2	CITED2	CITED2
213976_at	CIZ1	0.734210167	3.701646186			
1554264_at	CKAP2	-0.862949	0.393887585			
218252_at	CKAP2	-0.657015667	1.810509752			
201897_s_at	CKS1B	-0.700498333	3.029425085			
204170_s_at	CKS2	-0.689175	0.796301331			
1555543_a_at	CLCC1	0.633232	1.455154512			
207855_s_at	CLCC1	0.539232833	1.810509752			
201733_at	CLCN3	0.827458667	0.796301331	CLCN3		CLCN3
201734_at	CLCN3	0.598912333	1.455154512	CLCN3		CLCN3
201735_s_at	CLCN3	0.599272	2.499099453	CLCN3		CLCN3
223249_at	CLDN12	1.262943667	0			
220332_at	CLDN16	0.762344667	1.123589586			
209732_at	CLEC2B	1.09108	0			
219944_at	CLIP4	0.665638333	0.137080273	CLIP4		
204084_s_at	CLN5	0.621242833	0.393887585			
214252_s_at	CLN5	0.875791333	0			
222539_at	CLN6	-0.677670667	3.701646186			
204980_at	CLOCK	1.004254833	0.137080273			
1566151_at	CLP1	-1.869829833	0	CLP1		
204370_at	CLP1	-0.703512167	0	CLP1		
202799_at	CLPP	-0.741013	3.029425085	CLPP	CLPP	
204809_at	CLPX	0.523236667	1.123589586			
223507_at	CLPX	0.683203	1.810509752			
224733_at	CMTM3	-0.819308333	0			
217752_s_at	CNDP2	0.514230333	2.499099453			
200860_s_at	CNOT1	-0.505276667	1.123589586			
210203_at	CNOT4	0.643418167	0.550502435	CNOT4		

Probe Set ID	Gene Symbol	miR-128-miRNC	q value (%)	miR-128 tgts_M	miR-128 tgts_TS	miR-128 tgts_PT
203504_s_at	ABCA1	-1.2890325	0	ABCA1	ABCA1	ABCA1
210866_s_at	CNOT4	0.638982	1.123589586	CNOT4		
217970_s_at	CNOT6	-0.748280833	0	CNOT6	CNOT6	CNOT6
222476_at	CNOT6	-0.797902667	1.123589586	CNOT6	CNOT6	CNOT6
208597_at	CNTF /// ZFP91 /// ZFP91-CNTF	-0.753585333	3.029425085			
239989_at	CNTLN	-0.874934333	1.455154512	CNTLN		
203641_s_at	COBLL1	0.665418833	1.123589586	COBLL1		
215393_s_at	COBLL1	0.612853333	3.029425085	COBLL1		
225769_at	COG6	0.513846667	2.499099453			
231766_s_at	COL12A1	-0.707477167	1.810509752			
211343_s_at	COL13A1	0.782471667	0.796301331			
1556499_s_at	COL1A1	1.265750667	0			
202310_s_at	COL1A1	0.9854575	0			
225293_at	COL27A1	0.856627833	0.550502435			
221019_s_at	COLEC12	-2.1475835	0			
218439_s_at	COMMD10	0.696526667	0.230227638			
222637_at	COMMD10	0.986193167	0.393887585			
214337_at	COPA	1.049311333	0.137080273			
219397_at	COQ10B	0.7087455	0.796301331			
221676_s_at	CORO1C	-0.556356667	0.796301331	CORO1C	CORO1C	CORO1C
214277_at	COX11	0.758090667	3.029425085			
203880_at	COX17	0.607345	0.550502435			
235533_at	COX19	1.061341667	0.393887585			
218057_x_at	COX4NB	-0.563154333	1.455154512			
222504_s_at	COX4NB	-0.7147105	0.796301331			
227323_at	COX4NB	-0.8814145	1.455154512			
227363_s_at	COX4NB	-0.515366333	1.123589586			
202110_at	COX7B	-0.623295	0.393887585	COX7B		
201119_s_at	COX8A	-0.940352167	0.137080273	COX8A		
204662_at	CP110	-0.675386167	3.701646186			
222903_s_at	CPEB1	0.993935833	0			
226939_at	CPEB2	-0.520029333	1.810509752			
224828_at	CPEB4	0.576125333	1.810509752		CPEB4	CPEB4
224829_at	CPEB4	0.964438167	0.393887585		CPEB4	CPEB4
217557_s_at	CPM	-1.926951667	0			
243727_at	CPNE8	0.95158	0.230227638	CPNE8		
204920_at	CPS1	0.632080333	3.701646186			
217564_s_at	CPS1	1.039897833	1.455154512			
225565_at	CREB1	0.758965333	0.393887585			CREB1
225572_at	CREB1	0.698837667	3.701646186			CREB1
229228_at	CREB5	1.021611667	1.810509752			
201988_s_at	CREBL2	0.804431667	0			
201989_s_at	CREBL2	0.601861667	0.796301331			
201990_s_at	CREBL2	0.903065667	0			
202978_s_at	CREBZF	0.557802	2.499099453			
201200_at	CREG1	0.742873333	0.796301331			
203368_at	CRELD1	0.813908167	0.550502435			
241741_at	CRLS1	-0.685174	0.550502435	CRLS1		
202517_at	CRMP1	-0.560566167	1.810509752			

Probe Set ID	Gene Symbol	miR-128-miRNC	q value (%)	miR-128 tgts_M	miR-128 tgts_TS	miR-128 tgts_PT
203504_s_at	ABCA1	-1.2890325	0	ABCA1	ABCA1	ABCA1
201380_at	CRTAP	-0.510595167	1.810509752			
226656_at	CRTAP	-0.514678333	3.029425085			
227138_at	CRTAP	-1.0478285	0			
209674_at	CRY1	0.574908	3.701646186			
212695_at	CRY2	1.161649333	3.029425085			
206777_s_at	CRYBB2 /// CRYBB2P1	0.580601	1.455154512			
239077_at	CSGALNACT2	0.701412333	1.123589586			
222235_s_at	CSGALNACT2 /// RP1-19N1.1	0.743008333	3.701646186			
208865_at	CSNK1A1	0.850478333	0.393887585	CSNK1A1		
208866_at	CSNK1A1	0.778447333	0.137080273	CSNK1A1		
208867_s_at	CSNK1A1	0.692675	0.230227638	CSNK1A1		
231920_s_at	CSNK1G1	0.636494167	1.455154512			
212073_at	CSNK2A1 /// CSNK2A1P	0.602668333	3.701646186			
221260_s_at	CSRNP2	0.9531435	0.796301331			
225042_s_at	CSRNP2	0.5178905	1.810509752			
202190_at	CSTF1	-0.628886333	1.455154512			
238821_at	CSTF2	0.519454167	3.029425085			
203445_s_at	CTDSP2	-0.699894667	0	CTDSP2	CTDSP2	CTDSP2
201906_s_at	CTDSPL	-0.820845	0		CTDSPL	CTDSPL
225681_at	CTHRC1	0.532535	1.810509752			
223679_at	CTNNB1	0.859392333	1.455154512			
219080_s_at	CTPS2	-0.660626333	0.796301331	CTPS2		
202060_at	CTR9	-0.7882465	0.550502435			
201487_at	CTSC	-0.533928333	0.393887585			
225647_s_at	CTSC	-0.6533015	1.455154512			
202901_x_at	CTSS	1.715497167	0.796301331			
202902_s_at	CTSS	1.227022833	0			
214073_at	CTTN	1.0625095	0			
241747_s_at	CUL7	0.942785	0.230227638			
218970_s_at	CUTC	-0.522719833	0.550502435			
1569203_at	CXCL2	1.029807833	0	CXCL2	CXCL2	
219355_at	CXorf57	0.6428385	3.701646186			
205073_at	CYP2J2	1.465791167	0			
202314_at	CYP51A1	0.535478333	1.123589586			
1555842_at	CYTH2	0.864977833	1.123589586			
212480_at	CYTSA	0.824798833	0			
226666_at	DAAM1	-0.9139395	0			
226020_s_at	DAB1 /// OMA1	0.866582	0.393887585			
228942_s_at	DAB2IP	1.044189167	0			
219179_at	DACT1	0.688756833	0	DACT1		
202428_x_at	DBI	-0.915615	0.05396551	DBI		
209389_x_at	DBI	-0.854556667	0	DBI		
211070_x_at	DBI	-1.006536667	0	DBI		
205369_x_at	DBT	0.977485167	0.550502435			
244687_at	DBT	0.954439	0			

Probe Set ID	Gene Symbol	miR-128-miRNC	q value (%)	miR-128 tgts_M	miR-128 tgts_TS	miR-128 tgts_PT
203504_s_at	ABCA1	-1.2890325	0	ABCA1	ABCA1	ABCA1
219001_s_at	DCAF10	0.759336667	0.796301331			
226938_at	DCAF4	0.896120167	0.393887585			
221744_at	DCAF7	-0.690145	0.393887585	DCAF7		DCAF7
224730_at	DCAF7	-0.6085725	3.029425085	DCAF7		DCAF7
224748_at	DCAF7	-0.56786	0.550502435	DCAF7		DCAF7
222925_at	DCDC2	-1.001533833	0.05396551	DCDC2		
205399_at	DCLK1	1.4085605	0.393887585			
232505_at	DCTN2	0.7957755	0.393887585			
212855_at	DCUN1D4	-0.783080167	1.455154512	DCUN1D4	DCUN1D4	DCUN1D4
1553565_s_at	DDAH1	-0.566136667	0.796301331	DDAH1	DDAH1	
209094_at	DDAH1	-0.63342	1.455154512	DDAH1	DDAH1	
229456_s_at	DDAH1	-1.043041667	0	DDAH1	DDAH1	
243711_at	DDAH1	-1.001291167	0.05396551	DDAH1	DDAH1	
220694_at	DDEF1IT1	-1.47998	0.550502435			
225965_at	DDHD1	0.669032167	0.796301331			
225970_at	DDHD1	0.776318167	0			
244154_at	DDHD1	0.6768685	2.499099453			
208151_x_at	DDX17	0.67629	0.230227638	DDX17		
208719_s_at	DDX17	0.649405	1.123589586	DDX17		
213998_s_at	DDX17	0.681205	1.810509752	DDX17		
201584_s_at	DDX39	-0.765082333	2.499099453			
218943_s_at	DDX58	0.720401	0.796301331			
218986_s_at	DDX60	0.6380575	2.499099453			
219763_at	DENND1A	-1.179005833	0.796301331	DENND1A		
226980_at	DEPDC1B	-0.848204667	0.796301331			
1561114_a_at	DEPDC4	1.167598667	1.810509752			
203733_at	DEXI	0.563202333	3.029425085			
202534_x_at	DHFR	-0.616917167	1.123589586	DHFR		
48808_at	DHFR	-0.7676895	0.05396551	DHFR		
202802_at	DHPS	0.653674167	3.701646186			
218756_s_at	DHRS11	-0.523269333	1.455154512			
202481_at	DHRS3	0.689101667	3.029425085			
227094_at	DHTKD1	-0.758441833	0	DHTKD1		
205603_s_at	DIAPH2	0.51177	1.455154512			
205726_at	DIAPH2	0.7391735	0			
217246_s_at	DIAPH2	0.805049	0.230227638			
212888_at	DICER1	0.8405485	0.137080273			
213229_at	DICER1	0.703863667	3.701646186			
1555301_a_at	DIP2A	0.973043	0.550502435	DIP2A		
1561286_a_at	DIP2A	0.959651833	0.137080273	DIP2A		
224872_at	DIP2B	0.698047333	0.796301331			
212504_at	DIP2C	0.631051333	3.701646186			
214717_at	DKFZp434H 1419	-0.573467333	0.230227638			
229715_at	DKFZp686O 24166	0.614608833	0.393887585			
219908_at	DKK2	1.3448525	1.455154512	DKK2	DKK2	DKK2
202196_s_at	DKK3	0.698371667	0.796301331			
216870_x_at	DLEU2	-1.008364167	0.393887585			
215629_s_at	DLEU2 ///	-1.000967333	0			

Probe Set ID	Gene Symbol	miR-128-miRNC	q value (%)	miR-128 tgts_M	miR-128 tgts_TS	miR-128 tgts_PT
203504_s_at	ABCA1	-1.2890325	0	ABCA1	ABCA1	ABCA1
	DLEU2L					
230229_at	DLG1	-1.021478833	0			
210684_s_at	DLG4	1.129636333	0.550502435			
208086_s_at	DMD	0.577513833	1.123589586	DMD		
1554333_at	DNAJA4	1.287140167	0	DNAJA4		
209015_s_at	DNAJB6	0.796515333	0.137080273			
1554462_a_at	DNAJB9	1.857629	0			
202842_s_at	DNAJB9	1.750745333	0			
202843_at	DNAJB9	2.188857	0			
222620_s_at	DNAJC1	0.948597	0			
222621_at	DNAJC1	0.815874167	0.796301331			
221782_at	DNAJC10	0.501178	3.029425085			
212908_at	DNAJC16	0.662416833	1.455154512	DNAJC16		
212911_at	DNAJC16	0.629164667	2.499099453	DNAJC16		
213853_at	DNAJC24	0.566112833	3.701646186			
1556053_at	DNAJC7	1.099284167	0.230227638			
240144_at	DNASE1	0.6966625	0.796301331			
203912_s_at	DNASE1L1	0.713374667	1.455154512	DNASE1L1		
218457_s_at	DNMT3A	0.7893145	0.230227638			
219279_at	DOCK10	1.020011333	0			
215237_at	DOCK9	0.654685	0.796301331	DOCK9		
209691_s_at	DOK4	0.944619	0.05396551			
40612_at	DOPEY1	0.566782833	2.499099453	DOPEY1		
204514_at	DPH2	0.594276833	2.499099453			
1560916_a_at	DPY19L1	0.712951667	0.230227638			
212792_at	DPY19L1	1.007990667	0			
213391_at	DPY19L4	-0.60687	3.029425085	DPY19L4		
204751_x_at	DSC2	-0.870040333	0.550502435	DSC2		
226817_at	DSC2	-0.815042667	0.393887585	DSC2		
219000_s_at	DSCC1	0.7342775	0.230227638			
232825_s_at	DSEL	-0.706623	0.796301331			
204455_at	DST	-0.902813333	0	DST		
218585_s_at	DTL	-0.7779795	0.137080273			
222680_s_at	DTL	-0.719125	0.796301331			
208430_s_at	DTNA	1.2542555	0	DTNA		DTNA
210736_x_at	DTNA	1.273778667	0	DTNA		DTNA
211493_x_at	DTNA	1.431123667	0	DTNA		DTNA
227084_at	DTNA	0.991009833	0	DTNA		DTNA
236649_at	DTWD1	0.906457167	0			
1553984_s_at	DTYMK	-0.951497	0.137080273			
226440_at	DUSP22	0.6542235	0.796301331			
209457_at	DUSP5	-0.519385667	1.810509752		DUSP5	DUSP5
201908_at	DVL3	1.082679333	0			
223171_at	DYM	0.960408167	0			
211928_at	DYNC1H1	-0.520808333	3.701646186			
219469_at	DYNC2H1	-0.5417395	0.796301331	DYNC2H1		
201999_s_at	DYNLT1	-0.657522667	1.123589586	DYNLT1		
204557_s_at	DZIP1	0.630069333	2.499099453	DZIP1		
228033_at	E2F7	-0.946692	0.796301331	E2F7	E2F7	E2F7
241725_at	E2F7	-0.51414	1.810509752	E2F7	E2F7	E2F7

Probe Set ID	Gene Symbol	miR-128-miRNC	q value (%)	miR-128 tgts_M	miR-128 tgts_TS	miR-128 tgts_PT
203504_s_at	ABCA1	-1.2890325	0	ABCA1	ABCA1	ABCA1
219551_at	EAF2	1.062586333	0.05396551	EAF2		
227374_at	EARS2	0.516359167	3.701646186	EARS2		
201750_s_at	ECE1	-1.116269167	0.550502435	ECE1	ECE1	
201135_at	ECHS1	-0.64408	3.029425085			
227780_s_at	ECSCR	-0.576438833	1.810509752			
221399_at	EDA2R	0.502463333	3.029425085	EDA2R		
220342_x_at	EDEM3	0.568167833	1.455154512		EDEM3	EDEM3
204840_s_at	EEA1	0.655246667	2.499099453			
1553392_at	EFCAB3	1.5158365	3.029425085			
226800_at	EFCAB7	0.690949667	0.137080273			
202668_at	EFNB2	1.05726	1.123589586			
202669_s_at	EFNB2	0.856393	0			
218973_at	EFTUD1	0.761323	0			
1565483_at	EGFR	-0.537320667	3.701646186			EGFR
201693_s_at	EGR1	4.9807615	0			
201694_s_at	EGR1	5.650494333	0			
227404_s_at	EGR1	5.54022	0			
208669_s_at	EID1	-0.64464	1.455154512	EID1		
211698_at	EID1	-0.7358825	1.810509752	EID1		
231292_at	EID3	-1.069109667	0.550502435	EID3		
219426_at	EIF2C3	0.577047333	3.029425085	EIF2C3		
208725_at	EIF2S2	-1.246912167	0	EIF2S2	EIF2S2	
242550_at	EIF3B	1.064920833	2.499099453			
208770_s_at	EIF4EBP2	0.700755	0.550502435			
224645_at	EIF4EBP2	0.741945667	0.05396551			
208707_at	EIF5	-0.651520333	0.05396551		EIF5	
220198_s_at	EIF5A2	0.777270167	0.796301331			
235296_at	EIF5A2	0.595167333	3.701646186			
203490_at	ELF4	0.956906667	0.05396551	ELF4		
31845_at	ELF4	0.9510475	0	ELF4		
220363_s_at	ELMO2	0.5114775	2.499099453	ELMO2		
218028_at	ELOVL1	-0.717112167	1.810509752	ELOVL1		
57163_at	ELOVL1	-0.756754333	0.393887585	ELOVL1		
227075_at	ELP3	0.841878167	0.137080273			
204398_s_at	EML2	0.670081833	1.810509752			
201324_at	EMP1	-0.52266	2.499099453			
201325_s_at	EMP1	-0.587843333	1.810509752			
213895_at	EMP1	-0.735138333	0.550502435			
201341_at	ENC1	0.531054167	1.810509752			
201313_at	ENO2	0.833903	0	ENO2		
228682_at	ENOPH1	0.619921167	1.123589586			
204160_s_at	ENPP4	1.238764	0			
220153_at	ENTPD7	0.632297167	0			
226775_at	ENY2	-0.680133667	1.455154512			
217050_at	EPAG	0.70165	0.137080273			
1554481_a_at	EPB41	0.716	0.796301331		EPB41	
214530_x_at	EPB41	0.7301445	1.455154512		EPB41	
220161_s_at	EPB41L4B	0.952618167	1.455154512			
223427_s_at	EPB41L4B	1.109225333	0			
206114_at	EPHA4	1.284071333	0			

Probe Set ID	Gene Symbol	miR-128-miRNC	q value (%)	miR-128 tgts_M	miR-128 tgts_TS	miR-128 tgts_PT
203504_s_at	ABCA1	-1.2890325	0	ABCA1	ABCA1	ABCA1
227449_at	EPHA4	1.390955833	1.810509752			
228948_at	EPHA4	1.837396833	0			
229374_at	EPHA4	1.160378833	0.137080273			
210753_s_at	EPHB1	-1.273425667	0	EPHB1		
211898_s_at	EPHB1	-1.411022667	0	EPHB1		
230425_at	EPHB1	-1.2920955	0	EPHB1		
209589_s_at	EPHB2	0.676266167	1.810509752	EPHB2	EPHB2	EPHB2
203463_s_at	EPN2	0.867781833	1.810509752			
1555826_at	EPR1	-0.9612655	3.029425085			
210385_s_at	ERAP1	0.609392	0.393887585			
1554273_a_at	ERAP2	0.798373333	0.796301331			
219759_at	ERAP2	0.611111333	1.123589586			
217941_s_at	ERBB2IP	-0.618632167	0.796301331	ERBB2IP		
222473_s_at	ERBB2IP	-0.630966667	0.550502435	ERBB2IP		
215606_s_at	ERC1	0.844471	0.137080273			
228131_at	ERCC1	-1.287743833	0			
230108_at	ERCC6	0.680196333	1.810509752			
202441_at	ERLIN1	0.530608	0.550502435	ERLIN1		
202444_s_at	ERLIN1	0.578411667	0.796301331	ERLIN1		
218342_s_at	ERMP1	0.568858333	1.123589586			
222603_at	ERMP1	0.650387333	1.810509752			
207061_at	ERN1	1.088482	3.029425085			
220012_at	ERO1LB	1.342514833	0	ERO1LB		
235588_at	ESCO2	-0.6973165	1.123589586	ESCO2		
208394_x_at	ESM1	0.839423333	2.499099453			
219121_s_at	ESRP1	1.784737833	0			
208858_s_at	ESYT1	0.644788333	1.123589586			
1555829_at	ESYT2	1.672979167	0			
201574_at	ETF1	0.904732667	0			
224454_at	ETNK1	-0.806979167	0.550502435			
219432_at	EVC	-0.580122667	0.796301331	EVC		
204774_at	EVI2A	-0.606490833	1.123589586			
218363_at	EXD2	0.591619833	1.455154512			
224253_at	EXOC5	0.830283	1.810509752			
233924_s_at	EXOC6	0.933500167	0			
1559044_at	EXOSC1	0.798506	1.123589586	EXOSC1		
227696_at	EXOSC6	0.578764833	1.123589586			
1552314_a_at	EYA3	0.813835833	0.393887585			
206379_at	EYA3	0.813787333	0.393887585			
226170_at	EYA3	0.6192655	3.701646186			
211310_at	EZH1	1.518325	0	EZH1		
32259_at	EZH1	0.674427167	0.230227638	EZH1		
221664_s_at	F11R	0.904291667	0.137080273			
206429_at	F2RL1	2.696702667	0			
213506_at	F2RL1	2.320999833	0			
204363_at	F3	-0.764963333	0.137080273	F3	F3	
220285_at	FAM108B1	-0.7099855	2.499099453	FAM108B1	FAM18B1	
228872_at	FAM108B1	-0.690726333	3.701646186	FAM108B1	FAM18B1	
226697_at	FAM114A1	0.556128333	1.455154512			
218588_s_at	FAM114A2	0.789795	1.123589586			

Probe Set ID	Gene Symbol	miR-128-miRNC	q value (%)	miR-128 tgts_M	miR-128 tgts_TS	miR-128 tgts_PT
203504_s_at	ABCA1	-1.2890325	0	ABCA1	ABCA1	ABCA1
219629_at	FAM118A	0.659096333	1.810509752			
1553743_at	FAM119A	0.756456	1.810509752			
235177_at	FAM119A	0.5399755	3.701646186			
235931_at	FAM119A	0.5401475	3.029425085			
226774_at	FAM120B	-0.517241333	0.393887585			
222673_x_at	FAM122B	-0.8918295	0.796301331	FAM122B		
225361_x_at	FAM122B	-0.870126833	1.123589586	FAM122B		
201828_x_at	FAM127A	-0.793982833	1.455154512			
212995_x_at	FAM128B	-0.721113333	1.123589586			
221904_at	FAM131A	0.5389135	2.499099453			
218037_at	FAM134A	0.5148375	3.029425085			
221983_at	FAM134A	0.655427333	0.230227638			
221984_s_at	FAM134A	0.5386775	1.455154512			
223497_at	FAM135A	0.877333	3.029425085	FAM135A		
213896_x_at	FAM149B1	0.554656667	1.810509752			
238859_at	FAM149B1	0.567387333	1.455154512			
214825_at	FAM155A	-0.544078833	1.810509752	FAM155A	FAM155A	
1569465_at	FAM160A1	1.270455833	1.455154512			
242687_at	FAM160A1	1.107036333	0.550502435			
226155_at	FAM160B1	0.858650833	0			
231615_at	FAM164A	0.639974333	3.701646186			
233971_at	FAM166A	-1.223355833	0.137080273			
226614_s_at	FAM167A	-0.518301667	1.123589586			
213954_at	FAM169A	0.538367833	1.810509752			
242762_s_at	FAM171B	0.541995167	2.499099453			
226521_s_at	FAM175A	0.644715667	2.499099453			
227828_s_at	FAM176A	-0.5067735	2.499099453	FAM176A		
1557141_at	FAM188B /// INMT	0.628622667	3.029425085			
217896_s_at	FAM192A	-0.506375333	2.499099453	FAM192A		
223791_at	FAM27A /// FAM27B /// FAM27C	0.6170755	3.701646186			
201889_at	FAM3C	1.405462667	0.05396551			
218023_s_at	FAM53C	-0.683539167	0.137080273			
234944_s_at	FAM54A	-0.567979667	2.499099453			
217540_at	FAM55C	1.021577833	0.796301331			
235030_at	FAM55C	0.869890667	0.550502435			
243011_at	FAM55C	0.901166	0.230227638			
243606_at	FAM55C	0.700376667	3.029425085			
223038_s_at	FAM60A /// LOC728115	0.599896667	1.123589586			
216044_x_at	FAM69A	-0.691249667	0.137080273			
222665_at	FAM82B	-0.523494667	2.499099453			
1563900_at	FAM83B	1.350681833	0			
225687_at	FAM83D	-0.792328333	0.230227638			
226448_at	FAM89A	0.634665	2.499099453			
203420_at	FAM8A1	0.671458333	1.455154512			
203564_at	FANCG	-0.7518485	1.123589586			
213007_at	FANCI	-0.5225415	2.499099453			

Probe Set ID	Gene Symbol	miR-128-miRNC	q value (%)	miR-128 tgts_M	miR-128 tgts_TS	miR-128 tgts_PT
203504_s_at	ABCA1	-1.2890325	0	ABCA1	ABCA1	ABCA1
209955_s_at	FAP	0.554273333	0.230227638			
224865_at	FAR1	-0.7257585	0.393887585	FAR1		FAR1
227996_at	FARP1	0.605374333	1.810509752	FARP1		
239246_at	FARP1	0.611992667	0.137080273	FARP1		
212218_s_at	FASN	-0.758701	2.499099453			
1555480_a_at	FBLIM1	0.688801167	2.499099453	FBLIM1		
225258_at	FBLIM1	0.772166833	0.05396551	FBLIM1		
229247_at	FBLN7	0.637008	0			
1553798_a_at	FBXL13	1.058953333	0.05396551			
242034_at	FBXL17	0.8820765	1.810509752			
212229_s_at	FBXO21	-0.667261167	3.029425085	FBXO21	FBXO21	
225803_at	FBXO32	1.088443667	0			
241763_s_at	FBXO32	0.9184085	0.393887585			
225099_at	FBXO45	0.512836667	0.550502435			
242294_at	FBXO45	1.262634833	0			
209630_s_at	FBXW2	-0.676158333	1.123589586	FBXW2		
1555153_s_at	FCHO2	0.661533333	0.393887585			
1554360_at	FCHSD2	0.648006333	0.550502435			
204767_s_at	FEN1	-0.916958333	0.137080273			
204768_s_at	FEN1	-1.21914	0			
206412_at	FER	0.746803667	0			
209210_s_at	FERMT2	0.500631667	1.810509752			
214212_x_at	FERMT2	0.698858667	0.796301331			
230559_x_at	FGD4	1.314932	0			
242445_at	FGD4	0.58947	3.029425085			
1552721_a_at	FGF1	-0.691226833	1.455154512	FGF1		
205117_at	FGF1	-0.9124515	0.230227638	FGF1		
208240_s_at	FGF1	-0.521666	0.393887585	FGF1		
207501_s_at	FGF12	0.945248833	0			
205014_at	FGFBP1	-0.627925833	0			
1556282_at	FGFR1OP2	1.102928333	0			
243619_at	FGFR1OP2	0.945357667	1.455154512			
206492_at	FHIT	1.102788	1.123589586			
218980_at	FHOD3	0.592187167	1.123589586			
219249_s_at	FKBP10	0.9156675	0.393887585			
219117_s_at	FKBP11	0.550765333	0.230227638			
219390_at	FKBP14	0.898667167	0.137080273			
235311_at	FKBP14	0.815646167	0.796301331			
1568717_a_at	FKBP15	0.654986167	0.796301331			
200894_s_at	FKBP4	0.760743333	1.123589586			
200895_s_at	FKBP4	0.904828	0.393887585			
224856_at	FKBP5	0.742598833	0.230227638			
224002_s_at	FKBP7	1.668450333	0			
212169_at	FKBP9	-1.402643	0	FKBP9		
235291_s_at	FLJ32255	1.108581	0			
235292_at	FLJ32255	0.604698	3.701646186			
230047_at	FLJ32810	0.707257	3.029425085			
239421_at	FLJ35776	0.706133	2.499099453			
1559614_at	FLJ38773	-0.538285	3.029425085			
221944_at	FLJ42627	0.8218485	0.393887585			

Probe Set ID	Gene Symbol	miR-128-miRNC	q value (%)	miR-128 tgts_M	miR-128 tgts_TS	miR-128 tgts_PT
203504_s_at	ABCA1	-1.2890325	0	ABCA1	ABCA1	ABCA1
226419_s_at	FLJ44342	0.561832167	3.029425085			
1565786_x_at	FLJ45482	-0.638126167	1.810509752			
213746_s_at	FLNA	-0.857509333	1.810509752			
214752_x_at	FLNA	-1.060998	2.499099453			
240259_at	FLRT2	-0.940517833	0.05396551		FLRT2	FLRT2
1570156_s_at	FMN1	0.610484167	3.029425085			
1569257_at	FMNL1	0.925775	1.123589586			
232249_at	FMNL3	0.972789	0			
1558199_at	FN1	0.748224	0.796301331	FN1		
202304_at	FNDC3A	0.505753333	3.701646186	FNDC3A		
238961_s_at	FNDC3A	0.592138333	0.05396551	FNDC3A		
241611_s_at	FNDC3A	0.679973333	0	FNDC3A		
218618_s_at	FNDC3B	0.540646667	2.499099453			
222692_s_at	FNDC3B	0.908008333	0.393887585			
222693_at	FNDC3B	0.806825	1.123589586			
225922_at	FNIP2	0.700633667	1.123589586			
225924_at	FNIP2	1.015592667	0			
226460_at	FNIP2	0.922404667	0.137080273			
211074_at	FOLR1	-0.895653333	0.796301331			
213972_at	FOXD1	-0.638863333	1.810509752			
206377_at	FOXF2	0.839572333	0.230227638			
217310_s_at	FOXJ3	0.649958667	0.550502435			
202580_x_at	FOXM1	-0.671247167	3.029425085			
224889_at	FOXO3	0.574267333	1.455154512			
231548_at	FOXO3	0.568736333	1.455154512			
224837_at	FOXP1	0.527543	0.230227638			
224838_at	FOXP1	0.506790333	1.123589586			
209864_at	FRAT2	0.818052	1.455154512			
213056_at	FRMD4B	0.567706333	3.701646186			
1569470_a_at	FRMD5	0.722294	0			
238486_at	FRS2	0.689439333	1.123589586			
212548_s_at	FRYL	-0.8388505	1.455154512	FRYL	FRYL	
214211_at	FTH1	-0.534325	0.796301331			
205324_s_at	FTSJ1	-0.525879167	0			
223042_s_at	FUNDC2	0.6225445	0.550502435			
1565717_s_at	FUS /// NR1H3	-0.567770833	3.029425085			
206109_at	FUT1	2.0536745	3.029425085			
239020_at	FUT10	0.509116667	1.455154512			
238551_at	FUT11	0.76352	0	FUT11		
209892_at	FUT4	0.739183167	3.701646186			
209893_s_at	FUT4	0.674763833	3.701646186			
1554930_a_at	FUT8	0.543743333	0.796301331	FUT8		
203988_s_at	FUT8	0.510815333	3.029425085	FUT8		
1555523_a_at	FYCO1	0.548814833	3.029425085			
218204_s_at	FYCO1	0.929706333	0.137080273			
227405_s_at	FZD8	0.948250167	1.123589586			
223254_s_at	G2E3	0.529837	2.499099453			
207112_s_at	GAB1	-0.942176667	0.05396551			
225998_at	GAB1	-0.534278667	3.029425085			

Probe Set ID	Gene Symbol	miR-128-miRNC	q value (%)	miR-128 tgts_M	miR-128 tgts_TS	miR-128 tgts_PT
203504_s_at	ABCA1	-1.2890325	0	ABCA1	ABCA1	ABCA1
203146_s_at	GABBR1	0.619830667	1.123589586	GABBR1		
238569_at	GABBR1	1.328126167	0	GABBR1		
205219_s_at	GALK2	0.598981667	1.123589586			
207357_s_at	GALNT10	0.6230045	1.455154512	GALNT10		
212256_at	GALNT10	0.638681667	1.123589586	GALNT10		
229555_at	GALNT5	0.849871167	3.701646186	GALNT5		
219956_at	GALNT6	1.11698	0			
228303_at	GALNT6	1.034632333	2.499099453			
220124_at	GAN	0.988345	1.455154512			
216990_at	GART	0.500018333	1.810509752			
230097_at	GART	0.984167333	0.137080273			
244822_at	GART	0.972771833	0			
1598_g_at	GAS6	-0.856268	0.393887585			
214711_at	GATC	0.760762667	0.550502435			
225430_at	GATC	0.526358333	0.230227638			
224627_at	GBA2	0.902226	0.393887585			
202269_x_at	GBP1	1.144951167	0			
231577_s_at	GBP1	1.436445667	0			
202748_at	GBP2	0.791088667	0			
203765_at	GCA	1.0933865	0.137080273	GCA		
211020_at	GCNT2	-0.547862667	1.123589586	GCNT2	GCNT2	
230788_at	GCNT2	-0.538286333	3.029425085	GCNT2	GCNT2	
226271_at	GDAP1	-0.508718	2.499099453	GDAP1		
201864_at	GDI1	0.557404333	1.455154512			
1555606_a_at	GDPD1	0.989425833	0.550502435			
219722_s_at	GDPD3	0.7409955	0.230227638			
225712_at	GEMIN5	0.731786333	0.796301331			
225158_at	GFM1	-0.686365833	0.796301331	GFM1		
205100_at	GFPT2	-1.125707667	0		GFPT2	GFPT2
232024_at	GIMAP2	1.804540167	0.05396551			
1554345_a_at	GIN1	0.638484833	1.455154512			
206102_at	GINS1	-0.9834965	1.455154512			
229311_at	GKAP1	0.851190167	1.123589586			
229312_s_at	GKAP1	0.875646667	0.796301331			
213552_at	GLCE	1.093316833	0			
226136_at	GLIPR1	-0.583114667	0.550502435			
230258_at	GLIS3	1.256774833	0			
221932_s_at	GLRX5	-0.8668455	0.550502435	GLRX5		
203158_s_at	GLS	1.189056333	0.137080273			
221447_s_at	GLT8D2	0.56287	1.123589586			
227070_at	GLT8D2	0.818315333	0.550502435			
219267_at	GLTP	-0.845503333	0.550502435	GLTP	GLTP	GLTP
226177_at	GLTP	-0.9609485	0	GLTP	GLTP	GLTP
218506_x_at	GLYR1	0.617311167	3.701646186			
221628_s_at	GLYR1	0.860622833	0.230227638			
234488_s_at	GMCL1 /// GMCL1L	0.611697833	3.029425085			
220938_s_at	GMEB1	0.561123833	3.029425085			
224681_at	GNA12	-0.663404167	3.029425085	GNA12		
224861_at	GNAQ	0.6131945	3.029425085			

Probe Set ID	Gene Symbol	miR-128-miRNC	q value (%)	miR-128 tgts_M	miR-128 tgts_TS	miR-128 tgts_PT
203504_s_at	ABCA1	-1.2890325	0	ABCA1	ABCA1	ABCA1
212294_at	GNG12	-0.572563333	0.137080273		GNG12	GNG12
222834_s_at	GNG12	-0.503745	1.123589586		GNG12	GNG12
238487_at	GNL1	-0.751193333	2.499099453			
239068_at	GNL1	-0.796985833	0.230227638			
1553067_a_at	GNRHR2	-0.511494833	2.499099453			
226949_at	GOLGA3	0.514736167	0.796301331			
208797_s_at	GOLGA8A	0.640476667	1.123589586	GOLGA8A		
213650_at	GOLGA8A /// GOLGA8B	0.537438333	3.029425085			
213020_at	GOSR1	0.8339595	0.796301331	GOSR1		
200708_at	GOT2	-0.615498333	0.393887585	GOT2		
225424_at	GPAM	-0.574378333	1.455154512	GPAM	GPAM	GPAM
236026_at	GPATCH2	-0.548504833	0.796301331	GPATCH2		
222452_s_at	GPBP1L1	0.586271667	0.230227638			
212510_at	GPD1L	0.533826667	0.550502435			
211613_s_at	GPD2	-0.785762	0.796301331	GPD2	GPD2	
225447_at	GPD2	-0.651808333	0	GPD2	GPD2	
201141_at	GPNMB	-1.255051667	0	GPNMB		
211977_at	GPR107	0.624134833	0.796301331			
221814_at	GPR124	1.2945825	0.393887585			
204137_at	GPR137B	0.9410395	1.123589586			
230369_at	GPR161	1.030728	0	GPR161		
212070_at	GPR56	1.307611667	0			
220993_s_at	GPR63	0.576157	2.499099453			
203108_at	GPRC5A	-0.776711667	0			
201348_at	GPX3	0.815852833	1.123589586			
206204_at	GRB14	1.3526055	0			
218468_s_at	GREM1	-1.6777955	0	GREM1	GREM1	GREM1
218469_at	GREM1	-1.675781333	0	GREM1	GREM1	GREM1
229883_at	GRIN2D	-1.226445	0.550502435		GRIN2D	
238427_at	GRPEL2	0.507318333	1.123589586			
219233_s_at	GSDMB	0.685728667	1.810509752			
225276_at	GSPT1	-0.684118833	0.05396551			GSPT1
243325_at	GSTK1	1.338129833	0			
204149_s_at	GSTM4	2.044851667	0			
210912_x_at	GSTM4	1.672022	0			
200824_at	GSTP1	-0.795384167	3.701646186			
219770_at	GTDC1	1.140108	0			
238585_at	GTDC1	1.112458333	0			
202678_at	GTF2A2	-0.73523	0.796301331	GTF2A2		GTF2A2
205930_at	GTF2E1	0.7167185	0.393887585			
221540_x_at	GTF2H2 /// GTF2H2B /// GTF2H2C /// GTF2H2D	-0.505755333	3.701646186			
215470_at	GTF2H2B	0.5689435	1.810509752	GTF2H2B		
215569_at	GTF2IRD2 /// GTF2IRD2B	0.828984	2.499099453			
204318_s_at	GTSE1	-0.6601145	1.455154512			

Probe Set ID	Gene Symbol	miR-128-miRNC	q value (%)	miR-128 tgts_M	miR-128 tgts_TS	miR-128 tgts_PT
203504_s_at	ABCA1	-1.2890325	0	ABCA1	ABCA1	ABCA1
215915_at	GULP1	-0.782919667	0.230227638			
1555568_at	GUSBL1	0.549692833	0.796301331			
212205_at	H2AFV	-1.381255167	0			
212206_s_at	H2AFV	-0.7695525	0.05396551			
205436_s_at	H2AFX	-1.504254333	0.05396551			
213344_s_at	H2AFX	-1.3971905	0			
214500_at	H2AFY	-0.857136667	0.550502435	H2AFY		
214501_s_at	H2AFY	-0.588381667	1.123589586	H2AFY		
211998_at	H3F3B	-0.9749575	0.137080273	H3F3B	H3F3B	H3F3B
209252_at	HARS2	-0.6125955	1.810509752			
222685_at	HAUS6	-1.077225	0			
1555673_at	hCG_1749898	1.211810667	0.05396551			
242141_at	HDAC2	0.803699333	1.455154512			
206846_s_at	HDAC6	0.686516	3.029425085			
1552758_at	HDAC9	0.585769	0.393887585	HDAC9		
1552760_at	HDAC9	1.251275	0	HDAC9		
234393_at	HDAC9	1.072839833	0	HDAC9		
222916_s_at	HDLBP	-0.9167365	1.455154512			
225012_at	HDLBP	-0.775575833	0			
1554478_a_at	HEATR3	0.951359833	1.810509752			
232498_at	HEATR7A	0.845603167	1.455154512			
203430_at	HEBP2	0.530570667	1.810509752			
218603_at	HECA	0.514885333	1.455154512			
232080_at	HECW2	0.966497833	0.137080273			
213069_at	HEG1	-0.705301667	0		HEG1	
1552787_at	HELB	1.3801945	0	HELB		
1552788_a_at	HELB	1.0877875	0	HELB		
220085_at	HELLS	-0.5625335	0.796301331			
223556_at	HELLS	-1.023520667	0.137080273			
227349_at	HELLS	-1.358494	0			
228736_at	HELQ	1.2035745	0			
1553402_a_at	HFE	0.811214167	1.455154512	HFE		
220387_s_at	HHLA3	1.567653333	0			
234665_x_at	HHLA3	0.711887	3.701646186			
234703_at	HHLA3	1.411044	1.123589586			
223073_at	HIATL1	-0.587603333	3.701646186			
226648_at	HIF1AN	0.668576667	1.455154512			
1555960_at	HINT1	0.5679615	3.701646186			
205425_at	HIP1	1.386643667	0	HIP1	HIP1	
1552516_a_at	HIPK1	1.370240333	0			
212291_at	HIPK1	1.589518667	0			
212293_at	HIPK1	1.228114833	0			
204578_at	HISPPD2A	0.7036855	0.550502435			
214534_at	HIST1H1B	-1.048957333	0			
208553_at	HIST1H1E	-1.320174833	0.796301331			
208569_at	HIST1H2AB	-0.7483065	1.123589586			
208583_x_at	HIST1H2AJ	-1.0089545	0.796301331			
222067_x_at	HIST1H2BD	-0.606986167	3.029425085			
208527_x_at	HIST1H2BE	-0.632988333	2.499099453			

Probe Set ID	Gene Symbol	miR-128-miRNC	q value (%)	miR-128 tgts_M	miR-128 tgts_TS	miR-128 tgts_PT
203504_s_at	ABCA1	-1.2890325	0	ABCA1	ABCA1	ABCA1
208490_x_at	HIST1H2BF	-0.893058333	0.550502435			
215779_s_at	HIST1H2BG	-0.7014015	3.029425085			
208546_x_at	HIST1H2BH	-0.947511667	0.393887585			
208523_x_at	HIST1H2BI	-0.756486	2.499099453			
208576_s_at	HIST1H3B	-1.6626765	0			
208577_at	HIST1H3C	-1.153892333	0			
206951_at	HIST1H4E	-0.550901667	0.137080273			
218279_s_at	HIST2H2AA3	-1.357884167	0.05396551			
214290_s_at	HIST2H2AA3 /// HIST2H2AA4	-1.337889	0.550502435			
218280_x_at	HIST2H2AA3 /// HIST2H2AA4	-1.0537915	0.550502435			
218726_at	HJURP	-1.0162495	0.230227638			
215313_x_at	HLA-A	0.539791667	2.499099453	HLA-A		
209140_x_at	HLA-B	0.544056667	2.499099453	HLA-B		
208812_x_at	HLA-C	0.666533333	1.123589586			
214459_x_at	HLA-C	0.609155	2.499099453			
1558561_at	HM13	0.776145333	1.455154512			
219269_at	HMBOX1	0.561059667	1.455154512	HMBOX1		
210719_s_at	HMG20B	-1.004254167	3.701646186			
1567223_at	HMGA2	-0.700883333	1.455154512	HMGA2		
1567224_at	HMGA2	-0.71614	1.810509752	HMGA2		
224731_at	HMGB1	-0.659675	1.455154512			
203744_at	HMGB3	-0.63104	0.393887585	HMGB3	HMGB3	HMGB3
225601_at	HMGB3	-0.608409333	3.029425085	HMGB3	HMGB3	HMGB3
208668_x_at	HMGN2	-0.803446667	0.550502435			
209377_s_at	HMGN3	-0.5778445	3.029425085	HMGN3		
207456_at	HNF4G	1.375739333	0		HNF4G	
222040_at	HNRNPA1	-0.587354	0.796301331	HNRNPA1		
200072_s_at	HNRNPM	-0.521988333	3.701646186			
209068_at	HNRPDL	-0.572977	1.455154512			
225386_s_at	HNRPLL	1.05398	0	HNRPLL		
226651_at	HOMER1	0.9924535	1.123589586	HOMER1		
226395_at	HOOK3	0.753454	1.455154512			
214639_s_at	HOXA1	0.734355	3.029425085			
205453_at	HOXB2	-0.574344	2.499099453			
1554251_at	HP1BP3	-0.7042245	1.810509752	HP1BP3		
224591_at	HP1BP3	-0.521819	0.550502435	HP1BP3		
224592_x_at	HP1BP3	-0.522267333	3.029425085	HP1BP3		
219403_s_at	HPSE	2.261590667	0			
205579_at	HRH1	1.071519167	0.05396551			
205580_s_at	HRH1	0.706685333	1.810509752			
205404_at	HSD11B1	0.714428667	0			
204818_at	HSD17B2	0.615541667	1.455154512			
201413_at	HSD17B4	0.511663333	0.796301331			
214434_at	HSPA12A	1.047420833	0			

Probe Set ID	Gene Symbol	miR-128-miRNC	q value (%)	miR-128 tgts_M	miR-128 tgts_TS	miR-128 tgts_PT
203504_s_at	ABCA1	-1.2890325	0	ABCA1	ABCA1	ABCA1
227650_at	HSPA14	-0.535013667	1.123589586	HSPA14		
200799_at	HSPA1A	0.912225833	0.550502435			
201841_s_at	HSPB1	-0.901319667	3.029425085			
219998_at	HSPC159	0.527871167	0.550502435			
226188_at	HSPC159	1.208141333	0.05396551			
236281_x_at	HTR7	1.420861833	0			
204883_s_at	HUS1	0.718440667	0.796301331	HUS1		
202637_s_at	ICAM1	1.12393	0			
202638_s_at	ICAM1	1.2194745	0			
215485_s_at	ICAM1	1.187157333	0			
213620_s_at	ICAM2	1.449273	0			
212221_x_at	IDS	0.729525833	0.796301331	IDS		
212223_at	IDS	0.594967833	3.701646186	IDS		
36030_at	IFFO1	1.231476333	0			
225615_at	IFFO2	1.078455167	0	IFFO2		
214453_s_at	IFI44	1.190017333	0			
219209_at	IFIH1	1.2112655	0			
201315_x_at	IFITM2	-0.552058333	3.029425085			
204191_at	IFNAR1	0.711883167	3.029425085			
202727_s_at	IFNGR1	0.761679333	0.550502435			
211676_s_at	IFNGR1	0.506792	1.455154512			
201642_at	IFNGR2	1.088279667	0			
219174_at	IFT74	0.574445333	2.499099453			
61732_r_at	IFT74	0.5241475	1.810509752			
204703_at	IFT88	0.847187	0	IFT88		
1563098_at	IGFN1	1.072268333	0.796301331			
202491_s_at	IKBKAP	0.51449	1.810509752	IKBKAP		
209341_s_at	IKBKB	0.5322755	0.550502435	IKBKB		
217371_s_at	IL15	1.52344	0.796301331			
207375_s_at	IL15RA	0.826986167	1.123589586			
227997_at	IL17RD	0.980954167	3.701646186			
205067_at	IL1B	0.707715	0.05396551			
39402_at	IL1B	0.762493333	0			
205926_at	IL27RA	1.2808145	0			
1553032_at	IL31RA	0.9586405	0.550502435			
1555431_a_at	IL31RA	0.559295667	0.550502435			
243541_at	IL31RA	1.04411	0			
205945_at	IL6R	0.734516	0.550502435			
205798_at	IL7R	-0.641750667	1.810509752			
211506_s_at	IL8	-0.533047333	0.796301331	IL8		
214493_s_at	INADL	0.9581035	0.230227638			
205070_at	ING3	0.549569833	1.810509752	ING3		
228287_at	ING5	1.1775015	0		ING5	ING5
204926_at	INHBA	0.691301667	1.123589586			
210511_s_at	INHBA	0.543061667	1.123589586			
210587_at	INHBE	1.5613865	1.455154512			
231152_at	INO80D	0.660243833	1.810509752			
204552_at	INPP4A	0.888526833	0.230227638			
204553_x_at	INPP4A	0.810150167	0.137080273			
208363_s_at	INPP4A	0.672058	0.550502435			

Probe Set ID	Gene Symbol	miR-128-miRNC	q value (%)	miR-128 tgts_M	miR-128 tgts_TS	miR-128 tgts_PT
203504_s_at	ABCA1	-1.2890325	0	ABCA1	ABCA1	ABCA1
237056_at	INSC	1.547815333	0.393887585			
201626_at	INSIG1	0.530025333	3.029425085			
1553670_at	INTS4	0.600876833	2.499099453			
225169_at	INTS4	0.572596333	1.123589586			
222250_s_at	INTS7	0.625879167	2.499099453			
221185_s_at	IQCG	1.069745	0			
1569061_at	IQGAP3	-1.1421335	0	IQGAP3		
1569062_s_at	IQGAP3	-0.939022667	0.550502435	IQGAP3		
229538_s_at	IQGAP3	-0.592498833	0.393887585	IQGAP3		
241939_at	IQGAP3	-1.179525	0.796301331	IQGAP3		
238599_at	IRAK1BP1	0.6510935	3.701646186	IRAK1BP1		
231779_at	IRAK2	1.8337835	0			
203275_at	IRF2	0.512943333	0.796301331			
203882_at	IRF9	0.638458	1.455154512			
1555833_a_at	IRGQ	1.045915167	1.123589586			
239177_at	IRGQ	1.2818645	0.796301331			
64488_at	IRGQ	1.068586667	1.123589586			
208114_s_at	ISG20L2	0.992689667	0.796301331			
205032_at	ITGA2	0.999658333	0		ITGA2	
227314_at	ITGA2	0.880578167	1.810509752		ITGA2	
204626_s_at	ITGB3	1.445504	0			
204627_s_at	ITGB3	1.453644333	0			
204628_s_at	ITGB3	1.3837095	0			
216261_at	ITGB3	1.250216333	0.550502435			
205176_s_at	ITGB3BP	-0.546495833	0.393887585			
201124_at	ITGB5	-0.681106833	0.137080273			
205816_at	ITGB8	-2.003437167	1.810509752			
211323_s_at	ITPR1	1.063213333	0	ITPR1		
216944_s_at	ITPR1	0.679302833	1.123589586	ITPR1		
1568619_s_at	ITPRIPL2	0.662723	3.029425085	ITPRIPL2		
240941_at	ITSN2	1.276366333	0	ITSN2	ITSN2	ITSN2
201362_at	IVNS1ABP	1.316976833	0	IVNS1ABP		
201363_s_at	IVNS1ABP	1.350155667	0	IVNS1ABP		
206245_s_at	IVNS1ABP	0.721803333	0.05396551	IVNS1ABP		
244235_at	IVNS1ABP	0.825426667	2.499099453	IVNS1ABP		
209099_x_at	JAG1	1.089264833	3.029425085	JAG1	JAG1	
216268_s_at	JAG1	1.137125333	0	JAG1	JAG1	
219213_at	JAM2	1.289621333	0			
203298_s_at	JARID2	0.796438333	1.123589586			
1553533_at	JPH1	1.023479833	0			
1553764_a_at	JUB	-0.813552	0.05396551			
225806_at	JUB	-0.699696167	1.455154512			
213005_s_at	KANK1	1.069328667	0.550502435			
228777_at	KBTBD3	0.522391167	3.701646186			
222471_s_at	KCMF1	0.521457	3.029425085		KCMF1	
208213_s_at	KCNAB1	-0.872734167	0.137080273	KCNAB1	KCNAB1	KCNAB1
210078_s_at	KCNAB1	-0.630348333	3.029425085	KCNAB1	KCNAB1	KCNAB1
210079_x_at	KCNAB1	-0.991321	0.137080273	KCNAB1	KCNAB1	KCNAB1
210471_s_at	KCNAB1	-0.84353	0.230227638	KCNAB1	KCNAB1	KCNAB1
205952_at	KCNK3	0.925994	0.550502435			

Probe Set ID	Gene Symbol	miR-128-miRNC	q value (%)	miR-128 tgts_M	miR-128 tgts_TS	miR-128 tgts_PT
203504_s_at	ABCA1	-1.2890325	0	ABCA1	ABCA1	ABCA1
223658_at	KCNK6	1.601120167	0.05396551	KCNK6		
243435_at	KCNQ1OT1	-0.789268667	1.123589586			
223891_at	KCNQ5	1.0421145	0.05396551			
244623_at	KCNQ5	1.017806667	1.810509752			
239098_at	KCNRG	-0.989589	0.230227638			
240288_at	KCNRG	-0.717764	0.230227638			
205968_at	KCNS3	0.7775285	3.701646186			
223208_at	KCTD10	-0.566851667	3.029425085			
226518_at	KCTD10	-0.698086667	0.137080273			
212192_at	KCTD12	1.054693333	0.393887585			
219545_at	KCTD14	0.8678185	0			
58916_at	KCTD14	0.802103833	0.230227638			
222664_at	KCTD15	0.540151333	1.455154512			
222668_at	KCTD15	0.609326667	1.123589586			
229873_at	KCTD21	0.915927833	0.230227638			
238001_at	KCTD6	0.511388333	3.701646186			
208988_at	KDM2A	0.798761667	1.455154512	KDM2A		
208989_s_at	KDM2A	0.5916935	0.796301331	KDM2A		
201549_x_at	KDM5B	0.528897667	1.123589586			
203144_s_at	KIAA0040	3.026345167	2.499099453			
238659_at	KIAA0141	0.752939167	3.029425085			
212057_at	KIAA0182	0.811820333	1.455154512			
1560138_at	KIAA0226	0.782699	0.137080273			
213208_at	KIAA0240	0.666228333	1.455154512			
38892_at	KIAA0240	0.887378833	2.499099453			
222468_at	KIAA0319L	-0.6328275	0.393887585			
212355_at	KIAA0323	0.810006667	0.137080273			
212356_at	KIAA0323	0.767367	0.796301331			
212427_at	KIAA0368	-0.589078833	1.123589586			
37232_at	KIAA0586	-0.574714833	3.701646186			
203955_at	KIAA0649	0.614826	3.029425085			
212311_at	KIAA0746	1.246710167	0.05396551			
212314_at	KIAA0746	0.9922805	0.550502435			
230232_at	KIAA0746	1.302487667	0.230227638			
215268_at	KIAA0754	-0.780388333	3.029425085	KIAA0754		
215081_at	KIAA1024	1.191755667	3.029425085			
223161_at	KIAA1147	0.914650667	0.137080273			
223162_s_at	KIAA1147	0.867486667	0.230227638			
231856_at	KIAA1244	1.232423833	0.393887585			
243589_at	KIAA1267 /// LOC100294337	-0.767028333	0.796301331			
235301_at	KIAA1324L	0.629044167	0.550502435			
226222_at	KIAA1432	-0.598940333	1.123589586	KIAA1432		
213232_at	KIAA1467	0.813852	0.393887585	KIAA1467		
213234_at	KIAA1467	1.226027833	0	KIAA1467		
223575_at	KIAA1549	0.604099333	0.550502435		KIAA1549	
236078_at	KIAA1609	-0.706302667	0.796301331			
231956_at	KIAA1618	0.641956667	1.455154512			
232031_s_at	KIAA1632	0.548669833	2.499099453			

Probe Set ID	Gene Symbol	miR-128-miRNC	q value (%)	miR-128 tgts_M	miR-128 tgts_TS	miR-128 tgts_PT
203504_s_at	ABCA1	-1.2890325	0	ABCA1	ABCA1	ABCA1
236108_at	KIAA1632	0.523479833	1.810509752			
1559601_at	KIAA2018	0.536509667	3.029425085		KIAA218	
204444_at	KIF11	-0.718696667	0.393887585	KIF11		
202962_at	KIF13B	0.820710833	0.05396551	KIF13B		
206364_at	KIF14	-0.625691667	1.810509752			
236641_at	KIF14	-0.682301	0.550502435			
219306_at	KIF15	-1.210071	0			
221258_s_at	KIF18A	-0.651346667	0.393887585			
225878_at	KIF1B	0.855027333	0.796301331	KIF1B		
205235_s_at	KIF20B	-0.684363333	3.029425085			
204411_at	KIF21B	1.347847167	1.455154512	KIF21B		
232514_at	KIF27	0.881132833	1.810509752			
209408_at	KIF2C	-0.795688167	0.796301331			
203130_s_at	KIF5C	1.796927333	0			
209680_s_at	KIFC1	-0.5993035	3.701646186			
205664_at	KIN	0.5610595	1.123589586			
208467_at	KLF12	0.513375333	0.550502435			
227261_at	KLF12	0.898437833	1.810509752			
220266_s_at	KLF4	-0.508877	3.029425085	KLF4	KLF4	KLF4
1555832_s_at	KLF6	-0.554831667	1.123589586			
225732_at	KLHDC5	1.004066667	0.550502435			
225961_at	KLHDC5	0.51015	3.701646186			
226370_at	KLHL15	0.931311	1.455154512			
212882_at	KLHL18	0.822605333	0.393887585	KLHL18	KLHL18	
221986_s_at	KLHL24	0.547898333	0.393887585			
226158_at	KLHL24	0.9672205	0.393887585			
242088_at	KLHL24	0.790345	1.455154512			
228328_at	KLHL28	1.036437833	2.499099453	KLHL28		
235727_at	KLHL28	1.141157667	0	KLHL28		
220239_at	KLHL7	-0.742998833	0.550502435	KLHL7		
242648_at	KLHL8	0.893806333	1.123589586			
206316_s_at	KNTC1	-0.613475	1.455154512			
202057_at	KPNA1	0.659684833	3.029425085			
221502_at	KPNA3	-0.643513333	1.455154512			KPNA3
233122_at	KRTCAP2	1.354392333	0			
202202_s_at	LAMA4	1.073173833	0.05396551			
210990_s_at	LAMA4	0.582372333	3.701646186			
209270_at	LAMB3	0.528495	1.810509752			
202267_at	LAMC2	1.267939333	0	LAMC2		
207517_at	LAMC2	0.660938333	1.123589586	LAMC2		
217933_s_at	LAP3	0.564282333	1.123589586	LAP3		
1554679_a_at	LAPTM4B	-0.826705	0.137080273	LAPTM4B		
208029_s_at	LAPTM4B	-0.52547	0.550502435	LAPTM4B		
208767_s_at	LAPTM4B	-0.688905	0	LAPTM4B		
1555384_a_at	LARP4	0.8042	0.230227638		LARP4	
238959_at	LARP4	0.561381667	1.123589586		LARP4	
238960_s_at	LARP4	0.631667333	1.455154512		LARP4	
242019_at	LASS6	0.521409667	3.029425085			
211005_at	LAT /// SPNS1	1.023411833	3.701646186			

Probe Set ID	Gene Symbol	miR-128-miRNC	q value (%)	miR-128 tgts_M	miR-128 tgts_TS	miR-128 tgts_PT
203504_s_at	ABCA1	-1.2890325	0	ABCA1	ABCA1	ABCA1
221581_s_at	LAT2	1.604362667	0			
1570425_s_at	LATS1	0.638568	0.393887585			
1556886_a_at	LAYN	0.888875333	1.455154512			
232293_at	LCORL	-0.596288	3.029425085			
208885_at	LCP1	1.8591485	0			
202068_s_at	LDLR	-0.573075167	1.123589586	LDLR	LDLR	
211354_s_at	LEPR	0.744019167	1.123589586			
211355_x_at	LEPR	0.694816	1.455154512			
211356_x_at	LEPR	0.713678667	1.455154512			
202594_at	LEPROTL1	0.593432333	0.550502435			
202595_s_at	LEPROTL1	0.627747	0.137080273			
210393_at	LGR5	-1.198870667	0.796301331			
231976_at	LINS1	0.525709	3.701646186			
225793_at	LIX1L	0.522621667	1.123589586			
235036_at	LIX1L	0.916808667	0.05396551			
244881_at	LMLN	0.519900667	1.455154512			
203411_s_at	LMNA	-0.594091	2.499099453			
203276_at	LMNB1	-0.869214333	0	LMNB1		
216952_s_at	LMNB2	-0.971531667	1.123589586			
204249_s_at	LMO2	0.722241	0.796301331	LMO2		
241922_at	LMO4	-0.645178667	2.499099453			
228647_at	LOC100049716	0.611987833	0.550502435			
240407_at	LOC100126784	0.629013	1.123589586			
244074_at	LOC100129104	0.677263333	2.499099453			
244489_at	LOC100129268	0.6649825	1.123589586			
224256_at	LOC100129449	-0.902224333	0.550502435			
1556555_at	LOC100129461	1.171106333	0			
239029_at	LOC100129701	0.5488055	2.499099453			LOC100129701
234757_at	LOC100129884	-0.780134333	3.701646186			
236560_at	LOC100129884	-0.553480333	0.550502435			
242255_at	LOC100130837	0.887758333	0			
244659_at	LOC100131015	-0.611539833	3.029425085			
228712_at	LOC100132369	-0.890289667	0.796301331			
1558527_at	LOC100132707	0.901028167	0			
1558046_x_at	LOC100132864 /// LOC100293492 ///	0.541234333	3.701646186			

Probe Set ID	Gene Symbol	miR-128-miRNC	q value (%)	miR-128 tgts_M	miR-128 tgts_TS	miR-128 tgts_PT
203504_s_at	ABCA1	-1.2890325	0	ABCA1	ABCA1	ABCA1
	LOC389906 /// LOC441528 /// LOC729162					
1565666_s_at	LOC100133761 /// MUC6	-1.8353475	0.393887585			
1557783_at	LOC100133991	0.859474167	3.029425085			
235167_at	LOC100190986	-0.854171667	0.550502435			
213605_s_at	LOC100272216 /// LOC100292101	-0.6309315	0.550502435			
1569315_s_at	LOC100272228	1.480399667	0	LOC100272228		
228231_at	LOC100287081	0.759667333	0.550502435			
217480_x_at	LOC100287723 /// LOC642424 /// LOC642838	-1.758837167	0			
244766_at	LOC100288704 /// LOC595101 /// LOC641298 /// SMG1	-0.622438167	3.701646186			LOC100288704 /// LOC595101 /// LOC641298 /// SMG1
230746_s_at	LOC100288985	1.599512833	0			
227287_at	LOC100290973	-1.382144833	0			
227136_s_at	LOC100292024	0.939219	0.393887585			
221202_at	LOC100293871	0.876872333	1.123589586			
1558256_at	LOC148189	0.873184833	0.230227638			
232281_at	LOC148189	0.8092765	2.499099453			
1559433_at	LOC149773	1.311203	0.393887585			
225657_at	LOC152217	-0.657092	1.810509752			
1562048_at	LOC152225	0.885700667	0			
232645_at	LOC153684	0.971307833	3.701646186			
1559129_a_at	LOC158257	0.773181333	1.455154512			
232034_at	LOC203274	0.524926167	1.810509752			
1564651_at	LOC221710	0.770695833	0.550502435			
227124_at	LOC221710	0.677479	1.455154512			
235707_at	LOC221710	0.579384833	1.455154512			
1557131_at	LOC254100	1.517744333	1.455154512			
226793_at	LOC283267	0.678483333	0.393887585			

Probe Set ID	Gene Symbol	miR-128-miRNC	q value (%)	miR-128 tgts_M	miR-128 tgts_TS	miR-128 tgts_PT
203504_s_at	ABCA1	-1.2890325	0	ABCA1	ABCA1	ABCA1
229528_at	LOC283378	0.709730833	0.796301331			
233737_s_at	LOC284561	0.6370095	0.230227638			
236321_at	LOC285550	-0.739969	1.455154512			
232504_at	LOC285628	1.781827833	0			
222196_at	LOC286434	1.074320833	0			
239762_at	LOC286437	0.814957333	0			
228160_at	LOC339290	-0.624622833	1.123589586			
1563589_at	LOC340184	1.440981	0			
235606_at	LOC344595	0.6998985	0.550502435			
1558982_at	LOC375010	-0.575771333	2.499099453			
220602_s_at	LOC388152 /// LOC727751 /// LOC727849 /// LOC80154	0.7139085	1.123589586			
1560841_at	LOC389247	0.731755	3.029425085			
230815_at	LOC389765	0.669755667	3.701646186	LOC389765		
213556_at	LOC390940	1.264860167	0	LOC390940		
235482_at	LOC400960	0.6105405	1.455154512			
228601_at	LOC401022	0.568965333	3.029425085			
239182_at	LOC401022	0.626813833	1.123589586			
242042_s_at	LOC401022	0.518068333	2.499099453			
1570243_at	LOC440731	1.495253	0			
1556404_a_at	LOC441208	0.743135667	3.029425085			
1559957_a_at	LOC642852	0.751563167	1.123589586			
243656_at	LOC642852	0.664590833	0.550502435			
226404_at	LOC643167 /// RBM39	0.8556	0.393887585			
1557765_at	LOC643401	-0.599228333	3.701646186			
244105_at	LOC644231 /// WHAMML1 /// WHAMML2	0.959869667	0.230227638			
227976_at	LOC644538	-1.240517833	0.230227638	LOC644538		
231268_at	LOC645895 /// MYBL1	-0.925111667	1.123589586			
238715_at	LOC646014	0.565741667	0.796301331			
244268_x_at	LOC646214	0.633651333	3.029425085			
216902_s_at	LOC653390 /// LOC730092 /// RRN3	0.784669	0.05396551			
237182_at	LOC653479	1.031766667	0.05396551			
218641_at	LOC65998	0.658128	0.550502435			
228596_at	LOC728377	1.646793	0.550502435			
1558640_a_at	LOC728411	-0.872264	0.137080273			
235010_at	LOC729013	-0.694166333	1.123589586	LOC729013		
214291_at	LOC729046 /// RPL17	0.538117833	1.123589586			

Probe Set ID	Gene Symbol	miR-128-miRNC	q value (%)	miR-128 tgts_M	miR-128 tgts_TS	miR-128 tgts_PT
203504_s_at	ABCA1	-1.2890325	0	ABCA1	ABCA1	ABCA1
217544_at	LOC729806	0.883587	0.796301331			
236692_at	LOC729839	0.560832	0.796301331			
229978_at	LOC729993	-0.701168667	0.230227638			
216908_x_at	LOC730092	0.607679667	0.796301331			
213248_at	LOC730101	-1.106216167	1.810509752			
212957_s_at	LOC92249	0.6584685	1.123589586			
1569013_s_at	LOC96610	1.535053167	0			
226802_s_at	LOC96610	1.089342167	0			
233132_at	LOC96610	1.016399	0			
228808_s_at	LOXL2	-0.8993335	1.123589586	LOXL2		
220816_at	LPAR3	0.532297167	1.810509752			
231192_at	LPAR3	0.679548667	0.137080273			
231788_at	LPAR5	0.8128155	0			
226795_at	LRCH1	0.753065667	0.393887585			
235012_at	LRCH1	0.771179667	0.05396551			
211596_s_at	LRIG1	0.711855	1.455154512			
226908_at	LRIG3	1.128096167	1.123589586			
34697_at	LRP6	0.631088167	0.230227638	LRP6	LRP6	
211615_s_at	LRPPRC	-0.719891667	0			
211971_s_at	LRPPRC	-0.50962	1.455154512			
230194_at	LRPPRC	-1.082053833	1.123589586			
1553940_a_at	LRRC28	0.584885833	1.455154512	LRRC28		
227423_at	LRRC28	0.765424667	1.123589586	LRRC28		
236917_at	LRRC34	-0.846150667	0.393887585			
236918_s_at	LRRC34	-0.744229833	0.137080273			
224686_x_at	LRRC37A2	-0.598526667	1.810509752			
219338_s_at	LRRC49	1.008896167	0.230227638			
201862_s_at	LRRFIP1	0.54249	1.810509752	LRRFIP1		
218364_at	LRRFIP2	-0.649568333	0.796301331			
232704_s_at	LRRFIP2	-1.0139055	0			
202728_s_at	LTBP1	0.927014167	0.550502435	LTBP1		LTBP1
239212_at	LTV1	2.084952833	1.123589586			
1557066_at	LUC7L	1.434673333	0			
1557067_s_at	LUC7L	0.932535667	0.393887585			
223295_s_at	LUC7L	0.624503833	1.123589586			
218729_at	LXN	1.254442667	0			
206584_at	LY96	0.509990667	1.810509752			
226748_at	LYSMD2	0.911459833	0			
208633_s_at	MACF1	-0.604666667	1.123589586			
208634_s_at	MACF1	-0.739048333	0.550502435			
215222_x_at	MACF1	-0.663666667	1.123589586			
1554768_a_at	MAD2L1	-0.752199333	0.550502435			
203362_s_at	MAD2L1	-0.802265333	0.230227638			
223234_at	MAD2L2	-1.233333333	1.810509752			
36711_at	MAFF	0.7751855	0.137080273			
223313_s_at	MAGED4 /// MAGED4B	0.645225833	0.550502435			
221553_at	MAGT1	0.727702333	0.230227638			
202360_at	MAML1	0.7124435	3.029425085			
205088_at	MAMLD1	0.511758833	3.701646186			

Probe Set ID	Gene Symbol	miR-128-miRNC	q value (%)	miR-128 tgts_M	miR-128 tgts_TS	miR-128 tgts_PT
203504_s_at	ABCA1	-1.2890325	0	ABCA1	ABCA1	ABCA1
1554193_s_at	MANEA	0.50326	1.810509752			
1555227_a_at	MANEA	0.616059833	2.499099453			
208786_s_at	MAP1LC3B	-0.503882667	1.810509752			
202670_at	MAP2K1	0.561605	1.810509752			
205447_s_at	MAP3K12	0.713811333	0	MAP3K12		
206249_at	MAP3K13	1.0333965	0			
205192_at	MAP3K14	0.576311167	0.393887585			
242581_at	MAP3K15	0.939607167	3.029425085			
221695_s_at	MAP3K2	0.649919667	0.796301331			
226979_at	MAP3K2	0.50373	2.499099453			
220145_at	MAP9	0.997477833	0.05396551			
235550_at	MAP9	1.2320035	0.05396551			
210449_x_at	MAPK14	-0.754121167	1.455154512	MAPK14	MAPK14	MAPK14
211561_x_at	MAPK14	-0.868872667	1.123589586	MAPK14	MAPK14	MAPK14
210477_x_at	MAPK8	0.5589225	1.810509752			
226048_at	MAPK8	0.598381667	1.123589586			
1558839_at	MAPKBP1	0.622381667	1.123589586			
202501_at	MAPRE2	0.600953833	1.123589586	MAPRE2		
201737_s_at	MARCH6	0.774751	0.393887585			
201669_s_at	MARCKS	-0.545816667	2.499099453	MARCKS	MARCKS	MARCKS
201670_s_at	MARCKS	-0.55758	0.550502435	MARCKS	MARCKS	MARCKS
213002_at	MARCKS	-0.50317	1.123589586	MARCKS	MARCKS	MARCKS
225897_at	MARCKS	-0.7906045	0.137080273	MARCKS	MARCKS	MARCKS
200644_at	MARCKSL1	-0.9142705	1.123589586	MARCKSL1		
211082_x_at	MARK2	0.7959945	1.123589586			
243529_at	MARS2	0.905253333	0			
242260_at	MATR3	-0.628643167	3.701646186			
209332_s_at	MAX	0.941614667	1.123589586			
212064_x_at	MAZ	0.855252333	2.499099453			
228798_x_at	MAZ	0.7372105	3.701646186			
241813_at	MBD1	-0.731215333	3.029425085	MBD1		
1558111_at	MBNL1	-0.935592333	0.137080273			
235879_at	MBNL1	-0.779248	1.455154512			
226726_at	MBOAT2	0.506816833	0.796301331			
228938_at	MBP	0.756946833	1.810509752			
232092_at	MCART1	0.51176	0.550502435			
226238_at	MCEE	0.509265333	1.810509752			
220459_at	MCM3APAS	0.665529333	2.499099453			
222036_s_at	MCM4	-0.765275667	2.499099453	MCM4		
222037_at	MCM4	-1.0137155	3.029425085	MCM4		
1555465_at	MCOLN2	0.943278167	0			
217542_at	MDM2	-0.545884	0.137080273			
236814_at	MDM4	0.962945	0.550502435	MDM4		
1555300_a_at	MED12L	0.6915675	3.701646186			
215167_at	MED14	1.107279833	0	MED14	MED14	MED14
209363_s_at	MED21	0.662185	0.550502435			
242707_at	MED23	1.193036667	0.796301331			
1553993_s_at	MED25	-1.052325167	3.701646186			
222635_s_at	MED28	0.554587667	3.701646186	MED28		
219318_x_at	MED31	0.596330667	2.499099453	MED31		

Probe Set ID	Gene Symbol	miR-128-miRNC	q value (%)	miR-128 tgts_M	miR-128 tgts_TS	miR-128 tgts_PT
203504_s_at	ABCA1	-1.2890325	0	ABCA1	ABCA1	ABCA1
236241_at	MED31	0.767021333	1.810509752	MED31		
235473_at	MED6	0.559603	1.455154512			
212830_at	MEGF9	-0.858859833	0.137080273			
212831_at	MEGF9	-0.954309833	0.796301331			
204825_at	MELK	-0.585120833	1.123589586			
225955_at	METRNL	-0.609260833	2.499099453			
226634_at	METTL10	-0.539267333	0.393887585			
221079_s_at	METTL2A /// METTL2B	0.624188333	0.137080273			
232533_at	METTL8	-0.607281167	0.230227638			
1552312_a_at	MFAP3	1.056962333	0			
213123_at	MFAP3	1.031475333	0			
214588_s_at	MFAP3	1.372485	0			
225478_at	MFHAS1	1.3060895	0		MFHAS1	
219858_s_at	MFSD6	1.021952667	0			
213393_at	MFSD9	0.953543333	1.123589586			
213403_at	MFSD9	1.7375555	0			
231283_at	MGAT4A	1.422752333	3.701646186	MGAT4A		
228235_at	MGC16121	-0.98527	0			
1558166_at	MGC16275	-0.796555833	1.810509752			
1555874_x_at	MGC21881	-0.618591	1.455154512	MGC21881		
211718_at	MGC2889	1.0551995	1.123589586	MGC2889		
239104_at	MGC42157	0.683851	3.701646186			
1568987_at	MGC57346	0.797478	1.810509752			
214972_at	MGEA5	0.6873155	0	MGEA5		
225102_at	MGLL	-0.591045	2.499099453			
224918_x_at	MGST1	-0.758323333	0.137080273			
231736_x_at	MGST1	-0.636346667	0.137080273			
205905_s_at	MICA /// MICB	0.943032667	0			
243611_at	MICALCL	1.270103833	3.701646186			
206247_at	MICB	1.4308335	0	MICB		
221862_at	MIER2	-0.851043167	0.550502435	MIER2	MIER2	
1553336_a_at	MIER3	0.8786385	1.123589586	MIER3		MIER3
1554449_at	MIER3	0.697183833	1.455154512	MIER3		MIER3
1554450_s_at	MIER3	0.614944833	2.499099453	MIER3		MIER3
217871_s_at	MIF	-0.889451	3.701646186			
229437_at	MIR155HG	-0.5245445	1.810509752			
224917_at	MIR21	0.673340333	0.393887585			
226066_at	MITF	0.956190667	0			
212020_s_at	MKI67	-0.932407833	0.796301331			
212021_s_at	MKI67	-0.750905667	2.499099453			
212023_s_at	MKI67	-1.192289667	0			
225526_at	MKLN1	-0.506705333	2.499099453			
235067_at	MKLN1	-0.528046667	3.701646186			
209845_at	MKRN1	1.1002325	0.137080273			
200616_s_at	MLEC	0.907346667	0.137080273			
200617_at	MLEC	0.894170833	0			
204783_at	MLF1	0.945787667	1.455154512			
218883_s_at	MLF1IP	-0.966314167	0.05396551	MLF1IP		

**Probe Set ID**	**Gene Symbol**	**miR-128-miRNC**	**q value (%)**	**miR-128 tgts_M**	**miR-128 tgts_TS**	**miR-128 tgts_PT**
203504_s_at	ABCA1	-1.2890325	0	ABCA1	ABCA1	ABCA1
238025_at	MLKL	0.807381	0	MLKL		
225992_at	MLLT10	-0.506082667	0.393887585	MLLT10	MLLT1	
218211_s_at	MLPH	0.755324833	0			
202519_at	MLXIP	0.606896833	0.230227638			
225157_at	MLXIP	0.540753667	2.499099453			
236347_at	MMAA	0.8724325	1.123589586			
242702_at	MMAA	1.993090333	0.05396551			
242750_at	MMAA	1.4953535	0			
203414_at	MMD	0.8241155	0.550502435	MMD	MMD	MMD
203565_s_at	MNAT1	0.6376385	2.499099453			
214812_s_at	MOBKL1B	-0.660363333	1.123589586			
243052_at	MOBKL2C	0.8296145	1.810509752	MOBKL2C		
1557455_s_at	MOSPD1	0.528037667	2.499099453			
218853_s_at	MOSPD1	0.793055333	0.05396551			
221895_at	MOSPD2	0.807120833	1.123589586			
64883_at	MOSPD2	1.144976833	0.05396551			
213306_at	MPDZ	0.5426975	3.701646186			
1564308_a_at	MPP7	0.679773333	2.499099453			
238451_at	MPP7	1.088982167	0			
201874_at	MPZL1	0.578051667	1.810509752			
201875_s_at	MPZL1	0.619751	0.796301331			
207565_s_at	MR1	0.779708667	1.123589586			
210224_at	MR1	0.791324667	0			
219648_at	MREG	0.649733667	1.455154512			
203931_s_at	MRPL12	-1.1405845	0.05396551	MRPL12		
225315_at	MRPL21	-0.569325333	3.029425085			
218339_at	MRPL22	-0.535695	2.499099453			
220103_s_at	MRPS18C	0.630821667	1.810509752			
226256_at	MRPS22	0.636009333	2.499099453			
238555_at	MRPS31	1.337791333	0			
217942_at	MRPS35	0.582662	1.810509752			
220688_s_at	MRTO4	-0.561032833	3.029425085			
210947_s_at	MSH3	0.743662333	0			
212708_at	MSL1	0.600170833	0.550502435	MSL1		
208423_s_at	MSR1	0.6068275	1.123589586			
211887_x_at	MSR1	0.597667667	1.123589586			
227150_at	MTF1	0.910264333	0			
203433_at	MTHFS	0.830528833	0			
216303_s_at	MTMR1	0.923799167	0.393887585			
1556034_s_at	MTMR11	1.118861167	2.499099453			
205076_s_at	MTMR11	1.3872635	0			
204837_at	MTMR9	0.780712	0			
225215_s_at	MTRF1L	0.552893333	0.796301331	MTRF1L		
203036_s_at	MTSS1	0.858075	0			
203037_s_at	MTSS1	0.938178333	1.455154512			
210359_at	MTSS1	0.958435	0			
210360_s_at	MTSS1	0.761740333	0.796301331			
221290_s_at	MUM1	1.243357	0.137080273	MUM1		
206877_at	MXD1	0.744811	0.550502435			
228846_at	MXD1	1.176394	0			

Probe Set ID	Gene Symbol	miR-128-miRNC	q value (%)	miR-128 tgts_M	miR-128 tgts_TS	miR-128 tgts_PT
203504_s_at	ABCA1	-1.2890325	0	ABCA1	ABCA1	ABCA1
227326_at	MXRA7	0.650436	0.550502435			
213906_at	MYBL1	-1.020301	0	MYBL1		
212372_at	MYH10	-0.638005	0.230227638	MYH10	MYH1	
213067_at	MYH10	-0.792342833	0.230227638	MYH10	MYH1	
1564072_at	MYH16	0.524295833	3.701646186			
1563466_at	MYLK	-0.640457333	0.796301331			
224823_at	MYLK	-0.521580833	2.499099453			
1566108_at	MYNN	0.639985333	1.810509752			
215119_at	MYO16	-1.760120333	0			
219320_at	MYO19	0.761726	0			
225080_at	MYO1C	0.501136667	3.029425085			
212338_at	MYO1D	1.059617833	0	MYO1D		
201798_s_at	MYOF	-0.515585	1.123589586			
217518_at	MYOF	-1.0839095	0			
211874_s_at	MYST4	0.5443915	2.499099453			
235547_at	N4BP2L2	0.58248	1.455154512			
220311_at	N6AMT1	0.9601815	0			
228062_at	NAP1L5	-0.6363045	0.796301331	NAP1L5	NAP1L5	
228063_s_at	NAP1L5	-0.651773333	1.810509752	NAP1L5	NAP1L5	
242635_s_at	NAPEPLD	0.934349833	0	NAPEPLD	NAPEPLD	
219217_at	NARS2	0.771796833	0	NARS2		
242918_at	NASP	0.8095345	0.550502435			
224772_at	NAV1	-0.513992	0.550502435			
216632_at	NAV3	-1.017474667	0.796301331	NAV3	NAV3	NAV3
242191_at	NBPF10	0.7699295	0.05396551			
1568856_at	NBR1	1.521970667	1.455154512			
1568857_a_at	NBR1	0.989545833	0			
201383_s_at	NBR1	0.719066667	0.393887585			
201384_s_at	NBR1	0.52325	1.455154512			
227394_at	NCAM1	0.506381	2.499099453	NCAM1	NCAM1	
218662_s_at	NCAPG	-0.57513	1.123589586	NCAPG		
219588_s_at	NCAPG2	-0.713446333	0.230227638			
212949_at	NCAPH	-0.513554	0.393887585			
207738_s_at	NCKAP1	0.724320667	3.701646186	NCKAP1		
205731_s_at	NCOA2	0.70948	0.05396551			
205732_s_at	NCOA2	0.880083333	0.796301331			
203245_s_at	NCRNA00094	0.634399833	1.123589586			
220399_at	NCRNA00115	0.681572667	1.455154512			
1552258_at	NCRNA00152	-0.926714667	2.499099453			
204162_at	NDC80	-0.713508333	0.230227638			
227249_at	NDE1	-0.562352833	1.123589586			
208093_s_at	NDEL1	0.697096833	0.137080273			
230190_at	NDFIP2	0.506959333	2.499099453			
200632_s_at	NDRG1	0.764876667	0.05396551			
224368_s_at	NDRG3	0.596920333	1.810509752			
209159_s_at	NDRG4	0.661227	1.455154512			
218563_at	NDUFA3	-0.5391445	1.455154512			

Probe Set ID	Gene Symbol	miR-128-miRNC	q value (%)	miR-128 tgts_M	miR-128 tgts_TS	miR-128 tgts_PT
203504_s_at	ABCA1	-1.2890325	0	ABCA1	ABCA1	ABCA1
202001_s_at	NDUFA6	-0.82388	0			
227559_at	NDUFAF4	0.686844167	1.810509752			
223112_s_at	NDUFB10	-0.7329135	2.499099453			
203621_at	NDUFB5	0.602721667	2.499099453			
203606_at	NDUFS6	-0.608480833	2.499099453			
238320_at	NEAT1	-1.015751667	1.810509752			
213012_at	NEDD4	0.730826333	1.810509752		NEDD4	NEDD4
201840_at	NEDD8	-0.654248333	2.499099453			
202149_at	NEDD9	0.980094	1.123589586			
202150_s_at	NEDD9	0.7111215	0			
204641_at	NEK2	-1.3800265	0	NEK2	NEK2	NEK2
211080_s_at	NEK2	-0.834346667	0.796301331	NEK2	NEK2	NEK2
223158_s_at	NEK6	-0.508997833	0.796301331	NEK6	NEK6	NEK6
229877_at	NEO1	-0.598510833	1.810509752	NEO1	NEO1	NEO1
218888_s_at	NETO2	-0.714987667	1.810509752	NETO2		
231801_at	NFATC2	-1.1083065	0.550502435	NFATC2		
1567014_s_at	NFE2L2	0.596223333	1.123589586	NFE2L2		NFE2L2
1567015_at	NFE2L2	0.625163333	1.123589586	NFE2L2		NFE2L2
213032_at	NFIB	-0.542068333	0.796301331		NFIB	
213033_s_at	NFIB	-0.620654833	0.05396551		NFIB	
201502_s_at	NFKBIA	0.873017667	0			
204109_s_at	NFYA	0.54515	2.499099453			
228433_at	NFYA	0.759999667	1.123589586			
217963_s_at	NGFRAP1	-0.843855	0	NGFRAP1		NGFRAP1
1564746_at	NHEDC2	0.679339	0.230227638			
229491_at	NHEDC2	0.9369325	0.796301331			
219353_at	NHLRC2	-0.608423	3.701646186			
239314_at	NHLRC3	0.706184667	0.796301331	NHLRC3		
228933_at	NHS	0.685470667	0.796301331	NHS	NHS	NHS
242800_at	NHS	0.659384333	1.123589586	NHS	NHS	NHS
223981_at	NIN	0.605264	0.550502435			
1552696_at	NIPA1	0.768616667	0			
225752_at	NIPA1	0.6157485	0.05396551			
232158_x_at	NIPAL1	0.725093333	0.393887585			
220128_s_at	NIPAL2	0.784361667	0.796301331			
227001_at	NIPAL2	0.918933167	0			
202380_s_at	NKTR	-0.697418333	0.796301331	NKTR	NKTR	
210113_s_at	NLRP1	0.523906667	3.701646186			
212739_s_at	NME4	-1.0851975	0.137080273	NME4		
203964_at	NMI	0.650043	1.810509752	NMI		
1569631_at	NMNAT1	0.537738333	3.701646186			
1552712_a_at	NMNAT2	-0.616658167	1.810509752			
1556029_s_at	NMNAT2	-0.673486	1.123589586			
209755_at	NMNAT2	-0.966760667	0			
221567_at	NOL3	0.524391333	1.455154512			
59625_at	NOL3	0.631228833	1.810509752			
213462_at	NPAS2	0.575717	0.796301331			
39548_at	NPAS2	1.078871	0			
201467_s_at	NQO1	-0.733438167	0.550502435			
201468_s_at	NQO1	-1.436514167	0			

Probe Set ID	Gene Symbol	miR-128-miRNC	q value (%)	miR-128 tgts_M	miR-128 tgts_TS	miR-128 tgts_PT
203504_s_at	ABCA1	-1.2890325	0	ABCA1	ABCA1	ABCA1
210519_s_at	NQO1	-0.824393333	0.550502435			
204760_s_at	NR1D1 /// THRA	1.198274667	0			
31637_s_at	NR1D1 /// THRA	1.039797	0			
221803_s_at	NRBF2	0.612307	2.499099453	NRBF2	NRBF2	NRBF2
229422_at	NRD1	0.760673667	3.701646186			
208062_s_at	NRG2	0.891594333	0.137080273			
202599_s_at	NRIP1	0.506641667	3.029425085	NRIP1		NRIP1
202600_s_at	NRIP1	0.531353333	1.810509752	NRIP1		NRIP1
202395_at	NSF	0.5910285	1.123589586			
232520_s_at	NSFL1C	-0.549666	2.499099453	NSFL1C		
224666_at	NSMCE1	0.725165167	1.455154512			
226351_at	NSUN4	0.507439667	2.499099453	NSUN4		
230023_at	NSUN4	0.667256	2.499099453	NSUN4		
1553994_at	NT5E	-0.789125667	0	NT5E		
227486_at	NT5E	-0.724681667	0	NT5E		
222020_s_at	NTM	0.57858	3.701646186			
203978_at	NUBP1	0.621153	1.123589586			
219347_at	NUDT15	0.548630167	3.029425085			
224830_at	NUDT21	-0.530133667	3.701646186	NUDT21		
230329_s_at	NUDT6	0.536501	1.810509752	NUDT6		
228855_at	NUDT7	0.562276833	1.810509752			
214963_at	NUP160	-0.719685167	2.499099453			
219007_at	NUP43	-0.690653667	0.230227638	NUP43		
210793_s_at	NUP98	0.576817333	3.029425085			
204435_at	NUPL1	1.368551167	0			
223984_s_at	NUPL1	0.520492667	0.550502435			
218039_at	NUSAP1	-0.585228333	1.123589586	NUSAP1		
209629_s_at	NXT2	-0.581073167	1.455154512	NXT2	NXT2	NXT2
205552_s_at	OAS1	0.802398	0			
218400_at	OAS3	0.833755333	0.05396551			
201365_at	OAZ2	-0.768668667	0.137080273			
228781_at	OBFC1	0.706893	1.810509752			
235537_at	OCIAD1	0.7513805	0.550502435			
209925_at	OCLN	0.587058333	0.550502435			
208316_s_at	OCRL	0.500268833	2.499099453			
200790_at	ODC1	0.566836667	0.796301331			
221090_s_at	OGFOD1	-0.559392667	1.123589586			
225106_s_at	OGFOD1	-0.516329667	2.499099453			
225110_at	OGFOD1	-0.551544667	1.123589586			
226019_at	OMA1	0.842441333	0.230227638			
212213_x_at	OPA1	-1.022419667	0.05396551	OPA1	OPA1	
212214_at	OPA1	-0.507178333	3.029425085	OPA1	OPA1	
214306_at	OPA1	-0.7973645	1.123589586	OPA1	OPA1	
219105_x_at	ORC6L	-0.700021667	0.393887585	ORC6L		
227548_at	ORMDL1	0.579021833	0.393887585	ORMDL1		
222586_s_at	OSBPL11	0.543385	1.810509752			
209221_s_at	OSBPL2	0.932426333	3.701646186			
1554503_a_at	OSCAR	0.831291667	0.550502435			

Probe Set ID	Gene Symbol	miR-128-miRNC	q value (%)	miR-128 tgts_M	miR-128 tgts_TS	miR-128 tgts_PT
203504_s_at	ABCA1	-1.2890325	0	ABCA1	ABCA1	ABCA1
242930_at	OSGEP	0.740957667	0.137080273			
226140_s_at	OTUD1	1.242132	0			
238848_at	OTUD4	0.565106667	0.796301331			
202780_at	OXCT1	-0.522489167	0.796301331			
210448_s_at	P2RX5	0.6323395	2.499099453			
207455_at	P2RY1	0.867078333	1.455154512			
207543_s_at	P4HA1	0.811555667	0.796301331			
202733_at	P4HA2	0.855935	0.796301331			
231838_at	PABPC1L	0.606452667	0.230227638			
201013_s_at	PAICS	-0.794818333	0.05396551	PAICS		
222983_s_at	PAIP2	-0.62235	0.05396551	PAIP2	PAIP2	PAIP2
222984_at	PAIP2	-0.774615	0.137080273	PAIP2	PAIP2	PAIP2
209615_s_at	PAK1	0.633508333	1.123589586			
208877_at	PAK2	0.515177	2.499099453			
208878_s_at	PAK2	0.501206667	1.455154512			
214607_at	PAK3	0.502034333	3.701646186			
202336_s_at	PAM	0.518238333	1.810509752	PAM		
203117_s_at	PAN2	1.061411	0.796301331			
228966_at	PANK2	1.0481015	0.550502435	PANK2		
211918_x_at	PAPPA2	0.794561833	0.393887585			
228237_at	PAPPA2	1.070477	0.137080273			
214834_at	PAR5	-0.674282167	3.701646186			
211907_s_at	PARD6B	1.496513	0		PARD6B	
214827_at	PARD6B	1.69516	0		PARD6B	
235165_at	PARD6B	1.681253333	0		PARD6B	
218271_s_at	PARL	-0.540877833	3.701646186			
228881_at	PARL	-0.518709167	1.455154512			
204752_x_at	PARP2	-0.653551833	1.123589586			
215773_x_at	PARP2	-0.554933667	1.810509752			
234710_s_at	PARP6	0.688543333	0.230227638			
229515_at	PAWR	-0.503239	0.550502435	PAWR		
220355_s_at	PBRM1	0.847014667	0.393887585			
223238_s_at	PBRM1	0.616893167	1.810509752			
223899_at	PBRM1	0.868913167	2.499099453			
223900_s_at	PBRM1	0.567441667	1.810509752			
224152_s_at	PBRM1	0.716808333	0.393887585			
204082_at	PBX3	0.629165	0.393887585			
213517_at	PCBP2	0.793385	1.810509752			
238114_at	PCMTD1	-0.564876833	3.701646186			
219295_s_at	PCOLCE2	0.763237667	1.455154512			
205559_s_at	PCSK5	1.0386215	0			
205560_at	PCSK5	0.882286167	0.05396551			
213652_at	PCSK5	0.990036	0.230227638			
218676_s_at	PCTP	-0.697164167	1.123589586	PCTP		
218953_s_at	PCYOX1L	1.004265833	0			
220049_s_at	PDCD1LG2	0.655231167	2.499099453			
224399_at	PDCD1LG2	0.707691167	0			
204025_s_at	PDCD2	0.683951333	1.810509752			
212594_at	PDCD4	0.644791667	0.796301331			
208591_s_at	PDE3B	-0.671088333	1.123589586		PDE3B	

Probe Set ID	Gene Symbol	miR-128-miRNC	q value (%)	miR-128 tgts_M	miR-128 tgts_TS	miR-128 tgts_PT
203504_s_at	ABCA1	-1.2890325	0	ABCA1	ABCA1	ABCA1
206757_at	PDE5A	1.0000735	1.123589586			
212521_s_at	PDE8A	0.598371667	0.137080273			
205463_s_at	PDGFA	0.654759333	0.393887585			
216867_s_at	PDGFA	0.799891667	1.123589586			
222860_s_at	PDGFD	1.106136833	0			
203067_at	PDHX	-0.729284333	1.455154512	PDHX	PDHX	
211048_s_at	PDIA4	0.713300833	0.393887585			
203857_s_at	PDIA5	-0.9857715	0.393887585	PDIA5	PDIA5	PDIA5
206686_at	PDK1	-0.754323333	0	PDK1	PDK1	
226452_at	PDK1	-0.742918333	0.05396551	PDK1	PDK1	
206348_s_at	PDK3	0.931314833	1.123589586			
203243_s_at	PDLIM5	0.632546667	0.796301331			
224986_s_at	PDPK1	-0.610532167	0.393887585		PDPK1	
1554634_at	PDS5B	-1.396893167	0	PDS5B	PDS5B	PDS5B
219307_at	PDSS2	0.985278667	0			
209493_at	PDZD2	0.6899595	0.796301331			
200787_s_at	PEA15	-0.698291667	3.029425085			
200788_s_at	PEA15	-0.654291667	0.796301331			
205353_s_at	PEBP1	-0.548215	1.810509752			
210825_s_at	PEBP1	-0.50309	1.810509752			
211941_s_at	PEBP1	-0.8817345	1.123589586			
1553140_at	PELO	0.671989167	0.137080273			
208518_s_at	PER2	0.955391833	0.393887585			
1555131_a_at	PER3	1.117877167	0			
41858_at	PGAP2	0.690179833	1.810509752	PGAP2		
243815_at	PGBD4	0.749064333	0.550502435			
203501_at	PGCP	0.955409333	0.550502435			
208454_s_at	PGCP	0.544722	0.550502435			
235615_at	PGGT1B	-0.680436167	3.029425085	PGGT1B		
229553_at	PGM2L1	0.885473167	0.393887585		PGM2L1	PGM2L1
222622_at	PGP	-0.644207833	1.810509752			
213638_at	PHACTR1	0.720334333	0.230227638			
219235_s_at	PHACTR4	0.565450333	1.455154512			
200658_s_at	PHB	-1.0250625	1.810509752	PHB	PHB	PHB
200919_at	PHC2	-1.017561333	0.550502435	PHC2	PHC2	
238693_at	PHC3	0.766399333	1.455154512			
204866_at	PHF16	0.7631665	1.810509752			
235389_at	PHF20	-0.588654	3.701646186	PHF20	PHF2	
224442_at	PHF6	-0.7646435	1.455154512	PHF6		PHF6
201397_at	PHGDH	-0.825602167	0.05396551	PHGDH	PHGDH	
217999_s_at	PHLDA1	0.545875	1.455154512			
225842_at	PHLDA1	0.534828333	2.499099453			
212134_at	PHLDB1	0.720218	1.123589586			
213407_at	PHLPP2	0.6514845	0.796301331			
210191_s_at	PHTF1	0.560072667	0.137080273			
209780_at	PHTF2	0.525441333	3.701646186			
217097_s_at	PHTF2	0.698307167	1.810509752			
203035_s_at	PIAS3	0.535269333	2.499099453			
212511_at	PICALM	-0.594329167	3.701646186			
205078_at	PIGF	0.746955167	0.796301331			

Probe Set ID	Gene Symbol	miR-128-miRNC	q value (%)	miR-128 tgts_M	miR-128 tgts_TS	miR-128 tgts_PT
203504_s_at	ABCA1	-1.2890325	0	ABCA1	ABCA1	ABCA1
209625_at	PIGH	0.879984833	0			
213889_at	PIGL	1.003486333	0.137080273			
219048_at	PIGN	1.151709	0			
232101_s_at	PIGN	1.500877167	0			
1552291_at	PIGX	0.596370333	1.810509752			
1563111_a_at	PIGX	0.552261333	1.455154512			
224660_at	PIGY	-0.674981833	0.393887585			
213070_at	PIK3C2A	-0.8423785	0.550502435	PIK3C2A		
203879_at	PIK3CD	-0.500533	3.701646186			
1557719_at	PIKFYVE	0.656238333	1.123589586			
219155_at	PITPNC1	0.5456575	0.796301331			
218667_at	PJA1	0.568142333	1.455154512			
203688_at	PKD2	0.588998333	3.029425085			
204612_at	PKIA	0.671091667	0.550502435	PKIA		PKIA
216004_s_at	PKNOX1	0.659656	1.455154512			
210145_at	PLA2G4A	1.373579667	0			
219014_at	PLAC8	-0.6853255	1.123589586			
209318_x_at	PLAGL1	-0.981530333	0	PLAGL1		
211925_s_at	PLCB1	0.821602667	1.123589586	PLCB1		
224505_s_at	PLCD4	0.512745333	2.499099453			
204613_at	PLCG2	0.531142833	1.810509752			
205934_at	PLCL1	1.514160167	0			
213309_at	PLCL2	0.589469167	2.499099453	PLCL2		
218951_s_at	PLCXD1	0.6258695	2.499099453			
235230_at	PLCXD2	-0.742183833	3.701646186			
244430_at	PLD6	-0.785467667	2.499099453	PLD6		
201373_at	PLEC1	-0.724834167	3.029425085			
216971_s_at	PLEC1	-0.783949333	3.029425085			
226247_at	PLEKHA1	0.956074167	0	PLEKHA1		
238013_at	PLEKHA2	0.607759167	1.455154512	PLEKHA2		
233040_at	PLEKHA5	-0.755658167	3.029425085	PLEKHA5		
227247_at	PLEKHA8	0.817732	0.550502435			
202122_s_at	PLIN3	-0.6204925	2.499099453			
202240_at	PLK1	-0.8177395	2.499099453			
201939_at	PLK2	-0.53267	1.455154512	PLK2	PLK2	PLK2
204938_s_at	PLN	-0.629954833	2.499099453			
205190_at	PLS1	0.662077333	3.701646186			
1558140_at	PLXNA1	0.917825667	1.123589586			
221538_s_at	PLXNA1	0.850208167	1.455154512			
1567519_at	PLXNA3	0.617073667	3.029425085			
206471_s_at	PLXNC1	1.020073333	0.137080273			
217875_s_at	PMEPA1	0.880956833	1.810509752			
222449_at	PMEPA1	1.556049333	0			
222450_at	PMEPA1	1.519276333	0.05396551			
212037_at	PNN	-0.84844	1.810509752			
212836_at	POLD3	-0.579267167	0.230227638			
212100_s_at	POLDIP3	0.794360667	0.550502435			
215357_s_at	POLDIP3	0.6503445	0.796301331			
219317_at	POLI	0.544472833	1.123589586			
223261_at	POLK	0.6424215	3.701646186			

Probe Set ID	Gene Symbol	miR-128-miRNC	q value (%)	miR-128 tgts_M	miR-128 tgts_TS	miR-128 tgts_PT
203504_s_at	ABCA1	-1.2890325	0	ABCA1	ABCA1	ABCA1
209317_at	POLR1C	0.71646	0.796301331			
231041_at	POLR1E	0.524183333	1.810509752			
211730_s_at	POLR2L	-0.6459745	1.810509752			
227872_at	POLR3A	0.920119	0			
208361_s_at	POLR3D	0.808801333	0.796301331			
222490_at	POLR3E	0.532518	3.701646186			
217944_at	POMGNT1	0.888077	3.701646186			
242700_at	PON2	0.592585333	0	PON2		
204839_at	POP5	0.5068985	1.810509752			
209482_at	POP7	-0.510388833	3.701646186			
212226_s_at	PPAP2B	-1.288412167	0.796301331	PPAP2B		
212230_at	PPAP2B	-0.918661	0.796301331	PPAP2B		
223568_s_at	PPAPDC1B	0.727580167	0.393887585			
223569_at	PPAPDC1B	0.893052	0			
226150_at	PPAPDC1B	1.238480667	0			
226384_at	PPAPDC1B	0.656990167	0.550502435			
206870_at	PPARA	1.076342333	0		PPARA	
223438_s_at	PPARA	1.015817667	0		PPARA	
244689_at	PPARA	1.217887667	1.455154512		PPARA	
215959_at	PPFIBP2	0.933170167	0	PPFIBP2		
226336_at	PPIA	-0.686982167	2.499099453			
204517_at	PPIC	0.575556667	3.701646186			
204518_s_at	PPIC	0.700708333	0.393887585			
204186_s_at	PPID	0.614433333	0.230227638			
228469_at	PPID	0.791551333	3.701646186			
201489_at	PPIF	-0.818572833	0.137080273	PPIF	PPIF	
201490_s_at	PPIF	-0.805433	0.550502435	PPIF	PPIF	
217841_s_at	PPME1	-0.789642333	3.701646186	PPME1	PPME1	PPME1
49077_at	PPME1	-0.846704833	0.05396551	PPME1	PPME1	PPME1
200726_at	PPP1CC	-0.739175	0.230227638	PPP1CC	PPP1CC	PPP1CC
201702_s_at	PPP1R10	0.575063333	1.810509752			
216347_s_at	PPP1R13B	1.2429255	0			
224692_at	PPP1R15B	1.026138	0.05396551			
1552670_a_at	PPP1R3B	0.581086667	0.796301331			
222662_at	PPP1R3B	0.816196167	0.230227638			
204554_at	PPP1R3D	0.825358167	0.796301331			
227409_at	PPP1R3E	0.780701167	0.05396551			
227412_at	PPP1R3E	0.629643	3.029425085			
231966_at	PPP1R9A	0.687054167	3.701646186		PPP1R9A	
202313_at	PPP2R2A	0.597698333	0.796301331	PPP2R2A		
225066_at	PPP2R2D	0.831368	0.230227638			
209633_at	PPP2R3A	-0.646892667	0.137080273	PPP2R3A		
209817_at	PPP3CB	0.943735333	0			
203529_at	PPP6C	0.678163	0.550502435			
225426_at	PPP6C	1.224638333	0			
225429_at	PPP6C	0.959136	0.137080273			
225204_at	PPTC7	0.51952	2.499099453			
225213_at	PPTC7	0.53257	3.029425085			
230015_at	PRCD	0.781360333	1.455154512			
217192_s_at	PRDM1	0.815375833	0.230227638			

Probe Set ID	Gene Symbol	miR-128-miRNC	q value (%)	miR-128 tgts_M	miR-128 tgts_TS	miR-128 tgts_PT
203504_s_at	ABCA1	-1.2890325	0	ABCA1	ABCA1	ABCA1
228964_at	PRDM1	1.023198	2.499099453			
201923_at	PRDX4	0.75584	0			
224909_s_at	PREX1	0.945176333	3.701646186			
226065_at	PRICKLE1	-1.089096667	0	PRICKLE1		
226069_at	PRICKLE1	-0.8546405	0.230227638	PRICKLE1		
230708_at	PRICKLE1	-0.867449333	1.455154512	PRICKLE1		
225968_at	PRICKLE2	1.066466333	3.029425085			PRICKLE2
227892_at	PRKAA2	0.786268	0.05396551			
240349_at	PRKAA2	0.5156745	3.029425085			
1558027_s_at	PRKAB2	0.959563333	0			
214474_at	PRKAB2	0.993001333	0.05396551			
225278_at	PRKAB2	0.724419833	0.393887585			
233748_x_at	PRKAG2	-0.620658667	2.499099453	PRKAG2		
242482_at	PRKAR1A	1.503585833	0			
213010_at	PRKCDBP	-0.872613167	1.455154512			
205880_at	PRKD1	-0.517797833	2.499099453	PRKD1	PRKD1	PRKD1
211380_s_at	PRKG1	0.981884333	0			
226687_at	PRPF40A	-1.136711833	0.05396551			
202126_at	PRPF4B	0.549811333	0.796301331			
219392_x_at	PRR11	-0.671418333	0			
204919_at	PRR4	0.532517167	2.499099453			
202458_at	PRSS23	-0.961693333	0			
226279_at	PRSS23	-0.638842	1.810509752			
229441_at	PRSS23	-0.702657	1.810509752			
229178_at	PRTG	0.720053	0.137080273	PRTG		
209599_s_at	PRUNE	1.059869667	0			
232490_s_at	PRUNE	0.817574833	0.230227638			
226381_at	PS1TP4	-0.636035667	1.810509752			
203354_s_at	PSD3	0.552446667	0.393887585			
203355_s_at	PSD3	0.836010667	0.393887585			
201316_at	PSMA2	-0.5346315	0			
204279_at	PSMB9	0.567619167	3.029425085	PSMB9		
202353_s_at	PSMD12	-0.524493333	0.796301331			
228217_s_at	PSMG4	-0.640708333	1.123589586			
205194_at	PSPH	0.625292333	2.499099453			
201896_s_at	PSRC1	-0.921874	0.137080273			
230129_at	PSTK	0.8569735	0.137080273			
211271_x_at	PTBP1	-0.71181	3.701646186			
218683_at	PTBP2	-0.6487545	0.550502435	PTBP2		
218967_s_at	PTER	-0.993176667	1.810509752	PTER		PTER
222798_at	PTER	-0.850812333	1.123589586	PTER		PTER
230774_at	PTGR2	0.6760315	2.499099453			
238669_at	PTGS1	2.041026667	1.123589586			
209466_x_at	PTN	1.760239333	0			
211737_x_at	PTN	2.030057667	0			
200732_s_at	PTP4A1	0.564447333	1.123589586			
208615_s_at	PTP4A2	-0.598748333	3.029425085			
227741_at	PTPLB	0.515265333	2.499099453			
213136_at	PTPN2	0.668641	1.123589586			
241622_at	PTPN2	0.722165667	3.029425085			

**Probe Set ID**	**Gene Symbol**	**miR-128-miRNC**	**q value (%)**	**miR-128 tgts_M**	**miR-128 tgts_TS**	**miR-128 tgts_PT**
203504_s_at	ABCA1	-1.2890325	0	ABCA1	ABCA1	ABCA1
241623_at	PTPN2	0.588292333	0.796301331			
241983_at	PTPN2	0.934103167	3.701646186			
1320_at	PTPN21	0.8744265	0.550502435			
205438_at	PTPN21	0.830026333	0.05396551			
226380_at	PTPN21	0.694218333	3.029425085			
40524_at	PTPN21	0.991356	0			
208010_s_at	PTPN22	2.186679167	0			
227944_at	PTPN3	0.9068975	0		PTPN3	PTPN3
213795_s_at	PTPRA	0.634148333	2.499099453	PTPRA		
210173_at	PTPRJ	1.562673333	0	PTPRJ		
214137_at	PTPRJ	0.5507235	1.810509752	PTPRJ		
227396_at	PTPRJ	1.300752	0	PTPRJ		
203029_s_at	PTPRN2	0.603990667	2.499099453			
203030_s_at	PTPRN2	0.651452167	3.701646186			
211534_x_at	PTPRN2	0.818272667	1.123589586			
203554_x_at	PTTG1	-0.71956	1.810509752			
206157_at	PTX3	-0.554395333	1.810509752			
1553280_at	PUS10	0.963974	0.550502435			
214443_at	PVR	0.867335167	0.230227638	PVR		
214444_s_at	PVR	0.618465	1.455154512	PVR		
216283_s_at	PVR	0.535713667	3.029425085	PVR		
32699_s_at	PVR	0.5120695	1.810509752	PVR		
1562153_a_at	PVT1	-0.959949333	1.123589586	PVT1		
201607_at	PWP1	-0.624076833	1.123589586			
228336_at	PWWP2A	0.842858333	3.029425085	PWWP2A		
228337_at	PWWP2A	0.754866833	1.123589586	PWWP2A		
1552275_s_at	PXK	0.519450333	1.810509752			
201087_at	PXN	-0.660811167	0.796301331		PXN	
211823_s_at	PXN	-0.5105865	3.029425085		PXN	
215520_at	PYGO1	-0.507015167	1.455154512			
209123_at	QDPR	-0.858428167	0.05396551			
205174_s_at	QPCT	1.436272333	0			
229982_at	QSER1	0.574061667	0.550502435			
203831_at	R3HDM2	0.538233333	1.123589586	R3HDM2		
203883_s_at	RAB11FIP2	0.663509333	3.029425085	RAB11FIP2		
243496_at	RAB18	0.6137905	3.701646186			
213405_at	RAB22A	-0.891013667	0.550502435			
209515_s_at	RAB27A	-0.513471667	1.810509752	RAB27A		
207018_s_at	RAB27B	0.721332833	3.029425085			
227842_at	RAB30	-0.701617	3.701646186			
224710_at	RAB34	-0.626698833	1.455154512	RAB34		
223471_at	RAB3IP	0.813611333	0.393887585			
228432_at	RAB3IP	0.919318333	0.05396551			
231399_at	RAB3IP	0.776162167	1.810509752			
238526_at	RAB3IP	1.013137667	1.455154512			
238853_at	RAB3IP	1.156893	0			
204547_at	RAB40B	0.712555167	0.393887585			
228521_s_at	RAB4B	0.759191833	0			
208819_at	RAB8A	-0.650670333	0.796301331			
213982_s_at	RABGAP1L	0.502083333	1.810509752	RABGAP1L		

Probe Set ID	Gene Symbol	miR-128-miRNC	q value (%)	miR-128 tgts_M	miR-128 tgts_TS	miR-128 tgts_PT
203504_s_at	ABCA1	-1.2890325	0	ABCA1	ABCA1	ABCA1
215342_s_at	RABGAP1L	0.6718605	1.455154512	RABGAP1L		
203573_s_at	RABGGTA	0.592765833	1.123589586			
213970_at	RABL3	0.729621333	2.499099453			
222077_s_at	RACGAP1	0.837507167	0.796301331			
200608_s_at	RAD21	-0.589421667	3.701646186	RAD21		
223598_at	RAD23B	-0.7634595	0.230227638			
208393_s_at	RAD50	0.518304333	0.393887585	RAD50		
209349_at	RAD50	0.976756333	0.393887585	RAD50		
37793_r_at	RAD51L3	-0.674233333	0.05396551	RAD51L3		
205647_at	RAD52	0.562477	1.123589586	RAD52		
210630_s_at	RAD52	0.742103167	0	RAD52		
206591_at	RAG1	0.869118667	3.701646186	RAG1		
221809_at	RANBP10	0.628279167	0.393887585			
53987_at	RANBP10	0.644900833	0.137080273			
237856_at	RAP1GDS1	-0.560750333	1.810509752			
225186_at	RAPH1	0.546698333	1.123589586			
225188_at	RAPH1	0.509177333	3.701646186			
232901_at	RARS2	0.933751167	1.455154512			
202677_at	RASA1	-0.544778167	1.810509752			
1553986_at	RASEF	0.887314667	0			
1553122_s_at	RBAK	0.678498333	1.810509752			
228571_at	RBAK	0.906070167	0.393887585			
225396_at	RBBP4	-0.859317333	0.05396551			
237333_at	RBBP4	-0.506253333	1.123589586			
244872_at	RBBP4	-0.6771615	2.499099453			
224780_at	RBM17	-0.522205667	0.550502435			
224068_x_at	RBM22	0.553771167	3.029425085			
212033_at	RBM25	-0.578062833	2.499099453			
208319_s_at	RBM3	-0.783335	0.550502435			
219754_at	RBM41	0.913181833	0.550502435			
229440_at	RBM47	0.7083755	1.123589586			
213852_at	RBM8A	-0.5475355	1.123589586			
217856_at	RBM8A	-0.921110833	0.796301331			
216215_s_at	RBM9	0.513982667	1.810509752			
225310_at	RBMX	-0.594295	0.796301331			
207836_s_at	RBPMS	0.610321667	2.499099453	RBPMS		
218117_at	RBX1	-0.804696167	0.230227638	RBX1		
220202_s_at	RC3H2	0.818058333	0.05396551	RC3H2		
225813_at	RC3H2	0.76392	0.796301331	RC3H2		
230134_s_at	RC3H2	0.793653	0.137080273	RC3H2		
231716_at	RC3H2	0.752139667	0	RC3H2		
238421_at	RC3H2	1.263754	0	RC3H2		
208370_s_at	RCAN1	0.5240925	1.123589586			
204759_at	RCBTB2	1.482002833	0			
212749_s_at	RCHY1	-0.709529667	0.137080273	RCHY1		
204969_s_at	RDX	-0.553103333	1.455154512	RDX		
212398_at	RDX	-0.542493333	1.123589586	RDX		
224366_s_at	REPS1	-0.6564075	0	REPS1	REPS1	REPS1
205645_at	REPS2	1.275104667	0			
204535_s_at	REST	0.544633	2.499099453			

Probe Set ID	Gene Symbol	miR-128-miRNC	q value (%)	miR-128 tgts_M	miR-128 tgts_TS	miR-128 tgts_PT
203504_s_at	ABCA1	-1.2890325	0	ABCA1	ABCA1	ABCA1
233613_x_at	REXO2	0.532053333	1.810509752			
1552651_a_at	RFFL	0.528475333	1.455154512			
1562603_at	RFFL	0.6030065	2.499099453			
237919_at	RFFL	0.766483333	0.393887585			
203225_s_at	RFK	0.687941333	2.499099453			
238810_at	RFX3	1.132478667	0			
208492_at	RFXAP	-0.735281167	3.029425085			
229431_at	RFXAP	-0.8355455	0			
231877_at	RG9MTD2	1.255160667	3.701646186			
242450_at	RGMB	-0.673698667	0.796301331			
1554003_at	RGNEF	-0.6533505	1.455154512			
203169_at	RGP1	0.524015	1.810509752			
220334_at	RGS17	1.349857	0.393887585	RGS17	RGS17	
1554500_a_at	RGS7	0.912780667	0.796301331			
216970_at	RGS7	1.138286	0.393887585			
226948_at	RHBDD1	1.019165	3.701646186			
219202_at	RHBDF2	0.739597167	2.499099453	RHBDF2		
1554539_a_at	RHOF	-0.855184	2.499099453			
222148_s_at	RHOT1	0.502286667	3.701646186	RHOT1		
227196_at	RHPN2	0.684104167	1.810509752			
203431_s_at	RICS	0.507187833	2.499099453			
233781_s_at	RIF1	-0.994803833	0			
208371_s_at	RING1	0.566574167	2.499099453			
202130_at	RIOK3	-0.593442667	0.796301331	RIOK3		
218979_at	RMI1	-1.093354667	0.393887585	RMI1		
212478_at	RMND5A	0.613158	1.455154512	RMND5A		RMND5A
241343_at	RNASEH1	-0.762860667	0	RNASEH1		
221287_at	RNASEL	1.383406667	0			
229285_at	RNASEL	1.20003	0.796301331			
212724_at	RND3	-1.170987	0	RND3	RND3	RND3
208924_at	RNF11	0.634436167	3.701646186			
1565544_at	RNF141	0.9352595	3.701646186		RNF141	
228153_at	RNF144B	0.948850167	0			
235549_at	RNF144B	1.207158167	0			
239012_at	RNF144B	1.2224485	0			
239899_at	RNF145	-0.550807333	3.029425085			
1553127_a_at	RNF168	0.606013333	0.796301331			
220985_s_at	RNF170	1.065640667	1.123589586			
226104_at	RNF170	0.503445333	3.701646186			
242985_x_at	RNF180	1.4435385	0			
230662_at	RNF187	1.035835167	0.137080273			
213038_at	RNF19B	0.746033167	0			
36564_at	RNF19B	0.6451505	0.230227638			
227114_at	RNF214	0.610333667	0.796301331			
235492_at	RNF217	0.958755	0			
204668_at	RNF24	0.616795167	0.796301331			
203286_at	RNF44	0.566009333	2.499099453			
221194_s_at	RNFT1	1.062165667	0			RNFT1
221908_at	RNFT2	1.120356833	1.455154512			
221909_at	RNFT2	0.928233333	1.455154512			

Probe Set ID	Gene Symbol	miR-128-miRNC	q value (%)	miR-128 tgts_M	miR-128 tgts_TS	miR-128 tgts_PT
203504_s_at	ABCA1	-1.2890325	0	ABCA1	ABCA1	ABCA1
223824_at	RNLS	0.518698	3.701646186			
226975_at	RNPC3	0.50113	1.123589586			
218291_at	ROBLD3	-0.5242325	3.029425085			
219550_at	ROBO3	0.685487	0			
202762_at	ROCK2	-0.620832333	3.701646186			
205805_s_at	ROR1	1.5503005	0	ROR1		
210426_x_at	RORA	1.249375667	0			
210479_s_at	RORA	1.155515333	0			
226682_at	RORA	1.276724	0.05396551			
235567_at	RORA	0.755214833	1.810509752			
236266_at	RORA	0.896025333	1.455154512			
1557984_s_at	RPAP3	0.666058333	0.550502435			
218842_at	RPAP3	0.808395333	0.230227638			
207624_s_at	RPGR	0.885616	0.230227638	RPGR		
214744_s_at	RPL23	-0.941844833	0.230227638			
223707_at	RPL27A	-0.852355167	1.455154512			
224763_at	RPL37	0.630891333	0.137080273	RPL37		
224767_at	RPL37	0.836067167	0.796301331	RPL37		
210035_s_at	RPL5 /// SNORA66	-0.785397833	1.455154512			
227722_at	RPS23	0.547763	1.455154512			
1555878_at	RPS24	1.196675833	0	RPS24		
226660_at	RPS6KB1	-0.631320667	1.123589586	RPS6KB1	RPS6KB1	
1566603_s_at	RPUSD3	0.53607	1.455154512			
205540_s_at	RRAGB	0.723603	1.810509752			
222204_s_at	RRN3	0.726771167	0.550502435			
219037_at	RRP15	0.543524667	3.701646186			
214583_at	RSC1A1	0.793487167	3.029425085			
225780_at	RSC1A1	0.656382333	3.029425085			
213750_at	RSL1D1	0.724651667	3.029425085	RSL1D1		
223713_at	RSPH3	0.931953333	0			
225150_s_at	RTKN	0.674969	0.550502435	RTKN		
210222_s_at	RTN1	0.540665167	1.810509752			
1557388_at	RTTN	-0.943568667	3.701646186	RTTN		
1559946_s_at	RUVBL2	-0.657723833	1.810509752	RUVBL2	RUVBL2	
201459_at	RUVBL2	-0.7510735	2.499099453	RUVBL2	RUVBL2	
222614_at	RWDD2B	0.521712	1.123589586			
217728_at	S100A6	-0.781168	1.455154512			
230464_at	S1PR5	0.749063	3.029425085			
208607_s_at	SAA1 /// SAA2	-1.3409285	0.05396551			
214456_x_at	SAA1 /// SAA2	-1.315243	0.230227638			
238673_at	SAMD12	-0.611219	0.796301331	SAMD12	SAMD12	
242062_at	SAMD8	0.753615	0.230227638			
228531_at	SAMD9	0.806537	0.796301331			
226603_at	SAMD9L	0.575068333	1.455154512	SAMD9L		
235643_at	SAMD9L	0.9294115	0.796301331	SAMD9L		
204502_at	SAMHD1	0.97233	0.05396551			
201542_at	SAR1A	-0.800056667	0.230227638	SAR1A	SAR1A	

Probe Set ID	Gene Symbol	miR-128-miRNC	q value (%)	miR-128 tgts_M	miR-128 tgts_TS	miR-128 tgts_PT
203504_s_at	ABCA1	-1.2890325	0	ABCA1	ABCA1	ABCA1
201543_s_at	SAR1A	-0.55528	3.701646186	SAR1A	SAR1A	
210790_s_at	SAR1A	-0.864059667	0.137080273	SAR1A	SAR1A	
203455_s_at	SAT1	0.70527	1.455154512			
210592_s_at	SAT1	0.57232	1.455154512			
213988_s_at	SAT1	0.830095333	0			
203408_s_at	SATB1	0.745767	0.137080273	SATB1		
215064_at	SC5DL	0.839902667	1.123589586	SC5DL		
209741_x_at	SCAPER	1.249410333	0.393887585			
216399_s_at	SCAPER	1.005386167	0.796301331			
1555021_a_at	SCARF1	0.612726167	3.029425085			
206995_x_at	SCARF1	0.676910333	0			
224901_at	SCD5	0.561081333	1.123589586	SCD5		
1554921_a_at	SCEL	1.0900065	2.499099453			
226923_at	SCFD2	0.773794333	0			
203889_at	SCG5	-0.899345667	0			
223341_s_at	SCOC	0.538359333	3.029425085			
224786_at	SCOC	0.727369167	2.499099453			
212556_at	SCRIB	-0.941514	2.499099453			
219234_x_at	SCRN3	0.559715833	3.701646186			
221220_s_at	SCYL2	0.549709333	1.455154512			
218711_s_at	SDPR	-2.230168333	0			
223299_at	SEC11C	0.698121167	0.393887585			
201582_at	SEC23B	0.680719667	3.701646186			
212902_at	SEC24A	0.637486	1.123589586	SEC24A	SEC24A	
244841_at	SEC24A	1.052907167	0	SEC24A	SEC24A	
202375_at	SEC24D	0.624794	3.029425085			
209889_at	SEC31B	0.526237	3.701646186			
217716_s_at	SEC61A1	-0.926416833	0.796301331	SEC61A1	SEC61A1	SEC61A1
222385_x_at	SEC61A1	-0.976724833	0.393887585	SEC61A1	SEC61A1	SEC61A1
218265_at	SECISBP2	0.8263025	3.701646186			
224250_s_at	SECISBP2	1.073666667	0			
221931_s_at	SEH1L	0.593704333	3.701646186	SEH1L		
224888_at	SELI	0.8612305	0			
223209_s_at	SELS	0.814425	0.05396551			
206941_x_at	SEMA3E	1.478296167	0	SEMA3E		
46665_at	SEMA4C	0.7806775	3.029425085			
210124_x_at	SEMA4F	0.7301765	1.455154512	SEMA4F		
228660_x_at	SEMA4F	0.8378555	0.137080273	SEMA4F		
208939_at	SEPHS1	-0.558337333	0			
1553167_a_at	SEPSECS	0.807300833	0.230227638	SEPSECS		
231730_at	SEPSECS	0.764385333	1.123589586	SEPSECS		
235516_at	SEPSECS	0.762840833	0.796301331	SEPSECS		
212413_at	SEPT6	0.529223	3.701646186			
244508_at	SEPT7	-0.988346167	0.137080273	SEPT7		
200969_at	SERP1	0.968447333	0			
200970_s_at	SERP1	0.903086	0.230227638			
235475_at	SERP1	0.517134333	1.810509752			
202833_s_at	SERPINA1	1.775856833	0			
211429_s_at	SERPINA1	1.337586833	0.550502435			
1568765_at	SERPINE1	1.155627333	0.796301331			

**Probe Set ID**	**Gene Symbol**	**miR-128-miRNC**	**q value (%)**	**miR-128 tgts_M**	**miR-128 tgts_TS**	**miR-128 tgts_PT**
203504_s_at	ABCA1	-1.2890325	0	ABCA1	ABCA1	ABCA1
202627_s_at	SERPINE1	0.612526667	1.123589586			
202628_s_at	SERPINE1	0.787611667	0.05396551			
207714_s_at	SERPINH1	0.725395	0.796301331	SERPINH1		
235683_at	SESN3	0.939190833	0.05396551			
235684_s_at	SESN3	0.735329667	0.550502435			
219482_at	SETD4	0.519216667	1.455154512			
244653_at	SETD7	-0.765000833	0.393887585	SETD7	SETD7	
213370_s_at	SFMBT1	1.363956333	0	SFMBT1		
1553581_s_at	SFRS12IP1	0.607704333	0.137080273			
217608_at	SFRS12IP1	0.613699167	2.499099453			
230375_at	SFRS18	1.191120833	0	SFRS18		
238929_at	SFRS2B	1.192863	0			
1570507_at	SFRS2IP	1.171926333	1.810509752			SFRS2IP
237485_at	SFRS3	-0.582805	1.810509752			
239511_s_at	SFRS4	-0.8105235	1.810509752			
239512_at	SFRS4	-0.9725285	1.123589586			
241245_at	SFRS4	-0.813582333	3.029425085			
242837_at	SFRS4	-0.543956333	3.701646186			
213649_at	SFRS7	0.522276667	1.455154512			
243818_at	SFTA1P	-0.81549	0			
1553690_at	SGOL1	-1.215323	0			
208381_s_at	SGPL1	-0.538465	1.810509752			
232084_at	SGTB	0.852472333	0.796301331			
201810_s_at	SH3BP5	0.647566667	0.137080273			
209090_s_at	SH3GLB1	-0.680188333	0.393887585			
1554168_a_at	SH3KBP1	0.639645	0.796301331			
223082_at	SH3KBP1	0.564586667	0.393887585			
235692_at	SH3KBP1	1.000907167	0			
231823_s_at	SH3PXD2B	-0.7521065	1.123589586			
228461_at	SH3RF3	0.8713735	0.230227638			
220563_s_at	SHANK1	0.580878667	2.499099453			
213307_at	SHANK2	1.319562833	0			
243681_at	SHANK2	0.691696333	0.550502435			
1557458_s_at	SHB	1.107883167	0			
204657_s_at	SHB	0.774834	1.455154512			
230459_s_at	SHB	0.705174167	2.499099453			
224954_at	SHMT1	-0.883799	0.05396551			
224391_s_at	SIAE	1.236764833	0.05396551			
209339_at	SIAH2	0.869111667	0.05396551			
215856_at	SIGLEC15	0.6161	0.393887585			
208078_s_at	SIK1	0.6968395	0.550502435			
206558_at	SIM2	0.543818333	3.029425085			
211114_x_at	SIP1	0.633978833	1.123589586			
211115_x_at	SIP1	0.6232385	1.810509752			
1554624_a_at	SIRPB1	-0.50041	0.796301331	SIRPB1		
221562_s_at	SIRT3	-0.595778667	3.029425085			
231797_at	SIX4	0.621904667	1.455154512			
227165_at	SKA3	-0.751773333	0.230227638	SKA3		
204270_at	SKI	1.207287	0			
200711_s_at	SKP1	-0.780598833	0.796301331	SKP1		

Probe Set ID	Gene Symbol	miR-128-miRNC	q value (%)	miR-128 tgts_M	miR-128 tgts_TS	miR-128 tgts_PT
203504_s_at	ABCA1	-1.2890325	0	ABCA1	ABCA1	ABCA1
200719_at	SKP1	-0.841313667	0.05396551	SKP1		
225619_at	SLAIN1	0.9833475	0.230227638	SLAIN1		
224853_at	SLAIN2	0.53839	1.455154512			
224854_s_at	SLAIN2	0.52528	1.810509752			
219159_s_at	SLAMF7	0.848677333	0			
204404_at	SLC12A2	0.769120667	2.499099453			
225835_at	SLC12A2	0.9994895	1.810509752			
223596_at	SLC12A6	0.643794667	0.796301331	SLC12A6		
202234_s_at	SLC16A1	0.656718333	1.455154512	SLC16A1		
213522_s_at	SLC16A3	0.511869333	1.810509752			
207057_at	SLC16A7	0.630545167	2.499099453			
221041_s_at	SLC17A5	0.8117605	1.123589586	SLC17A5		
205857_at	SLC18A2	0.602966	1.810509752			
212810_s_at	SLC1A4	0.648430833	2.499099453			
218136_s_at	SLC25A37	0.609259667	0.550502435	SLC25A37		
222529_at	SLC25A37	0.583514667	3.029425085	SLC25A37		
226179_at	SLC25A37	1.238951667	0	SLC25A37		
242335_at	SLC25A37	0.8448025	0.550502435	SLC25A37		
212683_at	SLC25A44	0.713042667	2.499099453			
1552694_at	SLC2A13	0.940679667	1.123589586			
1552695_a_at	SLC2A13	1.004944833	1.810509752			
227176_at	SLC2A13	1.030843667	1.810509752			
234561_at	SLC2A13	0.894628333	1.810509752			
1555500_s_at	SLC2A4RG	0.5420605	1.455154512			
212907_at	SLC30A1	0.6553525	3.701646186			
228181_at	SLC30A1	0.679873	1.810509752			
1555334_s_at	SLC30A5	0.507325	0.796301331			
218989_x_at	SLC30A5	0.58533	1.455154512			
226217_at	SLC30A7	1.315987833	0.05396551			
226601_at	SLC30A7	0.8121215	0.05396551			
239596_at	SLC30A7	0.827719	1.123589586			
229500_at	SLC30A9	0.902659167	1.810509752			
203164_at	SLC33A1	0.894760333	0			
203165_s_at	SLC33A1	0.642718667	1.810509752			
203306_s_at	SLC35A1	0.526893667	1.123589586	SLC35A1		
206770_s_at	SLC35A3	0.688496667	1.810509752			
240335_at	SLC35F5	-1.115985	0	SLC35F5		
234978_at	SLC36A4	-1.105921667	0			
223304_at	SLC37A3	0.537328667	0.550502435			
214830_at	SLC38A6	-0.940375667	0.05396551	SLC38A6		
227046_at	SLC39A11	-0.643368333	0.137080273	SLC39A11	SLC39A11	SLC39A11
209267_s_at	SLC39A8	0.7559815	0.796301331			
223798_at	SLC41A2	1.629764	0			
235299_at	SLC41A2	1.644309333	0.05396551			
243894_at	SLC41A2	1.606067833	0			
219175_s_at	SLC41A3	0.769987667	2.499099453			
224931_at	SLC41A3	1.168822333	0			
228486_at	SLC44A1	1.331094167	2.499099453			
1552779_a_at	SLC44A5	1.561366833	0			
1569112_at	SLC44A5	1.811000167	0			

Probe Set ID	Gene Symbol	miR-128-miRNC	q value (%)	miR-128 tgts_M	miR-128 tgts_TS	miR-128 tgts_PT
203504_s_at	ABCA1	-1.2890325	0	ABCA1	ABCA1	ABCA1
242030_at	SLC4A1AP	0.529833833	3.029425085			
207603_at	SLC4A7	1.199155	0.05396551			
207604_s_at	SLC4A7	0.963056667	0			
209884_s_at	SLC4A7	0.766845333	0.393887585			
210286_s_at	SLC4A7	0.8087285	3.029425085			
206376_at	SLC6A15	0.604586667	3.029425085			
201195_s_at	SLC7A5	-1.1912105	0			
232057_at	SLC7A6OS	0.535573333	1.123589586			
1552671_a_at	SLC9A7	0.774740833	0.796301331			
219911_s_at	SLCO4A1	0.527905667	1.123589586	SLCO4A1		
214930_at	SLITRK5	-0.502442333	1.123589586			
226563_at	SMAD2	-0.671464333	0.796301331		SMAD2	
235598_at	SMAD2	-0.668606833	2.499099453		SMAD2	
218284_at	SMAD3	0.549375	1.455154512			
225282_at	SMAP2	0.532001167	3.701646186			
212167_s_at	SMARCB1	0.820546833	0.796301331	SMARCB1		
201320_at	SMARCC2	0.603422	3.701646186			
203183_s_at	SMARCD1	0.716861167	1.810509752			
1555677_s_at	SMC1A	0.956876667	0			
201589_at	SMC1A	0.615646167	1.455154512			
201664_at	SMC4	-0.52702	1.455154512			
215623_x_at	SMC4	-1.067805833	0			
204466_s_at	SNCA	1.12192	0			
207827_x_at	SNCA	0.844171667	0			
211546_x_at	SNCA	1.0241	0			
237833_s_at	SNCAIP	-0.639992	1.810509752			
1564911_at	SNHG4	-0.5450645	3.029425085			
219409_at	SNIP1	0.778738	1.810509752			
1558081_at	SNORA65	0.584008167	3.029425085			
212440_at	SNRNP27	-0.5742125	3.029425085	SNRNP27		
215905_s_at	SNRNP40	-0.547254167	3.029425085			
208821_at	SNRPB	-0.5988445	0.550502435			
205315_s_at	SNTB2	0.676255667	2.499099453			
227312_at	SNTB2	0.647428333	1.455154512			
220140_s_at	SNX11	-0.824539667	1.455154512			
224684_at	SNX12	-0.678705	0.393887585			
1555388_s_at	SNX25	0.590326667	1.123589586	SNX25		
227408_s_at	SNX25	0.658667	1.810509752	SNX25		
226249_at	SNX30	-0.676332333	1.455154512			
217792_at	SNX5	-0.732396833	0.137080273			
223028_s_at	SNX9	0.547081667	1.123589586			
218974_at	SOBP	0.922740833	1.810509752	SOBP		
203373_at	SOCS2	-0.718898	0.550502435			
209648_x_at	SOCS5	0.58481	2.499099453	SOCS5		
214462_at	SOCS6	0.674846167	3.029425085	SOCS6	SOCS6	SOCS6
227542_at	SOCS6	0.5960395	2.499099453	SOCS6	SOCS6	SOCS6
214015_at	SOCS7	1.4271755	0			
226572_at	SOCS7	1.343468	0			
228662_at	SOCS7	1.4170485	0			
212560_at	SORL1	0.847322833	3.029425085	SORL1		

Probe Set ID	Gene Symbol	miR-128-miRNC	q value (%)	miR-128 tgts_M	miR-128 tgts_TS	miR-128 tgts_PT
203504_s_at	ABCA1	-1.2890325	0	ABCA1	ABCA1	ABCA1
212797_at	SORT1	-0.782465333	0.393887585			
224818_at	SORT1	-0.553905833	1.810509752			
230943_at	SOX17	1.081499	3.029425085			
238285_at	SOX5	1.104465167	0.796301331			
224013_s_at	SOX7	0.5046145	0.796301331	SOX7	SOX7	
211736_at	SP2	0.842449667	1.810509752	SP2	SP2	SP2
209891_at	SPC25	-0.7327085	0.393887585			
230775_s_at	SPG20	0.513156667	2.499099453			
217827_s_at	SPG21	-0.709795	0.05396551	SPG21		
1568916_at	SPIRE2	1.055942667	2.499099453			
202363_at	SPOCK1	1.476264167	0			
209436_at	SPON1	-0.903211667	0.550502435			
209437_s_at	SPON1	-0.950896333	1.455154512			
213994_s_at	SPON1	-0.682259333	2.499099453			
1568574_x_at	SPP1	0.563135667	3.029425085	SPP1		
204011_at	SPRY2	0.551435	1.455154512	SPRY2	SPRY2	SPRY2
221489_s_at	SPRY4	0.772953333	0			
226075_at	SPSB1	0.674888333	1.123589586			
214856_at	SPTBN1	-0.627543333	1.810509752	SPTBN1	SPTBN1	
215918_s_at	SPTBN1	-0.666427833	1.455154512	SPTBN1	SPTBN1	
203127_s_at	SPTLC2	-0.789832167	0.393887585			
203128_at	SPTLC2	-0.6100975	1.455154512			
216202_s_at	SPTLC2	-0.6639135	1.123589586			
225095_at	SPTLC2	-0.8584805	0			
220456_at	SPTLC3	-0.672413	1.455154512	SPTLC3		
1557352_at	SQLE	0.5330605	3.701646186			
213324_at	SRC	0.796088667	0.137080273			
1556202_at	SRGAP2	-0.826095	3.029425085	SRGAP2	SRGAP2	
208920_at	SRI	-0.728147167	0.550502435	SRI		
208801_at	SRP72	-0.56917	1.123589586	SRP72	SRP72	
218140_x_at	SRPRB	0.602136667	0.550502435			
222532_at	SRPRB	0.575156667	0.796301331			
202816_s_at	SS18	-0.737588333	0.137080273	SS18	SS18	
202817_s_at	SS18	-0.506768333	3.029425085	SS18	SS18	
209954_x_at	SS18	-0.8286185	0.137080273	SS18	SS18	
216684_s_at	SS18	-0.878376667	0.230227638	SS18	SS18	
217051_s_at	SS18	-0.551445167	1.123589586	SS18	SS18	
226080_at	SSH2	0.890498	0.230227638			
204963_at	SSPN	1.259351833	0.05396551			
208666_s_at	ST13	-0.962450667	0.796301331	ST13		
208667_s_at	ST13	-0.8504375	0	ST13		
225033_at	ST3GAL1	0.708831667	0.137080273			
225034_at	ST3GAL1	0.629014333	2.499099453			
210942_s_at	ST3GAL6	-0.628987	1.810509752			
214970_s_at	ST6GAL1	0.656162167	1.455154512			
214971_s_at	ST6GAL1	0.533587333	1.455154512			
235334_at	ST6GALNAC3	1.667912	3.029425085	ST6GALNAC3	ST6GALNAC3	ST6GALNAC3
207524_at	ST7	0.709685667	1.455154512			
206925_at	ST8SIA4	0.921151667	0.393887585	ST8SIA4		

Probe Set ID	Gene Symbol	miR-128-miRNC	q value (%)	miR-128 tgts_M	miR-128 tgts_TS	miR-128 tgts_PT
203504_s_at	ABCA1	-1.2890325	0	ABCA1	ABCA1	ABCA1
221191_at	STAG3L1	-0.771516833	1.810509752	STAG3L1		
208194_s_at	STAM2	0.566896667	0.550502435	STAM2		
209649_at	STAM2	0.868212167	0	STAM2		
215044_s_at	STAM2	0.65217	1.123589586	STAM2		
228254_at	STAM2	1.170690667	0	STAM2		
242569_at	STAM2	1.264610167	0	STAM2		
227606_s_at	STAMBPL1	0.6176295	0.137080273			
227607_at	STAMBPL1	0.5176875	1.810509752			
223103_at	STARD10	1.957563	0			
208991_at	STAT3	0.67012	0			
208992_s_at	STAT3	0.571466667	1.455154512			
225289_at	STAT3	0.619959	1.810509752			
203010_at	STAT5A	0.981746667	0.137080273			
212549_at	STAT5B	0.5691725	0.137080273			
204595_s_at	STC1	1.181283833	0			
204596_s_at	STC1	1.506826333	0			
204597_x_at	STC1	1.377281333	0			
225871_at	STEAP2	0.8292155	0.796301331	STEAP2		
225649_s_at	STK35	0.605697	1.123589586	STK35	STK35	STK35
200783_s_at	STMN1	-0.7810655	3.701646186			
222557_at	STMN3	-0.754426667	1.810509752			
223245_at	STRBP	0.895879333	0			
223246_s_at	STRBP	0.724576667	1.123589586			
233252_s_at	STRBP	0.882638667	0.137080273			
203767_s_at	STS	0.810095333	2.499099453			
203770_s_at	STS	0.799686333	0.230227638			
227625_s_at	STUB1	-0.619897333	2.499099453			
212111_at	STX12	1.1006845	0.230227638			
212112_s_at	STX12	0.8854855	0.05396551			
207346_at	STX2	-0.678603833	1.455154512			
216985_s_at	STX3	0.500682	2.499099453	STX3		
1552618_at	STX6	0.522208333	2.499099453			
212799_at	STX6	1.096709833	0.137080273			
212800_at	STX6	0.627236333	0.796301331			
212631_at	STX7	0.563325	1.455154512	STX7		
202260_s_at	STXBP1	0.679116667	0.393887585			
235180_at	STYX	0.560924333	3.701646186	STYX		
222591_at	STYXL1	0.863884667	3.029425085			
232353_s_at	STYXL1	0.715007333	1.810509752			
233982_x_at	STYXL1	0.752477333	2.499099453			
221727_at	SUB1	-0.729936167	1.123589586			
224974_at	SUDS3	0.5216065	3.701646186			
222749_at	SUFU	0.7016565	3.029425085	SUFU		
226850_at	SUMF1	-2.200779	0	SUMF1		
201483_s_at	SUPT4H1	0.6123355	1.810509752			
225038_s_at	SURF6	0.6331025	1.810509752			
214954_at	SUSD5	0.668346667	1.810509752			
218619_s_at	SUV39H1	-0.8109265	3.701646186			
212287_at	SUZ12	-0.890667333	1.810509752	SUZ12	SUZ12	
230285_at	SVIP	0.514023333	0.550502435	SVIP		

Probe Set ID	Gene Symbol	miR-128-miRNC	q value (%)	miR-128 tgts_M	miR-128 tgts_TS	miR-128 tgts_PT
203504_s_at	ABCA1	-1.2890325	0	ABCA1	ABCA1	ABCA1
209307_at	SWAP70	0.632659667	2.499099453	SWAP70		
221276_s_at	SYNC	-0.658555833	1.455154512			
1558392_at	SYNE2	-0.730457667	0	SYNE2		
202761_s_at	SYNE2	-0.504632	3.029425085	SYNE2		
242774_at	SYNE2	-0.906810333	0.05396551	SYNE2		
243841_at	SYNE2	-0.635974167	0.796301331	SYNE2		
210612_s_at	SYNJ2	0.844315	0	SYNJ2		
1555052_a_at	SYT9	1.4037735	0.05396551	SYT9		
220613_s_at	SYTL2	1.029320667	1.810509752			
238423_at	SYTL3	0.8480625	3.029425085			
227703_s_at	SYTL4	0.521606333	1.455154512	SYTL4		
236248_x_at	TADA2B	0.991923167	0			
209938_at	TADA2L	0.715646167	1.123589586			
210537_s_at	TADA2L	0.829723167	0.550502435			
215273_s_at	TADA3L	0.809377667	0.393887585			
205966_at	TAF13	0.5609	1.123589586			
227884_at	TAF15	-0.687205167	0.393887585			
227891_s_at	TAF15	-0.584871833	1.810509752			
234168_at	TAF15	-0.755754833	0.137080273			
235154_at	TAF3	0.613657833	3.029425085			
216226_at	TAF4B	0.647391667	0.137080273			
210294_at	TAPBP	0.590009833	2.499099453			
218747_s_at	TAPBPL	0.706608333	0.393887585			
221264_s_at	TARDBP	-0.524774667	1.455154512			
217374_x_at	TARP /// TRGV3 /// TRGV5	1.888488167	0.230227638			
240206_at	TARS	0.510187333	0.550502435			
227611_at	TARSL2	1.103340333	1.123589586	TARSL2		
234042_at	TAS2R45	-0.969981667	0.796301331			
234108_at	TAS2R45	-0.686065833	2.499099453			
219443_at	TASP1	0.961827667	0	TASP1		
223231_at	TATDN1	0.537768333	1.455154512			
1568713_a_at	TBC1D1	0.705231	0.550502435	TBC1D1		
1569566_at	TBC1D1	0.650726333	3.029425085	TBC1D1		
222173_s_at	TBC1D2	-0.8869435	1.123589586			
226409_at	TBC1D20	0.674587667	0.550502435			
226664_at	TBC1D20	0.694756667	0.550502435			
227908_at	TBC1D24	0.892021333	2.499099453			
212796_s_at	TBC1D2B	0.737558833	2.499099453			
219771_at	TBC1D8B	0.875430667	0.230227638	TBC1D8B		
227395_at	TBCEL	0.751352667	2.499099453			
231997_at	TBCEL	1.112826	0.796301331			
233633_at	TBL1XR1	-0.916004333	2.499099453			
212685_s_at	TBL2	0.5223235	2.499099453			
208398_s_at	TBPL1	0.519806667	0.550502435			
226318_at	TBRG1	0.979702167	0.230227638			
219682_s_at	TBX3	0.814511333	1.455154512	TBX3		
225544_at	TBX3	0.954022667	1.455154512	TBX3		
226388_at	TCEA3	0.614503667	3.029425085			

Probe Set ID	Gene Symbol	miR-128-miRNC	q value (%)	miR-128 tgts_M	miR-128 tgts_TS	miR-128 tgts_PT
203504_s_at	ABCA1	-1.2890325	0	ABCA1	ABCA1	ABCA1
200085_s_at	TCEB2	-0.778419	2.499099453			
213877_x_at	TCEB2	-0.628985333	2.499099453			
202384_s_at	TCOF1	0.8525655	0.796301331			
205796_at	TCP11L1	0.865305333	0.550502435	TCP11L1		
205797_s_at	TCP11L1	0.824308333	1.455154512	TCP11L1		
1553861_at	TCP11L2	1.060875667	0.137080273	TCP11L2		
1565525_a_at	TCP11L2	0.846262333	1.123589586	TCP11L2		
228606_at	TCTEX1D2	0.544839167	1.455154512			
213361_at	TDRD7	0.782020667	0.137080273			
221053_s_at	TDRKH	1.317947	0.550502435			
226408_at	TEAD2	0.575376833	0.796301331			
228670_at	TEP1	0.798329333	1.455154512			
1569385_s_at	TET2	0.638883333	1.123589586	TET2	TET2	
214754_at	TET3	1.414618833	0.05396551			
235542_at	TET3	1.295641333	0			
218099_at	TEX2	0.523061	3.701646186	TEX2		
204654_s_at	TFAP2A	1.255121167	0	TFAP2A		
205286_at	TFAP2C	-0.8909145	0.796301331			
242939_at	TFDP1	-1.026494167	0	TFDP1		
203589_s_at	TFDP2	-0.505297167	1.123589586			
226157_at	TFDP2	-0.593121833	0.393887585			
206649_s_at	TFE3	0.754424667	1.455154512			
209277_at	TFPI2	1.260664333	0			
209278_s_at	TFPI2	0.954093333	0			
208249_s_at	TGDS	0.543651833	1.810509752			
205015_s_at	TGFA	0.662998667	2.499099453			
211258_s_at	TGFA	0.679819	1.123589586			
201042_at	TGM2	-0.739011667	0.05396551			
211003_x_at	TGM2	-0.781088333	1.455154512			
211573_x_at	TGM2	-0.628103333	2.499099453			
219292_at	THAP1	0.730982167	3.701646186			
227636_at	THAP5	0.967545833	1.123589586			
244190_at	THAP5	0.844239	0			
227813_at	THAP6	0.670287667	1.123589586	THAP6		
229253_at	THEM4	-0.724299	0	THEM4		
219122_s_at	THG1L	0.540751667	3.701646186			
222931_s_at	THNSL1	1.239501	0			
242163_at	THRAP3	1.336853167	0			
219153_s_at	THSD4	0.5345	1.810509752			
206555_s_at	THUMPD1	0.867338333	0.550502435			
213025_at	THUMPD1	0.707188333	3.029425085			
228234_at	TICAM2 /// TMED7-TICAM2	0.533758667	0.796301331			
239431_at	TICAM2 /// TMED7-TICAM2	0.502503333	1.123589586			
201821_s_at	TIMM17A	0.558996667	1.455154512	TIMM17A		
215171_s_at	TIMM17A	0.601049833	1.123589586	TIMM17A		
217612_at	TIMM50	-0.794937	3.701646186			

Probe Set ID	Gene Symbol	miR-128-miRNC	q value (%)	miR-128 tgts_M	miR-128 tgts_TS	miR-128 tgts_PT
203504_s_at	ABCA1	-1.2890325	0	ABCA1	ABCA1	ABCA1
201666_at	TIMP1	0.594584333	0.796301331			
201147_s_at	TIMP3	-1.207296667	0			
201149_s_at	TIMP3	-1.3090635	0			
201150_s_at	TIMP3	-1.579177333	0			
220052_s_at	TINF2	0.510486	0.550502435			
223776_x_at	TINF2	0.5302055	0.230227638			
1554091_a_at	TIRAP	1.183966	0			
1554408_a_at	TK1	-0.880844	3.701646186			
203220_s_at	TLE1	0.842310167	1.455154512	TLE1		
203222_s_at	TLE1	0.6881575	0	TLE1		
204872_at	TLE4	-1.330407	0			
216997_x_at	TLE4	-0.56603	1.123589586			
233575_s_at	TLE4	-0.500881667	1.123589586			
235765_at	TLE4	-0.764995167	0.796301331			
211077_s_at	TLK1	0.677716667	1.123589586			
211703_s_at	TM2D1	0.557349333	0.393887585			
220639_at	TM4SF20	-1.2259335	1.810509752	TM4SF20		
217974_at	TM7SF3	0.998865	0			
226478_at	TM7SF3	0.974641	0.05396551			
209150_s_at	TM9SF1	0.514476167	1.123589586			
213349_at	TMCC1	0.569825833	0.796301331		TMCC1	TMCC1
213351_s_at	TMCC1	0.646854	0.796301331		TMCC1	TMCC1
213352_at	TMCC1	0.856589333	2.499099453		TMCC1	TMCC1
205812_s_at	TMED9	0.980326667	1.455154512			
224496_s_at	TMEM107	-2.319192667	0			
235490_at	TMEM107	-2.558746333	0			
218834_s_at	TMEM132A	1.184536667	0			
219750_at	TMEM144	0.889858167	1.455154512			
220169_at	TMEM156	1.2796385	0			
241844_x_at	TMEM156	0.797299833	0			
223202_s_at	TMEM164	0.902658667	1.810509752			
226276_at	TMEM167A	-0.536558167	0.550502435	TMEM167A	TMEM167 A	
222495_at	TMEM167B	0.671421333	1.810509752			
229302_at	TMEM178	1.258114	0	TMEM178		
219253_at	TMEM185B	0.551659667	1.810509752			
225374_at	TMEM199	0.523440333	1.455154512			
239265_at	TMEM20	0.6924135	0.05396551	TMEM20		
222752_s_at	TMEM206	0.702911667	0.796301331			
222736_s_at	TMEM38B	0.824119333	0.05396551			
241392_at	TMEM39A	0.791331833	0			
219410_at	TMEM45A	0.895070833	0			
218073_s_at	TMEM48	-0.695563167	1.455154512	TMEM48		
231697_s_at	TMEM49	0.756098333	0.05396551			
204807_at	TMEM5	0.846499333	2.499099453			
204808_s_at	TMEM5	0.52001	0.230227638			
218815_s_at	TMEM51	0.827264	0			
226338_at	TMEM55A	0.9027805	1.123589586			
234980_at	TMEM56	0.868272167	3.701646186			
241342_at	TMEM65	1.23662	0.550502435			

Probe Set ID	Gene Symbol	miR-128-miRNC	q value (%)	miR-128 tgts_M	miR-128 tgts_TS	miR-128 tgts_PT
203504_s_at	ABCA1	-1.2890325	0	ABCA1	ABCA1	ABCA1
219448_at	TMEM70	0.903055	3.701646186	TMEM70		
225411_at	TMEM87B	0.513362333	3.701646186	TMEM87B		
231408_at	TMEM92	1.1122945	0			
235245_at	TMEM92	0.722644167	0.05396551			
214948_s_at	TMF1	0.997422333	0			
209753_s_at	TMPO	-0.503368333	1.123589586			
217733_s_at	TMSB10	-0.877435	0.393887585	TMSB10	TMSB1	TMSB10
226604_at	TMTC3	0.959635667	1.455154512			
208296_x_at	TNFAIP8	0.878491333	1.123589586			
210260_s_at	TNFAIP8	0.938972	0			
1552648_a_at	TNFRSF10A	0.768871833	0.05396551			
231775_at	TNFRSF10A	0.652983	0.393887585			
210654_at	TNFRSF10D	0.789658333	0	TNFRSF10D		
227345_at	TNFRSF10D	0.633996667	0.550502435	TNFRSF10D		
204933_s_at	TNFRSF11B	0.558738333	0.796301331			
214581_x_at	TNFRSF21	1.065331333	0.550502435			
218856_at	TNFRSF21	0.895401333	0			
216042_at	TNFRSF25	1.102461333	0.137080273			
221085_at	TNFSF15	2.783961	0			
218335_x_at	TNIP2	0.837049833	1.123589586			
232160_s_at	TNIP2	0.871409667	0.550502435			
48531_at	TNIP2	0.7555	0.550502435			
220655_at	TNIP3	2.921262833	0			
222562_s_at	TNKS2	0.530732667	3.029425085			
213201_s_at	TNNT1	1.089247333	0.550502435			
1557278_s_at	TNPO1	-0.8537615	0.137080273	TNPO1	TNPO1	
212635_at	TNPO1	-0.738175	0.550502435	TNPO1	TNPO1	
225765_at	TNPO1	-0.570330167	3.029425085	TNPO1	TNPO1	
1553346_a_at	TNRC6A	0.818029667	1.455154512			
224705_s_at	TNRC6A	0.745126667	0.137080273			
202704_at	TOB1	-0.755804167	2.499099453			
216100_s_at	TOR1AIP1	1.03633	0.05396551			
223930_at	TOR1AIP1	0.773395167	1.123589586			
209593_s_at	TOR1B	0.629693167	1.123589586			
232097_at	TOX4	1.166072	1.455154512	TOX4		
203476_at	TPBG	0.994011667	0			
201689_s_at	TPD52	0.558628333	3.701646186			
201691_s_at	TPD52	0.776072	0.393887585			
203786_s_at	TPD52L1	0.506874	2.499099453	TPD52L1		
221218_s_at	TPK1	0.809814667	0.230227638			
223686_at	TPK1	1.046712833	0			
1558532_at	TPM1	0.602433833	1.455154512	TPM1		
204083_s_at	TPM2	0.933481667	0			
212654_at	TPM2	0.952541833	3.029425085			
224164_at	TPM3	-0.693503667	3.701646186			
214195_at	TPP1	0.917888833	0			
224871_at	TPRG1L	0.6953985	1.123589586			
223031_s_at	TRAF7	-0.606823	3.029425085	TRAF7		
242497_at	TRAFD1	0.5459815	3.701646186	TRAFD1		
214924_s_at	TRAK1	0.815688833	3.029425085	TRAK1		

Probe Set ID	Gene Symbol	miR-128-miRNC	q value (%)	miR-128 tgts_M	miR-128 tgts_TS	miR-128 tgts_PT
203504_s_at	ABCA1	-1.2890325	0	ABCA1	ABCA1	ABCA1
226013_at	TRAK1	0.6949135	0	TRAK1		
202124_s_at	TRAK2	1.066334333	0			
202125_s_at	TRAK2	1.110968667	0			
217958_at	TRAPPC4	-1.002235167	0	TRAPPC4		
217959_s_at	TRAPPC4	-1.277003333	0	TRAPPC4		
233324_at	TRERF1	0.841818	0.05396551			
238520_at	TRERF1	0.983617333	0			
216298_at	TRGV5	1.331995833	0			
202241_at	TRIB1	1.598379333	0			
235641_at	TRIB1	1.157437	0.137080273			
1559681_a_at	TRIM16L	0.675759833	0	TRIM16L		
204732_s_at	TRIM23	0.813490667	3.701646186	TRIM23	TRIM23	
210994_x_at	TRIM23	0.5782615	2.499099453	TRIM23	TRIM23	
210995_s_at	TRIM23	0.565737333	0.393887585	TRIM23	TRIM23	
212118_at	TRIM27	0.5668205	0.393887585			
223830_s_at	TRIM5	0.750801167	0.393887585			
236175_at	TRIM55	-0.568981667	1.455154512	TRIM55		
231876_at	TRIM56	0.7408785	0.393887585			
230280_at	TRIM9	2.0382055	0.230227638			
1552580_at	TRIML2	0.890231667	0			
209778_at	TRIP11	0.598265	0.137080273			
204033_at	TRIP13	-0.695190833	0			
218877_s_at	TRMT11	0.50445	3.701646186			
221952_x_at	TRMT5	-0.87542	1.455154512			
222768_s_at	TRMT6	0.527885	1.455154512			
233970_s_at	TRMT6	0.751111167	0.05396551			
227862_at	TRNP1	0.530347333	0			
236052_at	TRNP1	0.606472	2.499099453			
207520_at	TROVE2	-0.937816667	1.455154512	TROVE2	TROVE2	TROVE2
210438_x_at	TROVE2	-0.708236667	0.550502435	TROVE2	TROVE2	TROVE2
212839_s_at	TROVE2	-0.892855	0.230227638	TROVE2	TROVE2	TROVE2
212852_s_at	TROVE2	-0.503776667	1.810509752	TROVE2	TROVE2	TROVE2
213027_at	TROVE2	-0.891877333	0	TROVE2	TROVE2	TROVE2
223436_s_at	TRPT1	0.689361333	2.499099453			
219632_s_at	TRPV1	1.144040167	0			
241606_s_at	TRUB1	0.790226	0.137080273			
209390_at	TSC1	0.652905667	0		TSC1	
204094_s_at	TSC22D2	0.8098285	0.137080273			
210953_at	TSC22D2	0.945605667	1.455154512			
210954_s_at	TSC22D2	0.544424333	0.796301331			
215547_at	TSC22D2	0.816461667	0.230227638			
208763_s_at	TSC22D3	0.672009333	1.810509752	TSC22D3		
225399_at	TSEN15	-0.511241667	1.810509752			
218132_s_at	TSEN34	-0.549048833	0.796301331	TSEN34		
241402_at	TSEN54	1.427876	0			
1555931_at	TSGA10	1.036281	3.701646186			
1555932_at	TSGA10	1.122880833	0.550502435			
220623_s_at	TSGA10	1.404409	0			
223392_s_at	TSHZ3	0.5981515	0			
209890_at	TSPAN5	0.55447	3.029425085			

Probe Set ID	Gene Symbol	miR-128-miRNC	q value (%)	miR-128 tgts_M	miR-128 tgts_TS	miR-128 tgts_PT
203504_s_at	ABCA1	-1.2890325	0	ABCA1	ABCA1	ABCA1
225387_at	TSPAN5	0.643407	1.810509752			
225388_at	TSPAN5	0.6991635	1.123589586			
209108_at	TSPAN6	-0.854464	2.499099453	TSPAN6		
239042_at	TSR1	0.593137333	3.701646186	TSR1		
225178_at	TTC14	0.9970595	0.393887585			
225180_at	TTC14	0.655706333	3.029425085			
231479_at	TTC33	1.013743667	0			
204822_at	TTK	-0.7019265	0.393887585			
228031_at	TTPAL	0.673631667	0.393887585			
201090_x_at	TUBA1B	-0.620266667	3.029425085			
212639_x_at	TUBA1B	-0.501973333	3.029425085			
213646_x_at	TUBA1B	-0.525411667	3.029425085			
209251_x_at	TUBA1C	-0.600918333	1.455154512			
211750_x_at	TUBA1C	-0.634113333	1.810509752			
208977_x_at	TUBB2C	-0.745258333	0.796301331			
213726_x_at	TUBB2C	-0.701856667	0.796301331			
202154_x_at	TUBB3	-0.660278333	1.455154512			
213476_x_at	TUBB3	-0.795851667	0.230227638			
221326_s_at	TUBD1	0.9382945	1.455154512			
230891_at	TUBE1	0.5997825	3.701646186			
201714_at	TUBG1	-0.628452833	2.499099453			
1554086_at	TUBGCP3	1.110383667	0			
228397_at	TUG1	-0.813692833	1.123589586			
1554163_at	TWIST2	0.577510833	1.455154512			
226784_at	TWISTNB	-0.591036833	1.455154512	TWISTNB		
209077_at	TXN2	-0.593528333	1.455154512			
220495_s_at	TXNDC15	0.501748333	1.810509752			
226747_at	TXNDC16	0.860187	1.455154512			
228743_at	TXNDC17	0.510192833	3.701646186			
1554047_at	TXNDC9	0.879973167	3.701646186			
201008_s_at	TXNIP	-1.9244995	0	TXNIP	TXNIP	
201009_s_at	TXNIP	-2.001359833	0	TXNIP	TXNIP	
201010_s_at	TXNIP	-2.0323135	0	TXNIP	TXNIP	
218347_at	TYW1	-0.747897167	0	TYW1		
1558356_at	UACA	-0.570533667	2.499099453			
223279_s_at	UACA	-0.5531575	1.810509752			
236715_x_at	UACA	-0.686389167	0			
238868_at	UACA	-1.484533667	0			
228353_x_at	UBASH3B	0.903019333	1.455154512			
238462_at	UBASH3B	0.842356667	3.029425085			
211764_s_at	UBE2D1	-1.140967	0.230227638	UBE2D1		
214590_s_at	UBE2D1	-1.13788	0.137080273	UBE2D1		
218837_s_at	UBE2D4	1.083613833	0			
225651_at	UBE2E2	-1.126534167	0.393887585	UBE2E2	UBE2E2	UBE2E2
226005_at	UBE2G1	0.759159833	0			
225179_at	UBE2K	-0.707348333	0.230227638	UBE2K	UBE2K	
200676_s_at	UBE2L3	0.686896	0.796301331	UBE2L3		
200684_s_at	UBE2L3	0.620913833	3.701646186	UBE2L3		
201523_x_at	UBE2N	-0.511350833	1.810509752	UBE2N	UBE2N	UBE2N
201524_x_at	UBE2N	-0.908216333	0.05396551	UBE2N	UBE2N	UBE2N

Probe Set ID	Gene Symbol	miR-128-miRNC	q value (%)	miR-128 tgts_M	miR-128 tgts_TS	miR-128 tgts_PT
203504_s_at	ABCA1	-1.2890325	0	ABCA1	ABCA1	ABCA1
202779_s_at	UBE2S	-1.023673667	3.701646186			
212404_s_at	UBE3B	0.614685	2.499099453			
227914_s_at	UBE3B	0.673489667	2.499099453			
1554794_a_at	UBE3C	0.555714833	1.455154512	UBE3C		
225445_at	UBN2	0.940962	0.393887585	UBN2		
238349_at	UBN2	0.860780333	0.796301331	UBN2		
238350_at	UBN2	0.862886833	0.550502435	UBN2		
226921_at	UBR1	-0.612165	1.123589586	UBR1	UBR1	
212756_s_at	UBR2	0.598286667	0.796301331			
230029_x_at	UBR3	1.188227833	0.393887585			
234982_at	UBR3	0.838677667	0.550502435			
244121_at	UBR3	0.9483185	0			
214881_s_at	UBTF	0.607596	1.810509752	UBTF		
212934_at	UBXN2B	0.503589167	2.499099453			
215983_s_at	UBXN8	0.973906	0			
227919_at	UCA1	-0.99319	0			
208997_s_at	UCP2	-1.2851575	0			
218190_s_at	UCRC	-0.783818167	0.796301331			
1552656_s_at	UHMK1	1.04659	0			
227740_at	UHMK1	0.820706667	0			
235003_at	UHMK1	0.804786667	0			
1554292_a_at	UHRF1BP1L	0.698273333	0.796301331			
221291_at	ULBP2	0.7267855	2.499099453			
238542_at	ULBP2	0.8159665	0.796301331			
202893_at	UNC13B	0.6776495	2.499099453			
1560320_a_at	UNQ2963	0.758240333	1.455154512			
217935_s_at	UQCC	-0.823454	0	UQCC		
202090_s_at	UQCR	-0.610699167	1.123589586			
222616_s_at	USP16	0.508651667	1.455154512			
226357_at	USP19	0.708962167	1.810509752			
207213_s_at	USP2	1.095245167	0	USP2		
218367_x_at	USP21	0.532846333	3.029425085			
232219_x_at	USP21	0.6359385	1.455154512			
234735_s_at	USP21	0.585067	1.123589586			
230004_at	USP24	-0.556895667	3.029425085			
226033_at	USP31	0.834871	0.137080273			
226035_at	USP31	0.897176333	0			
227256_at	USP31	0.588958333	0.796301331			
212066_s_at	USP34	-0.599973333	1.455154512	USP34		
232033_at	USP37	1.083132	0.550502435			
237439_at	USP43	0.811389167	3.701646186			
203870_at	USP46	0.657796	1.810509752		USP46	USP46
225925_s_at	USP48	0.695828	0.393887585			
230083_at	USP53	0.941820833	0			
231817_at	USP53	0.850071667	2.499099453			
221098_x_at	UTP14A	0.689121833	1.455154512			
203614_at	UTP14C	0.933465333	1.455154512			
238561_s_at	UTP23	0.6452475	3.029425085			
218495_at	UXT	0.525262333	3.029425085			
213326_at	VAMP1	0.544537833	2.499099453	VAMP1		

Probe Set ID	Gene Symbol	miR-128-miRNC	q value (%)	miR-128 tgts_M	miR-128 tgts_TS	miR-128 tgts_PT
203504_s_at	ABCA1	-1.2890325	0	ABCA1	ABCA1	ABCA1
219330_at	VANGL1	-0.637755167	0.137080273		VANGL1	
229997_at	VANGL1	-0.551715167	0.05396551		VANGL1	
225198_at	VAPA	-0.627839833	0.230227638			
201796_s_at	VARS	0.609038833	2.499099453			
224221_s_at	VAV3	2.711480167	0	VAV3	VAV3	VAV3
201472_at	VBP1	-0.820931333	0.393887585			
204620_s_at	VCAN	-0.592344833	1.455154512	VCAN		
211571_s_at	VCAN	-0.68537	0.550502435	VCAN		
215646_s_at	VCAN	-0.623589	0.796301331	VCAN		
221731_x_at	VCAN	-0.543758167	3.029425085	VCAN		
204254_s_at	VDR	1.716133667	0			
204255_s_at	VDR	1.307296667	1.810509752			
213692_s_at	VDR	1.408017833	1.455154512			
210512_s_at	VEGFA	1.104937667	0.230227638			
211527_x_at	VEGFA	0.713186	0			
229760_at	VEPH1	-0.6281685	0.796301331			
1553679_s_at	VKORC1L1	0.603451833	1.455154512			
209822_s_at	VLDLR	0.711660667	0.550502435	VLDLR		
1558549_s_at	VNN1	1.805833333	0.05396551			
227988_s_at	VPS13A	0.5737205	1.810509752	VPS13A		
1560060_s_at	VPS37C	0.7965195	0.393887585			
219053_s_at	VPS37C	0.714029833	1.123589586			
222627_at	VPS54	0.634943333	0.796301331			
203856_at	VRK1	-0.546727	1.455154512			
235818_at	VSTM1	0.740603167	0			
1562226_at	VWDE	0.501142833	1.123589586			
221725_at	WASF2	0.613088333	0.796301331			
224562_at	WASF2	0.575632667	3.029425085			
224563_at	WASF2	0.606848333	1.123589586			
205810_s_at	WASL	0.651455	2.499099453	WASL		
224800_at	WDFY1	-0.505709833	2.499099453	WDFY1		
240282_at	WDR1	1.3004795	2.499099453	WDR1		
1552430_at	WDR17	1.014875667	3.029425085	WDR17		
220917_s_at	WDR19	0.5199215	2.499099453	WDR19		
224897_at	WDR26	0.539098333	1.810509752	WDR26		
229141_at	WDR33	-1.179800167	3.029425085			
243832_at	WDR33	-0.7723765	1.455154512			
203855_at	WDR47	0.737884333	0.796301331	WDR47		
228295_at	WDR59	0.662339667	0.230227638	WDR59		
230612_at	WDR73	1.035735833	2.499099453			
235025_at	WDR89	0.8324365	0.550502435			
244038_at	WDR89	0.542373333	1.455154512			
212533_at	WEE1	-0.9728195	0.137080273	WEE1		WEE1
1557261_at	WHAMML1 /// WHAMML2	1.242827333	0			
202663_at	WIPF1	0.594253333	2.499099453			
202665_s_at	WIPF1	0.516901667	2.499099453			
213836_s_at	WIPI1	0.684744667	2.499099453			
202031_s_at	WIPI2	-0.551663833	3.029425085			

Probe Set ID	Gene Symbol	miR-128-miRNC	q value (%)	miR-128 tgts_M	miR-128 tgts_TS	miR-128 tgts_PT
203504_s_at	ABCA1	-1.2890325	0	ABCA1	ABCA1	ABCA1
204710_s_at	WIPI2	-0.723323167	3.029425085			
211994_at	WNK1	-0.641405	1.455154512	WNK1	WNK1	
210248_at	WNT7A	0.780351167	2.499099453			
222489_s_at	WRNIP1	0.562741	0.230227638			
213734_at	WSB2	0.649616667	0.550502435			
229630_s_at	WTAP	-0.564125	1.810509752	WTAP		WTAP
216074_x_at	WWC1	0.588408333	1.455154512			
1554762_a_at	WWC2	0.7482225	1.123589586			
218775_s_at	WWC2	0.656495	3.029425085			
225273_at	WWC3	0.594894	3.701646186			
1552737_s_at	WWP2	0.788207667	0	WWP2		
206536_s_at	XIAP	0.795924333	1.123589586			
225859_at	XIAP	0.719897	3.029425085			
1557436_at	XKR6	-0.566865	0.796301331			
205672_at	XPA	-0.630353333	2.499099453			
223055_s_at	XPO5	0.59546	1.455154512			
223057_s_at	XPO5	0.7299345	0.230227638			
203655_at	XRCC1	0.58898	1.455154512			
208318_s_at	XYLB	1.392730667	0			
216338_s_at	YIPF3	0.608933167	1.455154512			
212341_at	YIPF6	0.579288333	1.455154512			
215150_at	YOD1	0.814630333	0.550502435			
227309_at	YOD1	0.617484667	0.550502435			
227020_at	YPEL2	0.783524667	0	YPEL2	YPEL2	
229060_at	YPEL2	0.6872345	0.137080273	YPEL2	YPEL2	
222430_s_at	YTHDF2	0.501220333	0.393887585			
212426_s_at	YWHAQ	-0.63261	1.455154512	YWHAQ		
238613_at	ZAK	0.616726667	0.393887585			
213376_at	ZBTB1	0.654955167	0.550502435			
244581_at	ZBTB20	-1.030336167	1.123589586			
205340_at	ZBTB24	0.659062167	0.550502435	ZBTB24		
227111_at	ZBTB34	0.6922225	0.796301331		ZBTB34	
205256_at	ZBTB39	0.940516833	1.810509752		ZBTB39	
204181_s_at	ZBTB43	0.672000167	1.123589586			
204182_s_at	ZBTB43	0.828084167	3.701646186			
227991_x_at	ZBTB43	0.632332833	2.499099453			
1554469_at	ZBTB44	0.831187667	0.393887585	ZBTB44		
226554_at	ZBTB7A	0.593588333	0.550502435			
227809_at	ZC3H6	0.986935333	1.810509752			
241372_at	ZC3H6	0.836259333	0.796301331			
221193_s_at	ZCCHC10	0.6608775	1.810509752			
219062_s_at	ZCCHC2	0.999112833	0	ZCCHC2		
222816_s_at	ZCCHC2	1.696921667	0	ZCCHC2		
220933_s_at	ZCCHC6	0.690483	0.796301331			
236155_at	ZCCHC6	0.551695	1.810509752			
242776_at	ZCCHC6	0.69646	0			
243863_at	ZCWPW2	0.554588167	3.701646186			
243550_at	ZDHHC21	0.653324833	3.029425085			
226912_at	ZDHHC23	1.569156	0			
202456_s_at	ZER1	0.753495167	1.455154512			

Probe Set ID	Gene Symbol	miR-128-miRNC	q value (%)	miR-128 tgts_M	miR-128 tgts_TS	miR-128 tgts_PT
203504_s_at	ABCA1	-1.2890325	0	ABCA1	ABCA1	ABCA1
230358_at	ZER1	0.7053155	3.029425085			
219779_at	ZFHX4	0.609062333	1.123589586		ZFHX4	ZFHX4
222407_s_at	ZFP106	1.072288667	0.137080273			
231943_at	ZFP28	1.028413333	1.123589586			
234300_s_at	ZFP28	1.125138333	0			
234937_x_at	ZFP28	1.015445667	0			
240565_at	ZFP28	1.189270667	0			
211962_s_at	ZFP36L1	0.55436	1.455154512	ZFP36L1	ZFP36L1	
218968_s_at	ZFP64	0.947905167	0	ZFP64		
213073_at	ZFYVE26	0.787975333	0.550502435			
37943_at	ZFYVE26	0.844936667	0.550502435			
225218_at	ZFYVE27	0.667442333	2.499099453			
203556_at	ZHX2	1.322285333	0			
214670_at	ZKSCAN1	0.754974833	1.455154512	ZKSCAN1		
226344_at	ZMAT1	0.528087833	2.499099453			
224782_at	ZMAT2	-0.529036667	0.550502435			
221924_at	ZMIZ2	0.599678167	0.137080273	ZMIZ2		
210282_at	ZMYM2	-1.075613333	0.137080273			
1552970_s_at	ZMYM6	0.7626815	1.455154512			
1561892_at	ZMYM6	0.732348333	0.393887585			
219925_at	ZMYM6	0.938075	0			
227594_at	ZMYM6	0.8488685	0.796301331			
227595_at	ZMYM6	0.777646167	0.05396551			
230821_at	ZNF148	0.555354667	3.029425085			
213452_at	ZNF184	0.700451167	3.701646186			
223792_at	ZNF2	0.877716	1.123589586			
204327_s_at	ZNF202	0.605548333	1.455154512			
239937_at	ZNF207	1.046833	1.810509752			
207125_at	ZNF225	0.771634167	0.230227638			
227689_at	ZNF227	0.528229667	3.701646186			
219123_at	ZNF232	0.576597333	3.029425085			
242953_at	ZNF234	0.674824167	1.123589586			
1554045_at	ZNF24	1.081589833	0		ZNF24	
206862_at	ZNF254	0.796424333	1.810509752	ZNF254		
1558700_s_at	ZNF260	0.822571333	0.230227638			
228920_at	ZNF260	0.690647833	0.393887585			
205917_at	ZNF264	0.726886833	0.796301331			
230063_at	ZNF264	0.607661167	3.029425085			
225383_at	ZNF275	0.529587167	0.230227638			
1557684_at	ZNF286A	0.659974667	0	ZNF286A		
203520_s_at	ZNF318	0.660320167	0.550502435			
241704_x_at	ZNF320	0.564635667	2.499099453			
228711_at	ZNF37A	0.701060333	0.393887585			
212369_at	ZNF384	0.606003333	3.029425085			
220616_at	ZNF384	0.572694	3.701646186			
228927_at	ZNF397	0.852749167	0	ZNF397		
227768_at	ZNF407	0.524374833	3.029425085			
209944_at	ZNF410	0.714037333	2.499099453			
206810_at	ZNF44	0.932830833	3.701646186			
241066_at	ZNF449	0.8750665	0.230227638	ZNF449		

Probe Set ID	Gene Symbol	miR-128-miRNC	q value (%)	miR-128 tgts_M	miR-128 tgts_TS	miR-128 tgts_PT
203504_s_at	ABCA1	-1.2890325	0	ABCA1	ABCA1	ABCA1
234921_at	ZNF470	0.874494167	1.455154512	ZNF470		
213124_at	ZNF473	0.5141015	0.137080273			
226676_at	ZNF521	-0.511726	0.550502435			
226677_at	ZNF521	-0.520586	1.455154512			
228305_at	ZNF565	0.609512333	1.123589586			
204473_s_at	ZNF592	0.547934667	3.029425085			
230542_at	ZNF597	0.7100525	1.123589586			
242463_x_at	ZNF600	0.907596833	0.393887585			
212617_at	ZNF609	0.786883	0.393887585			
241499_at	ZNF621	0.554462333	1.455154512			
226509_at	ZNF641	1.624502667	0			
235179_at	ZNF641	1.501945167	0			
207219_at	ZNF643	1.1623555	0			
1553702_at	ZNF697	1.203209	0			
227080_at	ZNF697	1.165404333	0			
39891_at	ZNF710	0.784940833	0			
238510_at	ZNF720	0.587428	3.701646186	ZNF720		
1558277_at	ZNF740	1.157823667	0.230227638			
238687_x_at	ZNF770	0.588467	1.810509752			
215570_s_at	ZNF780A /// ZNF780B	0.55669	2.499099453			
214899_at	ZNF780B	0.7489	3.029425085			
239083_at	ZNF786	0.760706167	1.123589586			
214138_at	ZNF79	0.699032	0.230227638			
238436_s_at	ZNF805	0.766011833	1.810509752			
238437_at	ZNF805	0.5952785	1.810509752			
228046_at	ZNF827	0.566657333	1.123589586	ZNF827	ZNF827	ZNF827
243617_at	ZNF827	0.634734667	3.701646186	ZNF827	ZNF827	ZNF827
221645_s_at	ZNF83	0.546327667	1.455154512			
1564662_at	ZNF852	0.790391833	0.550502435			
213444_at	ZNF862	1.181195667	0.05396551			
226808_at	ZNF862	1.258181	0			
1564962_at	ZNF92	0.670812	1.810509752			
1564963_x_at	ZNF92	0.706909167	2.499099453			
218932_at	ZNHIT6	-0.700472667	0.137080273	ZNHIT6		
223639_s_at	ZNRD1	0.679632667	1.810509752			
225131_at	ZRANB1	0.64868	0.393887585			
225138_at	ZRANB1	0.549817333	1.810509752			
241345_at	ZRANB2	1.095827333	3.029425085			
228769_at	ZSCAN22	1.189661667	0			
1564039_at	ZSCAN23	0.780899167	0			
204026_s_at	ZWINT	-0.948288667	0.230227638			
215263_at	ZXDA /// ZXDB	0.603341	0			
216013_at	ZXDB	0.53733	1.123589586			
228005_at	ZXDB	0.521978833	3.701646186			
230106_at	ZXDC	0.502503667	3.029425085	ZXDC		
230209_at	ZXDC	1.582206	1.810509752	ZXDC		
234991_at	ZXDC	0.845447667	1.455154512	ZXDC		

Supplementary Table 3. Differentially expressed genes in siRNA transfected HEY cells. Significantly differentially expressed genes (fold change ≥1.4, FDR ≤5%) following EGFR siRNA transfection, compared to siNC transfection of HEY cells. 'Probeset ID' refers to Affymetrix HG-U133 Plus 2.0 probeset identifier. 'Gene Symbol' shows the official gene symbol for the corresponding Probeset ID. 'siRNA-siNC' refers to the difference between average log2 signal values of siRNA transfected cells and the siNC transfected cells. 'q-value (%)' shows the false discovery rate calculated using the SAM algorithm. All transfections were carried out in triplicates.

Probeset ID	Gene Symbol	siRNA-siNC	q-value (%)
201110_s_at	THBS1	1.15	3.43
201109_s_at	THBS1	1.06	3.43
202310_s_at	COL1A1	0.87	4.02
201108_s_at	THBS1	0.76	3.43
207950_s_at	ANK3	0.75	3.43
203139_at	DAPK1	0.57	3.43
231618_s_at	SUNC1	-0.82	0.00
229672_at	UQCC	-0.96	0.00
201983_s_at	EGFR	-1.04	0.00
208885_at	LCP1	-1.04	0.00
211607_x_at	EGFR	-1.15	0.00
210984_x_at	EGFR	-1.17	0.00
221786_at	C6orf120	-1.26	0.00

Supplementary Table 4. miRNA target enrichment analysis among up-regulated genes following miR-128 transfection. Genomica enrichment (FDR <0.05) of miRNA targets among significantly up-regulated genes following miR-128 transfection into HEY cells. Regulating miRNAs are predicted using miRanda (those with the prefix John04 or SloanKettering), TargetScan or PicTar. Only "up" genes, i.e. up-regulated in the transfection, are analyzed ('Set'). The other columns show the p-value of enrichment, the number of probesets in our dataset that are up-regulated and present in Genomica database ('Set Size'), compared to the number of probesets in each set in the background Affymetrix chip ('Total Size'). If a probeset is a predicted target of a miRNA then it is considered a 'hit' ('Set Hits' and 'Total Hits'). The percentages calculated from raw numbers of genes that belong in each set are also shown.

Set	Enriched Set	Enriched Value	P-value	Set Hits	Set Size	Set Hits (%)	Total Hits	Total Size	Total Hits (%)
Up	John04_hsa-miR-191	1	1.32E-08	36	2148	1.68	253	43407	0.59
Up	John04_hsa-miR-363	1	2.62E-04	36	2148	1.68	389	43407	0.9
Up	Targetscan_hsa-miR-409-5p	1	3.07E-03	28	2148	1.31	323	43407	0.75
Up	SloanKettering_hsa-miR-20	1	1.93E-07	44	2148	2.05	381	43407	0.88
Up	SloanKettering_hsa-miR-17_5p	1	1.27E-05	34	2148	1.59	309	43407	0.72
Up	John04_hsa-miR-106a	1	2.06E-03	43	2148	2.01	547	43407	1.27

Set	Enriched Set	Enriched Value	P-value	Set Hits	Set Size	Set Hits (%)	Total Hits	Total Size	Total Hits (%)
Up	John04_hsa-miR-148	1	3.83E-03	67	2148	3.12	968	43407	2.24
Up	John04_hsa-miR-18	1	7.64E-09	87	2148	4.06	921	43407	2.13
Up	John04_hsa-miR-183	1	2.48E-03	60	2148	2.8	832	43407	1.92
Up	John04_hsa-miR-93	1	6.30E-05	56	2148	2.61	657	43407	1.52
Up	John04_mmu-miR-148a	1	3.83E-03	67	2148	3.12	968	43407	2.24
Up	John04_mmu-miR-291_3p	1	7.17E-04	66	2148	3.08	887	43407	2.05
Up	John04_mmu-miR-300	1	2.84E-03	61	2148	2.84	854	43407	1.97
Up	Pictar_hsa-miR-183	1	1.20E-03	64	2148	2.98	872	43407	2.01
Up	Pictar_hsa-miR-33	1	7.07E-03	57	2148	2.66	823	43407	1.9
Up	Pictar_hsa-miR-145	1	2.21E-05	71	2148	3.31	862	43407	1.99
Up	Targetscan_hsa-miR-183	1	4.05E-04	66	2148	3.08	868	43407	2
Up	Targetscan_hsa-miR-199	1	2.16E-04	64	2148	2.98	816	43407	1.88
Up	Targetscan_hsa-miR-324-3p	1	4.00E-04	42	2148	1.96	488	43407	1.13
Up	Targetscan_hsa-miR-33	1	1.88E-03	55	2148	2.57	739	43407	1.71
Up	John04_hsa-miR-145	1	1.84E-04	58	2148	2.71	716	43407	1.65

Set	Enriched Set	Enriched Value	P-value	Set Hits	Set Size	Set Hits (%)	Total Hits	Total Size	Total Hits (%)
Up	John04_hsa-miR-30d	1	5.69E-04	68	2148	3.17	912	43407	2.11
Up	Pictar_hsa-miR-122a	1	8.67E-04	32	2148	1.49	355	43407	0.82
Up	Targetscan_hsa-miR-122a	1	1.29E-03	34	2148	1.59	394	43407	0.91
Up	Targetscan_hsa-miR-299	1	1.87E-04	56	2148	2.61	685	43407	1.58
Up	Targetscan_hsa-miR-378	1	1.97E-04	71	2148	3.31	926	43407	2.14
Up	John04_hsa-let-7a	1	1.71E-04	63	2148	2.94	793	43407	1.83
Up	John04_hsa-let-7c	1	5.12E-03	53	2148	2.47	742	43407	1.71
Up	John04_hsa-let-7d	1	1.31E-04	65	2148	3.03	817	43407	1.89
Up	John04_hsa-let-7e	1	6.60E-03	52	2148	2.43	735	43407	1.7
Up	John04_hsa-let-7f	1	8.98E-08	73	2148	3.4	767	43407	1.77
Up	John04_hsa-let-7g	1	1.50E-04	67	2148	3.12	853	43407	1.97
Up	John04_hsa-let-7i	1	4.47E-04	61	2148	2.84	790	43407	1.82
Up	John04_hsa-miR-221	1	3.72E-04	38	2148	1.77	426	43407	0.99
Up	John04_hsa-miR-98	1	6.58E-06	66	2148	3.08	755	43407	1.74
Up	John04_rno-miR-327	1	5.94E-04	45	2148	2.1	544	43407	1.26

Set	Enriched Set	Enriched Value	P-value	Set Hits	Set Size	Set Hits (%)	Total Hits	Total Size	Total Hits (%)
Up	John04_hsa-miR-29c	1	4.14E-03	62	2148	2.89	886	43407	2.05
Up	John04_mmu-miR-291_5p	1	6.92E-04	57	2148	2.66	739	43407	1.71
Up	Targetscan_hsa-miR-219	1	6.13E-03	54	2148	2.52	766	43407	1.77
Up	John04_hsa-miR-30c	1	8.29E-03	59	2148	2.75	864	43407	2
Up	John04_hsa-miR-30e	1	6.78E-03	66	2148	3.08	976	43407	2.25
Up	Pictar_hsa-miR-205	1	2.92E-04	62	2148	2.89	793	43407	1.83
Up	Targetscan_hsa-miR-18	1	1.30E-03	42	2148	1.96	518	43407	1.2
Up	Targetscan_hsa-miR-205	1	4.48E-07	72	2148	3.36	785	43407	1.81
Up	Targetscan_hsa-miR-486	1	1.32E-04	31	2148	1.45	305	43407	0.71
Up	John04_hsa-miR-30b	1	7.86E-03	51	2148	2.38	725	43407	1.68
Up	Targetscan_hsa-miR-485-5p	1	1.40E-04	45	2148	2.1	509	43407	1.18
Up	Targetscan_hsa-miR-140	1	8.18E-03	38	2148	1.77	509	43407	1.18
Up	John04_hsa-miR-208	1	4.23E-03	26	2148	1.22	300	43407	0.7
Up	Targetscan_hsa-miR-329	1	3.03E-03	52	2148	2.43	706	43407	1.63
Up	SloanKettering_hsa-miR-135	1	3.07E-03	15	2148	0.7	136	43407	0.32

Set	Enriched Set	Enriched Value	P-value	Set Hits	Set Size	Set Hits (%)	Total Hits	Total Size	Total Hits (%)
Up	John04_rno-miR-345	1	3.10E-03	19	2148	0.89	191	43407	0.45
Up	Pictar_hsa-miR-150	1	1.35E-04	53	2148	2.47	630	43407	1.46
Up	Pictar_hsa-miR-185	1	1.42E-03	62	2148	2.89	845	43407	1.95
Up	Targetscan_hsa-miR-136	1	5.89E-03	31	2148	1.45	386	43407	0.89
Up	Targetscan_hsa-miR-210	1	7.84E-04	8	2148	0.38	41	43407	0.1
Up	SloanKettering_hsa-miR-181a	1	5.31E-03	16	2148	0.75	158	43407	0.37
Up	Pictar_hsa-miR-21	1	4.97E-06	50	2148	2.33	514	43407	1.19
Up	Targetscan_hsa-miR-21	1	9.77E-05	49	2148	2.29	561	43407	1.3
Up	Targetscan_hsa-miR-503	1	9.91E-08	80	2148	3.73	871	43407	2.01
Up	Pictar_hsa-miR-30a-3p	1	7.84E-03	54	2148	2.52	776	43407	1.79
Up	Pictar_hsa-miR-30e-3p	1	6.77E-03	54	2148	2.52	770	43407	1.78
Up	Targetscan_hsa-miR-30-3p	1	5.30E-04	71	2148	3.31	959	43407	2.21
Up	Pictar_hsa-miR-146b	1	1.06E-07	44	2148	2.05	373	43407	0.86
Up	Pictar_hsa-miR-146a	1	5.50E-08	46	2148	2.15	390	43407	0.9
Up	Targetscan_hsa-miR-146	1	2.61E-09	49	2148	2.29	390	43407	0.9

Set	Enriched Set	Enriched Value	P-value	Set Hits	Set Size	Set Hits (%)	Total Hits	Total Size	Total Hits (%)
Up	Targetscan_hsa-miR-377	1	1.29E-03	63	2148	2.94	858	43407	1.98
Up	John04_hsa-miR-137	1	5.65E-03	41	2148	1.91	546	43407	1.26
Up	Pictar_hsa-miR-335	1	5.03E-04	40	2148	1.87	463	43407	1.07
Up	Targetscan_hsa-miR-335	1	2.45E-05	46	2148	2.15	487	43407	1.13
Up	Targetscan_hsa-miR-193	1	3.30E-03	36	2148	1.68	449	43407	1.04
Up	SloanKettering_hsa-miR-145	1	7.50E-07	15	2148	0.7	67	43407	0.16
Up	Pictar_hsa-miR-18a	1	8.24E-04	42	2148	1.96	506	43407	1.17
Up	Pictar_hsa-miR-18b	1	2.06E-03	41	2148	1.91	515	43407	1.19
Up	Targetscan_hsa-miR-139	1	1.03E-03	54	2148	2.52	703	43407	1.62
Up	SloanKettering_hsa-miR-19a	1	2.96E-03	26	2148	1.22	292	43407	0.68
Up	SloanKettering_hsa-miR-1	1	7.19E-03	15	2148	0.7	149	43407	0.35
Up	SloanKettering_hsa-miR-19b	1	1.11E-05	30	2148	1.4	255	43407	0.59
Up	SloanKettering_hsa-miR-206	1	2.30E-03	15	2148	0.7	132	43407	0.31
Up	John04_hsa-miR-194	1	8.34E-05	56	2148	2.61	664	43407	1.53
Up	John04_hsa-miR-205	1	1.35E-03	42	2148	1.96	519	43407	1.2

Set	Enriched Set	Enriched Value	P-value	Set Hits	Set Size	Set Hits (%)	Total Hits	Total Size	Total Hits (%)
Up	John04_mmu-miR-290	1	1.80E-03	44	2148	2.05	559	43407	1.29
Up	Pictar_hsa-miR-221	1	6.66E-04	58	2148	2.71	754	43407	1.74
Up	Pictar_hsa-miR-222	1	1.64E-03	54	2148	2.52	718	43407	1.66
Up	Pictar_hsa-miR-187	1	2.74E-03	7	2148	0.33	39	43407	0.09
Up	Targetscan_hsa-miR-191	1	1.58E-03	13	2148	0.61	102	43407	0.24
Up	SloanKettering_hsa-miR-26a	1	3.30E-04	21	2148	0.98	184	43407	0.43
Up	John04_hsa-miR-150	1	6.16E-03	32	2148	1.49	403	43407	0.93
Up	John04_hsa-miR-26a	1	1.48E-06	83	2148	3.87	978	43407	2.26
Up	John04_hsa-miR-26b	1	2.57E-06	83	2148	3.87	992	43407	2.29
Up	Targetscan_hsa-miR-485-3p	1	6.37E-03	41	2148	1.91	550	43407	1.27
Up	Targetscan_hsa-miR-192	1	5.19E-07	39	2148	1.82	330	43407	0.77
Up	Pictar_hsa-miR-22	1	1.50E-03	65	2148	3.03	897	43407	2.07
Up	Targetscan_hsa-miR-542-3p	1	8.50E-05	51	2148	2.38	588	43407	1.36
Up	Targetscan_hsa-miR-452	1	1.78E-03	41	2148	1.91	511	43407	1.18
Up	Targetscan_hsa-miR-221	1	9.92E-04	55	2148	2.57	718	43407	1.66

Set	Enriched Set	Enriched Value	P-value	Set Hits	Set Size	Set Hits (%)	Total Hits	Total Size	Total Hits (%)
Up	SloanKettering_ hsa-miR-9	1	6.76E-03	21	2148	0.98	235	43407	0.55
Up	SloanKettering_ hsa-miR-150	1	6.06E-03	8	2148	0.38	56	43407	0.13
Up	SloanKettering_ hsa-miR-15a	1	3.07E-03	20	2148	0.94	205	43407	0.48
Up	SloanKettering_ hsa-miR-15b	1	1.72E-03	21	2148	0.98	209	43407	0.49
Up	John04_hsa-miR-23b	1	3.16E-03	66	2148	3.08	943	43407	2.18
Up	SloanKettering_ hsa-miR-29c	1	3.48E-03	19	2148	0.89	193	43407	0.45
Up	SloanKettering_ hsa-let-7f	1	1.62E-05	19	2148	0.89	127	43407	0.3
Up	SloanKettering_ hsa-miR-29a	1	1.08E-03	14	2148	0.66	110	43407	0.26
Up	Pictar_hsa-miR-217	1	7.97E-04	49	2148	2.29	615	43407	1.42
Up	SloanKettering_ hsa-let-7e	1	2.68E-03	14	2148	0.66	121	43407	0.28
Up	SloanKettering_ hsa-let-7g	1	5.02E-05	18	2148	0.84	126	43407	0.3
Up	Targetscan_hsa-miR-189	1	2.31E-04	27	2148	1.26	259	43407	0.6
Up	SloanKettering_ hsa-let-7a	1	9.30E-04	17	2148	0.8	146	43407	0.34
Up	Pictar_hsa-miR-302a-star	1	9.57E-04	15	2148	0.7	121	43407	0.28
Up	John04_mmu-miR-292_3p	1	9.28E-04	21	2148	0.98	199	43407	0.46

Set	Enriched Set	Enriched Value	P-value	Set Hits	Set Size	Set Hits (%)	Total Hits	Total Size	Total Hits (%)
Up	SloanKettering_hsa-miR-7	1	1.20E-03	13	2148	0.61	99	43407	0.23
Up	SloanKettering_hsa-miR-363	1	9.07E-04	6	2148	0.28	24	43407	0.06
Up	SloanKettering_rno-miR-352	1	5.10E-03	6	2148	0.28	33	43407	0.08
Up	SloanKettering_hsa-miR-221	1	1.85E-05	12	2148	0.56	57	43407	0.14

Supplementary Table 5. Microarray expression levels of pre-transfected HEY cell miRNAs. Signal values from Affymetrix microarray analysis of miRNA expression from pre-transfected HEY cells. 'Probeset name' refers to the unique Affymetrix identifier for sequences on the chip. The log_2 transformed 'signal' values for each probeset ('Hey cell Signal_1' and 'Hey cell Signal_2') and the p-value of detection ['p-value (Hey cell Signal_1)' and 'p-value (Hey cell Signal_2)'] is also shown. Signal values with Wilcoxon $p < 0.06$ of detection are called "TRUE" [under 'Detection (Hey cell Signal_1)' and 'Detection (Hey cell Signal_2)']. The 'mean signal' column shows the average of the log_2 signal values from the 2 'Hey cell Signal (_1 and _2)' columns. Data shown are for human miRNAs (with hsa- prefix) only.

ProbeSet Name	Hey cell Signal_1	p-value (Hey cell Signal_1)	Detection (Hey cell Signal_1)	Hey cell Signal_2	p-value (Hey cell Signal_2)	Detection (Hey cell Signal_2)	Mean signal
hsa-let-7a_st	13.25778	2.05E-08	TRUE	13.14588	2.05E-08	TRUE	13.20183
hsa-let-7a-star_st	4.058473	0.6218237	FALSE	4.199147	0.3329969	FALSE	4.12881
hsa-let-7b_st	13.14117	2.05E-08	TRUE	13.0817	2.05E-08	TRUE	13.111435
hsa-let-7b-star_st	4.409339	0.137066	FALSE	4.378109	0.1409812	FALSE	4.393724
hsa-let-7c_st	12.93934	2.05E-08	TRUE	12.91432	2.05E-08	TRUE	12.92683
hsa-let-7c-star_st	4.030557	0.549224	FALSE	4.071347	0.5779037	FALSE	4.050952
hsa-let-7d_st	12.64366	2.05E-08	TRUE	12.59471	2.05E-08	TRUE	12.619185
hsa-let-7d-star_st	5.527161	0.001760183	TRUE	5.330665	0.004321587	TRUE	5.428913
hsa-let-7e_st	11.43213	2.05E-08	TRUE	11.57724	2.05E-08	TRUE	11.504685
hsa-let-7e-star_st	5.427573	0.01135333	TRUE	5.291793	0.009632347	TRUE	5.359683
hsa-let-7f_st	11.3144	2.13E-08	TRUE	10.91715	2.13E-08	TRUE	11.115775
hsa-let-7f-1-star_st	4.35369	0.245166	FALSE	4.324418	0.2616047	FALSE	4.339054
hsa-let-7f-2-star_st	3.810567	0.9163727	FALSE	3.728485	0.9709982	FALSE	3.769526
hsa-let-7g_st	11.06676	2.05E-08	TRUE	10.47923	2.05E-08	TRUE	10.772995
hsa-let-7g-star_st	4.645726	0.170433	FALSE	5.018413	0.05029794	TRUE	4.8320695
hsa-let-7i_st	12.87767	2.05E-08	TRUE	12.69815	2.05E-08	TRUE	12.78791
hsa-let-7i-star_st	4.897941	0.03327993	TRUE	4.186187	0.3906482	FALSE	4.542064
hsa-miR-1_st	3.919921	0.5825235	FALSE	3.746041	0.888293	FALSE	3.832981
hsa-miR-100_st	14.07139	2.05E-08	TRUE	14.09498	2.05E-08	TRUE	14.083185
hsa-miR-100-star_st	6.260762	3.52E-06	TRUE	5.568634	0.001586907	TRUE	5.914698
hsa-miR-101_st	3.870145	0.7124807	FALSE	3.967698	0.4037512	FALSE	3.9189215
hsa-miR-101-star_st	3.888543	0.5564451	FALSE	3.874936	0.7983921	FALSE	3.8817395

ProbeSet Name	Hey cell Signal_1	p-value (Hey cell Signal_1)	Detection (Hey cell Signal_1)	Hey cell Signal_2	p-value (Hey cell Signal_2)	Detection (Hey cell Signal_2)	Mean signal
hsa-miR-103_st	13.03601	2.05E-08	TRUE	13.16777	2.05E-08	TRUE	13.10189
hsa-miR-105_st	4.294719	0.2920117	FALSE	4.272607	0.3006915	FALSE	4.283663
hsa-miR-105-star_st	3.805122	0.7550848	FALSE	3.711114	0.7884334	FALSE	3.758118
hsa-miR-106a_st	13.93984	2.05E-08	TRUE	14.10852	2.05E-08	TRUE	14.02418
hsa-miR-106a-star_st	3.92974	0.8195356	FALSE	3.710705	0.9759511	FALSE	3.8202225
hsa-miR-106b_st	11.75925	2.05E-08	TRUE	11.69164	2.05E-08	TRUE	11.725445
hsa-miR-106b-star_st	8.863229	0.000552592	TRUE	8.751545	0.000483581	TRUE	8.807387
hsa-miR-107_st	12.85947	2.05E-08	TRUE	13.03369	2.05E-08	TRUE	12.94658
hsa-miR-10a_st	10.22714	2.05E-08	TRUE	10.30341	2.05E-08	TRUE	10.265275
hsa-miR-10a-star_st	6.144808	8.41E-06	TRUE	5.834525	3.77E-05	TRUE	5.9896665
hsa-miR-10b_st	7.696976	2.05E-08	TRUE	7.486101	3.54E-07	TRUE	7.5915385
hsa-miR-10b-star_st	5.559112	0.000694596	TRUE	5.987772	0.000138725	TRUE	5.773442
hsa-miR-1178_st	3.974417	0.7396107	FALSE	3.890355	0.8296705	FALSE	3.932386
hsa-miR-1179_st	3.932132	0.7966915	FALSE	3.844566	0.9294101	FALSE	3.888349
hsa-miR-1180_st	6.958779	0.00515984	TRUE	7.104779	0.004285616	TRUE	7.031779
hsa-miR-1181_st	3.779994	0.7555314	FALSE	3.737576	0.6660923	FALSE	3.758785
hsa-miR-1182_st	4.461731	0.2271221	FALSE	3.692998	0.8128802	FALSE	4.0773645
hsa-miR-1183_st	4.053176	0.5788962	FALSE	3.875713	0.5961379	FALSE	3.9644445
hsa-miR-1184_st	4.093147	0.5001072	FALSE	4.013458	0.6001728	FALSE	4.0533025
hsa-miR-1185_st	3.991694	0.3999361	FALSE	4.076181	0.3024583	FALSE	4.0339375
hsa-miR-1197_st	3.782105	0.7387544	FALSE	3.935822	0.7017496	FALSE	3.8589635
hsa-miR-1200_st	3.877525	0.8570771	FALSE	3.908956	0.7988055	FALSE	3.8932405
hsa-miR-1201_st	8.313408	2.05E-08	TRUE	7.806952	1.33E-07	TRUE	8.06018
hsa-miR-1202_st	4.814062	0.142928	FALSE	4.554039	0.1449084	FALSE	4.6840505
hsa-miR-1203_st	3.627222	0.7065709	FALSE	3.905005	0.6614579	FALSE	3.7661135
hsa-miR-1204_st	3.67434	0.9823341	FALSE	3.70108	0.8614276	FALSE	3.68771
hsa-miR-1205_st	3.702563	0.9120726	FALSE	3.651854	0.9955559	FALSE	3.6772085
hsa-miR-1206_st	3.986838	0.8666963	FALSE	3.766152	0.8979182	FALSE	3.876495
hsa-miR-1207-3p_st	3.734989	0.7950026	FALSE	3.738982	0.8480401	FALSE	3.7369855
hsa-miR-1207-5p_st	8.653416	0.001845067	TRUE	8.32209	0.002747166	TRUE	8.487753
hsa-miR-1208_st	5.364491	0.00501359	TRUE	4.78966	0.03148764	TRUE	5.0770755
hsa-miR-122_st	3.838904	0.8929132	FALSE	4.051953	0.7738851	FALSE	3.9454285
hsa-miR-1224-3p_st	4.270638	0.5355817	FALSE	4.288438	0.3161747	FALSE	4.279538
hsa-miR-1224-5p_st	5.341234	0.02300812	TRUE	4.790774	0.07816555	FALSE	5.066004
hsa-miR-1225-3p_st	3.458519	0.8187156	FALSE	3.760551	0.6525635	FALSE	3.609535
hsa-miR-1225-5p_st	7.544112	0.01889602	TRUE	6.706877	0.03248493	TRUE	7.1254945
hsa-miR-1226_st	4.487405	0.3054223	FALSE	4.695328	0.1295467	FALSE	4.5913665
hsa-miR-1226-star_st	5.053464	0.1985627	FALSE	4.17711	0.3913439	FALSE	4.615287
hsa-miR-1227_st	4.314933	0.3407527	FALSE	3.845557	0.7894204	FALSE	4.080245
hsa-miR-1228_st	4.84207	0.1639514	FALSE	4.998221	0.1097067	FALSE	4.9201455

ProbeSet Name	Hey cell Signal_1	p-value (Hey cell Signal_1)	Detection (Hey cell Signal_1)	Hey cell Signal_2	p-value (Hey cell Signal_2)	Detection (Hey cell Signal_2)	Mean signal
hsa-miR-1228-star_st	10.60605	5.12E-05	TRUE	10.94761	2.48E-05	TRUE	10.77683
hsa-miR-1229_st	3.91684	0.6293426	FALSE	4.161025	0.4948559	FALSE	4.0389325
hsa-miR-122-star_st	4.120712	0.7870396	FALSE	4.228479	0.4941563	FALSE	4.1745955
hsa-miR-1231_st	7.792675	0.001750734	TRUE	8.064671	0.001184275	TRUE	7.928673
hsa-miR-1233_st	3.923553	0.6287264	FALSE	4.06652	0.5045198	FALSE	3.9950365
hsa-miR-1234_st	4.696789	0.2935984	FALSE	4.375046	0.3910246	FALSE	4.5359175
hsa-miR-1236_st	4.399593	0.347063	FALSE	4.345309	0.2734794	FALSE	4.372451
hsa-miR-1237_st	4.430656	0.293526	FALSE	4.332287	0.2284614	FALSE	4.3814715
hsa-miR-1238_st	4.300852	0.2180848	FALSE	4.241601	0.3589978	FALSE	4.2712265
hsa-miR-124_st	4.239136	0.3525625	FALSE	4.26461	0.3939606	FALSE	4.251873
hsa-miR-1243_st	3.938997	0.7556437	FALSE	4.16093	0.514423	FALSE	4.0499635
hsa-miR-1244_st	3.948797	0.3163179	FALSE	4.201441	0.2336573	FALSE	4.075119
hsa-miR-1245_st	4.049077	0.1324202	FALSE	4.22826	0.09840415	FALSE	4.1386685
hsa-miR-1246_st	9.884957	2.05E-08	TRUE	8.966446	2.05E-08	TRUE	9.4257015
hsa-miR-1247_st	3.800135	0.7462912	FALSE	3.812388	0.7090199	FALSE	3.8062615
hsa-miR-1248_st	4.377492	0.07883506	FALSE	4.520214	0.08112705	FALSE	4.448853
hsa-miR-1249_st	4.014685	0.4585056	FALSE	4.074961	0.4501922	FALSE	4.044823
hsa-miR-124-star_st	3.811332	0.8721417	FALSE	3.949465	0.8638604	FALSE	3.8803985
hsa-miR-1250_st	3.8864	0.7443426	FALSE	4.049546	0.585423	FALSE	3.967973
hsa-miR-1251_st	3.698272	0.8367361	FALSE	3.730442	0.8900133	FALSE	3.714357
hsa-miR-1252_st	3.876377	0.6551263	FALSE	3.881181	0.7928174	FALSE	3.878779
hsa-miR-1253_st	4.126504	0.3782144	FALSE	3.809846	0.9235324	FALSE	3.968175
hsa-miR-1254_st	5.219257	0.09052552	FALSE	5.72315	0.04081295	TRUE	5.4712035
hsa-miR-1255a_st	4.141746	0.3478979	FALSE	3.835788	0.8410297	FALSE	3.988767
hsa-miR-1255b_st	4.013327	0.5249751	FALSE	3.881932	0.6204208	FALSE	3.9476295
hsa-miR-1256_st	3.960435	0.6259312	FALSE	4.304706	0.2074982	FALSE	4.1325705
hsa-miR-1257_st	4.022422	0.4334977	FALSE	4.044281	0.4058984	FALSE	4.0333515
hsa-miR-1258_st	3.729508	0.955588	FALSE	4.068294	0.5403249	FALSE	3.898901
hsa-miR-1259_st	3.904502	0.5423377	FALSE	3.922356	0.3573198	FALSE	3.913429
hsa-miR-125a-3p_st	7.0344	0.00034341	TRUE	7.180879	0.000247674	TRUE	7.1076395
hsa-miR-125a-5p_st	12.39773	2.05E-08	TRUE	12.21575	2.05E-08	TRUE	12.30674
hsa-miR-125b_st	14.0486	2.05E-08	TRUE	13.95277	2.05E-08	TRUE	14.000685
hsa-miR-125b-1-star_st	9.182639	9.38E-06	TRUE	9.374179	8.56E-06	TRUE	9.278409
hsa-miR-125b-2-star_st	5.972874	0.001100614	TRUE	6.316291	0.000946953	TRUE	6.1445825
hsa-miR-126_st	9.391552	2.05E-08	TRUE	9.144051	2.05E-08	TRUE	9.2678015
hsa-miR-1260_st	5.471249	0.005962729	TRUE	5.700044	0.000411837	TRUE	5.5856465
hsa-miR-1261_st	3.745836	0.8384898	FALSE	3.789426	0.9800241	FALSE	3.767631
hsa-miR-1262_st	4.529433	0.04446588	TRUE	4.840852	0.02889414	TRUE	4.6851425
hsa-miR-1263_st	4.116495	0.1085121	FALSE	3.876593	0.4011238	FALSE	3.996544
hsa-miR-1264_st	4.063839	0.4239051	FALSE	3.793446	0.9294156	FALSE	3.9286425

ProbeSet Name	Hey cell Signal_1	p-value (Hey cell Signal_1)	Detection (Hey cell Signal_1)	Hey cell Signal_2	p-value (Hey cell Signal_2)	Detection (Hey cell Signal_2)	Mean signal
hsa-miR-1265_st	3.592929	0.9322825	FALSE	3.837762	0.9109658	FALSE	3.7153455
hsa-miR-1266_st	4.527523	0.1902611	FALSE	4.173364	0.4073615	FALSE	4.3504435
hsa-miR-1267_st	4.475516	0.06970482	FALSE	4.400655	0.1599331	FALSE	4.4380855
hsa-miR-1268_st	7.597053	0.00291815	TRUE	8.140071	0.001251397	TRUE	7.868562
hsa-miR-1269_st	3.572433	0.8902153	FALSE	3.71034	0.7670372	FALSE	3.6413865
hsa-miR-126-star_st	3.729451	0.9558689	FALSE	3.515414	0.9929667	FALSE	3.6224325
hsa-miR-1270_st	3.886401	0.4144686	FALSE	3.931901	0.4383041	FALSE	3.909151
hsa-miR-1271_st	3.729445	0.5433474	FALSE	3.712057	0.6966677	FALSE	3.720751
hsa-miR-1272_st	4.382951	0.09444676	FALSE	4.099275	0.2931413	FALSE	4.241113
hsa-miR-1273_st	4.030603	0.3828007	FALSE	4.040782	0.4107601	FALSE	4.0356925
hsa-miR-127-3p_st	3.495043	0.9688597	FALSE	3.720772	0.8178636	FALSE	3.6079075
hsa-miR-1274a_st	8.138761	1.17E-05	TRUE	8.362957	1.17E-05	TRUE	8.250859
hsa-miR-1274b_st	7.348765	3.45E-05	TRUE	6.86198	0.000257899	TRUE	7.1053725
hsa-miR-1275_st	7.663939	1.17E-05	TRUE	7.900241	1.17E-05	TRUE	7.78209
hsa-miR-127-5p_st	4.209868	0.4239683	FALSE	3.761409	0.7911346	FALSE	3.9856385
hsa-miR-1276_st	4.046614	0.4669642	FALSE	3.973763	0.3821809	FALSE	4.0101885
hsa-miR-1277_st	4.083542	0.3544721	FALSE	3.744227	0.8674582	FALSE	3.9138845
hsa-miR-1278_st	4.12065	0.4326709	FALSE	3.991508	0.6125289	FALSE	4.056079
hsa-miR-1279_st	4.162684	0.3757135	FALSE	3.775079	0.9380397	FALSE	3.9688815
hsa-miR-128_st	8.089877	2.05E-08	TRUE	8.033474	3.83E-08	TRUE	8.0616755
hsa-miR-1280_st	8.216311	6.76E-05	TRUE	8.102686	6.19E-05	TRUE	8.1594985
hsa-miR-1281_st	6.652599	0.000255581	TRUE	6.413093	0.000839763	TRUE	6.532846
hsa-miR-1282_st	4.102411	0.4649099	FALSE	4.086248	0.6929181	FALSE	4.0943295
hsa-miR-1283_st	3.747735	0.8038064	FALSE	3.7669	0.8291143	FALSE	3.7573175
hsa-miR-1284_st	4.019132	0.4973205	FALSE	4.216774	0.316926	FALSE	4.117953
hsa-miR-1285_st	5.349429	0.008000197	TRUE	5.446939	0.002150405	TRUE	5.398184
hsa-miR-1286_st	4.244865	0.244649	FALSE	3.795718	0.8610238	FALSE	4.0202915
hsa-miR-1287_st	5.663781	0.005813187	TRUE	5.813761	0.00295949	TRUE	5.738771
hsa-miR-1288_st	4.225593	0.3122947	FALSE	4.476561	0.2064401	FALSE	4.351077
hsa-miR-1289_st	4.063657	0.4669278	FALSE	3.850991	0.5181684	FALSE	3.957324
hsa-miR-1290_st	4.008868	0.6761907	FALSE	4.095496	0.5372359	FALSE	4.052182
hsa-miR-1291_st	4.18479	0.4550874	FALSE	3.878307	0.6384108	FALSE	4.0315485
hsa-miR-1292_st	5.063153	0.1859845	FALSE	5.003838	0.1942677	FALSE	5.0334955
hsa-miR-1293_st	3.77933	0.8927168	FALSE	4.15137	0.4858351	FALSE	3.96535
hsa-miR-129-3p_st	6.216746	5.12E-05	TRUE	6.319264	6.27E-05	TRUE	6.268005
hsa-miR-1294_st	4.079598	0.3155612	FALSE	4.091678	0.5383502	FALSE	4.085638
hsa-miR-1295_st	4.20998	0.5053571	FALSE	3.894757	0.7394715	FALSE	4.0523685
hsa-miR-129-5p_st	5.932788	0.002162432	TRUE	6.52132	0.000758512	TRUE	6.227054
hsa-miR-1296_st	5.019146	0.1272031	FALSE	4.725874	0.1372646	FALSE	4.87251
hsa-miR-1297_st	3.831675	0.7965046	FALSE	3.935736	0.764167	FALSE	3.8837055

ProbeSet Name	Hey cell Signal_1	p-value (Hey cell Signal_1)	Detection (Hey cell Signal_1)	Hey cell Signal_2	p-value (Hey cell Signal_2)	Detection (Hey cell Signal_2)	Mean signal
hsa-miR-1298_st	4.136746	0.5132353	FALSE	4.205998	0.3481751	FALSE	4.171372
hsa-miR-1299_st	3.881611	0.8107135	FALSE	3.923866	0.7569234	FALSE	3.9027385
hsa-miR-129-star_st	4.233841	0.3371578	FALSE	4.34889	0.4213686	FALSE	4.2913655
hsa-miR-1300_st	4.329413	0.2312918	FALSE	4.026256	0.5673299	FALSE	4.1778345
hsa-miR-1301_st	5.712546	0.02439521	TRUE	6.219471	0.01509884	TRUE	5.9660085
hsa-miR-1302_st	4.162684	0.3589496	FALSE	3.980667	0.3230999	FALSE	4.0716755
hsa-miR-1303_st	4.28048	0.243544	FALSE	4.742559	0.04016499	TRUE	4.5115195
hsa-miR-1304_st	4.668435	0.02704736	TRUE	4.439771	0.07612841	FALSE	4.554103
hsa-miR-1305_st	4.063806	0.2805449	FALSE	4.045281	0.287529	FALSE	4.0545435
hsa-miR-1306_st	4.367698	0.1872749	FALSE	4.075241	0.5732486	FALSE	4.2214695
hsa-miR-1307_st	8.24764	0.004187266	TRUE	8.392941	0.002449422	TRUE	8.3202905
hsa-miR-1308_st	11.86021	2.05E-08	TRUE	11.9251	2.05E-08	TRUE	11.892655
hsa-miR-130a_st	11.7821	2.05E-08	TRUE	11.96835	2.05E-08	TRUE	11.875225
hsa-miR-130a-star_st	3.883188	0.8166621	FALSE	3.886419	0.8338863	FALSE	3.8848035
hsa-miR-130b_st	10.74775	2.05E-08	TRUE	11.059	2.05E-08	TRUE	10.903375
hsa-miR-130b-star_st	4.628899	0.04625674	TRUE	4.313211	0.220986	FALSE	4.471055
hsa-miR-132_st	6.168651	4.57E-05	TRUE	6.495519	2.59E-05	TRUE	6.332085
hsa-miR-1321_st	4.028269	0.6165079	FALSE	3.939694	0.8117104	FALSE	3.9839815
hsa-miR-1322_st	4.237696	0.3514095	FALSE	3.641637	0.9977611	FALSE	3.9396665
hsa-miR-1323_st	3.859403	0.4729383	FALSE	4.357524	0.1369562	FALSE	4.1084635
hsa-miR-1324_st	4.065589	0.1786031	FALSE	4.027172	0.3394723	FALSE	4.0463805
hsa-miR-132-star_st	4.116494	0.5930519	FALSE	4.136693	0.6401972	FALSE	4.1265935
hsa-miR-133a_st	4.386775	0.2658106	FALSE	4.614928	0.1626665	FALSE	4.5008515
hsa-miR-133b_st	4.024795	0.4308959	FALSE	4.411987	0.2520852	FALSE	4.218391
hsa-miR-134_st	3.957549	0.4428719	FALSE	3.957137	0.5125054	FALSE	3.957343
hsa-miR-135a_st	4.238505	0.232075	FALSE	4.012304	0.7044257	FALSE	4.1254045
hsa-miR-135a-star_st	4.886287	0.04648359	TRUE	4.856808	0.06387976	FALSE	4.8715475
hsa-miR-135b_st	4.111466	0.4410193	FALSE	3.932126	0.6921414	FALSE	4.021796
hsa-miR-135b-star_st	4.675759	0.1148762	FALSE	4.636218	0.09742299	FALSE	4.6559885
hsa-miR-136_st	3.612421	0.9982606	FALSE	3.784857	0.939743	FALSE	3.698639
hsa-miR-136-star_st	3.832556	0.8913156	FALSE	4.00728	0.7062799	FALSE	3.919918
hsa-miR-137_st	5.571562	0.000373441	TRUE	4.030271	0.2914439	FALSE	4.8009165
hsa-miR-138_st	10.67512	1.33E-07	TRUE	11.01498	2.05E-08	TRUE	10.84505
hsa-miR-138-1-star_st	6.660732	0.001204631	TRUE	7.038321	0.000517718	TRUE	6.8495265
hsa-miR-138-2-star_st	3.846452	0.7590488	FALSE	4.180392	0.452588	FALSE	4.013422
hsa-miR-139-3p_st	5.209308	0.09832136	FALSE	5.80447	0.03314766	TRUE	5.506889
hsa-miR-139-5p_st	8.974593	5.13E-06	TRUE	9.158172	1.04E-06	TRUE	9.0663825
hsa-miR-140-3p_st	9.343713	8.45E-07	TRUE	10.09007	2.47E-07	TRUE	9.7168915
hsa-miR-140-5p_st	6.938517	1.04E-06	TRUE	7.349401	5.08E-07	TRUE	7.143959
hsa-miR-141_st	4.003829	0.4148116	FALSE	3.984135	0.3906237	FALSE	3.993982

ProbeSet Name	Hey cell Signal_1	p-value (Hey cell Signal_1)	Detection (Hey cell Signal_1)	Hey cell Signal_2	p-value (Hey cell Signal_2)	Detection (Hey cell Signal_2)	Mean signal
hsa-miR-141-star_st	3.808734	0.7769587	FALSE	3.730524	0.8788894	FALSE	3.769629
hsa-miR-142-3p_st	3.907839	0.7018078	FALSE	4.135271	0.2685286	FALSE	4.021555
hsa-miR-142-5p_st	4.393313	0.1914127	FALSE	3.983637	0.6089602	FALSE	4.188475
hsa-miR-143_st	3.825619	0.7682737	FALSE	4.17311	0.196147	FALSE	3.9993645
hsa-miR-143-star_st	3.622722	0.9884471	FALSE	3.830069	0.9256036	FALSE	3.7263955
hsa-miR-144_st	3.88987	0.8257416	FALSE	3.658429	0.9603883	FALSE	3.7741495
hsa-miR-144-star_st	4.54898	0.08510514	FALSE	4.443688	0.08155821	FALSE	4.496334
hsa-miR-145_st	4.076632	0.4837695	FALSE	3.935344	0.7428999	FALSE	4.005988
hsa-miR-145-star_st	3.69331	0.9370632	FALSE	4.09185	0.6232684	FALSE	3.89258
hsa-miR-146a_st	9.892371	2.05E-08	TRUE	9.458002	2.05E-08	TRUE	9.6751865
hsa-miR-146a-star_st	3.932826	0.8403023	FALSE	3.959673	0.8064723	FALSE	3.9462495
hsa-miR-146b-3p_st	3.940066	0.7385262	FALSE	3.879378	0.8243953	FALSE	3.909722
hsa-miR-146b-5p_st	5.638412	0.000719438	TRUE	5.122806	0.007771982	TRUE	5.380609
hsa-miR-147_st	3.934614	0.8527917	FALSE	3.703895	0.9860308	FALSE	3.8192545
hsa-miR-147b_st	3.720689	0.9383315	FALSE	3.769934	0.8248218	FALSE	3.7453115
hsa-miR-148a_st	4.297123	0.2404453	FALSE	3.859208	0.9247932	FALSE	4.0781655
hsa-miR-148a-star_st	3.837608	0.7505646	FALSE	3.71436	0.976975	FALSE	3.775984
hsa-miR-148b_st	7.33912	7.15E-08	TRUE	6.989817	1.34E-06	TRUE	7.1644685
hsa-miR-148b-star_st	3.812966	0.773815	FALSE	4.057539	0.6188592	FALSE	3.9352525
hsa-miR-149_st	6.406921	0.01035959	TRUE	7.064232	0.004742904	TRUE	6.7355765
hsa-miR-149-star_st	10.55822	5.12E-05	TRUE	10.84167	4.17E-05	TRUE	10.699945
hsa-miR-150_st	4.429358	0.1136374	FALSE	4.311132	0.2150544	FALSE	4.370245
hsa-miR-150-star_st	5.34482	0.08877129	FALSE	5.310308	0.0837204	FALSE	5.327564
hsa-miR-151-3p_st	11.4873	2.05E-08	TRUE	11.4092	2.05E-08	TRUE	11.44825
hsa-miR-151-5p_st	12.35631	2.05E-08	TRUE	12.10842	2.05E-08	TRUE	12.232365
hsa-miR-152_st	8.966776	2.05E-08	TRUE	8.507835	2.05E-08	TRUE	8.7373055
hsa-miR-153_st	3.635304	0.9398467	FALSE	3.896847	0.702127	FALSE	3.7660755
hsa-miR-154_st	4.247752	0.5967494	FALSE	3.56561	0.7485933	FALSE	3.906681
hsa-miR-154-star_st	3.782104	0.9188092	FALSE	4.107383	0.60171	FALSE	3.9447435
hsa-miR-155_st	12.00718	2.05E-08	TRUE	11.93585	2.05E-08	TRUE	11.971515
hsa-miR-155-star_st	3.837274	0.8730041	FALSE	3.903417	0.7872415	FALSE	3.8703455
hsa-miR-15a_st	11.68857	2.05E-08	TRUE	11.72018	2.05E-08	TRUE	11.704375
hsa-miR-15a-star_st	4.633328	0.08129809	FALSE	4.642715	0.08421403	FALSE	4.6380215
hsa-miR-15b_st	12.02743	2.05E-08	TRUE	11.88489	2.05E-08	TRUE	11.95616
hsa-miR-15b-star_st	3.829473	0.8586974	FALSE	3.461467	0.9998754	FALSE	3.64547
hsa-miR-16_st	13.78419	2.05E-08	TRUE	13.836	2.05E-08	TRUE	13.810095
hsa-miR-16-1-star_st	4.807825	0.0374077	TRUE	4.179473	0.3196035	FALSE	4.493649
hsa-miR-16-2-star_st	3.854957	0.6870728	FALSE	4.273192	0.3399662	FALSE	4.0640745
hsa-miR-17_st	14.04648	2.05E-08	TRUE	14.18098	2.05E-08	TRUE	14.11373
hsa-miR-17-star_st	9.162098	2.05E-08	TRUE	9.693462	2.05E-08	TRUE	9.42778

ProbeSet Name	Hey cell Signal_1	p-value (Hey cell Signal_1)	Detection (Hey cell Signal_1)	Hey cell Signal_2	p-value (Hey cell Signal_2)	Detection (Hey cell Signal_2)	Mean signal
hsa-miR-181a_st	11.64863	2.05E-08	TRUE	11.87433	2.05E-08	TRUE	11.76148
hsa-miR-181a-2-star_st	7.614795	1.17E-05	TRUE	7.969118	1.17E-05	TRUE	7.7919565
hsa-miR-181a-star_st	6.343099	8.41E-06	TRUE	6.926866	1.91E-07	TRUE	6.6349825
hsa-miR-181b_st	11.42958	2.05E-08	TRUE	11.51119	2.05E-08	TRUE	11.470385
hsa-miR-181c_st	7.789087	5.52E-08	TRUE	8.261718	3.83E-08	TRUE	8.0254025
hsa-miR-181c-star_st	4.261766	0.2876629	FALSE	4.115753	0.4578565	FALSE	4.1887595
hsa-miR-181d_st	7.993557	2.05E-08	TRUE	7.951527	7.15E-08	TRUE	7.972542
hsa-miR-182_st	10.10565	2.05E-08	TRUE	10.46882	2.05E-08	TRUE	10.287235
hsa-miR-1825_st	5.602044	0.008993738	TRUE	5.37183	0.009795989	TRUE	5.486937
hsa-miR-1826_st	13.12944	2.05E-08	TRUE	13.12166	2.05E-08	TRUE	13.12555
hsa-miR-1827_st	4.144358	0.2463959	FALSE	4.36795	0.3429435	FALSE	4.256154
hsa-miR-182-star_st	3.735371	0.6166992	FALSE	3.964725	0.3703997	FALSE	3.850048
hsa-miR-183_st	7.461993	3.83E-08	TRUE	7.752619	1.04E-06	TRUE	7.607306
hsa-miR-183-star_st	6.070929	0.000107682	TRUE	5.819757	0.000439962	TRUE	5.945343
hsa-miR-184_st	3.516989	0.8782895	FALSE	4.279265	0.3294367	FALSE	3.898127
hsa-miR-185_st	9.886943	2.05E-08	TRUE	10.30511	2.05E-08	TRUE	10.0960265
hsa-miR-185-star_st	3.37854	0.9955702	FALSE	3.904876	0.7832204	FALSE	3.641708
hsa-miR-186_st	4.761401	0.0444038	TRUE	4.407786	0.1789268	FALSE	4.5845935
hsa-miR-186-star_st	4.26176	0.302312	FALSE	4.359915	0.2713635	FALSE	4.3108375
hsa-miR-187_st	3.683671	0.910246	FALSE	3.703895	0.8838601	FALSE	3.693783
hsa-miR-187-star_st	4.620574	0.3063985	FALSE	4.31843	0.4171086	FALSE	4.469502
hsa-miR-188-3p_st	4.3715	0.220156	FALSE	4.703772	0.08069304	FALSE	4.537636
hsa-miR-188-5p_st	5.131291	0.03737931	TRUE	5.134079	0.03587193	TRUE	5.132685
hsa-miR-18a_st	12.52347	2.05E-08	TRUE	12.6166	2.05E-08	TRUE	12.570035
hsa-miR-18a-star_st	8.318987	6.76E-05	TRUE	8.341663	5.66E-05	TRUE	8.330325
hsa-miR-18b_st	11.0414	2.05E-08	TRUE	10.77644	2.05E-08	TRUE	10.90892
hsa-miR-18b-star_st	3.977759	0.4098765	FALSE	4.361583	0.2232393	FALSE	4.169671
hsa-miR-190_st	3.718907	0.9645385	FALSE	3.805469	0.8844213	FALSE	3.762188
hsa-miR-190b_st	3.798754	0.4627526	FALSE	3.96308	0.4553025	FALSE	3.880917
hsa-miR-191_st	11.93913	2.05E-08	TRUE	11.99267	2.05E-08	TRUE	11.9659
hsa-miR-191-star_st	4.755446	0.1059457	FALSE	4.946556	0.0809902	FALSE	4.851001
hsa-miR-192_st	6.655739	2.22E-06	TRUE	6.484567	5.23E-05	TRUE	6.570153
hsa-miR-192-star_st	3.822355	0.6360226	FALSE	3.944767	0.364886	FALSE	3.883561
hsa-miR-193a-3p_st	6.702578	1.33E-07	TRUE	7.525941	2.05E-08	TRUE	7.1142595
hsa-miR-193a-5p_st	9.888112	3.48E-06	TRUE	10.11076	1.73E-06	TRUE	9.999436
hsa-miR-193b_st	9.320252	6.24E-06	TRUE	9.578338	6.24E-06	TRUE	9.449295
hsa-miR-193b-star_st	7.84963	0.000111208	TRUE	7.626097	9.16E-05	TRUE	7.7378635
hsa-miR-194_st	7.096095	1.04E-06	TRUE	7.355047	7.27E-07	TRUE	7.225571
hsa-miR-194-star_st	3.610557	0.98553	FALSE	4.298053	0.4939365	FALSE	3.954305
hsa-miR-195_st	8.064267	2.05E-08	TRUE	8.114392	7.27E-07	TRUE	8.0893295

ProbeSet Name	Hey cell Signal_1	p-value (Hey cell Signal_1)	Detection (Hey cell Signal_1)	Hey cell Signal_2	p-value (Hey cell Signal_2)	Detection (Hey cell Signal_2)	Mean signal
hsa-miR-195-star_st	4.152751	0.2710456	FALSE	4.112276	0.3865848	FALSE	4.1325135
hsa-miR-196a_st	7.311774	7.15E-08	TRUE	7.048921	1.91E-06	TRUE	7.1803475
hsa-miR-196a-star_st	4.329838	0.123447	FALSE	3.783658	0.5846748	FALSE	4.056748
hsa-miR-196b_st	7.38699	7.15E-08	TRUE	7.189413	9.37E-07	TRUE	7.2882015
hsa-miR-197_st	6.189903	0.002782203	TRUE	5.913174	0.004075397	TRUE	6.0515385
hsa-miR-198_st	5.255562	0.023335	TRUE	5.328732	0.02873867	TRUE	5.292147
hsa-miR-199a-3p_st	10.02901	2.05E-08	TRUE	9.804936	2.05E-08	TRUE	9.916973
hsa-miR-199a-5p_st	8.519522	1.17E-05	TRUE	8.778739	6.02E-06	TRUE	8.6491305
hsa-miR-199b-3p_st	9.967819	2.05E-08	TRUE	9.91703	2.05E-08	TRUE	9.9424245
hsa-miR-199b-5p_st	4.20818	0.4246216	FALSE	4.234586	0.3808025	FALSE	4.221383
hsa-miR-19a_st	10.58794	2.05E-08	TRUE	10.53714	2.05E-08	TRUE	10.56254
hsa-miR-19a-star_st	3.90777	0.8128526	FALSE	3.692999	0.9775242	FALSE	3.8003845
hsa-miR-19b_st	13.67883	2.05E-08	TRUE	13.70858	2.05E-08	TRUE	13.693705
hsa-miR-19b-1-star_st	6.127168	7.66E-05	TRUE	6.152342	5.83E-05	TRUE	6.139755
hsa-miR-19b-2-star_st	3.602146	0.9924034	FALSE	3.809522	0.9448231	FALSE	3.705834
hsa-miR-200a_st	3.892243	0.6917766	FALSE	4.27897	0.3633761	FALSE	4.0856065
hsa-miR-200a-star_st	3.979609	0.7061421	FALSE	3.683453	0.8231331	FALSE	3.831531
hsa-miR-200b_st	4.778102	0.04085614	TRUE	4.248898	0.3246399	FALSE	4.5135
hsa-miR-200b-star_st	4.152031	0.3682013	FALSE	4.145669	0.6482013	FALSE	4.14885
hsa-miR-200c_st	5.60622	0.000761583	TRUE	5.845002	0.000212194	TRUE	5.725611
hsa-miR-200c-star_st	3.9747	0.5477098	FALSE	3.85502	0.6170706	FALSE	3.91486
hsa-miR-202_st	3.773212	0.8899378	FALSE	4.143922	0.3134468	FALSE	3.958567
hsa-miR-202-star_st	3.788894	0.7538696	FALSE	4.035348	0.6482922	FALSE	3.912121
hsa-miR-203_st	4.383618	0.2518529	FALSE	4.255846	0.4028025	FALSE	4.319732
hsa-miR-204_st	4.447665	0.1435351	FALSE	4.652451	0.05279343	TRUE	4.550058
hsa-miR-205_st	3.625818	0.902554	FALSE	3.880012	0.8469521	FALSE	3.752915
hsa-miR-206_st	3.8864	0.6450502	FALSE	3.893012	0.7120483	FALSE	3.889706
hsa-miR-208a_st	4.04524	0.5765167	FALSE	3.99679	0.8228611	FALSE	4.021015
hsa-miR-208b_st	3.724085	0.9838014	FALSE	4.010234	0.6612424	FALSE	3.8671595
hsa-miR-20a_st	13.76445	2.05E-08	TRUE	13.69843	2.05E-08	TRUE	13.73144
hsa-miR-20a-star_st	5.484936	0.001510538	TRUE	4.781619	0.02977411	TRUE	5.1332775
hsa-miR-20b_st	12.84971	2.05E-08	TRUE	12.66518	2.05E-08	TRUE	12.757445
hsa-miR-20b-star_st	3.903262	0.6986997	FALSE	3.947862	0.467509	FALSE	3.925562
hsa-miR-21_st	10.38654	2.05E-08	TRUE	9.90873	2.05E-08	TRUE	10.147635
hsa-miR-210_st	9.050625	0.000505588	TRUE	8.901565	0.000483581	TRUE	8.976095
hsa-miR-211_st	4.401057	0.314892	FALSE	4.800705	0.04900452	TRUE	4.600881
hsa-miR-212_st	4.385426	0.2949412	FALSE	4.754534	0.09933865	FALSE	4.56998
hsa-miR-214_st	4.694	0.1521323	FALSE	4.74034	0.1411407	FALSE	4.71717
hsa-miR-214-star_st	3.42675	0.9992249	FALSE	3.377351	0.999824	FALSE	3.4020505
hsa-miR-215_st	3.71732	0.9707584	FALSE	3.65994	0.914957	FALSE	3.68863

ProbeSet Name	Hey cell Signal_1	p-value (Hey cell Signal_1)	Detection (Hey cell Signal_1)	Hey cell Signal_2	p-value (Hey cell Signal_2)	Detection (Hey cell Signal_2)	Mean signal
hsa-miR-216a_st	4.19894	0.2654704	FALSE	3.885463	0.6468608	FALSE	4.0422015
hsa-miR-216b_st	3.965611	0.5901774	FALSE	4.091359	0.4088387	FALSE	4.028485
hsa-miR-217_st	3.708187	0.8714949	FALSE	4.224815	0.3957103	FALSE	3.966501
hsa-miR-218_st	3.833514	0.8169317	FALSE	3.766152	0.8827029	FALSE	3.799833
hsa-miR-218-1-star_st	3.948865	0.4059502	FALSE	3.824119	0.6691757	FALSE	3.886492
hsa-miR-218-2-star_st	4.102464	0.5298406	FALSE	4.266135	0.4531239	FALSE	4.1842995
hsa-miR-219-1-3p_st	4.027505	0.3132527	FALSE	4.114937	0.334783	FALSE	4.071221
hsa-miR-219-2-3p_st	3.828039	0.7034477	FALSE	3.979695	0.4568513	FALSE	3.903867
hsa-miR-219-5p_st	3.760351	0.9290358	FALSE	3.940122	0.6937125	FALSE	3.8502365
hsa-miR-21-star_st	7.379362	2.70E-05	TRUE	7.759491	1.17E-05	TRUE	7.5694265
hsa-miR-22_st	12.28333	2.05E-08	TRUE	12.48744	2.05E-08	TRUE	12.385385
hsa-miR-220a_st	4.236133	0.3309993	FALSE	4.272793	0.4170989	FALSE	4.254463
hsa-miR-220b_st	3.90156	0.7875335	FALSE	4.447812	0.2385241	FALSE	4.174686
hsa-miR-220c_st	3.780792	0.6275673	FALSE	3.60019	0.6633664	FALSE	3.690491
hsa-miR-221_st	13.71301	2.05E-08	TRUE	13.67924	2.05E-08	TRUE	13.696125
hsa-miR-221-star_st	7.380317	3.83E-08	TRUE	6.736021	3.54E-07	TRUE	7.058169
hsa-miR-222_st	13.18137	2.05E-08	TRUE	13.05637	2.05E-08	TRUE	13.11887
hsa-miR-222-star_st	7.964883	2.05E-08	TRUE	7.986437	2.05E-08	TRUE	7.97566
hsa-miR-223_st	4.44523	0.05799889	TRUE	4.823123	0.01589037	TRUE	4.6341765
hsa-miR-223-star_st	3.774049	0.9011675	FALSE	4.0359	0.8280412	FALSE	3.9049745
hsa-miR-224_st	6.790905	3.00E-06	TRUE	6.490772	6.71E-05	TRUE	6.6408385
hsa-miR-22-star_st	6.700493	1.73E-06	TRUE	5.886416	0.000258554	TRUE	6.2934545
hsa-miR-23a_st	14.07356	2.05E-08	TRUE	13.96663	2.05E-08	TRUE	14.020095
hsa-miR-23a-star_st	7.825211	9.28E-05	TRUE	8.133495	6.76E-05	TRUE	7.979353
hsa-miR-23b_st	12.95084	2.05E-08	TRUE	12.83041	2.05E-08	TRUE	12.890625
hsa-miR-23b-star_st	4.44324	0.2017044	FALSE	4.495137	0.1209215	FALSE	4.4691885
hsa-miR-24_st	13.6108	2.05E-08	TRUE	13.71277	2.05E-08	TRUE	13.661785
hsa-miR-24-1-star_st	3.988124	0.6866776	FALSE	3.897335	0.9116896	FALSE	3.9427295
hsa-miR-24-2-star_st	8.030046	2.05E-08	TRUE	8.27725	2.05E-08	TRUE	8.153648
hsa-miR-25_st	10.90728	2.05E-08	TRUE	10.7226	2.05E-08	TRUE	10.81494
hsa-miR-25-star_st	5.555406	0.004851778	TRUE	5.671277	0.005390134	TRUE	5.6133415
hsa-miR-26a_st	12.84485	2.05E-08	TRUE	12.82214	2.05E-08	TRUE	12.833495
hsa-miR-26a-1-star_st	3.673287	0.6914546	FALSE	3.698351	0.8713537	FALSE	3.685819
hsa-miR-26a-2-star_st	4.114666	0.3959299	FALSE	4.380797	0.1877646	FALSE	4.2477315
hsa-miR-26b_st	5.266018	0.001120008	TRUE	4.87576	0.03177686	TRUE	5.070889
hsa-miR-26b-star_st	4.780072	0.03445593	TRUE	4.135298	0.3155666	FALSE	4.457685
hsa-miR-27a_st	12.57553	2.05E-08	TRUE	12.84544	2.05E-08	TRUE	12.710485
hsa-miR-27a-star_st	9.504432	3.54E-07	TRUE	9.900805	2.47E-07	TRUE	9.7026185
hsa-miR-27b_st	9.86807	2.05E-08	TRUE	9.877617	2.05E-08	TRUE	9.8728435
hsa-miR-27b-star_st	5.928037	0.000188057	TRUE	6.437035	4.23E-05	TRUE	6.182536

ProbeSet Name	Hey cell Signal_1	p-value (Hey cell Signal_1)	Detection (Hey cell Signal_1)	Hey cell Signal_2	p-value (Hey cell Signal_2)	Detection (Hey cell Signal_2)	Mean signal
hsa-miR-28-3p_st	9.852942	2.05E-08	TRUE	9.774219	2.05E-08	TRUE	9.8135805
hsa-miR-28-5p_st	10.45352	2.05E-08	TRUE	10.56691	2.05E-08	TRUE	10.510215
hsa-miR-296-3p_st	4.461213	0.2459542	FALSE	4.545229	0.2678587	FALSE	4.503221
hsa-miR-296-5p_st	4.325723	0.3820266	FALSE	4.323389	0.4168358	FALSE	4.324556
hsa-miR-297_st	3.802657	0.8329407	FALSE	3.67532	0.9847863	FALSE	3.7389885
hsa-miR-298_st	3.874487	0.6551499	FALSE	3.969567	0.554555	FALSE	3.922027
hsa-miR-299-3p_st	4.316647	0.1972369	FALSE	4.311314	0.18752	FALSE	4.3139805
hsa-miR-299-5p_st	3.931579	0.7460359	FALSE	3.878307	0.7115523	FALSE	3.904943
hsa-miR-29a_st	13.51647	2.05E-08	TRUE	13.36726	2.05E-08	TRUE	13.441865
hsa-miR-29a-star_st	3.861657	0.856346	FALSE	3.990462	0.6769534	FALSE	3.9260595
hsa-miR-29b_st	9.970066	2.05E-08	TRUE	10.23165	2.05E-08	TRUE	10.100858
hsa-miR-29b-1-star_st	9.061357	2.05E-08	TRUE	8.908263	2.05E-08	TRUE	8.98481
hsa-miR-29b-2-star_st	5.195711	0.01490681	TRUE	5.076156	0.02617954	TRUE	5.1359335
hsa-miR-29c_st	7.039926	1.03E-07	TRUE	6.96658	2.47E-07	TRUE	7.003253
hsa-miR-29c-star_st	4.490908	0.1306837	FALSE	4.53687	0.07920701	FALSE	4.513889
hsa-miR-300_st	4.100615	0.5279948	FALSE	3.782823	0.8264092	FALSE	3.941719
hsa-miR-301a_st	8.999187	2.05E-08	TRUE	8.911174	2.05E-08	TRUE	8.9551805
hsa-miR-301b_st	4.212873	0.3144	FALSE	4.660367	0.0375449	TRUE	4.43662
hsa-miR-302a_st	3.679544	0.8921469	FALSE	4.04428	0.6179889	FALSE	3.861912
hsa-miR-302a-star_st	4.014927	0.5781744	FALSE	3.87221	0.9052343	FALSE	3.9435685
hsa-miR-302b_st	3.739034	0.9173374	FALSE	3.703895	0.992786	FALSE	3.7214645
hsa-miR-302b-star_st	3.83602	0.1078447	FALSE	3.828455	0.2269657	FALSE	3.8322375
hsa-miR-302c_st	3.698062	0.7129895	FALSE	3.703895	0.8887892	FALSE	3.7009785
hsa-miR-302c-star_st	4.203002	0.5034055	FALSE	4.181862	0.3963328	FALSE	4.192432
hsa-miR-302d_st	3.927685	0.774882	FALSE	3.705661	0.9570726	FALSE	3.816673
hsa-miR-302d-star_st	3.880051	0.6593373	FALSE	4.400568	0.1527875	FALSE	4.1403095
hsa-miR-302e_st	3.756855	0.9915972	FALSE	4.02828	0.5351376	FALSE	3.8925675
hsa-miR-302f_st	4.010996	0.618574	FALSE	3.820144	0.9146804	FALSE	3.91557
hsa-miR-30a_st	11.33071	2.05E-08	TRUE	11.30665	2.05E-08	TRUE	11.31868
hsa-miR-30a-star_st	9.542118	2.05E-08	TRUE	9.541613	2.05E-08	TRUE	9.5418655
hsa-miR-30b_st	9.958064	2.05E-08	TRUE	9.720056	2.05E-08	TRUE	9.83906
hsa-miR-30b-star_st	5.986281	0.000108452	TRUE	6.200021	0.000120505	TRUE	6.093151
hsa-miR-30c_st	11.29447	2.05E-08	TRUE	11.20706	2.05E-08	TRUE	11.250765
hsa-miR-30c-1-star_st	4.769015	0.07111707	FALSE	5.477239	0.01181197	TRUE	5.123127
hsa-miR-30c-2-star_st	7.511297	2.05E-08	TRUE	7.628953	2.05E-08	TRUE	7.570125
hsa-miR-30d_st	9.684246	2.05E-08	TRUE	9.660586	2.05E-08	TRUE	9.672416
hsa-miR-30d-star_st	4.177591	0.4676147	FALSE	3.905482	0.8324472	FALSE	4.0415365
hsa-miR-30e_st	7.222992	1.33E-07	TRUE	7.132535	1.04E-06	TRUE	7.1777635
hsa-miR-30e-star_st	7.510564	3.83E-08	TRUE	6.895238	4.41E-06	TRUE	7.202901
hsa-miR-31_st	3.671488	0.9383768	FALSE	3.727649	0.9244084	FALSE	3.6995685

ProbeSet Name	Hey cell Signal_1	p-value (Hey cell Signal_1)	Detection (Hey cell Signal_1)	Hey cell Signal_2	p-value (Hey cell Signal_2)	Detection (Hey cell Signal_2)	Mean signal
hsa-miR-31-star_st	4.051027	0.5751347	FALSE	4.054307	0.6186572	FALSE	4.052667
hsa-miR-32_st	4.080504	0.4874305	FALSE	3.756927	0.9136334	FALSE	3.9187155
hsa-miR-320a_st	11.34273	2.05E-08	TRUE	11.60002	2.05E-08	TRUE	11.471375
hsa-miR-320b_st	11.29085	2.05E-08	TRUE	11.60579	2.05E-08	TRUE	11.44832
hsa-miR-320c_st	11.30556	2.05E-08	TRUE	11.57421	2.05E-08	TRUE	11.439885
hsa-miR-320d_st	10.75707	2.05E-08	TRUE	10.86508	2.05E-08	TRUE	10.811075
hsa-miR-323-3p_st	4.105589	0.6069993	FALSE	3.604398	0.8767163	FALSE	3.8549935
hsa-miR-323-5p_st	3.106819	0.9078943	FALSE	3.850213	0.5319916	FALSE	3.478516
hsa-miR-324-3p_st	7.780801	0.001848006	TRUE	7.919982	0.001638247	TRUE	7.8503915
hsa-miR-324-5p_st	8.589381	0.000655339	TRUE	8.986996	0.000483581	TRUE	8.7881885
hsa-miR-325_st	4.257049	0.4057356	FALSE	3.8376	0.5295268	FALSE	4.0473245
hsa-miR-326_st	3.956652	0.3920549	FALSE	3.768158	0.5419138	FALSE	3.862405
hsa-miR-328_st	4.159292	0.4506812	FALSE	4.199529	0.5425952	FALSE	4.1794105
hsa-miR-329_st	3.970313	0.3376943	FALSE	3.952975	0.423375	FALSE	3.961644
hsa-miR-32-star_st	3.852492	0.6292697	FALSE	3.881181	0.5982715	FALSE	3.8668365
hsa-miR-330-3p_st	6.374653	0.0109384	TRUE	6.437079	0.01033869	TRUE	6.405866
hsa-miR-330-5p_st	4.046971	0.562011	FALSE	3.915195	0.6730492	FALSE	3.981083
hsa-miR-331-3p_st	6.864896	0.000650976	TRUE	6.55796	0.001109542	TRUE	6.711428
hsa-miR-331-5p_st	5.797997	0.008501192	TRUE	6.093332	0.002565847	TRUE	5.9456645
hsa-miR-335_st	5.622231	0.000453953	TRUE	5.695851	0.000363929	TRUE	5.659041
hsa-miR-335-star_st	3.955444	0.5281475	FALSE	4.095158	0.4586904	FALSE	4.025301
hsa-miR-337-3p_st	4.563292	0.08630962	FALSE	4.411986	0.07002068	FALSE	4.487639
hsa-miR-337-5p_st	4.193087	0.2631173	FALSE	4.006785	0.3240659	FALSE	4.099936
hsa-miR-338-3p_st	3.921505	0.4535367	FALSE	4.126955	0.3510246	FALSE	4.02423
hsa-miR-338-5p_st	5.037811	0.01803095	TRUE	4.937376	0.03320538	TRUE	4.9875935
hsa-miR-339-3p_st	7.227656	0.01376169	TRUE	7.792341	0.007172582	TRUE	7.5099985
hsa-miR-339-5p_st	7.966775	0.001440141	TRUE	8.216959	0.001143585	TRUE	8.091867
hsa-miR-33a_st	3.828206	0.7967433	FALSE	3.948527	0.4017134	FALSE	3.8883665
hsa-miR-33a-star_st	4.347897	0.1374208	FALSE	4.553307	0.06342371	FALSE	4.450602
hsa-miR-33b_st	3.696597	0.9617728	FALSE	3.752521	0.9781448	FALSE	3.724559
hsa-miR-33b-star_st	4.193037	0.5355298	FALSE	4.340283	0.5888814	FALSE	4.26666
hsa-miR-340_st	3.815474	0.7333414	FALSE	3.809414	0.8355172	FALSE	3.812444
hsa-miR-340-star_st	3.720405	0.9753059	FALSE	3.857708	0.9368957	FALSE	3.7890565
hsa-miR-342-3p_st	8.489235	1.17E-05	TRUE	8.752133	6.02E-06	TRUE	8.620684
hsa-miR-342-5p_st	5.240386	0.002530064	TRUE	5.0764	0.009458131	TRUE	5.158393
hsa-miR-345_st	6.322359	0.0116637	TRUE	6.679934	0.007248365	TRUE	6.5011465
hsa-miR-346_st	5.44629	0.08046463	FALSE	5.314171	0.06510813	FALSE	5.3802305
hsa-miR-34a_st	11.48083	2.05E-08	TRUE	11.63825	2.05E-08	TRUE	11.55954
hsa-miR-34a-star_st	8.463707	2.05E-08	TRUE	8.367687	2.05E-08	TRUE	8.415697
hsa-miR-34b_st	4.116495	0.5177085	FALSE	4.17711	0.4656454	FALSE	4.1468025

ProbeSet Name	Hey cell Signal_1	p-value (Hey cell Signal_1)	Detection (Hey cell Signal_1)	Hey cell Signal_2	p-value (Hey cell Signal_2)	Detection (Hey cell Signal_2)	Mean signal
hsa-miR-34b-star_st	4.761217	0.03343002	TRUE	4.795035	0.03467235	TRUE	4.778126
hsa-miR-34c-3p_st	4.359985	0.2692571	FALSE	4.206358	0.4857394	FALSE	4.2831715
hsa-miR-34c-5p_st	3.861472	0.8939115	FALSE	4.143691	0.3678715	FALSE	4.0025815
hsa-miR-361-3p_st	4.831573	0.09701066	FALSE	4.718982	0.06317347	FALSE	4.7752775
hsa-miR-361-5p_st	11.22049	2.05E-08	TRUE	11.37724	2.05E-08	TRUE	11.298865
hsa-miR-362-3p_st	4.423656	0.07232472	FALSE	4.373031	0.1719543	FALSE	4.3983435
hsa-miR-362-5p_st	6.969214	3.83E-08	TRUE	7.234544	2.05E-08	TRUE	7.101879
hsa-miR-363_st	4.085313	0.6185949	FALSE	4.199151	0.3472325	FALSE	4.142232
hsa-miR-363-star_st	4.027505	0.6387644	FALSE	4.07861	0.5681288	FALSE	4.0530575
hsa-miR-365_st	4.517023	0.2632387	FALSE	4.454588	0.1540934	FALSE	4.4858055
hsa-miR-367_st	3.930166	0.6098896	FALSE	3.888076	0.8381546	FALSE	3.909121
hsa-miR-367-star_st	4.285926	0.106075	FALSE	4.161735	0.3519641	FALSE	4.2238305
hsa-miR-369-3p_st	4.141056	0.5501054	FALSE	3.926553	0.4622097	FALSE	4.0338045
hsa-miR-369-5p_st	3.938997	0.592765	FALSE	3.81468	0.6286529	FALSE	3.8768385
hsa-miR-370_st	3.795783	0.712053	FALSE	4.011487	0.640092	FALSE	3.903635
hsa-miR-371-3p_st	3.851804	0.7739521	FALSE	4.21377	0.4769424	FALSE	4.032787
hsa-miR-371-5p_st	4.921411	0.04507086	TRUE	4.713943	0.1093896	FALSE	4.817677
hsa-miR-372_st	4.183377	0.3484201	FALSE	4.263911	0.2050205	FALSE	4.223644
hsa-miR-373_st	3.632411	0.9853121	FALSE	3.762522	0.6599941	FALSE	3.6974665
hsa-miR-373-star_st	5.394078	0.008606655	TRUE	4.894591	0.05489975	TRUE	5.1443345
hsa-miR-374a_st	4.164869	0.2725741	FALSE	3.823667	0.6592945	FALSE	3.994268
hsa-miR-374a-star_st	4.351226	0.116101	FALSE	4.151402	0.1903195	FALSE	4.251314
hsa-miR-374b_st	4.44908	0.04156358	TRUE	4.645103	0.04516261	TRUE	4.5470915
hsa-miR-374b-star_st	5.04333	0.03489241	TRUE	5.717365	0.000969084	TRUE	5.3803475
hsa-miR-375_st	3.542313	0.923592	FALSE	3.390872	0.9703995	FALSE	3.4665925
hsa-miR-376a_st	3.962384	0.1388021	FALSE	4.113528	0.1867275	FALSE	4.037956
hsa-miR-376a-star_st	3.959147	0.6664306	FALSE	4.18318	0.3072525	FALSE	4.0711635
hsa-miR-376b_st	4.214849	0.3124118	FALSE	3.878307	0.651742	FALSE	4.046578
hsa-miR-376c_st	3.804842	0.6237683	FALSE	4.041564	0.6538348	FALSE	3.923203
hsa-miR-377_st	3.723636	0.9296317	FALSE	3.932126	0.4044331	FALSE	3.827881
hsa-miR-377-star_st	3.798322	0.7798398	FALSE	3.990462	0.6079559	FALSE	3.894392
hsa-miR-378_st	10.4987	2.05E-08	TRUE	10.74733	2.05E-08	TRUE	10.623015
hsa-miR-378-star_st	6.49208	0.001065462	TRUE	6.910004	0.000490943	TRUE	6.701042
hsa-miR-379_st	4.018666	0.5833824	FALSE	4.268188	0.3837671	FALSE	4.143427
hsa-miR-379-star_st	3.943397	0.7064631	FALSE	4.18397	0.4526217	FALSE	4.0636835
hsa-miR-380_st	3.984816	0.5737847	FALSE	3.959507	0.5065987	FALSE	3.9721615
hsa-miR-380-star_st	3.881351	0.7984123	FALSE	3.979695	0.5335684	FALSE	3.930523
hsa-miR-381_st	3.853717	0.8189476	FALSE	3.824782	0.7879536	FALSE	3.8392495
hsa-miR-382_st	3.896189	0.8343616	FALSE	3.821469	0.8696277	FALSE	3.858829
hsa-miR-383_st	4.017117	0.6768663	FALSE	3.881532	0.9370254	FALSE	3.9493245

ProbeSet Name	Hey cell Signal_1	p-value (Hey cell Signal_1)	Detection (Hey cell Signal_1)	Hey cell Signal_2	p-value (Hey cell Signal_2)	Detection (Hey cell Signal_2)	Mean signal
hsa-miR-384_st	3.765955	0.9239839	FALSE	3.81303	0.8508592	FALSE	3.7894925
hsa-miR-409-3p_st	4.205003	0.4290074	FALSE	4.154153	0.5113637	FALSE	4.179578
hsa-miR-409-5p_st	4.000717	0.6870136	FALSE	3.881181	0.770983	FALSE	3.940949
hsa-miR-410_st	4.086976	0.7037769	FALSE	3.93688	0.6136336	FALSE	4.011928
hsa-miR-411_st	3.714956	0.9299247	FALSE	3.935821	0.7166102	FALSE	3.8253885
hsa-miR-411-star_st	3.944336	0.3965995	FALSE	4.054842	0.6017542	FALSE	3.999589
hsa-miR-412_st	3.920713	0.8616076	FALSE	4.298779	0.4676921	FALSE	4.109746
hsa-miR-421_st	7.147796	7.27E-07	TRUE	7.370607	5.08E-07	TRUE	7.2592015
hsa-miR-422a_st	9.95235	2.47E-07	TRUE	10.02749	2.47E-07	TRUE	9.98992
hsa-miR-423-3p_st	9.468464	9.44E-05	TRUE	9.672494	6.60E-05	TRUE	9.570479
hsa-miR-423-5p_st	7.74637	0.000134007	TRUE	8.052301	6.76E-05	TRUE	7.8993355
hsa-miR-424_st	6.798087	3.93E-07	TRUE	6.574832	3.54E-07	TRUE	6.6864595
hsa-miR-424-star_st	10.95021	2.05E-08	TRUE	11.1037	2.05E-08	TRUE	11.026955
hsa-miR-425_st	10.65754	2.05E-08	TRUE	10.73504	2.05E-08	TRUE	10.69629
hsa-miR-425-star_st	6.480427	0.0110382	TRUE	6.812973	0.006254818	TRUE	6.6467
hsa-miR-429_st	4.19894	0.1705141	FALSE	3.899575	0.6625661	FALSE	4.0492575
hsa-miR-431_st	3.781602	0.392839	FALSE	3.496956	0.7042443	FALSE	3.639279
hsa-miR-431-star_st	3.674339	0.7108653	FALSE	3.781373	0.850499	FALSE	3.727856
hsa-miR-432_st	6.959232	6.26E-05	TRUE	6.817521	0.000131837	TRUE	6.8883765
hsa-miR-432-star_st	3.399358	0.9131929	FALSE	3.628878	0.8756734	FALSE	3.514118
hsa-miR-433_st	3.651544	0.8145517	FALSE	4.117547	0.480315	FALSE	3.8845455
hsa-miR-448_st	3.662035	0.8506925	FALSE	3.592212	0.9125836	FALSE	3.6271235
hsa-miR-449a_st	4.308248	0.2395336	FALSE	3.943358	0.5256907	FALSE	4.125803
hsa-miR-449b_st	3.923585	0.5777347	FALSE	4.18909	0.3923577	FALSE	4.0563375
hsa-miR-450a_st	3.926942	0.6778746	FALSE	3.879213	0.9059538	FALSE	3.9030775
hsa-miR-450b-3p_st	4.055386	0.3523176	FALSE	3.573226	0.8758541	FALSE	3.814306
hsa-miR-450b-5p_st	3.544382	0.7402608	FALSE	3.94481	0.5028381	FALSE	3.744596
hsa-miR-451_st	4.41973	0.1369254	FALSE	3.929036	0.6943743	FALSE	4.174383
hsa-miR-452_st	6.67858	1.73E-06	TRUE	6.289215	2.59E-05	TRUE	6.4838975
hsa-miR-452-star_st	3.711299	0.906099	FALSE	3.739811	0.9659663	FALSE	3.725555
hsa-miR-453_st	3.841091	0.6069345	FALSE	4.079556	0.413562	FALSE	3.9603235
hsa-miR-454_st	6.152946	7.81E-06	TRUE	5.542192	0.004369434	TRUE	5.847569
hsa-miR-454-star_st	3.831262	0.8369657	FALSE	3.98014	0.5888253	FALSE	3.905701
hsa-miR-455-3p_st	11.12649	2.05E-08	TRUE	11.22028	2.05E-08	TRUE	11.173385
hsa-miR-455-5p_st	7.910523	2.05E-08	TRUE	7.879559	1.33E-07	TRUE	7.895041
hsa-miR-483-3p_st	4.191784	0.2018123	FALSE	4.392884	0.2416335	FALSE	4.292334
hsa-miR-483-5p_st	4.6347	0.07203483	FALSE	4.617806	0.1299706	FALSE	4.626253
hsa-miR-484_st	5.348094	0.05443101	TRUE	4.841135	0.1892893	FALSE	5.0946145
hsa-miR-485-3p_st	3.943346	0.4521222	FALSE	4.304315	0.1467259	FALSE	4.1238305
hsa-miR-485-5p_st	3.892829	0.7682304	FALSE	3.979695	0.7429408	FALSE	3.936262

ProbeSet Name	Hey cell Signal_1	p-value (Hey cell Signal_1)	Detection (Hey cell Signal_1)	Hey cell Signal_2	p-value (Hey cell Signal_2)	Detection (Hey cell Signal_2)	Mean signal
hsa-miR-486-3p_st	4.134856	0.5420291	FALSE	4.273312	0.204537	FALSE	4.204084
hsa-miR-486-5p_st	5.214447	0.0799602	FALSE	4.963392	0.09753851	FALSE	5.0889195
hsa-miR-487a_st	4.136078	0.4869339	FALSE	4.200295	0.4315059	FALSE	4.1681865
hsa-miR-487b_st	3.973408	0.6263136	FALSE	4.04428	0.5939404	FALSE	4.008844
hsa-miR-488_st	4.191642	0.2839728	FALSE	3.715142	0.9271194	FALSE	3.953392
hsa-miR-488-star_st	3.837274	0.952756	FALSE	4.373733	0.1858008	FALSE	4.1055035
hsa-miR-489_st	4.068357	0.5057915	FALSE	4.188136	0.5079623	FALSE	4.1282465
hsa-miR-490-3p_st	3.758442	0.8425336	FALSE	4.282236	0.4570412	FALSE	4.020339
hsa-miR-490-5p_st	4.341979	0.3586989	FALSE	4.043633	0.5434839	FALSE	4.192806
hsa-miR-491-3p_st	4.155323	0.614058	FALSE	4.019274	0.774105	FALSE	4.0872985
hsa-miR-491-5p_st	6.1705	0.00250132	TRUE	6.425521	0.001293509	TRUE	6.2980105
hsa-miR-492_st	4.282005	0.4094029	FALSE	4.526297	0.242071	FALSE	4.404151
hsa-miR-493_st	4.48596	0.1453358	FALSE	4.122112	0.4252124	FALSE	4.304036
hsa-miR-493-star_st	4.198169	0.3800416	FALSE	4.188744	0.3072525	FALSE	4.1934565
hsa-miR-494_st	5.63579	0.000599901	TRUE	5.696928	0.001064373	TRUE	5.666359
hsa-miR-495_st	3.643177	0.6649976	FALSE	3.881181	0.6413472	FALSE	3.762179
hsa-miR-496_st	4.112961	0.5713509	FALSE	3.798505	0.8244288	FALSE	3.955733
hsa-miR-497_st	5.947753	0.00012296	TRUE	6.171935	8.87E-05	TRUE	6.059844
hsa-miR-497-star_st	4.02303	0.6326542	FALSE	4.04428	0.5024709	FALSE	4.033655
hsa-miR-498_st	4.923378	0.06908072	FALSE	5.032862	0.02071069	TRUE	4.97812
hsa-miR-499-3p_st	3.97771	0.303127	FALSE	3.915286	0.4529903	FALSE	3.946498
hsa-miR-499-5p_st	4.207151	0.3831833	FALSE	4.057351	0.5395135	FALSE	4.132251
hsa-miR-500_st	6.958417	3.83E-08	TRUE	6.749035	1.73E-06	TRUE	6.853726
hsa-miR-500-star_st	6.369739	0.00046875	TRUE	6.942269	0.00034341	TRUE	6.656004
hsa-miR-501-3p_st	5.932824	0.002114834	TRUE	5.872178	0.002798033	TRUE	5.902501
hsa-miR-501-5p_st	5.710539	0.000469972	TRUE	4.877228	0.05049802	TRUE	5.2938835
hsa-miR-502-3p_st	6.405606	2.61E-05	TRUE	6.763336	1.73E-06	TRUE	6.584471
hsa-miR-502-5p_st	4.367465	0.1168102	FALSE	4.300923	0.312056	FALSE	4.334194
hsa-miR-503_st	10.90164	2.05E-08	TRUE	11.2476	2.05E-08	TRUE	11.07462
hsa-miR-504_st	4.468273	0.1609424	FALSE	4.05108	0.5400748	FALSE	4.2596765
hsa-miR-505_st	7.240119	2.05E-08	TRUE	7.18032	3.83E-08	TRUE	7.2102195
hsa-miR-505-star_st	6.684371	4.57E-07	TRUE	7.2526	3.83E-08	TRUE	6.9684855
hsa-miR-506_st	3.729508	0.7880169	FALSE	3.639535	0.9617266	FALSE	3.6845215
hsa-miR-507_st	3.553063	0.9707584	FALSE	3.553809	0.9820151	FALSE	3.553436
hsa-miR-508-3p_st	4.112099	0.2828276	FALSE	4.081444	0.2009154	FALSE	4.0967715
hsa-miR-508-5p_st	4.027504	0.4967488	FALSE	4.18158	0.3784354	FALSE	4.104542
hsa-miR-509-3-5p_st	3.76935	0.8387579	FALSE	4.141474	0.4139936	FALSE	3.955412
hsa-miR-509-3p_st	3.452567	0.9918223	FALSE	4.039241	0.48378	FALSE	3.745904
hsa-miR-509-5p_st	4.207032	0.1751283	FALSE	4.231334	0.2039361	FALSE	4.219183
hsa-miR-510_st	3.700382	0.9637713	FALSE	4.072307	0.5242771	FALSE	3.8863445

ProbeSet Name	Hey cell Signal_1	p-value (Hey cell Signal_1)	Detection (Hey cell Signal_1)	Hey cell Signal_2	p-value (Hey cell Signal_2)	Detection (Hey cell Signal_2)	Mean signal
hsa-miR-511_st	3.674974	0.9268514	FALSE	3.882509	0.8105507	FALSE	3.7787415
hsa-miR-512-3p_st	3.569501	0.4812775	FALSE	3.899404	0.3395547	FALSE	3.7344525
hsa-miR-512-5p_st	4.254316	0.4885301	FALSE	3.857541	0.6884778	FALSE	4.0559285
hsa-miR-513a-3p_st	4.115269	0.6599474	FALSE	3.986516	0.7921931	FALSE	4.0508925
hsa-miR-513a-5p_st	3.770756	0.6434341	FALSE	3.789649	0.7823306	FALSE	3.7802025
hsa-miR-513b_st	4.080624	0.3078528	FALSE	3.990754	0.7314004	FALSE	4.035689
hsa-miR-513c_st	3.960111	0.8368392	FALSE	3.789409	0.8173334	FALSE	3.87476
hsa-miR-514_st	3.815658	0.4655952	FALSE	4.020171	0.3475384	FALSE	3.9179145
hsa-miR-515-3p_st	3.887777	0.9050608	FALSE	3.785647	0.8413139	FALSE	3.836712
hsa-miR-515-5p_st	3.934307	0.8038064	FALSE	4.053321	0.6596034	FALSE	3.993814
hsa-miR-516a-3p_st	3.594727	0.9901389	FALSE	3.869199	0.9525231	FALSE	3.731963
hsa-miR-516a-5p_st	3.659872	0.9910498	FALSE	4.016975	0.5766147	FALSE	3.8384235
hsa-miR-516b_st	3.837273	0.836305	FALSE	3.800865	0.9059952	FALSE	3.819069
hsa-miR-516b-star_st	3.782105	0.8499294	FALSE	3.573227	0.9714102	FALSE	3.677666
hsa-miR-517a_st	3.974908	0.6946518	FALSE	4.133779	0.5396692	FALSE	4.0543435
hsa-miR-517b_st	3.729508	0.9617728	FALSE	3.683705	0.9860308	FALSE	3.7066065
hsa-miR-517c_st	3.611803	0.9917904	FALSE	3.888401	0.8348274	FALSE	3.750102
hsa-miR-517-star_st	4.11736	0.2690832	FALSE	4.176542	0.2357864	FALSE	4.146951
hsa-miR-518a-3p_st	3.446815	0.9766951	FALSE	3.662751	0.8118929	FALSE	3.554783
hsa-miR-518a-5p_st	3.923002	0.7302512	FALSE	3.503353	0.9398805	FALSE	3.7131775
hsa-miR-518b_st	3.98006	0.7564362	FALSE	4.068021	0.7111939	FALSE	4.0240405
hsa-miR-518c_st	3.935116	0.1652158	FALSE	4.054295	0.1718797	FALSE	3.9947055
hsa-miR-518c-star_st	3.730274	0.8445377	FALSE	3.559711	0.9883332	FALSE	3.6449925
hsa-miR-518d-3p_st	3.819478	0.6601523	FALSE	4.070041	0.5186864	FALSE	3.9447595
hsa-miR-518d-5p_st	4.339338	0.3219203	FALSE	4.050632	0.5627415	FALSE	4.194985
hsa-miR-518e_st	3.995858	0.6963575	FALSE	4.08956	0.3417127	FALSE	4.042709
hsa-miR-518e-star_st	3.90198	0.5765526	FALSE	3.889378	0.7111939	FALSE	3.895679
hsa-miR-518f_st	4.093259	0.3845177	FALSE	3.587891	0.8886055	FALSE	3.840575
hsa-miR-518f-star_st	3.907851	0.5531505	FALSE	3.483429	0.8591417	FALSE	3.69564
hsa-miR-519a_st	3.835804	0.7019257	FALSE	3.707371	0.930776	FALSE	3.7715875
hsa-miR-519a-star_st	3.54856	0.9363923	FALSE	3.52765	0.9913279	FALSE	3.538105
hsa-miR-519b-3p_st	3.812804	0.5596753	FALSE	4.203683	0.1920122	FALSE	4.0082435
hsa-miR-519b-5p_st	3.783021	0.6547992	FALSE	3.649988	0.8089203	FALSE	3.7165045
hsa-miR-519c-3p_st	3.667849	0.9846988	FALSE	4.023116	0.6396275	FALSE	3.8454825
hsa-miR-519c-5p_st	4.014771	0.4678391	FALSE	3.585886	0.9495234	FALSE	3.8003285
hsa-miR-519d_st	3.795563	0.941078	FALSE	4.035985	0.5593826	FALSE	3.915774
hsa-miR-519e_st	4.110226	0.5817406	FALSE	4.143016	0.3968859	FALSE	4.126621
hsa-miR-519e-star_st	3.642294	0.9390351	FALSE	3.944427	0.6528597	FALSE	3.7933605
hsa-miR-520a-3p_st	3.892367	0.5456793	FALSE	3.708177	0.6908869	FALSE	3.800272
hsa-miR-520a-5p_st	4.091393	0.5226581	FALSE	3.879119	0.841112	FALSE	3.985256

ProbeSet Name	Hey cell Signal_1	p-value (Hey cell Signal_1)	Detection (Hey cell Signal_1)	Hey cell Signal_2	p-value (Hey cell Signal_2)	Detection (Hey cell Signal_2)	Mean signal
hsa-miR-520b_st	4.337839	0.3780008	FALSE	4.300296	0.2793784	FALSE	4.3190675
hsa-miR-520c-3p_st	4.1485	0.3936969	FALSE	3.918572	0.8475497	FALSE	4.033536
hsa-miR-520c-5p_st	4.011654	0.7739521	FALSE	4.00046	0.6926981	FALSE	4.006057
hsa-miR-520d-3p_st	4.086782	0.2201285	FALSE	3.962957	0.3373818	FALSE	4.0248695
hsa-miR-520d-5p_st	3.938997	0.6777679	FALSE	3.878698	0.8134534	FALSE	3.9088475
hsa-miR-520e_st	4.05978	0.5080643	FALSE	4.066619	0.5829808	FALSE	4.0631995
hsa-miR-520f_st	3.492955	0.9946665	FALSE	3.869868	0.8573419	FALSE	3.6814115
hsa-miR-520g_st	4.275958	0.2914418	FALSE	4.080223	0.3941785	FALSE	4.1780905
hsa-miR-520h_st	3.870297	0.5380455	FALSE	4.01817	0.5180748	FALSE	3.9442335
hsa-miR-521_st	3.972246	0.6190668	FALSE	3.815179	0.8507148	FALSE	3.8937125
hsa-miR-522_st	3.664089	0.9579136	FALSE	4.038481	0.5656908	FALSE	3.851285
hsa-miR-522-star_st	3.871367	0.6429819	FALSE	4.093426	0.6082008	FALSE	3.9823965
hsa-miR-523_st	4.096522	0.538012	FALSE	4.078319	0.6047758	FALSE	4.0874205
hsa-miR-523-star_st	3.782104	0.6048398	FALSE	3.823666	0.8886678	FALSE	3.802885
hsa-miR-524-3p_st	3.895343	0.4904378	FALSE	3.747505	0.4635865	FALSE	3.821424
hsa-miR-524-5p_st	3.621454	0.9731191	FALSE	3.925102	0.7878731	FALSE	3.773278
hsa-miR-525-3p_st	3.744776	0.6180672	FALSE	3.968782	0.495066	FALSE	3.856779
hsa-miR-525-5p_st	3.855948	0.8386667	FALSE	3.596804	0.9736227	FALSE	3.726376
hsa-miR-526a_st	4.182644	0.3855365	FALSE	3.871162	0.540667	FALSE	4.026903
hsa-miR-526b_st	4.17387	0.4639899	FALSE	4.260913	0.4129297	FALSE	4.2173915
hsa-miR-526b-star_st	3.754835	0.874871	FALSE	3.844364	0.9176131	FALSE	3.7995995
hsa-miR-527_st	4.061071	0.5455509	FALSE	4.016181	0.5335331	FALSE	4.038626
hsa-miR-532-3p_st	5.621797	0.02951488	TRUE	5.43602	0.05370051	TRUE	5.5289085
hsa-miR-532-5p_st	7.707351	1.17E-05	TRUE	7.949915	1.17E-05	TRUE	7.828633
hsa-miR-539_st	4.064056	0.4683201	FALSE	4.390618	0.1824084	FALSE	4.227337
hsa-miR-541_st	3.782105	0.5818397	FALSE	3.635484	0.8042535	FALSE	3.7087945
hsa-miR-541-star_st	4.115592	0.5804335	FALSE	3.894967	0.7949302	FALSE	4.0052795
hsa-miR-542-3p_st	4.364634	0.1661289	FALSE	4.146534	0.1395894	FALSE	4.255584
hsa-miR-542-5p_st	5.453632	0.007863452	TRUE	5.591519	0.004052761	TRUE	5.5225755
hsa-miR-543_st	3.985187	0.5953295	FALSE	3.91582	0.6616709	FALSE	3.9505035
hsa-miR-544_st	3.76759	0.7260374	FALSE	3.734674	0.6396275	FALSE	3.751132
hsa-miR-545_st	3.849599	0.461124	FALSE	3.766152	0.7229632	FALSE	3.8078755
hsa-miR-545-star_st	3.642954	0.5361155	FALSE	3.636141	0.4824148	FALSE	3.6395475
hsa-miR-548a-3p_st	4.435707	0.1014314	FALSE	4.077708	0.2649289	FALSE	4.2567075
hsa-miR-548a-5p_st	4.122822	0.4710414	FALSE	3.672432	0.9109722	FALSE	3.897627
hsa-miR-548b-3p_st	3.958543	0.6361713	FALSE	4.160802	0.3803903	FALSE	4.0596725
hsa-miR-548b-5p_st	3.782105	0.8258841	FALSE	3.932126	0.8585168	FALSE	3.8571155
hsa-miR-548c-3p_st	4.123325	0.1675852	FALSE	4.135272	0.3233835	FALSE	4.1292985
hsa-miR-548c-5p_st	3.89218	0.7772719	FALSE	4.094689	0.7127033	FALSE	3.9934345
hsa-miR-548d-3p_st	4.123924	0.4064913	FALSE	3.902237	0.8981776	FALSE	4.0130805

ProbeSet Name	Hey cell Signal_1	p-value (Hey cell Signal_1)	Detection (Hey cell Signal_1)	Hey cell Signal_2	p-value (Hey cell Signal_2)	Detection (Hey cell Signal_2)	Mean signal
hsa-miR-548d-5p_st	3.922039	0.5990874	FALSE	3.878307	0.7525445	FALSE	3.900173
hsa-miR-548e_st	3.988124	0.7028735	FALSE	3.908541	0.7590316	FALSE	3.9483325
hsa-miR-548f_st	3.898425	0.6761805	FALSE	3.997552	0.4323498	FALSE	3.9479885
hsa-miR-548g_st	4.185207	0.296577	FALSE	4.228869	0.2396899	FALSE	4.207038
hsa-miR-548h_st	3.766775	0.9290358	FALSE	3.89809	0.9099492	FALSE	3.8324325
hsa-miR-548i_st	4.097689	0.3057609	FALSE	4.082894	0.5715425	FALSE	4.0902915
hsa-miR-548j_st	3.613161	0.9477497	FALSE	3.928214	0.9417311	FALSE	3.7706875
hsa-miR-548k_st	4.187449	0.3267375	FALSE	4.09901	0.3146196	FALSE	4.1432295
hsa-miR-548l_st	3.655862	0.9556123	FALSE	3.798832	0.8584221	FALSE	3.727347
hsa-miR-548m_st	3.852826	0.7839063	FALSE	3.641637	0.7444724	FALSE	3.7472315
hsa-miR-548n_st	3.775766	0.5584728	FALSE	3.938683	0.2880492	FALSE	3.8572245
hsa-miR-548o_st	3.741035	0.9367085	FALSE	3.801571	0.8895523	FALSE	3.771303
hsa-miR-548p_st	4.067286	0.1412653	FALSE	4.18909	0.1202655	FALSE	4.128188
hsa-miR-549_st	3.631453	0.6372942	FALSE	3.860529	0.5487206	FALSE	3.745991
hsa-miR-550_st	5.000689	0.07637079	FALSE	5.149182	0.0550432	TRUE	5.0749355
hsa-miR-550-star_st	5.805284	0.000506872	TRUE	5.441785	0.002608784	TRUE	5.6235345
hsa-miR-551a_st	3.750712	0.5567772	FALSE	3.891219	0.7821946	FALSE	3.8209655
hsa-miR-551b_st	4.453907	0.17614	FALSE	4.685008	0.07692655	FALSE	4.5694575
hsa-miR-551b-star_st	6.027129	0.001486885	TRUE	5.956176	0.001540874	TRUE	5.9916525
hsa-miR-552_st	4.052038	0.6027051	FALSE	4.190989	0.4363961	FALSE	4.1215135
hsa-miR-553_st	4.043039	0.297874	FALSE	4.113454	0.2950224	FALSE	4.0782465
hsa-miR-554_st	4.034569	0.2903994	FALSE	3.774985	0.7338743	FALSE	3.904777
hsa-miR-555_st	3.97748	0.6721056	FALSE	3.889037	0.8899302	FALSE	3.9332585
hsa-miR-556-3p_st	3.913007	0.5531458	FALSE	3.847717	0.4647327	FALSE	3.880362
hsa-miR-556-5p_st	3.619651	0.4554712	FALSE	3.681614	0.5919474	FALSE	3.6506325
hsa-miR-557_st	4.052978	0.5468037	FALSE	3.93513	0.4842668	FALSE	3.994054
hsa-miR-558_st	4.079658	0.6612121	FALSE	3.945654	0.698099	FALSE	4.012656
hsa-miR-559_st	4.585574	0.0617639	FALSE	4.699267	0.02601264	TRUE	4.6424205
hsa-miR-561_st	4.25989	0.257692	FALSE	4.442163	0.1322562	FALSE	4.3510265
hsa-miR-562_st	3.74159	0.9707584	FALSE	3.840666	0.6838791	FALSE	3.791128
hsa-miR-563_st	3.831858	0.7530136	FALSE	3.651846	0.980956	FALSE	3.741852
hsa-miR-564_st	4.387299	0.2430212	FALSE	4.324512	0.2604889	FALSE	4.3559055
hsa-miR-566_st	3.968199	0.7566602	FALSE	4.228718	0.4869422	FALSE	4.0984585
hsa-miR-567_st	3.858776	0.8566658	FALSE	3.90241	0.8221202	FALSE	3.880593
hsa-miR-568_st	3.837273	0.891726	FALSE	3.80881	0.6806163	FALSE	3.8230415
hsa-miR-569_st	4.288822	0.3350515	FALSE	3.845351	0.7237591	FALSE	4.0670865
hsa-miR-570_st	3.799477	0.3961032	FALSE	4.269595	0.1242473	FALSE	4.034536
hsa-miR-571_st	3.952803	0.7958874	FALSE	3.878307	0.8319772	FALSE	3.915555
hsa-miR-572_st	5.739026	0.0828618	FALSE	5.631927	0.08883692	FALSE	5.6854765
hsa-miR-573_st	3.674339	0.938373	FALSE	3.917438	0.6027244	FALSE	3.7958885

ProbeSet Name	Hey cell Signal_1	p-value (Hey cell Signal_1)	Detection (Hey cell Signal_1)	Hey cell Signal_2	p-value (Hey cell Signal_2)	Detection (Hey cell Signal_2)	Mean signal
hsa-miR-574-3p_st	9.573371	6.24E-06	TRUE	9.869659	2.86E-06	TRUE	9.721515
hsa-miR-574-5p_st	6.056917	4.03E-05	TRUE	6.70692	2.46E-06	TRUE	6.3819185
hsa-miR-575_st	4.174225	0.3438007	FALSE	4.128918	0.4440675	FALSE	4.1515715
hsa-miR-576-3p_st	4.062325	0.1818742	FALSE	5.018163	0.009137175	TRUE	4.540244
hsa-miR-576-5p_st	4.309227	0.3277567	FALSE	4.114842	0.5975484	FALSE	4.2120345
hsa-miR-577_st	3.697125	0.7745874	FALSE	3.910249	0.5304001	FALSE	3.803687
hsa-miR-578_st	3.988124	0.1589409	FALSE	3.703895	0.635302	FALSE	3.8460095
hsa-miR-579_st	3.894972	0.6434427	FALSE	4.260057	0.3981457	FALSE	4.0775145
hsa-miR-580_st	3.825432	0.9764963	FALSE	4.05	0.6289961	FALSE	3.937716
hsa-miR-581_st	3.784677	0.9610736	FALSE	3.881181	0.7056739	FALSE	3.832929
hsa-miR-582-3p_st	3.952499	0.5505304	FALSE	4.584504	0.0795037	FALSE	4.2685015
hsa-miR-582-5p_st	3.94685	0.4758968	FALSE	4.252396	0.2398855	FALSE	4.099623
hsa-miR-583_st	3.970992	0.6032587	FALSE	3.798147	0.7991463	FALSE	3.8845695
hsa-miR-584_st	7.447094	2.05E-08	TRUE	7.624513	2.05E-08	TRUE	7.5358035
hsa-miR-585_st	3.880562	0.7307878	FALSE	3.949141	0.7354065	FALSE	3.9148515
hsa-miR-586_st	4.109369	0.6185094	FALSE	3.723502	0.9109722	FALSE	3.9164355
hsa-miR-587_st	4.069919	0.5610173	FALSE	4.013098	0.5733172	FALSE	4.0415085
hsa-miR-588_st	4.045167	0.6005207	FALSE	3.864943	0.8560448	FALSE	3.955055
hsa-miR-589_st	6.320792	0.000420883	TRUE	6.224229	0.000921213	TRUE	6.2725105
hsa-miR-589-star_st	7.344418	3.20E-05	TRUE	7.850092	1.17E-05	TRUE	7.597255
hsa-miR-590-3p_st	3.769236	0.8349618	FALSE	3.743622	0.4398704	FALSE	3.756429
hsa-miR-590-5p_st	4.695969	0.01694104	TRUE	4.643748	0.0778811	FALSE	4.6698585
hsa-miR-591_st	4.220948	0.3650243	FALSE	4.374566	0.1967154	FALSE	4.297757
hsa-miR-592_st	3.701924	0.9558689	FALSE	3.545642	0.9315744	FALSE	3.623783
hsa-miR-593_st	4.837232	0.04121908	TRUE	4.928342	0.04677261	TRUE	4.882787
hsa-miR-593-star_st	3.101588	0.9680594	FALSE	2.774193	0.9951157	FALSE	2.9378905
hsa-miR-595_st	3.912405	0.5413811	FALSE	3.854763	0.5906612	FALSE	3.883584
hsa-miR-596_st	4.486268	0.346769	FALSE	4.797775	0.2479003	FALSE	4.6420215
hsa-miR-597_st	3.76042	0.8244639	FALSE	3.434942	0.8179352	FALSE	3.597681
hsa-miR-598_st	4.756566	0.024415	TRUE	4.618736	0.06093652	FALSE	4.687651
hsa-miR-599_st	3.730285	0.8016428	FALSE	3.887381	0.7078271	FALSE	3.808833
hsa-miR-600_st	3.937282	0.709349	FALSE	3.816734	0.4877967	FALSE	3.877008
hsa-miR-601_st	4.001557	0.7038789	FALSE	4.179389	0.3474648	FALSE	4.090473
hsa-miR-602_st	6.240932	0.09359368	FALSE	6.378741	0.08677292	FALSE	6.3098365
hsa-miR-603_st	4.014771	0.5730008	FALSE	3.844903	0.6187958	FALSE	3.929837
hsa-miR-604_st	4.029827	0.7867348	FALSE	4.084137	0.4950534	FALSE	4.056982
hsa-miR-605_st	4.421587	0.241398	FALSE	4.219505	0.3477647	FALSE	4.320546
hsa-miR-606_st	4.319701	0.0615292	FALSE	4.738008	0.006407762	TRUE	4.5288545
hsa-miR-607_st	3.724085	0.8822266	FALSE	3.925879	0.5787995	FALSE	3.824982
hsa-miR-608_st	3.301695	0.9253837	FALSE	3.180124	0.9079857	FALSE	3.2409095

ProbeSet Name	Hey cell Signal_1	p-value (Hey cell Signal_1)	Detection (Hey cell Signal_1)	Hey cell Signal_2	p-value (Hey cell Signal_2)	Detection (Hey cell Signal_2)	Mean signal
hsa-miR-609_st	3.885145	0.7148587	FALSE	3.882844	0.9429455	FALSE	3.8839945
hsa-miR-610_st	4.39193	0.2015708	FALSE	4.456765	0.1652174	FALSE	4.4243475
hsa-miR-611_st	3.061375	0.9312414	FALSE	2.827015	0.9260019	FALSE	2.944195
hsa-miR-612_st	3.671489	0.6762174	FALSE	4.529558	0.5238747	FALSE	4.1005235
hsa-miR-613_st	3.671229	0.7860206	FALSE	3.878307	0.6387182	FALSE	3.774768
hsa-miR-614_st	3.699297	0.9065493	FALSE	4.153728	0.5260763	FALSE	3.9265125
hsa-miR-615-3p_st	4.520157	0.1677212	FALSE	4.67928	0.1290785	FALSE	4.5997185
hsa-miR-615-5p_st	2.89671	0.9738274	FALSE	3.138088	0.9211925	FALSE	3.017399
hsa-miR-616_st	4.146812	0.5649739	FALSE	3.668056	0.8064005	FALSE	3.907434
hsa-miR-616-star_st	4.034314	0.6125814	FALSE	3.839918	0.8400822	FALSE	3.937116
hsa-miR-617_st	4.082909	0.253227	FALSE	3.795806	0.4383041	FALSE	3.9393575
hsa-miR-618_st	4.152257	0.4524161	FALSE	4.064955	0.488651	FALSE	4.108606
hsa-miR-619_st	4.527741	0.1280131	FALSE	4.537824	0.235976	FALSE	4.5327825
hsa-miR-620_st	3.719563	0.7538403	FALSE	3.764977	0.8739322	FALSE	3.74227
hsa-miR-621_st	3.701981	0.8649791	FALSE	4.135271	0.4527526	FALSE	3.918626
hsa-miR-622_st	4.494615	0.3530487	FALSE	4.295579	0.3288879	FALSE	4.395097
hsa-miR-623_st	4.796748	0.140741	FALSE	4.409204	0.3451956	FALSE	4.602976
hsa-miR-624_st	3.820546	0.9045044	FALSE	3.935821	0.7429042	FALSE	3.8781835
hsa-miR-624-star_st	4.110322	0.3212123	FALSE	4.411986	0.2021871	FALSE	4.261154
hsa-miR-625_st	9.533315	2.05E-08	TRUE	9.675993	2.05E-08	TRUE	9.604654
hsa-miR-625-star_st	4.317377	0.2139484	FALSE	4.291561	0.2633171	FALSE	4.304469
hsa-miR-626_st	4.578326	0.07557661	FALSE	4.663607	0.04854319	TRUE	4.6209665
hsa-miR-627_st	4.338558	0.05275135	TRUE	4.37548	0.0667443	FALSE	4.357019
hsa-miR-628-3p_st	4.752416	0.09404901	FALSE	4.732757	0.05638837	TRUE	4.7425865
hsa-miR-628-5p_st	3.941535	0.4569422	FALSE	4.098099	0.4696396	FALSE	4.019817
hsa-miR-629_st	3.929043	0.3351543	FALSE	4.250291	0.1429395	FALSE	4.089667
hsa-miR-629-star_st	4.23952	0.3615893	FALSE	4.70037	0.1435662	FALSE	4.469945
hsa-miR-630_st	3.796089	0.868533	FALSE	3.917711	0.7488306	FALSE	3.8569
hsa-miR-631_st	4.130648	0.3783777	FALSE	3.782257	0.6197174	FALSE	3.9564525
hsa-miR-632_st	3.996082	0.5453847	FALSE	4.065454	0.5227342	FALSE	4.030768
hsa-miR-633_st	3.817396	0.8726658	FALSE	3.974389	0.4691718	FALSE	3.8958925
hsa-miR-634_st	4.655919	0.09171977	FALSE	4.104418	0.454296	FALSE	4.3801685
hsa-miR-635_st	4.331712	0.5049654	FALSE	4.657455	0.1455988	FALSE	4.4945835
hsa-miR-636_st	4.223406	0.4220848	FALSE	4.648967	0.2172398	FALSE	4.4361865
hsa-miR-637_st	4.575109	0.2992126	FALSE	4.878978	0.252205	FALSE	4.7270435
hsa-miR-638_st	11.96706	1.29E-07	TRUE	12.13443	1.29E-07	TRUE	12.050745
hsa-miR-639_st	4.184123	0.3929093	FALSE	3.494496	0.7595924	FALSE	3.8393095
hsa-miR-640_st	4.01007	0.7063541	FALSE	3.935821	0.7043582	FALSE	3.9729455
hsa-miR-641_st	5.009493	0.01029102	TRUE	5.135797	0.008609486	TRUE	5.072645
hsa-miR-642_st	4.444835	0.2325747	FALSE	4.412399	0.1949984	FALSE	4.428617

ProbeSet Name	Hey cell Signal_1	p-value (Hey cell Signal_1)	Detection (Hey cell Signal_1)	Hey cell Signal_2	p-value (Hey cell Signal_2)	Detection (Hey cell Signal_2)	Mean signal
hsa-miR-643_st	3.785896	0.7891093	FALSE	4.003252	0.6595603	FALSE	3.894574
hsa-miR-644_st	3.852104	0.8671445	FALSE	4.097754	0.6654454	FALSE	3.974929
hsa-miR-645_st	3.808683	0.6666657	FALSE	3.703895	0.8172026	FALSE	3.756289
hsa-miR-646_st	3.988124	0.6331156	FALSE	4.038856	0.6452075	FALSE	4.01349
hsa-miR-647_st	3.47911	0.9536732	FALSE	3.434942	0.9973829	FALSE	3.457026
hsa-miR-648_st	4.162684	0.4554332	FALSE	4.202701	0.3985997	FALSE	4.1826925
hsa-miR-649_st	3.919739	0.718143	FALSE	3.908577	0.7220761	FALSE	3.914158
hsa-miR-650_st	3.433351	0.9047038	FALSE	3.92077	0.5202386	FALSE	3.6770605
hsa-miR-651_st	3.747785	0.9761488	FALSE	3.818435	0.8452777	FALSE	3.78311
hsa-miR-652_st	7.901204	1.17E-05	TRUE	8.065471	1.17E-05	TRUE	7.9833375
hsa-miR-653_st	4.0317	0.14131	FALSE	3.766122	0.6322541	FALSE	3.898911
hsa-miR-654-3p_st	3.934767	0.6367003	FALSE	3.823781	0.8838951	FALSE	3.879274
hsa-miR-654-5p_st	3.798463	0.6527658	FALSE	3.593699	0.684297	FALSE	3.696081
hsa-miR-655_st	3.983079	0.7337719	FALSE	3.935821	0.6719326	FALSE	3.95945
hsa-miR-656_st	3.841085	0.6846374	FALSE	3.935821	0.807206	FALSE	3.888453
hsa-miR-657_st	3.859697	0.6693218	FALSE	3.760372	0.7887769	FALSE	3.8100345
hsa-miR-658_st	4.62283	0.2675838	FALSE	4.559347	0.4035289	FALSE	4.5910885
hsa-miR-659_st	4.454639	0.2119378	FALSE	4.848621	0.102629	FALSE	4.65163
hsa-miR-660_st	6.288859	2.72E-05	TRUE	6.293735	0.000108201	TRUE	6.291297
hsa-miR-661_st	3.322417	0.910138	FALSE	3.677765	0.8028256	FALSE	3.500091
hsa-miR-662_st	3.959602	0.4995387	FALSE	3.669387	0.7658755	FALSE	3.8144945
hsa-miR-663_st	10.5103	0.000178449	TRUE	10.6648	0.000178449	TRUE	10.58755
hsa-miR-663b_st	3.736621	0.77376	FALSE	3.408893	0.8076742	FALSE	3.572757
hsa-miR-664_st	4.570993	0.06838157	FALSE	4.384512	0.1047572	FALSE	4.4777525
hsa-miR-664-star_st	4.6711	0.1452551	FALSE	4.663496	0.07869932	FALSE	4.667298
hsa-miR-665_st	5.055865	0.1493158	FALSE	5.132016	0.1139892	FALSE	5.0939405
hsa-miR-668_st	3.840742	0.7383781	FALSE	3.945145	0.6898683	FALSE	3.8929435
hsa-miR-671-3p_st	4.815671	0.1456338	FALSE	4.5895	0.2107421	FALSE	4.7025855
hsa-miR-671-5p_st	8.231975	0.003990533	TRUE	8.276594	0.003478216	TRUE	8.2542845
hsa-miR-675_st	4.643448	0.3352104	FALSE	4.666546	0.3000176	FALSE	4.654997
hsa-miR-7_st	5.302485	0.001032183	TRUE	4.330284	0.1059748	FALSE	4.8163845
hsa-miR-708_st	12.06751	2.05E-08	TRUE	12.12757	2.05E-08	TRUE	12.09754
hsa-miR-708-star_st	5.081185	0.004631702	TRUE	5.325858	0.002653851	TRUE	5.2035215
hsa-miR-7-1-star_st	5.471228	0.001428516	TRUE	4.885294	0.02657487	TRUE	5.178261
hsa-miR-720_st	10.20363	2.47E-07	TRUE	9.928072	2.47E-07	TRUE	10.065851
hsa-miR-7-2-star_st	4.510819	0.1847057	FALSE	4.699393	0.07472251	FALSE	4.605106
hsa-miR-744_st	8.70441	0.000628377	TRUE	8.656784	0.000483581	TRUE	8.680597
hsa-miR-744-star_st	4.632213	0.09065528	FALSE	4.914126	0.02741246	TRUE	4.7731695
hsa-miR-758_st	3.859867	0.8754287	FALSE	3.763794	0.8876556	FALSE	3.8118305
hsa-miR-760_st	4.837722	0.1274959	FALSE	4.708109	0.1316313	FALSE	4.7729155

ProbeSet Name	Hey cell Signal_1	p-value (Hey cell Signal_1)	Detection (Hey cell Signal_1)	Hey cell Signal_2	p-value (Hey cell Signal_2)	Detection (Hey cell Signal_2)	Mean signal
hsa-miR-765_st	4.046831	0.5632122	FALSE	3.900065	0.817022	FALSE	3.973448
hsa-miR-766_st	4.719189	0.2758213	FALSE	4.594862	0.3818856	FALSE	4.6570255
hsa-miR-767-3p_st	3.832144	0.9050608	FALSE	4.239129	0.3776647	FALSE	4.0356365
hsa-miR-767-5p_st	4.433182	0.1346944	FALSE	4.08751	0.4076909	FALSE	4.260346
hsa-miR-768-3p_st	8.48217	1.17E-05	TRUE	8.017783	1.17E-05	TRUE	8.2499765
hsa-miR-768-5p_st	9.402832	2.05E-08	TRUE	8.964705	2.05E-08	TRUE	9.1837685
hsa-miR-769-3p_st	4.759942	0.2246646	FALSE	4.710318	0.1431998	FALSE	4.73513
hsa-miR-769-5p_st	6.481266	0.000308651	TRUE	6.713896	0.000306987	TRUE	6.597581
hsa-miR-770-5p_st	3.848725	0.6435872	FALSE	3.947297	0.6047476	FALSE	3.898011
hsa-miR-802_st	4.153572	0.3818098	FALSE	4.305092	0.2077166	FALSE	4.229332
hsa-miR-873_st	3.785975	0.8847164	FALSE	3.828043	0.8497	FALSE	3.807009
hsa-miR-874_st	5.963933	0.07955744	FALSE	5.881582	0.09030714	FALSE	5.9227575
hsa-miR-875-3p_st	4.198941	0.1884007	FALSE	3.911893	0.3700442	FALSE	4.055417
hsa-miR-875-5p_st	3.709593	0.922632	FALSE	4.015528	0.5260491	FALSE	3.8625605
hsa-miR-876-3p_st	3.890883	0.9045135	FALSE	3.786877	0.8041504	FALSE	3.83888
hsa-miR-876-5p_st	3.748373	0.9140697	FALSE	3.996855	0.6383556	FALSE	3.872614
hsa-miR-877_st	6.247721	0.00154855	TRUE	6.13884	0.002226933	TRUE	6.1932805
hsa-miR-877-star_st	3.88987	0.6391857	FALSE	4.303388	0.4608909	FALSE	4.096629
hsa-miR-885-3p_st	6.155805	0.002493656	TRUE	6.677706	0.000801268	TRUE	6.4167555
hsa-miR-885-5p_st	5.454425	0.004333878	TRUE	4.432704	0.153047	FALSE	4.9435645
hsa-miR-886-3p_st	10.58867	5.08E-07	TRUE	10.93109	2.05E-08	TRUE	10.75988
hsa-miR-886-5p_st	10.62171	3.95E-06	TRUE	10.71346	5.02E-07	TRUE	10.667585
hsa-miR-887_st	5.117541	0.1450408	FALSE	5.487192	0.1631682	FALSE	5.3023665
hsa-miR-888_st	3.722581	0.9803475	FALSE	3.928366	0.7873548	FALSE	3.8254735
hsa-miR-888-star_st	3.88987	0.7624832	FALSE	4.071829	0.3633923	FALSE	3.9808495
hsa-miR-889_st	3.928509	0.4067622	FALSE	4.06952	0.2911307	FALSE	3.9990145
hsa-miR-890_st	4.034314	0.5348458	FALSE	4.336214	0.1956646	FALSE	4.185264
hsa-miR-891a_st	4.085426	0.5276688	FALSE	4.039991	0.26259	FALSE	4.0627085
hsa-miR-891b_st	3.887656	0.4311051	FALSE	3.813871	0.9052343	FALSE	3.8507635
hsa-miR-892a_st	3.837274	0.7944276	FALSE	3.849442	0.9159077	FALSE	3.843358
hsa-miR-892b_st	3.555408	0.9366919	FALSE	3.719685	0.9324513	FALSE	3.6375465
hsa-miR-9_st	3.941875	0.3752613	FALSE	3.815495	0.4330827	FALSE	3.878685
hsa-miR-920_st	4.172246	0.4024919	FALSE	4.021329	0.4819054	FALSE	4.0967875
hsa-miR-921_st	4.36731	0.3095008	FALSE	4.185671	0.4862677	FALSE	4.2764905
hsa-miR-922_st	3.490981	0.8169681	FALSE	3.936748	0.7023157	FALSE	3.7138645
hsa-miR-923_st	9.935972	2.05E-08	TRUE	10.18631	2.05E-08	TRUE	10.061141
hsa-miR-924_st	3.883108	0.7957023	FALSE	3.769799	0.9125836	FALSE	3.8264535
hsa-miR-92a_st	13.31682	2.05E-08	TRUE	13.44341	2.05E-08	TRUE	13.380115
hsa-miR-92a-1-star_st	9.135947	3.48E-06	TRUE	9.416792	3.54E-07	TRUE	9.2763695
hsa-miR-92a-2-star_st	3.994089	0.6065331	FALSE	3.990263	0.4496844	FALSE	3.992176

ProbeSet Name	Hey cell Signal_1	p-value (Hey cell Signal_1)	Detection (Hey cell Signal_1)	Hey cell Signal_2	p-value (Hey cell Signal_2)	Detection (Hey cell Signal_2)	Mean signal
hsa-miR-92b_st	8.489407	0.000711255	TRUE	8.523087	0.000575051	TRUE	8.506247
hsa-miR-92b-star_st	7.430676	0.01134661	TRUE	7.490614	0.008905658	TRUE	7.460645
hsa-miR-93_st	12.19179	2.05E-08	TRUE	12.34337	2.05E-08	TRUE	12.26758
hsa-miR-933_st	5.643908	0.03975105	TRUE	5.717902	0.03563017	TRUE	5.680905
hsa-miR-934_st	3.856082	0.5217686	FALSE	4.063932	0.459712	FALSE	3.960007
hsa-miR-935_st	5.351268	0.07054949	FALSE	5.084011	0.118783	FALSE	5.2176395
hsa-miR-936_st	6.736971	0.000233301	TRUE	7.129507	0.000152061	TRUE	6.933239
hsa-miR-937_st	4.177038	0.5935757	FALSE	4.043628	0.6594062	FALSE	4.110333
hsa-miR-938_st	4.711708	0.09940834	FALSE	4.125199	0.3905774	FALSE	4.4184535
hsa-miR-939_st	5.816607	0.08231006	FALSE	5.65814	0.08369344	FALSE	5.7373735
hsa-miR-93-star_st	6.140809	0.002503207	TRUE	6.621677	0.001100055	TRUE	6.381243
hsa-miR-940_st	4.471837	0.2970239	FALSE	3.761409	0.6055794	FALSE	4.116623
hsa-miR-941_st	5.778267	0.04449993	TRUE	6.350277	0.01818575	TRUE	6.064272
hsa-miR-942_st	4.01647	0.5115468	FALSE	3.864563	0.9050276	FALSE	3.9405165
hsa-miR-943_st	4.895525	0.03832408	TRUE	4.442006	0.1590342	FALSE	4.6687655
hsa-miR-944_st	4.631762	0.1099908	FALSE	4.506423	0.1482886	FALSE	4.5690925
hsa-miR-95_st	3.748559	0.818207	FALSE	4.056669	0.3357451	FALSE	3.902614
hsa-miR-96_st	4.767233	0.04596073	TRUE	4.694583	0.05749543	TRUE	4.730908
hsa-miR-96-star_st	4.33795	0.3723719	FALSE	3.971281	0.8105023	FALSE	4.1546155
hsa-miR-98_st	5.675147	3.35E-05	TRUE	5.642511	0.000145532	TRUE	5.658829
hsa-miR-99a_st	11.62423	2.05E-08	TRUE	11.19659	2.05E-08	TRUE	11.41041
hsa-miR-99a-star_st	3.870248	0.6101778	FALSE	4.104395	0.4835711	FALSE	3.9873215
hsa-miR-99b_st	10.83678	3.54E-07	TRUE	11.04576	2.47E-07	TRUE	10.94127
hsa-miR-99b-star_st	7.039983	0.004568201	TRUE	7.140141	0.004288437	TRUE	7.090062
hsa-miR-9-star_st	4.29178	0.2544417	FALSE	3.996081	0.5070534	FALSE	4.1439305

Supplementary Table 6. miRNA target enrichment analysis among up-regulated genes following miR-7 transfection. Genomica enrichment (FDR <0.05) of miRNA targets among significantly up-regulated genes following miR-7 transfection into HEY cells. Regulating miRNAs are predicted using miRanda (those with the prefix John04 or SloanKettering), TargetScan or PicTar. Only "up" genes, i.e. up-regulated in the transfection, are analyzed ('Set'). The other columns show the p-value of enrichment, the number of probesets in our dataset that are up-regulated and present in Genomica database ('Set Size'), compared to the number of probesets in each set in the background Affymetrix chip ('Total Size'). If a probeset is a predicted target of a miRNA then it is considered a 'hit' ('Set Hits' and 'Total Hits'). The percentages calculated from raw numbers of genes that belong in each set are also shown.

Set	Enriched Set	Enriched Value	P-value	Set Hits	Set Size	Set Hits (%)	Total Hits	Total Size	Total Hits (%)
Up	Pictar_hsa-miR-199b	1	4.12E-05	11	174	6.33	613	43407	1.42
Up	Pictar_hsa-miR-199a	1	3.60E-06	11	174	6.33	471	43407	1.09

Supplementary Table 7. Hub genes and their targets affected by miR-7 in HEY cells. GeneGo was used to analyze the most significant interactions within the set of differentially expressed genes following miR-7 transfection. The analysis gives a list of "hub" genes interacting with other differentially expressed genes (i.e. nodes) which suggests that miRNAs are regulating the node genes indirectly through regulating the hubs. "IDs in active dataset" refer to the Probeset IDs in the dataset that passed the GeneGo FDR threshold of 5% and are acting as hubs. Corresponding gene names/symbols are given in "Object name" column. "IDs of corresponding network object from GeneGo network" refer to probeset IDs of objects in the dataset that the hubs are interacting with in GeneGo networks. The names of these nodes are given in the last column. If a hub gene is found to regulate another gene which may also act as a hub then the row is shaded. Genes that are predicted to be miR-7 targets by miRanda or may act as transcription factors are marked with 'x'.

IDs in active data set	Object name	miR-7 tgts_miRanda	Transcription Factor	IDs of corresponding network object from GeneGo network	Corresponding network object name
201783_s_at; 230202_at	RelA (p65 NF-kB subunit)	x	x	1555938_x_at	Vimentin
201783_s_at; 230202_at	RelA (p65 NF-kB subunit)	x	x	1564907_s_at; 242260_at	Matrin-3
201783_s_at; 230202_at	RelA (p65 NF-kB subunit)	x	x	1568574_x_at; 209875_s_at	Osteopontin
201783_s_at; 230202_at	RelA (p65 NF-kB subunit)	x	x	1569788_at	SIAT8A
201783_s_at; 230202_at	RelA (p65 NF-kB subunit)	x	x	200690_at	GRP75
201783_s_at; 230202_at	RelA (p65 NF-kB subunit)	x	x	201042_at	TGM2

IDs in active data set	Object name	miR-7 tgts_miRanda	Transcription Factor	IDs of corresponding network object from GeneGo network	Corresponding network object name
201783_s_at; 230202_at	RelA (p65 NF-kB subunit)	x	x	201244_s_at	c-Raf-1
201783_s_at; 230202_at	RelA (p65 NF-kB subunit)	x	x	202443_x_at; 212377_s_at	NOTCH2 (2ICD)
201783_s_at; 230202_at	RelA (p65 NF-kB subunit)	x	x	202847_at	PPCKM
201783_s_at; 230202_at	RelA (p65 NF-kB subunit)	x	x	202859_x_at; 211506_s_at	IL-8
201783_s_at; 230202_at	RelA (p65 NF-kB subunit)	x	x	202886_s_at	PPP2R1B
201783_s_at; 230202_at	RelA (p65 NF-kB subunit)	x	x	203220_s_at; 203222_s_at	TLE1
201783_s_at; 230202_at	RelA (p65 NF-kB subunit)	x	x	204296_at	DCTN1(p150Glued)
201783_s_at; 230202_at	RelA (p65 NF-kB subunit)	x	x	204470_at	GRO-1
201783_s_at; 230202_at	RelA (p65 NF-kB subunit)	x	x	204799_at	Y637
201783_s_at; 230202_at	RelA (p65 NF-kB subunit)	x	x	205067_at; 39402_at	IL-1 beta
201783_s_at; 230202_at	RelA (p65 NF-kB subunit)	x	x	205289_at	BMP2
201783_s_at; 230202_at	RelA (p65 NF-kB subunit)	x	x	205416_s_at	MJD (ataxin-3)
201783_s_at; 230202_at	RelA (p65 NF-kB subunit)	x	x	205798_at	IL7RA
201783_s_at; 230202_at	RelA (p65 NF-kB subunit)	x	x	207196_s_at	TNIP1
201783_s_at; 230202_at	RelA (p65 NF-kB subunit)	x	x	207563_s_at; 207564_x_at; 209240_at; 212307_s_at; 220594_at; 229787_s_at	OGT (GlcNAc transferase)

IDs in active data set	Object name	miR-7 tgts_miRanda	Transcription Factor	IDs of corresponding network object from GeneGo network	Corresponding network object name
201783_s_at; 230202_at	RelA (p65 NF-kB subunit)	x	x	209383_at	C/EBP zeta
201783_s_at; 230202_at	RelA (p65 NF-kB subunit)	x	x	209774_x_at	GRO-2
201783_s_at; 230202_at	RelA (p65 NF-kB subunit)	x	x	209946_at	VEGF-C
201783_s_at; 230202_at	RelA (p65 NF-kB subunit)	x	x	210385_s_at; 212580_at	ARTS-1
201783_s_at; 230202_at	RelA (p65 NF-kB subunit)	x	x	211434_s_at	CCRL2
201783_s_at; 230202_at	RelA (p65 NF-kB subunit)	x	x	212188_at	KCTD12
201783_s_at; 230202_at	RelA (p65 NF-kB subunit)	x	x	214528_s_at	PAX8
201783_s_at; 230202_at	RelA (p65 NF-kB subunit)	x	x	215485_s_at	ICAM1
201783_s_at; 230202_at	RelA (p65 NF-kB subunit)	x	x	217996_at	PHLDA1
201783_s_at; 230202_at	RelA (p65 NF-kB subunit)	x	x	220266_s_at; 221841_s_at	KLF4
201783_s_at; 230202_at	RelA (p65 NF-kB subunit)	x	x	221477_s_at	SOD2
201783_s_at; 230202_at	RelA (p65 NF-kB subunit)	x	x	223217_s_at	IKBZ
201783_s_at; 230202_at	RelA (p65 NF-kB subunit)	x	x	224654_at	DDX21
201783_s_at; 230202_at	RelA (p65 NF-kB subunit)	x	x	224917_at	microRNA 21
201783_s_at; 230202_at	RelA (p65 NF-kB subunit)	x	x	227143_s_at	Bid
201783_s_at; 230202_at	RelA (p65 NF-kB subunit)	x	x	228363_at; 235222_x_at	XIAP

IDs in active data set	Object name	miR-7 tgts_miRanda	Transcription Factor	IDs of corresponding network object from GeneGo network	Corresponding network object name
201783_s_at; 230202_at	RelA (p65 NF-kB subunit)	x	x	235020_at	TAF4B
201783_s_at; 230202_at	RelA (p65 NF-kB subunit)	x	x	235795_at	PAX6
201783_s_at; 230202_at	RelA (p65 NF-kB subunit)	x	x	240221_at	Casein kinase I alpha
202827_s_at	MMP-14			201010_s_at	TXNIP (VDUP1)
202827_s_at	MMP-14			201042_at	TGM2
202827_s_at	MMP-14			201147_s_at; 201149_s_at; 201150_s_at	TIMP3
202827_s_at	MMP-14			201646_at; 201647_s_at; 224983_at	CD36L2
202827_s_at	MMP-14			201920_at; 230494_at	GLVR1
202827_s_at	MMP-14			202007_at	Nidogen
202827_s_at	MMP-14			202267_at	LAMC2
202827_s_at	MMP-14			202443_x_at; 212377_s_at	NOTCH2
202827_s_at	MMP-14			202620_s_at	PLOD2
202827_s_at	MMP-14			202859_x_at; 211506_s_at	IL-8
202827_s_at	MMP-14			203821_at; 38037_at	HB-EGF
202827_s_at	MMP-14			203888_at	Thrombomodulin
202827_s_at	MMP-14			205227_at	IL1RAP
202827_s_at	MMP-14			207332_s_at; 208691_at	TfR1
202827_s_at	MMP-14			209281_s_at	PMCA1

IDs in active data set	Object name	miR-7 tgts_miRanda	Transcription Factor	IDs of corresponding network object from GeneGo network	Corresponding network object name
202827_s_at	MMP-14			210495_x_at; 211719_x_at; 212464_s_at; 216442_x_at	Fibronectin
202827_s_at	MMP-14			211607_x_at	EGFR
202827_s_at	MMP-14			212097_at	Caveolin-1
202827_s_at	MMP-14			212761_at	TCF7L2 (TCF4)
202827_s_at	MMP-14			224953_at	YIPF5
202827_s_at	MMP-14			226217_at	ZnT7
202827_s_at	MMP-14			226545_at; 229900_at	CD109
202827_s_at	MMP-14			232231_at	RUNX2
202827_s_at	MMP-14			239627_at	TMED9
202827_s_at	MMP-14			34478_at	Rab-11B
206011_at	Caspase-1	x		1554479_a_at; 204950_at	CARD8
206011_at	Caspase-1	x		1555938_x_at	Vimentin
206011_at	Caspase-1	x		1556607_at; 209536_s_at; 233660_at	EHD4
206011_at	Caspase-1	x		1564907_s_at; 242260_at	Matrin-3
206011_at	Caspase-1	x		200681_at	Glyoxalase I
206011_at	Caspase-1	x		200755_s_at; 200756_x_at; 200757_s_at; 214845_s_at	Calumenin
206011_at	Caspase-1	x		203555_at	PTPN18
206011_at	Caspase-1	x		205067_at; 39402_at	IL-1 beta

IDs in active data set	Object name	miR-7 tgts_miRanda	Transcription Factor	IDs of corresponding network object from GeneGo network	Corresponding network object name
206011_at	Caspase-1	x		205416_s_at	MJD (ataxin-3)
206011_at	Caspase-1	x		208374_s_at	CAPZA1
206011_at	Caspase-1	x		209615_s_at; 226507_at	PAK1
206011_at	Caspase-1	x		209822_s_at	VLDLR
206011_at	Caspase-1	x		214291_at	RPL17
206011_at	Caspase-1	x		218850_s_at	LIMD1
206011_at	Caspase-1	x		222519_s_at	HIPPI
209037_s_at	EHD1	x		1556607_at; 209536_s_at; 233660_at	EHD4
209037_s_at	EHD1	x		201783_s_at; 230202_at	RelA (p65 NF-kB subunit)
209037_s_at	EHD1	x		222597_at	SNAP-29
209037_s_at	EHD1	x		226581_at	Rabenosyn-5
209037_s_at	EHD1	x		227428_at	GABP alpha
209112_at	p27KIP1			200952_s_at	Cyclin D2
209112_at	p27KIP1			201549_x_at; 211202_s_at	PLU-1
209112_at	p27KIP1			201783_s_at; 230202_at	RelA (p65 NF-kB subunit)
209112_at	p27KIP1			203358_s_at	EZH2
209112_at	p27KIP1			204254_s_at	VDR
209112_at	p27KIP1			204872_at	TLE4
209112_at	p27KIP1			205899_at	Cyclin A1

IDs in active data set	Object name	miR-7 tgts_miRanda	Transcription Factor	IDs of corresponding network object from GeneGo network	Corresponding network object name
209112_at	p27KIP1			210743_s_at	CDC14a
209112_at	p27KIP1			211716_x_at	RhoGDI alpha
209112_at	p27KIP1			212265_at; 212636_at; 214541_s_at	QKI
209112_at	p27KIP1			220266_s_at; 221841_s_at	KLF4
209112_at	p27KIP1			224851_at	CDK6
209112_at	p27KIP1			225984_at	AMPK alpha 1 subunit
209112_at	p27KIP1			232466_at	Cullin 4A
209822_s_at	VLDLR			201042_at	TGM2
209822_s_at	VLDLR			201109_s_at	Thrombospondin 1
209822_s_at	VLDLR			209676_at; 213258_at	TFPI
209822_s_at	VLDLR			223130_s_at	MIR (Idol)
211607_x_at	EGFR	x		1564907_s_at; 242260_at	Matrin-3
211607_x_at	EGFR	x		200690_at	GRP75
211607_x_at	EGFR	x		200998_s_at; 200999_s_at	CLIMP-63
211607_x_at	EGFR	x		201096_s_at	ARF4
211607_x_at	EGFR	x		201244_s_at	c-Raf-1
211607_x_at	EGFR	x		202267_at	LAMC2
211607_x_at	EGFR	x		203821_at; 38037_at	HB-EGF
211607_x_at	EGFR	x		204011_at	SPRY2

IDs in active data set	Object name	miR-7 tgts_miRanda	Transcription Factor	IDs of corresponding network object from GeneGo network	Corresponding network object name
211607_x_at	EGFR	x		204254_s_at	VDR
211607_x_at	EGFR	x		204686_at	IRS-1
211607_x_at	EGFR	x		205016_at; 211258_s_at	TGF-alpha
211607_x_at	EGFR	x		205447_s_at	ZPK(MAP3K12)
211607_x_at	EGFR	x		206011_at	Caspase-1
211607_x_at	EGFR	x		208958_at	ERp44
211607_x_at	EGFR	x		209409_at	GRB10
211607_x_at	EGFR	x		209615_s_at; 226507_at	PAK1
211607_x_at	EGFR	x		210495_x_at; 211719_x_at; 212464_s_at; 216442_x_at	Fibronectin
211607_x_at	EGFR	x		210517_s_at	AKAP12
211607_x_at	EGFR	x		211063_s_at	NCK1
211607_x_at	EGFR	x		212097_at	Caveolin-1
211607_x_at	EGFR	x		212761_at	TCF7L2 (TCF4)
211607_x_at	EGFR	x		212870_at	SOS2
211607_x_at	EGFR	x		213070_at	PI3K class II (CII-alpha)
211607_x_at	EGFR	x		215485_s_at	ICAM1
211607_x_at	EGFR	x		216971_s_at	Plectin 1
211607_x_at	EGFR	x		218310_at	RABGEF1
211607_x_at	EGFR	x		224657_at	MIG6

IDs in active data set	Object name	miR-7 tgts_miRanda	Transcription Factor	IDs of corresponding network object from GeneGo network	Corresponding network object name
211607_x_at	EGFR	x		224844_at	SLAIN2
211607_x_at	EGFR	x		224938_at	NUFIP2
211607_x_at	EGFR	x		225231_at; 243475_at	c-Cbl
211607_x_at	EGFR	x		226572_at	SOCS7
211607_x_at	EGFR	x		226934_at	CPSF6
211607_x_at	EGFR	x		234932_s_at	CDCP1
211607_x_at	EGFR	x		235057_at	Itch
211607_x_at	EGFR	x		238855_at	AHNAK
211607_x_at	EGFR	x		241755_at	UQCRC2
212761_at	TCF7L2 (TCF4)		x	1552721_a_at; 205117_at; 208240_s_at	FGF1
212761_at	TCF7L2 (TCF4)		x	1555938_x_at	Vimentin
212761_at	TCF7L2 (TCF4)		x	1568574_x_at; 209875_s_at	Osteopontin
212761_at	TCF7L2 (TCF4)		x	200794_x_at	DAZAP2
212761_at	TCF7L2 (TCF4)		x	200952_s_at	Cyclin D2
212761_at	TCF7L2 (TCF4)		x	202859_x_at; 211506_s_at	IL-8
212761_at	TCF7L2 (TCF4)		x	204254_s_at	VDR
212761_at	TCF7L2 (TCF4)		x	205168_at; 225442_at; 227561_at	DDR2
212761_at	TCF7L2 (TCF4)		x	209383_at	C/EBP zeta
212761_at	TCF7L2 (TCF4)		x	210172_at	ZNF162

IDs in active data set	Object name	miR-7 tgts_miRanda	Transcription Factor	IDs of corresponding network object from GeneGo network	Corresponding network object name
212761_at	TCF7L2 (TCF4)		x	210255_at	RAD51B
212761_at	TCF7L2 (TCF4)		x	211527_x_at	VEGF-A
212761_at	TCF7L2 (TCF4)		x	220266_s_at; 221841_s_at	KLF4
212761_at	TCF7L2 (TCF4)		x	222449_at	PMEPA1
212761_at	TCF7L2 (TCF4)		x	224851_at	CDK6
212761_at	TCF7L2 (TCF4)		x	224994_at; 225019_at	CaMK II delta
212761_at	TCF7L2 (TCF4)		x	228938_at	Myelin basic protein
212761_at	TCF7L2 (TCF4)		x	232231_at	RUNX2
212761_at	TCF7L2 (TCF4)		x	235795_at	PAX6
215245_x_at	FMR1			1567213_at; 1567214_a_at; 212037_at	Pinin
215245_x_at	FMR1			202920_at; 202921_s_at	Ankyrin-B
215245_x_at	FMR1			209615_s_at; 226507_at	PAK1
215245_x_at	FMR1			220099_s_at	LUC7L2
215245_x_at	FMR1			220563_s_at	SHANK1
215245_x_at	FMR1			222576_s_at	eIF2C1 (Argonaute-1)
215245_x_at	FMR1			222956_at	Fidgetin
215245_x_at	FMR1			224662_at	KIF5B
215245_x_at	FMR1			224938_at	NUFIP2
215245_x_at	FMR1			225984_at	AMPK alpha 1 subunit

IDs in active data set	Object name	miR-7 tgts_miRanda	Transcription Factor	IDs of corresponding network object from GeneGo network	Corresponding network object name
215245_x_at	FMR1			228938_at	Myelin basic protein
215245_x_at	FMR1			235057_at	Itch
227025_at	PPHLN1			1555938_x_at	Vimentin
227025_at	PPHLN1			201042_at	TGM2
227025_at	PPHLN1			206011_at	Caspase-1
227025_at	PPHLN1			237833_s_at	Synphilin 1

Supplementary Table 8. Hub genes and their targets affected by miR-128 in HEY cells. GeneGo was used to analyze the most significant interactions within the set of differentially expressed genes following miR-128 transfection. The analysis gives a list of "hub" genes interacting with other differentially expressed genes (i.e. nodes) which suggests that miRNAs are regulating the node genes indirectly through regulating the hubs. "IDs in active dataset" refer to the probeset IDs in the dataset that passed the GeneGo FDR threshold of 5% and are acting as hubs. Corresponding gene names/symbols are given in "object name" column. "IDs of corresponding network object from GeneGo network" refer to probeset IDs of objects in the dataset that the hubs are interacting with in GeneGo networks. The names of these nodes are given in the last column. If a hub gene is found to regulate another gene which may also act as a hub then the row is shaded. Genes that are predicted to be miR-128 targets by miRanda or may act as transcription factors are marked with 'x'.

IDs in active data set	Object name	miR-128 tgts_miRanda	Transcription Factor	IDs of corresponding network object from GeneGo network	Corresponding network object name
1552703_s_at; 206011_at; 209970_x_at; 211366_x_at; 211367_s_at; 211368_s_at	Caspase-1	x		1552701_a_at; 1552703_s_at	Pseudo-ICE
1552703_s_at; 206011_at; 209970_x_at; 211366_x_at; 211367_s_at; 211368_s_at	Caspase-1	x		1554091_a_at	TIRAP (Mal)
1552703_s_at; 206011_at; 209970_x_at; 211366_x_at; 211367_s_at; 211368_s_at	Caspase-1	x		1554479_a_at; 204950_at	CARD8

IDs in active data set	Object name	miR-128 tgts_miRanda	Transcription Factor	IDs of corresponding network object from GeneGo network	Corresponding network object name
1552703_s_at; 206011_at; 209970_x_at; 211366_x_at; 211367_s_at; 211368_s_at	Caspase-1	x		1552701_a_at; 1552703_s_at	Pseudo-ICE
1552703_s_at; 206011_at; 209970_x_at; 211366_x_at; 211367_s_at; 211368_s_at	Caspase-1	x		1555543_a_at; 207855_s_at	CLCC1
1552703_s_at; 206011_at; 209970_x_at; 211366_x_at; 211367_s_at; 211368_s_at	Caspase-1	x		1559893_at; 228495_at	CCDC75
1552703_s_at; 206011_at; 209970_x_at; 211366_x_at; 211367_s_at; 211368_s_at	Caspase-1	x		1567014_s_at; 1567015_at	NRF2
1552703_s_at; 206011_at; 209970_x_at; 211366_x_at; 211367_s_at; 211368_s_at	Caspase-1	x		1570507_at	SFRS2IP
1552703_s_at; 206011_at; 209970_x_at; 211366_x_at; 211367_s_at; 211368_s_at	Caspase-1	x		201841_s_at	HSP27
1552703_s_at; 206011_at; 209970_x_at; 211366_x_at; 211367_s_at; 211368_s_at	Caspase-1	x		203275_at	IRF2
1552703_s_at; 206011_at; 209970_x_at;	Caspase-1	x		205067_at; 39402_at	IL-1 beta

IDs in active data set	Object name	miR-128 tgts_miRanda	Transcription Factor	IDs of corresponding network object from GeneGo network	Corresponding network object name
1552703_s_at; 206011_at; 209970_x_at; 211366_x_at; 211367_s_at; 211368_s_at	Caspase-1	x		1552701_a_at; 1552703_s_at	Pseudo-ICE
211366_x_at; 211367_s_at; 211368_s_at					
1552703_s_at; 206011_at; 209970_x_at; 211366_x_at; 211367_s_at; 211368_s_at	Caspase-1	x		205415_s_at	MJD (ataxin-3)
1552703_s_at; 206011_at; 209970_x_at; 211366_x_at; 211367_s_at; 211368_s_at	Caspase-1	x		205425_at	HIP1
1552703_s_at; 206011_at; 209970_x_at; 211366_x_at; 211367_s_at; 211368_s_at	Caspase-1	x		205996_s_at; 212173_at; 212174_at; 212175_s_at	AK2
1552703_s_at; 206011_at; 209970_x_at; 211366_x_at; 211367_s_at; 211368_s_at	Caspase-1	x		207181_s_at	Caspase-7
1552703_s_at; 206011_at; 209970_x_at; 211366_x_at; 211367_s_at; 211368_s_at	Caspase-1	x		208374_s_at	CAPZA1
1552703_s_at; 206011_at; 209970_x_at; 211366_x_at; 211367_s_at; 211368_s_at	Caspase-1	x		209615_s_at	PAK1

IDs in active data set	Object name	miR-128 tgts_miRanda	Transcription Factor	IDs of corresponding network object from GeneGo network	Corresponding network object name
1552703_s_at; 206011_at; 209970_x_at; 211366_x_at; 211367_s_at; 211368_s_at	Caspase-1	x		1552701_a_at; 1552703_s_at	Pseudo-ICE
1552703_s_at; 206011_at; 209970_x_at; 211366_x_at; 211367_s_at; 211368_s_at	Caspase-1	x		209822_s_at	VLDLR
1552703_s_at; 206011_at; 209970_x_at; 211366_x_at; 211367_s_at; 211368_s_at	Caspase-1	x		210113_s_at	CARD7
1552703_s_at; 206011_at; 209970_x_at; 211366_x_at; 211367_s_at; 211368_s_at	Caspase-1	x		210145_at	PA24A
1552703_s_at; 206011_at; 209970_x_at; 211366_x_at; 211367_s_at; 211368_s_at	Caspase-1	x		211995_x_at; 212988_x_at	Actin cytoplasmic 2
1552703_s_at; 206011_at; 209970_x_at; 211366_x_at; 211367_s_at; 211368_s_at	Caspase-1	x		213012_at	NEDD4
1552703_s_at; 206011_at; 209970_x_at; 211366_x_at; 211367_s_at; 211368_s_at	Caspase-1	x		213596_at	Caspase-4
1552703_s_at; 206011_at; 209970_x_at;	Caspase-1	x		214291_at	RPL17

IDs in active data set	Object name	miR-128 tgts_miRanda	Transcription Factor	IDs of corresponding network object from GeneGo network	Corresponding network object name
1552703_s_at; 206011_at; 209970_x_at; 211366_x_at; 211367_s_at; 211368_s_at	Caspase-1	x		1552701_a_at; 1552703_s_at	Pseudo-ICE
211366_x_at; 211367_s_at; 211368_s_at					
1552703_s_at; 206011_at; 209970_x_at; 211366_x_at; 211367_s_at; 211368_s_at	Caspase-1	x		216632_at	NAV3
1552703_s_at; 206011_at; 209970_x_at; 211366_x_at; 211367_s_at; 211368_s_at	Caspase-1	x		218943_s_at	RIG-I
1552703_s_at; 206011_at; 209970_x_at; 211366_x_at; 211367_s_at; 211368_s_at	Caspase-1	x		222036_s_at; 222037_at	MCM4
1552703_s_at; 206011_at; 209970_x_at; 211366_x_at; 211367_s_at; 211368_s_at	Caspase-1	x		224782_at	ZMAT2
1552703_s_at; 206011_at; 209970_x_at; 211366_x_at; 211367_s_at; 211368_s_at	Caspase-1	x		225386_s_at	SRRF
1552703_s_at; 206011_at; 209970_x_at; 211366_x_at; 211367_s_at; 211368_s_at	Caspase-1	x		238034_at	Calnexin

IDs in active data set	Object name	miR-128 tgts_miRanda	Transcription Factor	IDs of corresponding network object from GeneGo network	Corresponding network object name
1552703_s_at; 206011_at; 209970_x_at; 211366_x_at; 211367_s_at; 211368_s_at	Caspase-1	x		1552701_a_at; 1552703_s_at	Pseudo-ICE
1552703_s_at; 206011_at; 209970_x_at; 211366_x_at; 211367_s_at; 211368_s_at	Caspase-1	x		242260_at	Matrin-3
1554168_a_at; 223082_at; 235692_at	CIN85			1554086_at	TUBGCP3
1554168_a_at; 223082_at; 235692_at	CIN85			201896_s_at	DDA3
1554168_a_at; 223082_at; 235692_at	CIN85			204318_s_at	GTSE1
1554168_a_at; 223082_at; 235692_at	CIN85			208151_x_at; 208719_s_at; 213998_s_at	DDX17
1554168_a_at; 223082_at; 235692_at	CIN85			208374_s_at	CAPZA1
1554168_a_at; 223082_at; 235692_at	CIN85			208865_at; 208866_at; 208867_s_at	Casein kinase I alpha
1554168_a_at; 223082_at; 235692_at	CIN85			208877_at; 208878_s_at	PAK2
1554168_a_at; 223082_at; 235692_at	CIN85			209682_at	CBL-B
1554168_a_at; 223082_at; 235692_at	CIN85			210612_s_at	SYNJ2
1554168_a_at; 223082_at; 235692_at	CIN85			210684_s_at	PSD-95
1554168_a_at; 223082_at; 235692_at	CIN85			213012_at	NEDD4

IDs in active data set	Object name	miR-128 tgts_miRanda	Transcription Factor	IDs of corresponding network object from GeneGo network	Corresponding network object name
1552703_s_at; 206011_at; 209970_x_at; 211366_x_at; 211367_s_at; 211368_s_at	Caspase-1	x		1552701_a_at; 1552703_s_at	Pseudo-ICE
1554168_a_at; 223082_at; 235692_at	CIN85			213649_at	SFRS7 (9G8)
1554168_a_at; 223082_at; 235692_at	CIN85			213976_at	CIZ1
1554168_a_at; 223082_at; 235692_at	CIN85			215606_s_at	ELKS
1554168_a_at; 223082_at; 235692_at	CIN85			222077_s_at	RacGAP1
1554168_a_at; 223082_at; 235692_at	CIN85			223208_at; 226518_at	KCTD10
1554168_a_at; 223082_at; 235692_at	CIN85			225186_at; 225188_at	Lpd
1554168_a_at; 223082_at; 235692_at	CIN85			225521_at	ANAPC7
1554168_a_at; 223082_at; 235692_at	CIN85			226020_s_at	DAB1
1554168_a_at; 223082_at; 235692_at	CIN85			235430_at	C14orf43
1554168_a_at; 223082_at; 235692_at	CIN85			239651_at	ANAPC5
1565483_at	EGFR			1552703_s_at; 206011_at; 209970_x_at; 211366_x_at; 211367_s_at; 211368_s_at	Caspase-1
1565483_at	EGFR			1554168_a_at; 223082_at; 235692_at	CIN85

**IDs in active data set**	**Object name**	**miR-128 tgts_miRanda**	**Transcription Factor**	**IDs of corresponding network object from GeneGo network**	**Corresponding network object name**
1552703_s_at; 206011_at; 209970_x_at; 211366_x_at; 211367_s_at; 211368_s_at	Caspase-1	x		1552701_a_at; 1552703_s_at	Pseudo-ICE
1565483_at	EGFR			1554679_a_at; 208029_s_at; 208767_s_at	LAPTM4B
1565483_at	EGFR			1557719_at	PIP5KIII
1565483_at	EGFR			1558199_at	Fibronectin
1565483_at	EGFR			1558532_at	Tropomyosin-1
1565483_at	EGFR			1567014_s_at; 1567015_at	NRF2
1565483_at	EGFR			1568765_at; 202627_s_at; 202628_s_at	PAI1
1565483_at	EGFR			1569385_s_at	TET2
1565483_at	EGFR			200021_at	Cofilin, non-muscle
1565483_at	EGFR			200726_at	PP1-cat gamma
1565483_at	EGFR			200824_at	GSTP1
1565483_at	EGFR			200894_s_at; 200895_s_at	FKBP4
1565483_at	EGFR			201087_at; 211823_s_at	Paxillin
1565483_at	EGFR			201373_at; 216971_s_at	Plectin 1
1565483_at	EGFR			201693_s_at; 201694_s_at; 227404_s_at	EGR1
1565483_at	EGFR			201840_at	NEDD8
1565483_at	EGFR			201841_s_at	HSP27
1565483_at	EGFR			202267_at; 207517_at	LAMC2
1565483_at	EGFR			202637_s_at; 202638_s_at; 215485_s_at	ICAM1
1565483_at	EGFR			202670_at	MEK1(MAP2K1)
1565483_at	EGFR			202677_at	p120GAP
1565483_at	EGFR			203010_at	STAT5A
1565483_at	EGFR			203065_s_at; 212097_at	Caveolin-1
1565483_at	EGFR			203276_at	Lamin B1
1565483_at	EGFR			203373_at	SOCS2
1565483_at	EGFR			203431_s_at	p200RhoGAP

IDs in active data set	Object name	miR-128 tgts_miRanda	Transcription Factor	IDs of corresponding network object from GeneGo network	Corresponding network object name
1552703_s_at; 206011_at; 209970_x_at; 211366_x_at; 211367_s_at; 211368_s_at	Caspase-1	x		1552701_a_at; 1552703_s_at	Pseudo-ICE
1565483_at	EGFR			203688_at	Polycystin 2
1565483_at	EGFR			204254_s_at; 204255_s_at; 213692_s_at	VDR
1565483_at	EGFR			204620_s_at; 211571_s_at; 215646_s_at; 221731_x_at	Versican
1565483_at	EGFR			204654_s_at	AP-2A
1565483_at	EGFR			205015_s_at; 211258_s_at	TGF-alpha
1565483_at	EGFR			205032_at; 227314_at	ITGA2
1565483_at	EGFR			205192_at	NIK(MAP3K14)
1565483_at	EGFR			205425_at	HIP1
1565483_at	EGFR			205447_s_at	ZPK(MAP3K12)
1565483_at	EGFR			205645_at	REPS2
1565483_at	EGFR			205810_s_at	N-WASP
1565483_at	EGFR			206204_at	GRB14
1565483_at	EGFR			206348_s_at	PDK3
1565483_at	EGFR			206412_at	Fer
1565483_at	EGFR			206846_s_at	HDAC6
1565483_at	EGFR			207112_s_at; 225998_at	GAB1
1565483_at	EGFR			207181_s_at	Caspase-7
1565483_at	EGFR			208062_s_at	Neuregulin 2
1565483_at	EGFR			208194_s_at; 209649_at; 215044_s_at; 228254_at; 242569_at	STAM2
1565483_at	EGFR			208546_x_at	H2BFQ
1565483_at	EGFR			208553_at	HIST1H1E
1565483_at	EGFR			208812_x_at; 214459_x_at	HLA-Cw3
1565483_at	EGFR			208832_at; 208833_s_at	Ataxin-10
1565483_at	EGFR			208977_x_at; 213726_x_at	Tubulin beta 2C

IDs in active data set	Object name	miR-128 tgts_miRanda	Transcription Factor	IDs of corresponding network object from GeneGo network	Corresponding network object name
1552703_s_at; 206011_at; 209970_x_at; 211366_x_at; 211367_s_at; 211368_s_at	Caspase-1	x		1552701_a_at; 1552703_s_at	Pseudo-ICE
1565483_at	EGFR			208991_at; 208992_s_at; 225289_at	STAT3
1565483_at	EGFR			209251_x_at; 211750_x_at	Tubulin alpha-1C
1565483_at	EGFR			209332_s_at	Max
1565483_at	EGFR			209615_s_at	PAK1
1565483_at	EGFR			209633_at	PPP2R3A
1565483_at	EGFR			209648_x_at	SOCS5
1565483_at	EGFR			209682_at	CBL-B
1565483_at	EGFR			210173_at; 214137_at; 227396_at	DEP-1
1565483_at	EGFR			210449_x_at; 211561_x_at	p38alpha (MAPK14)
1565483_at	EGFR			211596_s_at	LIG-1
1565483_at	EGFR			211928_at	Dynein 1, cytoplasmic, heavy chain
1565483_at	EGFR			211986_at	AHNAK
1565483_at	EGFR			212073_at	Casein kinase II, alpha chain (CSNK2A1)
1565483_at	EGFR			212100_s_at; 215357_s_at	PDIP46
1565483_at	EGFR			212362_at	Ca-ATPase2
1565483_at	EGFR			212426_s_at	14-3-3 theta
1565483_at	EGFR			212549_at	STAT5B
1565483_at	EGFR			213003_s_at	C/EBPdelta
1565483_at	EGFR			213070_at	PI3K class II (CII-alpha)
1565483_at	EGFR			213324_at	c-Src
1565483_at	EGFR			214015_at; 226572_at; 228662_at	SOCS7
1565483_at	EGFR			214132_at	ATP5C
1565483_at	EGFR			214744_s_at	RPL23
1565483_at	EGFR			216990_at; 230097_at;	PUR2

IDs in active data set	Object name	miR-128 tgts_miRanda	Transcription Factor	IDs of corresponding network object from GeneGo network	Corresponding network object name
1552703_s_at; 206011_at; 209970_x_at; 211366_x_at; 211367_s_at; 211368_s_at	Caspase-1	x		1552701_a_at; 1552703_s_at	Pseudo-ICE
				244822_at	
1565483_at	EGFR			223679_at	Beta-catenin
1565483_at	EGFR			224221_s_at	VAV-3
1565483_at	EGFR			224853_at; 224854_s_at	SLAIN2
1565483_at	EGFR			225396_at; 237333_at; 244872_at	RBBP4 (RbAp48)
1565483_at	EGFR			226404_at	CAPER
1565483_at	EGFR			226408_at	TEF-4
1565483_at	EGFR			226713_at; 228693_at; 235051_at; 236831_at	YMER
1565483_at	EGFR			227394_at	NCAM1
1565483_at	EGFR			227625_s_at	CHIP
1565483_at	EGFR			227997_at	IL17RD
1565483_at	EGFR			228353_x_at; 238462_at	Sts-1
1565483_at	EGFR			229141_at; 243832_at	WDR33
1565483_at	EGFR			232505_at	DCTN2
1565483_at	EGFR			237856_at	Rap1GDS1
1565483_at	EGFR			238759_at	Girdin
1565483_at	EGFR			242260_at	Matrin-3
1565483_at	EGFR			242482_at	PRKAR1A
200711_s_at; 200719_at	SKP1	x		200726_at	PP1-cat gamma
200711_s_at; 200719_at	SKP1	x		201316_at	PSMA2
200711_s_at; 200719_at	SKP1	x		201502_s_at	NFKBIA
200711_s_at; 200719_at	SKP1	x		201693_s_at; 201694_s_at; 227404_s_at	EGR1
200711_s_at; 200719_at	SKP1	x		201897_s_at	CKS1
200711_s_at; 200719_at	SKP1	x		201908_at	DVL-3

IDs in active data set	Object name	miR-128 tgts_miRanda	Transcription Factor	IDs of corresponding network object from GeneGo network	Corresponding network object name
1552703_s_at; 206011_at; 209970_x_at; 211366_x_at; 211367_s_at; 211368_s_at	Caspase-1	x		1552701_a_at; 1552703_s_at	Pseudo-ICE
200711_s_at; 200719_at	SKP1	x		204826_at	Cyclin F
200711_s_at; 200719_at	SKP1	x		208838_at; 208839_s_at	TIP120A
200711_s_at; 200719_at	SKP1	x		208988_at; 208989_s_at	FBXL11
200711_s_at; 200719_at	SKP1	x		209630_s_at	FBXW2
200711_s_at; 200719_at	SKP1	x		213359_at; 221480_at	AUF1
200711_s_at; 200719_at	SKP1	x		218432_at; 229955_at	Fbxo3
200711_s_at; 200719_at	SKP1	x		221436_s_at	Tome-1
200711_s_at; 200719_at	SKP1	x		225099_at; 242294_at	FBXO45
200711_s_at; 200719_at	SKP1	x		225803_at; 241763_s_at	MAFbx
200711_s_at; 200719_at	SKP1	x		241747_s_at	Cullin7
200726_at	PP1-cat gamma	x		1559044_at	CSL4
200726_at	PP1-cat gamma	x		1570352_at	ATM
200726_at	PP1-cat gamma	x		200021_at	Cofilin, non-muscle
200726_at	PP1-cat gamma	x		201702_s_at	PNUTS
200726_at	PP1-cat gamma	x		202094_at; 202095_s_at	Survivin
200726_at	PP1-cat gamma	x		204554_at	PPP1R3D
200726_at	PP1-cat gamma	x		204641_at; 211080_s_at	Nek2A
200726_at	PP1-cat gamma	x		204839_at	POP5
200726_at	PP1-cat gamma	x		206665_s_at; 212312_at	Bcl-XL
200726_at	PP1-cat	x		209090_s_at	Endophilin B1

IDs in active data set	Object name	miR-128 tgts_miRanda	Transcription Factor	IDs of corresponding network object from GeneGo network	Corresponding network object name
1552703_s_at; 206011_at; 209970_x_at; 211366_x_at; 211367_s_at; 211368_s_at	Caspase-1	x		1552701_a_at; 1552703_s_at	Pseudo-ICE
	gamma				
200726_at	PP1-cat gamma	x		209332_s_at	Max
200726_at	PP1-cat gamma	x		209339_at	SIAH2
200726_at	PP1-cat gamma	x		209482_at	RPP20
200726_at	PP1-cat gamma	x		212020_s_at; 212021_s_at; 212023_s_at	Antigen KI-67
200726_at	PP1-cat gamma	x		215119_at	MYR8
200726_at	PP1-cat gamma	x		219235_s_at	Phactr4
200726_at	PP1-cat gamma	x		227696_at	MTR3
200726_at	PP1-cat gamma	x		231966_at	Neurabin-1
200726_at	PP1-cat gamma	x		232097_at	TOX4
200732_s_at	PTP4A1			201090_x_at; 212639_x_at; 213646_x_at	Tubulin alpha-1B
200732_s_at	PTP4A1			201693_s_at; 201694_s_at; 227404_s_at	EGR1
200732_s_at	PTP4A1			206684_s_at; 228830_s_at; 235160_at; 244587_at	ATF-7
200732_s_at	PTP4A1			208296_x_at; 210260_s_at	TNFAIP8
200732_s_at	PTP4A1			208615_s_at	PTP4A2
200732_s_at	PTP4A1			225565_at; 225572_at	CREB1
200732_s_at	PTP4A1			238487_at; 239068_at	GNL1
200790_at	DCOR			201365_at	OAZ2
200790_at	DCOR			201467_s_at;	NQO1

IDs in active data set	Object name	miR-128 tgts_miRanda	Transcription Factor	IDs of corresponding network object from GeneGo network	Corresponding network object name
1552703_s_at; 206011_at; 209970_x_at; 211366_x_at; 211367_s_at; 211368_s_at	Caspase-1	x		1552701_a_at; 1552703_s_at	Pseudo-ICE
				201468_s_at; 210519_s_at	
200790_at	DCOR			204109_s_at; 228433_at	NFYA
200790_at	DCOR			204654_s_at	AP-2A
200790_at	DCOR			206877_at; 228846_at	MAD
200790_at	DCOR			209332_s_at	Max
200790_at	DCOR			218284_at	SMAD3
200790_at	DCOR			220266_s_at	KLF4
200790_at	DCOR			230821_at	ZNF148
200894_s_at; 200895_s_at	FKBP4			204186_s_at; 228469_at	Cyclophilin D
200894_s_at; 200895_s_at	FKBP4			204466_s_at; 207827_x_at; 211546_x_at	Alpha-synuclein
200894_s_at; 200895_s_at	FKBP4			207181_s_at	Caspase-7
200894_s_at; 200895_s_at	FKBP4			212073_at	Casein kinase II, alpha chain (CSNK2A1)
200894_s_at; 200895_s_at	FKBP4			213070_at	PI3K class II (CII-alpha)
200894_s_at; 200895_s_at	FKBP4			217728_at	Calcyclin
200894_s_at; 200895_s_at	FKBP4			219343_at; 228561_at	CDC37L1
200894_s_at; 200895_s_at	FKBP4			226336_at	Cyclophilin A
200894_s_at; 200895_s_at	FKBP4			232505_at	DCTN2
200894_s_at; 200895_s_at	FKBP4			233748_x_at	AMPK gamma2
201087_at; 211823_s_at	Paxillin			1555842_at	Cytohesin2
201087_at; 211823_s_at	Paxillin			1555878_at	RPS24
201087_at; 211823_s_at	Paxillin			1556499_s_at; 202310_s_at	COL1A1

IDs in active data set	Object name	miR-128 tgts_miRanda	Transcription Factor	IDs of corresponding network object from GeneGo network	Corresponding network object name
1552703_s_at; 206011_at; 209970_x_at; 211366_x_at; 211367_s_at; 211368_s_at	Caspase-1	x		1552701_a_at; 1552703_s_at	Pseudo-ICE
201087_at; 211823_s_at	Paxillin			1558199_at	Fibronectin
201087_at; 211823_s_at	Paxillin			1558532_at	Tropomyosin-1
201087_at; 211823_s_at	Paxillin			1565717_s_at	LXR-alpha
201087_at; 211823_s_at	Paxillin			1567223_at; 1567224_at	HMGA2
201087_at; 211823_s_at	Paxillin			200072_s_at	hnRNP M
201087_at; 211823_s_at	Paxillin			201042_at; 211003_x_at; 211573_x_at	TGM2
201087_at; 211823_s_at	Paxillin			201714_at	Tubulin gamma 1
201087_at; 211823_s_at	Paxillin			202149_at; 202150_s_at	CAS-L
201087_at; 211823_s_at	Paxillin			202441_at; 202444_s_at	SPFH1
201087_at; 211823_s_at	Paxillin			202677_at	p120GAP
201087_at; 211823_s_at	Paxillin			204626_s_at; 204627_s_at; 204628_s_at; 216261_at	ITGB3
201087_at; 211823_s_at	Paxillin			205369_x_at; 244687_at	BCKD-E2
201087_at; 211823_s_at	Paxillin			205645_at	REPS2
201087_at; 211823_s_at	Paxillin			207112_s_at; 225998_at	GAB1
201087_at; 211823_s_at	Paxillin			208977_x_at; 213726_x_at	Tubulin beta 2C
201087_at; 211823_s_at	Paxillin			209589_s_at	Ephrin-B receptor 2
201087_at; 211823_s_at	Paxillin			210173_at; 214137_at; 227396_at	DEP-1
201087_at;	Paxillin			210477_x_at;	JNK1(MAPK8)

IDs in active data set	Object name	miR-128 tgts_miRanda	Transcription Factor	IDs of corresponding network object from GeneGo network	Corresponding network object name
1552703_s_at; 206011_at; 209970_x_at; 211366_x_at; 211367_s_at; 211368_s_at	Caspase-1	x		1552701_a_at; 1552703_s_at	Pseudo-ICE
211823_s_at				226048_at	
201087_at; 211823_s_at	Paxillin			211711_s_at; 242622_x_at	PTEN
201087_at; 211823_s_at	Paxillin			211995_x_at; 212988_x_at	Actin cytoplasmic 2
201087_at; 211823_s_at	Paxillin			212205_at; 212206_s_at	H2AFV
201087_at; 211823_s_at	Paxillin			212372_at; 213067_at	MYH10
201087_at; 211823_s_at	Paxillin			212426_s_at	14-3-3 theta
201087_at; 211823_s_at	Paxillin			213746_s_at; 214752_x_at	Filamin A
201087_at; 211823_s_at	Paxillin			214073_at	Cortactin
201087_at; 211823_s_at	Paxillin			214607_at	PAK3
201087_at; 211823_s_at	Paxillin			214744_s_at	RPL23
201087_at; 211823_s_at	Paxillin			221725_at; 224562_at; 224563_at	WASF2
201087_at; 211823_s_at	Paxillin			235412_at	BETA-PIX
201090_x_at; 212639_x_at; 213646_x_at	Tubulin alpha-1B			1565483_at	EGFR
201090_x_at; 212639_x_at; 213646_x_at	Tubulin alpha-1B			200894_s_at; 200895_s_at	FKBP4
201090_x_at; 212639_x_at; 213646_x_at	Tubulin alpha-1B			201087_at; 211823_s_at	Paxillin
201090_x_at; 212639_x_at; 213646_x_at	Tubulin alpha-1B			201502_s_at	NFKBIA
201090_x_at; 212639_x_at; 213646_x_at	Tubulin alpha-1B			201714_at	Tubulin gamma 1

IDs in active data set	Object name	miR-128 tgts_miRanda	Transcription Factor	IDs of corresponding network object from GeneGo network	Corresponding network object name
1552703_s_at; 206011_at; 209970_x_at; 211366_x_at; 211367_s_at; 211368_s_at	Caspase-1	x		1552701_a_at; 1552703_s_at	Pseudo-ICE
201090_x_at; 212639_x_at; 213646_x_at	Tubulin alpha-1B			202154_x_at; 213476_x_at	Tubulin beta 3
201090_x_at; 212639_x_at; 213646_x_at	Tubulin alpha-1B			203463_s_at	EPN2
201090_x_at; 212639_x_at; 213646_x_at	Tubulin alpha-1B			204270_at	Ski
201090_x_at; 212639_x_at; 213646_x_at	Tubulin alpha-1B			204466_s_at; 207827_x_at; 211546_x_at	Alpha-synuclein
201090_x_at; 212639_x_at; 213646_x_at	Tubulin alpha-1B			205647_at; 210630_s_at	Rad52
201090_x_at; 212639_x_at; 213646_x_at	Tubulin alpha-1B			206846_s_at	HDAC6
201090_x_at; 212639_x_at; 213646_x_at	Tubulin alpha-1B			210282_at	ZNF198
201090_x_at; 212639_x_at; 213646_x_at	Tubulin alpha-1B			210684_s_at	PSD-95
201090_x_at; 212639_x_at; 213646_x_at	Tubulin alpha-1B			221264_s_at	TARDBP (TDP43)
201090_x_at; 212639_x_at; 213646_x_at	Tubulin alpha-1B			222077_s_at	RacGAP1
201090_x_at; 212639_x_at; 213646_x_at	Tubulin alpha-1B			226563_at; 235598_at	SMAD2
201502_s_at	NFKBIA			201042_at; 211003_x_at; 211573_x_at	TGM2
201502_s_at	NFKBIA			201316_at	PSMA2
201502_s_at	NFKBIA			201792_at	AEBP1
201502_s_at	NFKBIA			202491_s_at	IKAP

IDs in active data set	Object name	miR-128 tgts_miRanda	Transcription Factor	IDs of corresponding network object from GeneGo network	Corresponding network object name
1552703_s_at; 206011_at; 209970_x_at; 211366_x_at; 211367_s_at; 211368_s_at	Caspase-1	x		1552701_a_at; 1552703_s_at	Pseudo-ICE
201502_s_at	NFKBIA			202672_s_at	ATF-3
201502_s_at	NFKBIA			202762_at	ROCK2
201502_s_at	NFKBIA			205192_at	NIK(MAP3K14)
201502_s_at	NFKBIA			205415_s_at	MJD (ataxin-3)
201502_s_at	NFKBIA			205731_s_at; 205732_s_at	NCOA2 (GRIP1/TIF2)
201502_s_at	NFKBIA			206967_at	Cyclin T1
201502_s_at	NFKBIA			209341_s_at	IKK-beta
201502_s_at	NFKBIA			210426_x_at; 210479_s_at; 226682_at; 235567_at; 236266_at	ROR-alpha
201502_s_at	NFKBIA			211764_s_at; 214590_s_at	UBE2D1
201502_s_at	NFKBIA			211995_x_at; 212988_x_at	Actin cytoplasmic 2
201502_s_at	NFKBIA			212073_at	Casein kinase II, alpha chain (CSNK2A1)
201502_s_at	NFKBIA			212521_s_at	PDE8A
201502_s_at	NFKBIA			214744_s_at	RPL23
201502_s_at	NFKBIA			215606_s_at	ELKS
201502_s_at	NFKBIA			218832_x_at	Beta-arrestin1
201502_s_at	NFKBIA			221562_s_at	Sirtuin3
201502_s_at	NFKBIA			226648_at	FIH-1
201502_s_at	NFKBIA			227075_at	ELP3
201502_s_at	NFKBIA			229515_at	Par-4
201693_s_at; 201694_s_at; 227404_s_at	EGR1		x	1553127_a_at	RNF168
201693_s_at; 201694_s_at; 227404_s_at	EGR1		x	1554408_a_at	TK1
201693_s_at; 201694_s_at; 227404_s_at	EGR1		x	1555832_s_at	KLF6
201693_s_at; 201694_s_at;	EGR1		x	1557352_at	ERG1

IDs in active data set	Object name	miR-128 tgts_miRanda	Transcription Factor	IDs of corresponding network object from GeneGo network	Corresponding network object name
1552703_s_at; 206011_at; 209970_x_at; 211366_x_at; 211367_s_at; 211368_s_at	Caspase-1	x		1552701_a_at; 1552703_s_at	Pseudo-ICE
227404_s_at					
201693_s_at; 201694_s_at; 227404_s_at	EGR1		x	1558199_at	Fibronectin
201693_s_at; 201694_s_at; 227404_s_at	EGR1		x	1558233_s_at; 1565269_s_at	ATF-1
201693_s_at; 201694_s_at; 227404_s_at	EGR1		x	1568574_x_at	Osteopontin
201693_s_at; 201694_s_at; 227404_s_at	EGR1		x	1568765_at; 202627_s_at; 202628_s_at	PAI1
201693_s_at; 201694_s_at; 227404_s_at	EGR1		x	1569203_at	GRO-2
201693_s_at; 201694_s_at; 227404_s_at	EGR1		x	200632_s_at	NDRG1
201693_s_at; 201694_s_at; 227404_s_at	EGR1		x	200658_s_at	Prohibitin
201693_s_at; 201694_s_at; 227404_s_at	EGR1		x	200783_s_at	Stathmin
201693_s_at; 201694_s_at; 227404_s_at	EGR1		x	200952_s_at; 200953_s_at	Cyclin D2
201693_s_at; 201694_s_at; 227404_s_at	EGR1		x	201042_at; 211003_x_at; 211573_x_at	TGM2
201693_s_at; 201694_s_at; 227404_s_at	EGR1		x	201313_at	ENO2
201693_s_at; 201694_s_at; 227404_s_at	EGR1		x	201324_at; 201325_s_at; 213895_at	EMP1 (Tmp)
201693_s_at; 201694_s_at;	EGR1		x	201373_at; 216971_s_at	Plectin 1

IDs in active data set	Object name	miR-128 tgts_miRanda	Transcription Factor	IDs of corresponding network object from GeneGo network	Corresponding network object name
1552703_s_at; 206011_at; 209970_x_at; 211366_x_at; 211367_s_at; 211368_s_at	Caspase-1	x		1552701_a_at; 1552703_s_at	Pseudo-ICE
227404_s_at					
201693_s_at; 201694_s_at; 227404_s_at	EGR1		x	201549_x_at	PLU-1
201693_s_at; 201694_s_at; 227404_s_at	EGR1		x	201642_at	IFNGR2
201693_s_at; 201694_s_at; 227404_s_at	EGR1		x	201666_at	TIMP1
201693_s_at; 201694_s_at; 227404_s_at	EGR1		x	201714_at	Tubulin gamma 1
201693_s_at; 201694_s_at; 227404_s_at	EGR1		x	202068_s_at	LDLR
201693_s_at; 201694_s_at; 227404_s_at	EGR1		x	202094_at; 202095_s_at	Survivin
201693_s_at; 201694_s_at; 227404_s_at	EGR1		x	202304_at; 238961_s_at; 241611_s_at	Fndc3a
201693_s_at; 201694_s_at; 227404_s_at	EGR1		x	202314_at	CYP51A1
201693_s_at; 201694_s_at; 227404_s_at	EGR1		x	202441_at; 202444_s_at	SPFH1
201693_s_at; 201694_s_at; 227404_s_at	EGR1		x	202517_at	CRMP1
201693_s_at; 201694_s_at; 227404_s_at	EGR1		x	202637_s_at; 202638_s_at; 215485_s_at	ICAM1
201693_s_at; 201694_s_at; 227404_s_at	EGR1		x	202672_s_at	ATF-3
201693_s_at; 201694_s_at;	EGR1		x	202727_s_at; 211676_s_at	IFNGR1

IDs in active data set	Object name	miR-128 tgts_miRanda	Transcription Factor	IDs of corresponding network object from GeneGo network	Corresponding network object name
1552703_s_at; 206011_at; 209970_x_at; 211366_x_at; 211367_s_at; 211368_s_at	Caspase-1	x		1552701_a_at; 1552703_s_at	Pseudo-ICE
227404_s_at					
201693_s_at; 201694_s_at; 227404_s_at	EGR1		x	202779_s_at	UBE2S
201693_s_at; 201694_s_at; 227404_s_at	EGR1		x	203504_s_at; 203505_at; 216066_at	ABCA1
201693_s_at; 201694_s_at; 227404_s_at	EGR1		x	204109_s_at; 228433_at	NFYA
201693_s_at; 201694_s_at; 227404_s_at	EGR1		x	204279_at	PSMB9
201693_s_at; 201694_s_at; 227404_s_at	EGR1		x	204363_at	Tissue factor
201693_s_at; 201694_s_at; 227404_s_at	EGR1		x	204654_s_at	AP-2A
201693_s_at; 201694_s_at; 227404_s_at	EGR1		x	204933_s_at	Osteoprotegerin
201693_s_at; 201694_s_at; 227404_s_at	EGR1		x	204980_at	CLOCK
201693_s_at; 201694_s_at; 227404_s_at	EGR1		x	205067_at; 39402_at	IL-1 beta
201693_s_at; 201694_s_at; 227404_s_at	EGR1		x	205463_s_at; 216867_s_at	PDGF-A
201693_s_at; 201694_s_at; 227404_s_at	EGR1		x	206377_at	FOXF2
201693_s_at; 201694_s_at; 227404_s_at	EGR1		x	206877_at; 228846_at	MAD
201693_s_at; 201694_s_at;	EGR1		x	207181_s_at	Caspase-7

IDs in active data set	Object name	miR-128 tgts_miRanda	Transcription Factor	IDs of corresponding network object from GeneGo network	Corresponding network object name
1552703_s_at; 206011_at; 209970_x_at; 211366_x_at; 211367_s_at; 211368_s_at	Caspase-1	x		1552701_a_at; 1552703_s_at	Pseudo-ICE
227404_s_at					
201693_s_at; 201694_s_at; 227404_s_at	EGR1		x	208786_s_at	MAP1LC3B
201693_s_at; 201694_s_at; 227404_s_at	EGR1		x	209277_at; 209278_s_at	TFPI-2
201693_s_at; 201694_s_at; 227404_s_at	EGR1		x	209307_at	SWAP-70
201693_s_at; 201694_s_at; 227404_s_at	EGR1		x	209332_s_at	Max
201693_s_at; 201694_s_at; 227404_s_at	EGR1		x	209408_at	MCAK
201693_s_at; 201694_s_at; 227404_s_at	EGR1		x	209457_at	DUSP5
201693_s_at; 201694_s_at; 227404_s_at	EGR1		x	209466_x_at; 211737_x_at	Pleiotrophin (OSF1)
201693_s_at; 201694_s_at; 227404_s_at	EGR1		x	209955_s_at	Seprase
201693_s_at; 201694_s_at; 227404_s_at	EGR1		x	210426_x_at; 210479_s_at; 226682_at; 235567_at; 236266_at	ROR-alpha
201693_s_at; 201694_s_at; 227404_s_at	EGR1		x	210512_s_at; 211527_x_at	VEGF-A
201693_s_at; 201694_s_at; 227404_s_at	EGR1		x	210947_s_at	MSH3
201693_s_at; 201694_s_at; 227404_s_at	EGR1		x	211323_s_at; 216944_s_at	IP3R1

IDs in active data set	Object name	miR-128 tgts_miRanda	Transcription Factor	IDs of corresponding network object from GeneGo network	Corresponding network object name
1552703_s_at; 206011_at; 209970_x_at; 211366_x_at; 211367_s_at; 211368_s_at	Caspase-1	x		1552701_a_at; 1552703_s_at	Pseudo-ICE
201693_s_at; 201694_s_at; 227404_s_at	EGR1		x	211506_s_at	IL-8
201693_s_at; 201694_s_at; 227404_s_at	EGR1		x	211711_s_at; 242622_x_at	PTEN
201693_s_at; 201694_s_at; 227404_s_at	EGR1		x	212205_at; 212206_s_at	H2AFV
201693_s_at; 201694_s_at; 227404_s_at	EGR1		x	212221_x_at; 212223_at	IDS
201693_s_at; 201694_s_at; 227404_s_at	EGR1		x	212362_at	Ca-ATPase2
201693_s_at; 201694_s_at; 227404_s_at	EGR1		x	212749_s_at	PIRH2
201693_s_at; 201694_s_at; 227404_s_at	EGR1		x	213003_s_at	C/EBPdelta
201693_s_at; 201694_s_at; 227404_s_at	EGR1		x	213348_at	p57
201693_s_at; 201694_s_at; 227404_s_at	EGR1		x	213596_at	Caspase-4
201693_s_at; 201694_s_at; 227404_s_at	EGR1		x	214443_at; 214444_s_at; 216283_s_at; 32699_s_at	PVR
201693_s_at; 201694_s_at; 227404_s_at	EGR1		x	215064_at	SC5D
201693_s_at; 201694_s_at; 227404_s_at	EGR1		x	215916_at	nAChR epsilon
201693_s_at; 201694_s_at;	EGR1		x	217192_s_at; 228964_at	BLIMP1 (PRDI-BF1)

IDs in active data set	Object name	miR-128 tgts_miRanda	Transcription Factor	IDs of corresponding network object from GeneGo network	Corresponding network object name
1552703_s_at; 206011_at; 209970_x_at; 211366_x_at; 211367_s_at; 211368_s_at	Caspase-1	x		1552701_a_at; 1552703_s_at	Pseudo-ICE
227404_s_at					
201693_s_at; 201694_s_at; 227404_s_at	EGR1		x	217542_at	MDM2
201693_s_at; 201694_s_at; 227404_s_at	EGR1		x	217728_at	Calcyclin
201693_s_at; 201694_s_at; 227404_s_at	EGR1		x	217841_s_at; 49077_at	PPME1
201693_s_at; 201694_s_at; 227404_s_at	EGR1		x	217871_s_at	MIF
201693_s_at; 201694_s_at; 227404_s_at	EGR1		x	217999_s_at; 225842_at	PHLDA1
201693_s_at; 201694_s_at; 227404_s_at	EGR1		x	218832_x_at	Beta-arrestin1
201693_s_at; 201694_s_at; 227404_s_at	EGR1		x	219235_s_at	Phactr4
201693_s_at; 201694_s_at; 227404_s_at	EGR1		x	219306_at	HKLP2
201693_s_at; 201694_s_at; 227404_s_at	EGR1		x	219403_s_at	Heparanase 1
201693_s_at; 201694_s_at; 227404_s_at	EGR1		x	220334_at	RGS17
201693_s_at; 201694_s_at; 227404_s_at	EGR1		x	221502_at	Karyopherin alpha 3
201693_s_at; 201694_s_at; 227404_s_at	EGR1		x	221664_s_at	JAM1
201693_s_at; 201694_s_at;	EGR1		x	221986_s_at; 226158_at;	KLHL24

IDs in active data set	Object name	miR-128 tgts_miRanda	Transcription Factor	IDs of corresponding network object from GeneGo network	Corresponding network object name
1552703_s_at; 206011_at; 209970_x_at; 211366_x_at; 211367_s_at; 211368_s_at	Caspase-1	x		1552701_a_at; 1552703_s_at	Pseudo-ICE
227404_s_at				242088_at	
201693_s_at; 201694_s_at; 227404_s_at	EGR1		x	222173_s_at	TBC1D2
201693_s_at; 201694_s_at; 227404_s_at	EGR1		x	224861_at	G-protein alpha-q
201693_s_at; 201694_s_at; 227404_s_at	EGR1		x	226005_at	UBE2G1
201693_s_at; 201694_s_at; 227404_s_at	EGR1		x	226075_at	SSB1
201693_s_at; 201694_s_at; 227404_s_at	EGR1		x	226336_at	Cyclophilin A
201693_s_at; 201694_s_at; 227404_s_at	EGR1		x	228065_at	BCL9-2
201693_s_at; 201694_s_at; 227404_s_at	EGR1		x	230329_s_at	GFG-1
201693_s_at; 201694_s_at; 227404_s_at	EGR1		x	231801_at	NF-AT1(NFATC2)
201693_s_at; 201694_s_at; 227404_s_at	EGR1		x	231966_at	Neurabin-1
201693_s_at; 201694_s_at; 227404_s_at	EGR1		x	233781_s_at	RIF1
201693_s_at; 201694_s_at; 227404_s_at	EGR1		x	236281_x_at	HTR7
201693_s_at; 201694_s_at; 227404_s_at	EGR1		x	242482_at	PRKAR1A
201693_s_at; 201694_s_at;	EGR1		x	242707_at	DRIP130

IDs in active data set	Object name	miR-128 tgts_miRanda	Transcription Factor	IDs of corresponding network object from GeneGo network	Corresponding network object name
1552703_s_at; 206011_at; 209970_x_at; 211366_x_at; 211367_s_at; 211368_s_at	Caspase-1	x		1552701_a_at; 1552703_s_at	Pseudo-ICE
227404_s_at					
201693_s_at; 201694_s_at; 227404_s_at	EGR1		x	244463_at	ADAM23
201862_s_at	LRRFIP1	x	x	1565483_at	EGFR
201862_s_at	LRRFIP1	x	x	202240_at	PLK1
201862_s_at	LRRFIP1	x	x	204654_s_at	AP-2A
201862_s_at	LRRFIP1	x	x	205463_s_at; 216867_s_at	PDGF-A
201862_s_at	LRRFIP1	x	x	205731_s_at; 205732_s_at	NCOA2 (GRIP1/TIF2)
201862_s_at	LRRFIP1	x	x	223679_at	Beta-catenin
202240_at	PLK1			1553502_a_at	AKAP2
202240_at	PLK1			1553690_at	Sgo1
202240_at	PLK1			1554264_at; 218252_at	CKAP2
202240_at	PLK1			1554462_a_at; 202842_s_at; 202843_at	DnaJB9
202240_at	PLK1			1554768_a_at; 203362_s_at	MAD2a
202240_at	PLK1			1556051_a_at	BICD1
202240_at	PLK1			1561079_at; 213035_at; 226025_at; 229307_at	ANKRD28
202240_at	PLK1			1568574_x_at	Osteopontin
202240_at	PLK1			201316_at	PSMA2
202240_at	PLK1			201458_s_at; 209974_s_at	BUB3
202240_at	PLK1			201896_s_at	DDA3
202240_at	PLK1			201939_at	PLK2
202240_at	PLK1			202094_at; 202095_s_at	Survivin
202240_at	PLK1			202384_s_at	TCOF1
202240_at	PLK1			202580_x_at	FOXM1
202240_at	PLK1			204026_s_at	HZwint-1
202240_at	PLK1			204318_s_at	GTSE1
202240_at	PLK1			204444_at	KNSL1

IDs in active data set	Object name	miR-128 tgts_miRanda	Transcription Factor	IDs of corresponding network object from GeneGo network	Corresponding network object name
1552703_s_at; 206011_at; 209970_x_at; 211366_x_at; 211367_s_at; 211368_s_at	Caspase-1	x		1552701_a_at; 1552703_s_at	Pseudo-ICE
202240_at	PLK1			204641_at; 211080_s_at	Nek2A
202240_at	PLK1			205167_s_at; 216914_at; 217010_s_at	CDC25C
202240_at	PLK1			207828_s_at; 209172_s_at	CENP-F
202240_at	PLK1			209251_x_at; 211750_x_at	Tubulin alpha-1C
202240_at	PLK1			209341_s_at	IKK-beta
202240_at	PLK1			209680_s_at	MKLP2
202240_at	PLK1			210440_s_at; 210743_s_at	CDC14a
202240_at	PLK1			210793_s_at	NUP98
202240_at	PLK1			211077_s_at	TLK1
202240_at	PLK1			211986_at	AHNAK
202240_at	PLK1			212480_at	KIAA0376
202240_at	PLK1			212533_at	Wee1
202240_at	PLK1			212801_at	Citron
202240_at	PLK1			213746_s_at; 214752_x_at	Filamin A
202240_at	PLK1			213750_at	RSL1D1
202240_at	PLK1			214073_at	Cortactin
202240_at	PLK1			214607_at	PAK3
202240_at	PLK1			214856_at; 215918_s_at	Beta-fodrin
202240_at	PLK1			214948_s_at	TMF1
202240_at	PLK1			215606_s_at	ELKS
202240_at	PLK1			217542_at	MDM2
202240_at	PLK1			218542_at	Cep55
202240_at	PLK1			218883_s_at	CENP-50
202240_at	PLK1			219918_s_at; 232238_at	ASPM
202240_at	PLK1			221520_s_at	CDCA8
202240_at	PLK1			222036_s_at; 222037_at	MCM4
202240_at	PLK1			222077_s_at	RacGAP1
202240_at	PLK1			222685_at	HAUS6

IDs in active data set	Object name	miR-128 tgts_miRanda	Transcription Factor	IDs of corresponding network object from GeneGo network	Corresponding network object name
1552703_s_at; 206011_at; 209970_x_at; 211366_x_at; 211367_s_at; 211368_s_at	Caspase-1	x		1552701_a_at; 1552703_s_at	Pseudo-ICE
202240_at	PLK1			226336_at	Cyclophilin A
202240_at	PLK1			241813_at	MBD1
202240_at	PLK1			242030_at	Kanadaptin
202580_x_at	FOXM1		x	1552656_s_at; 227740_at; 235003_at	KIS
202580_x_at	FOXM1		x	200783_s_at	Stathmin
202580_x_at	FOXM1		x	201853_s_at	CDC25B
202580_x_at	FOXM1		x	201897_s_at	CKS1
202580_x_at	FOXM1		x	202094_at; 202095_s_at	Survivin
202580_x_at	FOXM1		x	202678_at	TFIIA gamma chain
202580_x_at	FOXM1		x	203655_at	XRCC1
202580_x_at	FOXM1		x	204826_at	Cyclin F
202580_x_at	FOXM1		x	205015_s_at; 211258_s_at	TGF-alpha
202580_x_at	FOXM1		x	205167_s_at; 216914_at; 217010_s_at	CDC25C
202580_x_at	FOXM1		x	205235_s_at	KRMP1
202580_x_at	FOXM1		x	207828_s_at; 209172_s_at	CENP-F
202580_x_at	FOXM1		x	210477_x_at; 226048_at	JNK1(MAPK8)
202580_x_at	FOXM1		x	210512_s_at; 211527_x_at	VEGF-A
202580_x_at	FOXM1		x	213007_at	FANCI (KIAA1794)
202580_x_at	FOXM1		x	213012_at	NEDD4
203035_s_at	PIAS3		x	1558233_s_at; 1565269_s_at	ATF-1
203035_s_at	PIAS3		x	1567223_at; 1567224_at	HMGA2
203035_s_at	PIAS3		x	202599_s_at; 202600_s_at	RIP140
203035_s_at	PIAS3		x	205731_s_at; 205732_s_at	NCOA2 (GRIP1/TIF2)
203035_s_at	PIAS3		x	208213_s_at; 210078_s_at; 210079_x_at;	KCNAB1

IDs in active data set	Object name	miR-128 tgts_miRanda	Transcription Factor	IDs of corresponding network object from GeneGo network	Corresponding network object name
1552703_s_at; 206011_at; 209970_x_at; 211366_x_at; 211367_s_at; 211368_s_at	Caspase-1	x		1552701_a_at; 1552703_s_at	Pseudo-ICE
				210471_s_at	
203035_s_at	PIAS3		x	208991_at; 208992_s_at; 225289_at	STAT3
203035_s_at	PIAS3		x	209339_at	SIAH2
203035_s_at	PIAS3		x	210426_x_at; 210479_s_at; 226682_at; 235567_at; 236266_at	ROR-alpha
203035_s_at	PIAS3		x	212287_at	SUZ12
203035_s_at	PIAS3		x	218284_at	SMAD3
203035_s_at	PIAS3		x	219497_s_at; 219498_s_at; 222891_s_at	CTIP1
203035_s_at	PIAS3		x	221924_at	Zimp7
203035_s_at	PIAS3		x	224889_at; 231548_at	FOXO3A
203035_s_at	PIAS3		x	226066_at	MITF
203035_s_at	PIAS3		x	226563_at; 235598_at	SMAD2
203035_s_at	PIAS3		x	226687_at	HYPA
203035_s_at	PIAS3		x	241813_at	MBD1
203065_s_at; 212097_at	Caveolin-1	x		1552648_a_at; 231775_at	DR4(TNFRSF10A)
203065_s_at; 212097_at	Caveolin-1	x		1552671_a_at	NHE7
203065_s_at; 212097_at	Caveolin-1	x		1553994_at; 227486_at	5'-NTD
203065_s_at; 212097_at	Caveolin-1	x		200021_at	Cofilin, non-muscle
203065_s_at; 212097_at	Caveolin-1	x		200783_s_at	Stathmin
203065_s_at; 212097_at	Caveolin-1	x		200894_s_at; 200895_s_at	FKBP4
203065_s_at; 212097_at	Caveolin-1	x		201313_at	ENO2
203065_s_at; 212097_at	Caveolin-1	x		201549_x_at	PLU-1

IDs in active data set	Object name	miR-128 tgts_miRanda	Transcription Factor	IDs of corresponding network object from GeneGo network	Corresponding network object name
1552703_s_at; 206011_at; 209970_x_at; 211366_x_at; 211367_s_at; 211368_s_at	Caspase-1	x		1552701_a_at; 1552703_s_at	Pseudo-ICE
203065_s_at; 212097_at	Caveolin-1	x		201693_s_at; 201694_s_at; 227404_s_at	EGR1
203065_s_at; 212097_at	Caveolin-1	x		201840_at	NEDD8
203065_s_at; 212097_at	Caveolin-1	x		203010_at	STAT5A
203065_s_at; 212097_at	Caveolin-1	x		203504_s_at; 203505_at; 216066_at	ABCA1
203065_s_at; 212097_at	Caveolin-1	x		204186_s_at; 228469_at	Cyclophilin D
203065_s_at; 212097_at	Caveolin-1	x		205032_at; 227314_at	ITGA2
203065_s_at; 212097_at	Caveolin-1	x		205715_at	BST1
203065_s_at; 212097_at	Caveolin-1	x		208591_s_at	PDE3B
203065_s_at; 212097_at	Caveolin-1	x		209925_at	Occludin
203065_s_at; 212097_at	Caveolin-1	x		210449_x_at; 211561_x_at	p38alpha (MAPK14)
203065_s_at; 212097_at	Caveolin-1	x		210753_s_at; 211898_s_at; 230425_at	Ephrin-B receptor 1
203065_s_at; 212097_at	Caveolin-1	x		211074_at	FR-alpha
203065_s_at; 212097_at	Caveolin-1	x		211323_s_at; 216944_s_at	IP3R1
203065_s_at; 212097_at	Caveolin-1	x		211711_s_at; 242622_x_at	PTEN
203065_s_at; 212097_at	Caveolin-1	x		212218_s_at	FASN
203065_s_at; 212097_at	Caveolin-1	x		212287_at	SUZ12
203065_s_at; 212097_at	Caveolin-1	x		212549_at	STAT5B
203065_s_at; 212097_at	Caveolin-1	x		213010_at	PRKCDBP

IDs in active data set	Object name	miR-128 tgts_miRanda	Transcription Factor	IDs of corresponding network object from GeneGo network	Corresponding network object name
1552703_s_at; 206011_at; 209970_x_at; 211366_x_at; 211367_s_at; 211368_s_at	Caspase-1	x		1552701_a_at; 1552703_s_at	Pseudo-ICE
203065_s_at; 212097_at	Caveolin-1	x		213746_s_at; 214752_x_at	Filamin A
203065_s_at; 212097_at	Caveolin-1	x		215346_at; 222292_at	CD40(TNFRSF5)
203065_s_at; 212097_at	Caveolin-1	x		216985_s_at	Syntaxin 3
203065_s_at; 212097_at	Caveolin-1	x		217963_s_at	NADE(NGFRAP1)
203065_s_at; 212097_at	Caveolin-1	x		221489_s_at	SPRY4
203065_s_at; 212097_at	Caveolin-1	x		224861_at	G-protein alpha-q
203065_s_at; 212097_at	Caveolin-1	x		226336_at	Cyclophilin A
203065_s_at; 212097_at	Caveolin-1	x		226563_at; 235598_at	SMAD2
203065_s_at; 212097_at	Caveolin-1	x		235412_at	BETA-PIX
203065_s_at; 212097_at	Caveolin-1	x		238669_at	COX-1 (PTGS1)
203065_s_at; 212097_at	Caveolin-1	x		244508_at	Septin 7
203065_s_at; 212097_at	Caveolin-1	x		34697_at	LRP6
203213_at; 203214_x_at; 210559_s_at	CDK1 (p34)			1553502_a_at	AKAP2
203213_at; 203214_x_at; 210559_s_at	CDK1 (p34)			1554264_at; 218252_at	CKAP2
203213_at; 203214_x_at; 210559_s_at	CDK1 (p34)			1554481_a_at; 214530_x_at	EPB41
203213_at; 203214_x_at; 210559_s_at	CDK1 (p34)			1554634_at	APRIN
203213_at; 203214_x_at; 210559_s_at	CDK1 (p34)			1565483_at	EGFR

IDs in active data set	Object name	miR-128 tgts_miRanda	Transcription Factor	IDs of corresponding network object from GeneGo network	Corresponding network object name
1552703_s_at; 206011_at; 209970_x_at; 211366_x_at; 211367_s_at; 211368_s_at	Caspase-1	x		1552701_a_at; 1552703_s_at	Pseudo-ICE
203213_at; 203214_x_at; 210559_s_at	CDK1 (p34)			1567223_at; 1567224_at	HMGA2
203213_at; 203214_x_at; 210559_s_at	CDK1 (p34)			1570425_s_at	LATS1
203213_at; 203214_x_at; 210559_s_at	CDK1 (p34)			200711_s_at; 200719_at	SKP1
203213_at; 203214_x_at; 210559_s_at	CDK1 (p34)			200726_at	PP1-cat gamma
203213_at; 203214_x_at; 210559_s_at	CDK1 (p34)			200783_s_at	Stathmin
203213_at; 203214_x_at; 210559_s_at	CDK1 (p34)			201320_at	BAF170
203213_at; 203214_x_at; 210559_s_at	CDK1 (p34)			201373_at; 216971_s_at	Plectin 1
203213_at; 203214_x_at; 210559_s_at	CDK1 (p34)			201397_at	SERA
203213_at; 203214_x_at; 210559_s_at	CDK1 (p34)			201615_x_at	Caldesmon
203213_at; 203214_x_at; 210559_s_at	CDK1 (p34)			201853_s_at	CDC25B
203213_at; 203214_x_at; 210559_s_at	CDK1 (p34)			201897_s_at	CKS1
203213_at; 203214_x_at; 210559_s_at	CDK1 (p34)			202094_at; 202095_s_at	Survivin
203213_at; 203214_x_at; 210559_s_at	CDK1 (p34)			202240_at	PLK1

IDs in active data set	Object name	miR-128 tgts_miRanda	Transcription Factor	IDs of corresponding network object from GeneGo network	Corresponding network object name
1552703_s_at; 206011_at; 209970_x_at; 211366_x_at; 211367_s_at; 211368_s_at	Caspase-1	x		1552701_a_at; 1552703_s_at	Pseudo-ICE
203213_at; 203214_x_at; 210559_s_at	CDK1 (p34)			202384_s_at	TCOF1
203213_at; 203214_x_at; 210559_s_at	CDK1 (p34)			202580_x_at	FOXM1
203213_at; 203214_x_at; 210559_s_at	CDK1 (p34)			202704_at	Tob1
203213_at; 203214_x_at; 210559_s_at	CDK1 (p34)			203220_s_at; 203222_s_at	TLE1
203213_at; 203214_x_at; 210559_s_at	CDK1 (p34)			203276_at	Lamin B1
203213_at; 203214_x_at; 210559_s_at	CDK1 (p34)			203298_s_at	JARID2
203213_at; 203214_x_at; 210559_s_at	CDK1 (p34)			203554_x_at	Securin
203213_at; 203214_x_at; 210559_s_at	CDK1 (p34)			203564_at	FANCG
203213_at; 203214_x_at; 210559_s_at	CDK1 (p34)			204109_s_at; 228433_at	NFYA
203213_at; 203214_x_at; 210559_s_at	CDK1 (p34)			204170_s_at	CKS2
203213_at; 203214_x_at; 210559_s_at	CDK1 (p34)			204444_at	KNSL1
203213_at; 203214_x_at; 210559_s_at	CDK1 (p34)			204502_at	SAMHD1
203213_at; 203214_x_at; 210559_s_at	CDK1 (p34)			204767_s_at; 204768_s_at	FEN1

**IDs in active data set**	**Object name**	**miR-128 tgts_miRanda**	**Transcription Factor**	**IDs of corresponding network object from GeneGo network**	**Corresponding network object name**
1552703_s_at; 206011_at; 209970_x_at; 211366_x_at; 211367_s_at; 211368_s_at	Caspase-1	x		1552701_a_at; 1552703_s_at	Pseudo-ICE
203213_at; 203214_x_at; 210559_s_at	CDK1 (p34)			204826_at	Cyclin F
203213_at; 203214_x_at; 210559_s_at	CDK1 (p34)			204920_at; 217564_s_at	CPSM
203213_at; 203214_x_at; 210559_s_at	CDK1 (p34)			205167_s_at; 216914_at; 217010_s_at	CDC25C
203213_at; 203214_x_at; 210559_s_at	CDK1 (p34)			205899_at	Cyclin A1
203213_at; 203214_x_at; 210559_s_at	CDK1 (p34)			206665_s_at; 212312_at	Bcl-XL
203213_at; 203214_x_at; 210559_s_at	CDK1 (p34)			208093_s_at	NUDEL
203213_at; 203214_x_at; 210559_s_at	CDK1 (p34)			208553_at	HIST1H1E
203213_at; 203214_x_at; 210559_s_at	CDK1 (p34)			208991_at; 208992_s_at; 225289_at	STAT3
203213_at; 203214_x_at; 210559_s_at	CDK1 (p34)			209615_s_at	PAK1
203213_at; 203214_x_at; 210559_s_at	CDK1 (p34)			209753_s_at	TMPOA
203213_at; 203214_x_at; 210559_s_at	CDK1 (p34)			211074_at	FR-alpha
203213_at; 203214_x_at; 210559_s_at	CDK1 (p34)			211928_at	Dynein 1, cytoplasmic, heavy chain
203213_at; 203214_x_at; 210559_s_at	CDK1 (p34)			211986_at	AHNAK

IDs in active data set	Object name	miR-128 tgts_miRanda	Transcription Factor	IDs of corresponding network object from GeneGo network	Corresponding network object name
1552703_s_at; 206011_at; 209970_x_at; 211366_x_at; 211367_s_at; 211368_s_at	Caspase-1	x		1552701_a_at; 1552703_s_at	Pseudo-ICE
203213_at; 203214_x_at; 210559_s_at	CDK1 (p34)			212020_s_at; 212021_s_at; 212023_s_at	Antigen KI-67
203213_at; 203214_x_at; 210559_s_at	CDK1 (p34)			212073_at	Casein kinase II, alpha chain (CSNK2A1)
203213_at; 203214_x_at; 210559_s_at	CDK1 (p34)			212426_s_at	14-3-3 theta
203213_at; 203214_x_at; 210559_s_at	CDK1 (p34)			212533_at	Wee1
203213_at; 203214_x_at; 210559_s_at	CDK1 (p34)			212836_at	POLD reg (p68)
203213_at; 203214_x_at; 210559_s_at	CDK1 (p34)			212934_at	UBXN2B
203213_at; 203214_x_at; 210559_s_at	CDK1 (p34)			212949_at	BRRN1
203213_at; 203214_x_at; 210559_s_at	CDK1 (p34)			213226_at	Cyclin A2
203213_at; 203214_x_at; 210559_s_at	CDK1 (p34)			213324_at	c-Src
203213_at; 203214_x_at; 210559_s_at	CDK1 (p34)			213348_at	p57
203213_at; 203214_x_at; 210559_s_at	CDK1 (p34)			213359_at; 221480_at	AUF1
203213_at; 203214_x_at; 210559_s_at	CDK1 (p34)			213746_s_at; 214752_x_at	Filamin A
203213_at; 203214_x_at; 210559_s_at	CDK1 (p34)			214073_at	Cortactin

IDs in active data set	Object name	miR-128 tgts_miRanda	Transcription Factor	IDs of corresponding network object from GeneGo network	Corresponding network object name
1552703_s_at; 206011_at; 209970_x_at; 211366_x_at; 211367_s_at; 211368_s_at	Caspase-1	x		1552701_a_at; 1552703_s_at	Pseudo-ICE
203213_at; 203214_x_at; 210559_s_at	CDK1 (p34)			214195_at	CLN2 (Tripeptidyl-peptidase I)
203213_at; 203214_x_at; 210559_s_at	CDK1 (p34)			215905_s_at	HPRP8BP
203213_at; 203214_x_at; 210559_s_at	CDK1 (p34)			216100_s_at; 223930_at	LAP1
203213_at; 203214_x_at; 210559_s_at	CDK1 (p34)			218073_s_at	TMEM48
203213_at; 203214_x_at; 210559_s_at	CDK1 (p34)			218542_at	Cep55
203213_at; 203214_x_at; 210559_s_at	CDK1 (p34)			218662_s_at	CAP-G
203213_at; 203214_x_at; 210559_s_at	CDK1 (p34)			219105_x_at	ORC6L
203213_at; 203214_x_at; 210559_s_at	CDK1 (p34)			220355_s_at; 223238_s_at; 223899_at; 223900_s_at; 224152_s_at	BAF180
203213_at; 203214_x_at; 210559_s_at	CDK1 (p34)			221676_s_at	CORO1C
203213_at; 203214_x_at; 210559_s_at	CDK1 (p34)			222077_s_at	RacGAP1
203213_at; 203214_x_at; 210559_s_at	CDK1 (p34)			222616_s_at	USP16
203213_at; 203214_x_at; 210559_s_at	CDK1 (p34)			222903_s_at	CPEB1
203213_at;	CDK1			224731_at	HMG1 (amphoterin)

IDs in active data set	Object name	miR-128 tgts_miRanda	Transcription Factor	IDs of corresponding network object from GeneGo network	Corresponding network object name
1552703_s_at; 206011_at; 209970_x_at; 211366_x_at; 211367_s_at; 211368_s_at	Caspase-1	x		1552701_a_at; 1552703_s_at	Pseudo-ICE
203214_x_at; 210559_s_at	(p34)				
203213_at; 203214_x_at; 210559_s_at	CDK1 (p34)			225521_at	ANAPC7
203213_at; 203214_x_at; 210559_s_at	CDK1 (p34)			225565_at; 225572_at	CREB1
203213_at; 203214_x_at; 210559_s_at	CDK1 (p34)			226020_s_at	DAB1
203213_at; 203214_x_at; 210559_s_at	CDK1 (p34)			226336_at	Cyclophilin A
203213_at; 203214_x_at; 210559_s_at	CDK1 (p34)			227249_at	NudE
203213_at; 203214_x_at; 210559_s_at	CDK1 (p34)			228131_at	ERCC-1
203213_at; 203214_x_at; 210559_s_at	CDK1 (p34)			230229_at	SAP97 (DLG1)
203213_at; 203214_x_at; 210559_s_at	CDK1 (p34)			230662_at	RNF187
203213_at; 203214_x_at; 210559_s_at	CDK1 (p34)			236814_at	MDM4
203213_at; 203214_x_at; 210559_s_at	CDK1 (p34)			238034_at	Calnexin
203213_at; 203214_x_at; 210559_s_at	CDK1 (p34)			242918_at	NASP
204011_at	SPRY2	x		1554168_a_at; 223082_at; 235692_at	CIN85
204011_at	SPRY2	x		1565483_at	EGFR

IDs in active data set	Object name	miR-128 tgts_miRanda	Transcription Factor	IDs of corresponding network object from GeneGo network	Corresponding network object name
1552703_s_at; 206011_at; 209970_x_at; 211366_x_at; 211367_s_at; 211368_s_at	Caspase-1	x		1552701_a_at; 1552703_s_at	Pseudo-ICE
204011_at	SPRY2	x		203065_s_at; 212097_at	Caveolin-1
204011_at	SPRY2	x		204654_s_at	AP-2A
204011_at	SPRY2	x		205286_at	AP-2C
204011_at	SPRY2	x		205880_at	PKC-mu
204011_at	SPRY2	x		206870_at; 223438_s_at; 244689_at	PPAR-alpha
204011_at	SPRY2	x		209339_at	SIAH2
204011_at	SPRY2	x		209682_at	CBL-B
204011_at	SPRY2	x		213012_at	NEDD4
204011_at	SPRY2	x		213324_at	c-Src
204011_at	SPRY2	x		214443_at; 214444_s_at; 216283_s_at; 32699_s_at	PVR
204011_at	SPRY2	x		221489_s_at	SPRY4
204011_at	SPRY2	x		225565_at; 225572_at	CREB1
204011_at	SPRY2	x		238486_at	FRS2
204092_s_at; 208079_s_at; 208080_at	Aurora-A			1553764_a_at; 225806_at	Ajuba
204092_s_at; 208079_s_at; 208080_at	Aurora-A			1565483_at	EGFR
204092_s_at; 208079_s_at; 208080_at	Aurora-A			200676_s_at; 200684_s_at	UBCH7
204092_s_at; 208079_s_at; 208080_at	Aurora-A			201502_s_at	NFKBIA
204092_s_at; 208079_s_at; 208080_at	Aurora-A			201523_x_at; 201524_x_at	E2N(UBC13)
204092_s_at; 208079_s_at; 208080_at	Aurora-A			201853_s_at	CDC25B
204092_s_at;	Aurora-A			202149_at;	CAS-L

IDs in active data set	Object name	miR-128 tgts_miRanda	Transcription Factor	IDs of corresponding network object from GeneGo network	Corresponding network object name
1552703_s_at; 206011_at; 209970_x_at; 211366_x_at; 211367_s_at; 211368_s_at	Caspase-1	x		1552701_a_at; 1552703_s_at	Pseudo-ICE
208079_s_at; 208080_at				202150_s_at	
204092_s_at; 208079_s_at; 208080_at	Aurora-A			202240_at	PLK1
204092_s_at; 208079_s_at; 208080_at	Aurora-A			202517_at	CRMP1
204092_s_at; 208079_s_at; 208080_at	Aurora-A			202580_x_at	FOXM1
204092_s_at; 208079_s_at; 208080_at	Aurora-A			202677_at	p120GAP
204092_s_at; 208079_s_at; 208080_at	Aurora-A			203010_at	STAT5A
204092_s_at; 208079_s_at; 208080_at	Aurora-A			203554_x_at	Securin
204092_s_at; 208079_s_at; 208080_at	Aurora-A			203952_at	ATF-6 alpha
204092_s_at; 208079_s_at; 208080_at	Aurora-A			204162_at	HEC
204092_s_at; 208079_s_at; 208080_at	Aurora-A			204901_at; 216091_s_at	beta-TrCP
204092_s_at; 208079_s_at; 208080_at	Aurora-A			204962_s_at; 210821_x_at	CENP-A
204092_s_at; 208079_s_at; 208080_at	Aurora-A			205046_at	CENP-E
204092_s_at; 208079_s_at; 208080_at	Aurora-A			206846_s_at	HDAC6
204092_s_at;	Aurora-A			208093_s_at	NUDEL

IDs in active data set	Object name	miR-128 tgts_miRanda	Transcription Factor	IDs of corresponding network object from GeneGo network	Corresponding network object name
1552703_s_at; 206011_at; 209970_x_at; 211366_x_at; 211367_s_at; 211368_s_at	Caspase-1	x		1552701_a_at; 1552703_s_at	Pseudo-ICE
208079_s_at; 208080_at					
204092_s_at; 208079_s_at; 208080_at	Aurora-A			209341_s_at	IKK-beta
204092_s_at; 208079_s_at; 208080_at	Aurora-A			209615_s_at	PAK1
204092_s_at; 208079_s_at; 208080_at	Aurora-A			209715_at; 212126_at	HP1 alpha
204092_s_at; 208079_s_at; 208080_at	Aurora-A			218580_x_at; 225552_x_at; 225555_x_at	AKIP
204092_s_at; 208079_s_at; 208080_at	Aurora-A			220145_at; 235550_at	ASAP
204092_s_at; 208079_s_at; 208080_at	Aurora-A			222903_s_at	CPEB1
204092_s_at; 208079_s_at; 208080_at	Aurora-A			223981_at	Ninein
204092_s_at; 208079_s_at; 208080_at	Aurora-A			224828_at; 224829_at	CPEB4
204162_at	HEC			1554768_a_at; 203362_s_at	MAD2a
204162_at	HEC			1555677_s_at; 201589_at	SMC1
204162_at	HEC			1569061_at; 1569062_s_at; 229538_s_at; 241939_at	IQGAP3
204162_at	HEC			203002_at	AMOTL2
204162_at	HEC			204026_s_at	HZwint-1
204162_at	HEC			204641_at; 211080_s_at	Nek2A
204162_at	HEC			209002_s_at	CALCOCO1

IDs in active data set	Object name	miR-128 tgts_miRanda	Transcription Factor	IDs of corresponding network object from GeneGo network	Corresponding network object name
1552703_s_at; 206011_at; 209970_x_at; 211366_x_at; 211367_s_at; 211368_s_at	Caspase-1	x		1552701_a_at; 1552703_s_at	Pseudo-ICE
204162_at	HEC			209891_at	SPBC25
204162_at	HEC			218883_s_at	CENP-50
204162_at	HEC			219409_at	SNIP1
204162_at	HEC			219918_s_at; 232238_at	ASPM
204162_at	HEC			242163_at	TRAP150
204162_at	HEC			48117_at	STAF36 (CCDC101)
204254_s_at; 204255_s_at; 213692_s_at	VDR		x	1554251_at; 224591_at; 224592_x_at	HP1-BP74 protein
204254_s_at; 204255_s_at; 213692_s_at	VDR		x	1556499_s_at; 202310_s_at	COL1A1
204254_s_at; 204255_s_at; 213692_s_at	VDR		x	1558199_at	Fibronectin
204254_s_at; 204255_s_at; 213692_s_at	VDR		x	1568574_x_at	Osteopontin
204254_s_at; 204255_s_at; 213692_s_at	VDR		x	200952_s_at; 200953_s_at	Cyclin D2
204254_s_at; 204255_s_at; 213692_s_at	VDR		x	201502_s_at	NFKBIA
204254_s_at; 204255_s_at; 213692_s_at	VDR		x	202599_s_at; 202600_s_at	RIP140
204254_s_at; 204255_s_at; 213692_s_at	VDR		x	202637_s_at; 202638_s_at; 215485_s_at	ICAM1
204254_s_at; 204255_s_at; 213692_s_at	VDR		x	203108_at	RAIG1
204254_s_at; 204255_s_at; 213692_s_at	VDR		x	203183_s_at	BAF60A
204254_s_at;	VDR		x	204270_at	Ski

IDs in active data set	Object name	miR-128 tgts_miRanda	Transcription Factor	IDs of corresponding network object from GeneGo network	Corresponding network object name
1552703_s_at; 206011_at; 209970_x_at; 211366_x_at; 211367_s_at; 211368_s_at	Caspase-1	x		1552701_a_at; 1552703_s_at	Pseudo-ICE
204255_s_at; 213692_s_at					
204254_s_at; 204255_s_at; 213692_s_at	VDR		x	204626_s_at; 204627_s_at; 204628_s_at; 216261_at	ITGB3
204254_s_at; 204255_s_at; 213692_s_at	VDR		x	204654_s_at	AP-2A
204254_s_at; 204255_s_at; 213692_s_at	VDR		x	204933_s_at	Osteoprotegerin
204254_s_at; 204255_s_at; 213692_s_at	VDR		x	205463_s_at; 216867_s_at	PDGF-A
204254_s_at; 204255_s_at; 213692_s_at	VDR		x	205579_at; 205580_s_at	Histamine H1 receptor
204254_s_at; 204255_s_at; 213692_s_at	VDR		x	205603_s_at; 205726_at; 217246_s_at	HDIA2
204254_s_at; 204255_s_at; 213692_s_at	VDR		x	205731_s_at; 205732_s_at	NCOA2 (GRIP1/TIF2)
204254_s_at; 204255_s_at; 213692_s_at	VDR		x	206846_s_at	HDAC6
204254_s_at; 204255_s_at; 213692_s_at	VDR		x	206870_at; 223438_s_at; 244689_at	PPAR-alpha
204254_s_at; 204255_s_at; 213692_s_at	VDR		x	206877_at; 228846_at	MAD
204254_s_at; 204255_s_at; 213692_s_at	VDR		x	206925_at	SIAT8D
204254_s_at; 204255_s_at; 213692_s_at	VDR		x	207181_s_at	Caspase-7

IDs in active data set	Object name	miR-128 tgts_miRanda	Transcription Factor	IDs of corresponding network object from GeneGo network	Corresponding network object name
1552703_s_at; 206011_at; 209970_x_at; 211366_x_at; 211367_s_at; 211368_s_at	Caspase-1	x		1552701_a_at; 1552703_s_at	Pseudo-ICE
204254_s_at; 204255_s_at; 213692_s_at	VDR		x	207375_s_at	IL-15RA
204254_s_at; 204255_s_at; 213692_s_at	VDR		x	207992_s_at	AMD3
204254_s_at; 204255_s_at; 213692_s_at	VDR		x	208010_s_at	70Z-PEP
204254_s_at; 204255_s_at; 213692_s_at	VDR		x	209277_at; 209278_s_at	TFPI-2
204254_s_at; 204255_s_at; 213692_s_at	VDR		x	209318_x_at	Zac1
204254_s_at; 204255_s_at; 213692_s_at	VDR		x	209363_s_at	SRB7
204254_s_at; 204255_s_at; 213692_s_at	VDR		x	209377_s_at	HMGN3
204254_s_at; 204255_s_at; 213692_s_at	VDR		x	210512_s_at; 211527_x_at	VEGF-A
204254_s_at; 204255_s_at; 213692_s_at	VDR		x	212020_s_at; 212021_s_at; 212023_s_at	Antigen KI-67
204254_s_at; 204255_s_at; 213692_s_at	VDR		x	212897_at	CDK11
204254_s_at; 204255_s_at; 213692_s_at	VDR		x	213003_s_at	C/EBPdelta
204254_s_at; 204255_s_at; 213692_s_at	VDR		x	214744_s_at	RPL23
204254_s_at; 204255_s_at; 213692_s_at	VDR		x	215167_at	TRAP170

IDs in active data set	Object name	miR-128 tgts_miRanda	Transcription Factor	IDs of corresponding network object from GeneGo network	Corresponding network object name
1552703_s_at; 206011_at; 209970_x_at; 211366_x_at; 211367_s_at; 211368_s_at	Caspase-1	x		1552701_a_at; 1552703_s_at	Pseudo-ICE
204254_s_at; 204255_s_at; 213692_s_at	VDR		x	216705_s_at	ADA
204254_s_at; 204255_s_at; 213692_s_at	VDR		x	217557_s_at	Carboxypeptidase M
204254_s_at; 204255_s_at; 213692_s_at	VDR		x	218284_at	SMAD3
204254_s_at; 204255_s_at; 213692_s_at	VDR		x	218832_x_at	Beta-arrestin1
204254_s_at; 204255_s_at; 213692_s_at	VDR		x	219249_s_at	FKBP10
204254_s_at; 204255_s_at; 213692_s_at	VDR		x	219318_x_at; 236241_at	TRAP18
204254_s_at; 204255_s_at; 213692_s_at	VDR		x	220655_at	ABIN-3
204254_s_at; 204255_s_at; 213692_s_at	VDR		x	221218_s_at; 223686_at	TPK1
204254_s_at; 204255_s_at; 213692_s_at	VDR		x	224221_s_at	VAV-3
204254_s_at; 204255_s_at; 213692_s_at	VDR		x	228131_at	ERCC-1
204254_s_at; 204255_s_at; 213692_s_at	VDR		x	230229_at	SAP97 (DLG1)
204254_s_at; 204255_s_at; 213692_s_at	VDR		x	242163_at	TRAP150
204254_s_at; 204255_s_at; 213692_s_at	VDR		x	242707_at	DRIP130

IDs in active data set	Object name	miR-128 tgts_miRanda	Transcription Factor	IDs of corresponding network object from GeneGo network	Corresponding network object name
1552703_s_at; 206011_at; 209970_x_at; 211366_x_at; 211367_s_at; 211368_s_at	Caspase-1	x		1552701_a_at; 1552703_s_at	Pseudo-ICE
204254_s_at; 204255_s_at; 213692_s_at	VDR		x	242918_at	NASP
204613_at	PLC-gamma 2			1554168_a_at; 223082_at; 235692_at	CIN85
204613_at	PLC-gamma 2			1558199_at	Fibronectin
204613_at	PLC-gamma 2			1568856_at; 1568857_a_at; 201383_s_at; 201384_s_at	NBR1
204613_at	PLC-gamma 2			200919_at	EDR2
204613_at	PLC-gamma 2			201999_s_at	Tctex-1
204613_at	PLC-gamma 2			203688_at	Polycystin 2
204613_at	PLC-gamma 2			204011_at	SPRY2
204613_at	PLC-gamma 2			204162_at	HEC
204613_at	PLC-gamma 2			205812_s_at	TMED9
204613_at	PLC-gamma 2			205880_at	PKC-mu
204613_at	PLC-gamma 2			207112_s_at; 225998_at	GAB1
204613_at	PLC-gamma 2			209682_at	CBL-B
204613_at	PLC-gamma 2			211005_at	LAT
204613_at	PLC-gamma 2			212118_at	RFP
204613_at	PLC-gamma 2			213324_at	c-Src
204613_at	PLC-gamma 2			213746_s_at; 214752_x_at	Filamin A
204613_at	PLC-			214856_at;	Beta-fodrin

IDs in active data set	Object name	miR-128 tgts_miRanda	Transcription Factor	IDs of corresponding network object from GeneGo network	Corresponding network object name
1552703_s_at; 206011_at; 209970_x_at; 211366_x_at; 211367_s_at; 211368_s_at	Caspase-1	x		1552701_a_at; 1552703_s_at	Pseudo-ICE
	gamma 2			215918_s_at	
204613_at	PLC-gamma 2			221581_s_at	WBSCR5(NTAL)
204613_at	PLC-gamma 2			224366_s_at	REPS1
204613_at	PLC-gamma 2			232097_at	TOX4
204901_at; 216091_s_at	beta-TrCP			1554086_at	TUBGCP3
204901_at; 216091_s_at	beta-TrCP			1558199_at	Fibronectin
204901_at; 216091_s_at	beta-TrCP			200711_s_at; 200719_at	SKP1
204901_at; 216091_s_at	beta-TrCP			201502_s_at	NFKBIA
204901_at; 216091_s_at	beta-TrCP			201840_at	NEDD8
204901_at; 216091_s_at	beta-TrCP			201939_at	PLK2
204901_at; 216091_s_at	beta-TrCP			202196_s_at	DKK3
204901_at; 216091_s_at	beta-TrCP			203554_x_at	Securin
204901_at; 216091_s_at	beta-TrCP			204191_at	IFNAR1
204901_at; 216091_s_at	beta-TrCP			204535_s_at	NRSF
204901_at; 216091_s_at	beta-TrCP			204809_at; 223507_at	ClpX
204901_at; 216091_s_at	beta-TrCP			208370_s_at	Calcipressin 1
204901_at; 216091_s_at	beta-TrCP			208518_s_at	PER2
204901_at; 216091_s_at	beta-TrCP			209341_s_at	IKK-beta
204901_at; 216091_s_at	beta-TrCP			211764_s_at; 214590_s_at	UBE2D1
204901_at; 216091_s_at	beta-TrCP			212533_at	Wee1

IDs in active data set	Object name	miR-128 tgts_miRanda	Transcription Factor	IDs of corresponding network object from GeneGo network	Corresponding network object name
1552703_s_at; 206011_at; 209970_x_at; 211366_x_at; 211367_s_at; 211368_s_at	Caspase-1	x		1552701_a_at; 1552703_s_at	Pseudo-ICE
204901_at; 216091_s_at	beta-TrCP			212594_at	PDCD4
204901_at; 216091_s_at	beta-TrCP			218284_at	SMAD3
204901_at; 216091_s_at	beta-TrCP			222749_at	SUFU
204901_at; 216091_s_at	beta-TrCP			223679_at	Beta-catenin
204901_at; 216091_s_at	beta-TrCP			224889_at; 231548_at	FOXO3A
204901_at; 216091_s_at	beta-TrCP			225131_at; 225138_at	TRABID
204901_at; 216091_s_at	beta-TrCP			229422_at	Nardilysin
204901_at; 216091_s_at	beta-TrCP			230229_at	SAP97 (DLG1)
204901_at; 216091_s_at	beta-TrCP			230280_at	TRIM9
204962_s_at; 210821_x_at	CENP-A			200919_at	EDR2
204962_s_at; 210821_x_at	CENP-A			202580_x_at	FOXM1
204962_s_at; 210821_x_at	CENP-A			203975_s_at; 229808_at	ChAF1 subunit A
204962_s_at; 210821_x_at	CENP-A			204739_at	CENP-C
204962_s_at; 210821_x_at	CENP-A			204752_x_at; 215773_x_at	PARP-2
204962_s_at; 210821_x_at	CENP-A			205603_s_at; 205726_at; 217246_s_at	HDIA2
204962_s_at; 210821_x_at	CENP-A			207590_s_at; 214804_at	CENP-I (FSHPRH1)
204962_s_at; 210821_x_at	CENP-A			208371_s_at	RING1
204962_s_at; 210821_x_at	CENP-A			208597_at	ZFP91
204962_s_at; 210821_x_at	CENP-A			209715_at; 212126_at	HP1 alpha

IDs in active data set	Object name	miR-128 tgts_miRanda	Transcription Factor	IDs of corresponding network object from GeneGo network	Corresponding network object name
1552703_s_at; 206011_at; 209970_x_at; 211366_x_at; 211367_s_at; 211368_s_at	Caspase-1	x		1552701_a_at; 1552703_s_at	Pseudo-ICE
204962_s_at; 210821_x_at	CENP-A			214500_at; 214501_s_at	Macro-H2A.1 histone
204962_s_at; 210821_x_at	CENP-A			218726_at	HJURP
204962_s_at; 210821_x_at	CENP-A			218883_s_at	CENP-50
204962_s_at; 210821_x_at	CENP-A			222077_s_at	RacGAP1
204962_s_at; 210821_x_at	CENP-A			225396_at; 237333_at; 244872_at	RBBP4 (RbAp48)
204962_s_at; 210821_x_at	CENP-A			229630_s_at	WTAP
205046_at	CENP-E			200711_s_at; 200719_at	SKP1
205046_at	CENP-E			200726_at	PP1-cat gamma
205046_at	CENP-E			202240_at	PLK1
205046_at	CENP-E			204162_at	HEC
205046_at	CENP-E			204962_s_at; 210821_x_at	CENP-A
205046_at	CENP-E			207828_s_at; 209172_s_at	CENP-F
205046_at	CENP-E			220355_s_at; 223238_s_at; 223899_at; 223900_s_at; 224152_s_at	BAF180
205046_at	CENP-E			221258_s_at	KIF18A
205046_at	CENP-E			244508_at	Septin 7
205176_s_at	NRIF3		x	201124_at	ITGB5
205176_s_at	NRIF3		x	203368_at	CRELD1
205176_s_at	NRIF3		x	204109_s_at; 228433_at	NFYA
205176_s_at	NRIF3		x	204626_s_at; 204627_s_at; 204628_s_at; 216261_at	ITGB3
205176_s_at	NRIF3		x	204760_s_at; 31637_s_at	TR-alpha

IDs in active data set	Object name	miR-128 tgts_miRanda	Transcription Factor	IDs of corresponding network object from GeneGo network	Corresponding network object name
1552703_s_at; 206011_at; 209970_x_at; 211366_x_at; 211367_s_at; 211368_s_at	Caspase-1	x		1552701_a_at; 1552703_s_at	Pseudo-ICE
205176_s_at	NRIF3		x	204962_s_at; 210821_x_at	CENP-A
205176_s_at	NRIF3		x	205425_at	HIP1
205176_s_at	NRIF3		x	206665_s_at; 212312_at	Bcl-XL
205176_s_at	NRIF3		x	211995_x_at; 212988_x_at	Actin cytoplasmic 2
205176_s_at	NRIF3		x	213226_at	Cyclin A2
205176_s_at	NRIF3		x	223042_s_at	FUNDC2
205176_s_at	NRIF3		x	233781_s_at	RIF1
205176_s_at	NRIF3		x	238555_at	MRPS31
206536_s_at; 225859_at	XIAP			201523_x_at; 201524_x_at	E2N(UBC13)
206536_s_at; 225859_at	XIAP			202094_at; 202095_s_at	Survivin
206536_s_at; 225859_at	XIAP			202672_s_at	ATF-3
206536_s_at; 225859_at	XIAP			202901_x_at; 202902_s_at	Cathepsin S
206536_s_at; 225859_at	XIAP			203010_at	STAT5A
206536_s_at; 225859_at	XIAP			203065_s_at; 212097_at	Caveolin-1
206536_s_at; 225859_at	XIAP			203564_at	FANCG
206536_s_at; 225859_at	XIAP			205209_at; 208218_s_at; 208223_s_at; 213198_at	ALK-4
206536_s_at; 225859_at	XIAP			207181_s_at	Caspase-7
206536_s_at; 225859_at	XIAP			211711_s_at; 242622_x_at	PTEN
206536_s_at; 225859_at	XIAP			211764_s_at; 214590_s_at	UBE2D1
206536_s_at; 225859_at	XIAP			213596_at	Caspase-4
206536_s_at; 225859_at	XIAP			215606_s_at	ELKS

IDs in active data set	Object name	miR-128 tgts_miRanda	Transcription Factor	IDs of corresponding network object from GeneGo network	Corresponding network object name
1552703_s_at; 206011_at; 209970_x_at; 211366_x_at; 211367_s_at; 211368_s_at	Caspase-1	x		1552701_a_at; 1552703_s_at	Pseudo-ICE
206536_s_at; 225859_at	XIAP			217542_at	MDM2
206536_s_at; 225859_at	XIAP			218837_s_at	UBE2D4
206536_s_at; 225859_at	XIAP			221695_s_at; 226979_at	MAP3K2 (MEKK2)
206536_s_at; 225859_at	XIAP			225276_at	GSPT1
206536_s_at; 225859_at	XIAP			225651_at	UBE2E2
206536_s_at; 225859_at	XIAP			227625_s_at	CHIP
207714_s_at	HSP47	x		1554168_a_at; 223082_at; 235692_at	CIN85
207714_s_at	HSP47	x		1555832_s_at	KLF6
207714_s_at	HSP47	x		1556499_s_at; 202310_s_at	COL1A1
207714_s_at	HSP47	x		201087_at; 211823_s_at	Paxillin
207714_s_at	HSP47	x		202241_at; 235641_at	C8FW (GIG2)
207714_s_at	HSP47	x		208423_s_at; 211887_x_at	08p22/MSR1(CD204)
207714_s_at	HSP47	x		208991_at; 208992_s_at; 225289_at	STAT3
207714_s_at	HSP47	x		211736_at	SP2
207714_s_at	HSP47	x		214534_at	Histone H1b
207714_s_at	HSP47	x		214881_s_at	UBF
207714_s_at	HSP47	x		221019_s_at	COLEC12
208939_at	SEPHS1			1553167_a_at; 231730_at; 235516_at	SLA/LP
208939_at	SEPHS1			202269_x_at; 231577_s_at	GBP1
208939_at	SEPHS1			202517_at	CRMP1
208939_at	SEPHS1			202748_at	GBP2
208939_at	SEPHS1			203455_s_at;	SSAT

IDs in active data set	Object name	miR-128 tgts_miRanda	Transcription Factor	IDs of corresponding network object from GeneGo network	Corresponding network object name
1552703_s_at; 206011_at; 209970_x_at; 211366_x_at; 211367_s_at; 211368_s_at	Caspase-1	x		1552701_a_at; 1552703_s_at	Pseudo-ICE
				210592_s_at; 213988_s_at	
208939_at	SEPHS1			210654_at; 227345_at	TRUNDD(TNFRSF 10D)
208939_at	SEPHS1			217871_s_at	MIF
208991_at; 208992_s_at; 225289_at	STAT3		x	1553032_at; 1555431_a_at; 243541_at	IL-31RA
208991_at; 208992_s_at; 225289_at	STAT3		x	1558233_s_at; 1565269_s_at	ATF-1
208991_at; 208992_s_at; 225289_at	STAT3		x	1558662_s_at; 222915_s_at	BANK
208991_at; 208992_s_at; 225289_at	STAT3		x	1561054_a_at	CCDC14
208991_at; 208992_s_at; 225289_at	STAT3		x	1567223_at; 1567224_at	HMGA2
208991_at; 208992_s_at; 225289_at	STAT3		x	1568574_x_at	Osteopontin
208991_at; 208992_s_at; 225289_at	STAT3		x	1568765_at; 202627_s_at; 202628_s_at	PAI1
208991_at; 208992_s_at; 225289_at	STAT3		x	1598_g_at	Gas6
208991_at; 208992_s_at; 225289_at	STAT3		x	200658_s_at	Prohibitin
208991_at; 208992_s_at; 225289_at	STAT3		x	200783_s_at	Stathmin
208991_at; 208992_s_at; 225289_at	STAT3		x	201487_at; 225647_s_at	Cathepsin C
208991_at;	STAT3		x	201666_at	TIMP1

IDs in active data set	Object name	miR-128 tgts_miRanda	Transcription Factor	IDs of corresponding network object from GeneGo network	Corresponding network object name
1552703_s_at; 206011_at; 209970_x_at; 211366_x_at; 211367_s_at; 211368_s_at	Caspase-1	x		1552701_a_at; 1552703_s_at	Pseudo-ICE
208992_s_at; 225289_at					
208991_at; 208992_s_at; 225289_at	STAT3		x	201693_s_at; 201694_s_at; 227404_s_at	EGR1
208991_at; 208992_s_at; 225289_at	STAT3		x	201841_s_at	HSP27
208991_at; 208992_s_at; 225289_at	STAT3		x	202057_at	Karyopherin alpha 1
208991_at; 208992_s_at; 225289_at	STAT3		x	202060_at	CTR9
208991_at; 208992_s_at; 225289_at	STAT3		x	202090_s_at	UQCR11
208991_at; 208992_s_at; 225289_at	STAT3		x	202092_s_at	BART1
208991_at; 208992_s_at; 225289_at	STAT3		x	202094_at; 202095_s_at	Survivin
208991_at; 208992_s_at; 225289_at	STAT3		x	202580_x_at	FOXM1
208991_at; 208992_s_at; 225289_at	STAT3		x	202637_s_at; 202638_s_at; 215485_s_at	ICAM1
208991_at; 208992_s_at; 225289_at	STAT3		x	202748_at	GBP2
208991_at; 208992_s_at; 225289_at	STAT3		x	202833_s_at; 211429_s_at	Alpha 1-antitrypsin
208991_at; 208992_s_at; 225289_at	STAT3		x	203065_s_at; 212097_at	Caveolin-1
208991_at;	STAT3		x	203373_at	SOCS2

IDs in active data set	Object name	miR-128 tgts_miRanda	Transcription Factor	IDs of corresponding network object from GeneGo network	Corresponding network object name
1552703_s_at; 206011_at; 209970_x_at; 211366_x_at; 211367_s_at; 211368_s_at	Caspase-1	x		1552701_a_at; 1552703_s_at	Pseudo-ICE
208992_s_at; 225289_at					
208991_at; 208992_s_at; 225289_at	STAT3		x	203964_at	NMI
208991_at; 208992_s_at; 225289_at	STAT3		x	204191_at	IFNAR1
208991_at; 208992_s_at; 225289_at	STAT3		x	204435_at; 223984_s_at	NUP58
208991_at; 208992_s_at; 225289_at	STAT3		x	204455_at	BPAG1
208991_at; 208992_s_at; 225289_at	STAT3		x	204535_s_at	NRSF
208991_at; 208992_s_at; 225289_at	STAT3		x	204557_s_at	DZIP
208991_at; 208992_s_at; 225289_at	STAT3		x	204662_at	CP110
208991_at; 208992_s_at; 225289_at	STAT3		x	205798_at	IL7RA
208991_at; 208992_s_at; 225289_at	STAT3		x	205805_s_at	ROR1
208991_at; 208992_s_at; 225289_at	STAT3		x	205945_at	IL6RA
208991_at; 208992_s_at; 225289_at	STAT3		x	206412_at	Fer
208991_at; 208992_s_at; 225289_at	STAT3		x	206665_s_at; 212312_at	Bcl-XL
208991_at;	STAT3		x	207980_s_at;	CITED2

IDs in active data set	Object name	miR-128 tgts_miRanda	Transcription Factor	IDs of corresponding network object from GeneGo network	Corresponding network object name
1552703_s_at; 206011_at; 209970_x_at; 211366_x_at; 211367_s_at; 211368_s_at	Caspase-1	x		1552701_a_at; 1552703_s_at	Pseudo-ICE
208992_s_at; 225289_at				209357_at	
208991_at; 208992_s_at; 225289_at	STAT3		x	208607_s_at; 214456_x_at	SAA1
208991_at; 208992_s_at; 225289_at	STAT3		x	208607_s_at; 214456_x_at	SAA2
208991_at; 208992_s_at; 225289_at	STAT3		x	208920_at	Sorcin
208991_at; 208992_s_at; 225289_at	STAT3		x	209270_at	LAMB3
208991_at; 208992_s_at; 225289_at	STAT3		x	209339_at	SIAH2
208991_at; 208992_s_at; 225289_at	STAT3		x	209341_s_at	IKK-beta
208991_at; 208992_s_at; 225289_at	STAT3		x	209466_x_at; 211737_x_at	Pleiotrophin (OSF1)
208991_at; 208992_s_at; 225289_at	STAT3		x	209589_s_at	Ephrin-B receptor 2
208991_at; 208992_s_at; 225289_at	STAT3		x	209925_at	Occludin
208991_at; 208992_s_at; 225289_at	STAT3		x	210294_at	Tapasin
208991_at; 208992_s_at; 225289_at	STAT3		x	210449_x_at; 211561_x_at	p38alpha (MAPK14)
208991_at; 208992_s_at; 225289_at	STAT3		x	210477_x_at; 226048_at	JNK1(MAPK8)
208991_at;	STAT3		x	210512_s_at;	VEGF-A

IDs in active data set	Object name	miR-128 tgts_miRanda	Transcription Factor	IDs of corresponding network object from GeneGo network	Corresponding network object name
1552703_s_at; 206011_at; 209970_x_at; 211366_x_at; 211367_s_at; 211368_s_at	Caspase-1	x		1552701_a_at; 1552703_s_at	Pseudo-ICE
208992_s_at; 225289_at				211527_x_at	
208991_at; 208992_s_at; 225289_at	STAT3		x	211148_s_at	Angiopoietin 2
208991_at; 208992_s_at; 225289_at	STAT3		x	211354_s_at; 211355_x_at; 211356_x_at	Leptin receptor
208991_at; 208992_s_at; 225289_at	STAT3		x	211506_s_at	IL-8
208991_at; 208992_s_at; 225289_at	STAT3		x	212218_s_at	FASN
208991_at; 208992_s_at; 225289_at	STAT3		x	212533_at	Wee1
208991_at; 208992_s_at; 225289_at	STAT3		x	213003_s_at	C/EBPdelta
208991_at; 208992_s_at; 225289_at	STAT3		x	213324_at	c-Src
208991_at; 208992_s_at; 225289_at	STAT3		x	213852_at; 217856_at	RBM8 (Y14)
208991_at; 208992_s_at; 225289_at	STAT3		x	213972_at	FoxD1
208991_at; 208992_s_at; 225289_at	STAT3		x	214015_at; 226572_at; 228662_at	SOCS7
208991_at; 208992_s_at; 225289_at	STAT3		x	214073_at	Cortactin
208991_at; 208992_s_at; 225289_at	STAT3		x	214462_at; 227542_at	SOCS6
208991_at;	STAT3		x	214856_at;	Beta-fodrin

IDs in active data set	Object name	miR-128 tgts_miRanda	Transcription Factor	IDs of corresponding network object from GeneGo network	Corresponding network object name
1552703_s_at; 206011_at; 209970_x_at; 211366_x_at; 211367_s_at; 211368_s_at	Caspase-1	x		1552701_a_at; 1552703_s_at	Pseudo-ICE
208992_s_at; 225289_at				215918_s_at	
208991_at; 208992_s_at; 225289_at	STAT3		x	214948_s_at	TMF1
208991_at; 208992_s_at; 225289_at	STAT3		x	217192_s_at; 228964_at	BLIMP1 (PRDI-BF1)
208991_at; 208992_s_at; 225289_at	STAT3		x	219179_at	DACT1
208991_at; 208992_s_at; 225289_at	STAT3		x	220038_at	SGK3
208991_at; 208992_s_at; 225289_at	STAT3		x	220266_s_at	KLF4
208991_at; 208992_s_at; 225289_at	STAT3		x	221218_s_at; 223686_at	TPK1
208991_at; 208992_s_at; 225289_at	STAT3		x	221287_at; 229285_at	RNaseL
208991_at; 208992_s_at; 225289_at	STAT3		x	221685_s_at	CCDC99
208991_at; 208992_s_at; 225289_at	STAT3		x	222077_s_at	RacGAP1
208991_at; 208992_s_at; 225289_at	STAT3		x	222539_at	CLN6
208991_at; 208992_s_at; 225289_at	STAT3		x	222557_at	Stathmin 3
208991_at; 208992_s_at; 225289_at	STAT3		x	223158_s_at	NEK6
208991_at;	STAT3		x	223679_at	Beta-catenin

IDs in active data set	Object name	miR-128 tgts_miRanda	Transcription Factor	IDs of corresponding network object from GeneGo network	Corresponding network object name
1552703_s_at; 206011_at; 209970_x_at; 211366_x_at; 211367_s_at; 211368_s_at	Caspase-1	x		1552701_a_at; 1552703_s_at	Pseudo-ICE
208992_s_at; 225289_at					
208991_at; 208992_s_at; 225289_at	STAT3		x	223834_at	PD-L1
208991_at; 208992_s_at; 225289_at	STAT3		x	224368_s_at	NDRG3
208991_at; 208992_s_at; 225289_at	STAT3		x	224772_at	NAV1
208991_at; 208992_s_at; 225289_at	STAT3		x	224994_at	CaMK II delta
208991_at; 208992_s_at; 225289_at	STAT3		x	226440_at	JKAP
208991_at; 208992_s_at; 225289_at	STAT3		x	226563_at; 235598_at	SMAD2
208991_at; 208992_s_at; 225289_at	STAT3		x	227114_at	RNF214
208991_at; 208992_s_at; 225289_at	STAT3		x	227249_at	NudE
208991_at; 208992_s_at; 225289_at	STAT3		x	230821_at	ZNF148
208991_at; 208992_s_at; 225289_at	STAT3		x	242916_at	CEP110
208991_at; 208992_s_at; 225289_at	STAT3		x	244463_at	ADAM23
209390_at	Hamartin			1553034_at; 1554237_at; 227785_at	SDCCAG8
209390_at	Hamartin			200974_at	ACTA2

IDs in active data set	Object name	miR-128 tgts_miRanda	Transcription Factor	IDs of corresponding network object from GeneGo network	Corresponding network object name
1552703_s_at; 206011_at; 209970_x_at; 211366_x_at; 211367_s_at; 211368_s_at	Caspase-1	x		1552701_a_at; 1552703_s_at	Pseudo-ICE
209390_at	Hamartin			201939_at	PLK2
209390_at	Hamartin			202240_at	PLK1
209390_at	Hamartin			202336_s_at	PAM
209390_at	Hamartin			202677_at	p120GAP
209390_at	Hamartin			202762_at	ROCK2
209390_at	Hamartin			203213_at; 203214_x_at; 210559_s_at	CDK1 (p34)
209390_at	Hamartin			203565_s_at	MAT1
209390_at	Hamartin			204969_s_at; 212398_at	RDX (radixin)
209390_at	Hamartin			206377_at	FOXF2
209390_at	Hamartin			207738_s_at	NckAP1
209390_at	Hamartin			209341_s_at	IKK-beta
209390_at	Hamartin			210449_x_at; 211561_x_at	p38alpha (MAPK14)
209390_at	Hamartin			212426_s_at	14-3-3 theta
209390_at	Hamartin			214073_at	Cortactin
209390_at	Hamartin			214211_at	FTH1
209390_at	Hamartin			217963_s_at	NADE(NGFRAP1)
209390_at	Hamartin			220266_s_at	KLF4
209390_at	Hamartin			223112_s_at	NDUFB10
209390_at	Hamartin			224889_at; 231548_at	FOXO3A
209615_s_at	PAK1			1554168_a_at; 223082_at; 235692_at	CIN85
209615_s_at	PAK1			1556499_s_at; 202310_s_at	COL1A1
209615_s_at	PAK1			1563466_at; 224823_at	MYLK1
209615_s_at	PAK1			200783_s_at	Stathmin
209615_s_at	PAK1			201087_at; 211823_s_at	Paxillin
209615_s_at	PAK1			201542_at; 201543_s_at; 210790_s_at	SAR1A
209615_s_at	PAK1			201615_x_at	Caldesmon
209615_s_at	PAK1			201864_at	RABGDIA

IDs in active data set	Object name	miR-128 tgts_miRanda	Transcription Factor	IDs of corresponding network object from GeneGo network	Corresponding network object name
1552703_s_at; 206011_at; 209970_x_at; 211366_x_at; 211367_s_at; 211368_s_at	Caspase-1	x		1552701_a_at; 1552703_s_at	Pseudo-ICE
209615_s_at	PAK1			202240_at	PLK1
209615_s_at	PAK1			202670_at	MEK1(MAP2K1)
209615_s_at	PAK1			203065_s_at; 212097_at	Caveolin-1
209615_s_at	PAK1			204444_at	KNSL1
209615_s_at	PAK1			205192_at	NIK(MAP3K14)
209615_s_at	PAK1			206665_s_at; 212312_at	Bcl-XL
209615_s_at	PAK1			210477_x_at; 226048_at	JNK1(MAPK8)
209615_s_at	PAK1			212073_at	Casein kinase II, alpha chain (CSNK2A1)
209615_s_at	PAK1			212372_at; 213067_at	MYH10
209615_s_at	PAK1			212556_at	SCRIB
209615_s_at	PAK1			212897_at	CDK11
209615_s_at	PAK1			213324_at	c-Src
209615_s_at	PAK1			213746_s_at; 214752_x_at	Filamin A
209615_s_at	PAK1			214073_at	Cortactin
209615_s_at	PAK1			222077_s_at	RacGAP1
209615_s_at	PAK1			223679_at	Beta-catenin
209615_s_at	PAK1			224986_s_at	PDK (PDPK1)
209615_s_at	PAK1			228938_at	Myelin basic protein
209615_s_at	PAK1			231801_at	NF-AT1(NFATC2)
209615_s_at	PAK1			235412_at	BETA-PIX
210294_at	Tapasin			203275_at	IRF2
210294_at	Tapasin			207565_s_at; 210224_at	MR1
210294_at	Tapasin			208812_x_at; 214459_x_at	HLA-Cw3
210294_at	Tapasin			208812_x_at; 214459_x_at	HLA-C
210294_at	Tapasin			209140_x_at	HLA-B27
210294_at	Tapasin			215313_x_at	HLA-A2
210294_at	Tapasin			215313_x_at	HLA-A
210294_at	Tapasin			217192_s_at; 228964_at	BLIMP1 (PRDI-BF1)

IDs in active data set	Object name	miR-128 tgts_miRanda	Transcription Factor	IDs of corresponding network object from GeneGo network	Corresponding network object name
1552703_s_at; 206011_at; 209970_x_at; 211366_x_at; 211367_s_at; 211368_s_at	Caspase-1	x		1552701_a_at; 1552703_s_at	Pseudo-ICE
210294_at	Tapasin			228033_at; 241725_at	E2F7
210385_s_at	ARTS-1			1554273_a_at; 219759_at	ERAP2
210385_s_at	ARTS-1			1565717_s_at	FUS
210385_s_at	ARTS-1			203275_at	IRF2
210385_s_at	ARTS-1			205945_at	IL6RA
210385_s_at	ARTS-1			217192_s_at; 228964_at	BLIMP1 (PRDI-BF1)
210385_s_at	ARTS-1			224986_s_at	PDK (PDPK1)
210385_s_at	ARTS-1			226660_at	p70 S6 kinase1
210449_x_at; 211561_x_at	p38alpha (MAPK14)	x	x	200783_s_at	Stathmin
210449_x_at; 211561_x_at	p38alpha (MAPK14)	x	x	200824_at	GSTP1
210449_x_at; 211561_x_at	p38alpha (MAPK14)	x	x	201693_s_at; 201694_s_at; 227404_s_at	EGR1
210449_x_at; 211561_x_at	p38alpha (MAPK14)	x	x	201841_s_at	HSP27
210449_x_at; 211561_x_at	p38alpha (MAPK14)	x	x	201853_s_at	CDC25B
210449_x_at; 211561_x_at	p38alpha (MAPK14)	x	x	202672_s_at	ATF-3
210449_x_at; 211561_x_at	p38alpha (MAPK14)	x	x	204404_at; 225835_at	NKCC1
210449_x_at; 211561_x_at	p38alpha (MAPK14)	x	x	204654_s_at	AP-2A
210449_x_at; 211561_x_at	p38alpha (MAPK14)	x	x	204739_at	CENP-C
210449_x_at; 211561_x_at	p38alpha (MAPK14)	x	x	204840_s_at	EEA1
210449_x_at; 211561_x_at	p38alpha (MAPK14)	x	x	205167_s_at; 216914_at; 217010_s_at	CDC25C
210449_x_at; 211561_x_at	p38alpha (MAPK14)	x	x	205731_s_at; 205732_s_at	NCOA2 (GRIP1/TIF2)
210449_x_at; 211561_x_at	p38alpha (MAPK14)	x	x	206665_s_at; 212312_at	Bcl-XL

IDs in active data set	Object name	miR-128 tgts_miRanda	Transcription Factor	IDs of corresponding network object from GeneGo network	Corresponding network object name
1552703_s_at; 206011_at; 209970_x_at; 211366_x_at; 211367_s_at; 211368_s_at	Caspase-1	x		1552701_a_at; 1552703_s_at	Pseudo-ICE
210449_x_at; 211561_x_at	p38alpha (MAPK14)	x	x	206870_at; 223438_s_at; 244689_at	PPAR-alpha
210449_x_at; 211561_x_at	p38alpha (MAPK14)	x	x	208885_at	Plastin
210449_x_at; 211561_x_at	p38alpha (MAPK14)	x	x	209332_s_at	Max
210449_x_at; 211561_x_at	p38alpha (MAPK14)	x	x	209339_at	SIAH2
210449_x_at; 211561_x_at	p38alpha (MAPK14)	x	x	209341_s_at	IKK-beta
210449_x_at; 211561_x_at	p38alpha (MAPK14)	x	x	209589_s_at	Ephrin-B receptor 2
210449_x_at; 211561_x_at	p38alpha (MAPK14)	x	x	210145_at	PA24A
210449_x_at; 211561_x_at	p38alpha (MAPK14)	x	x	210512_s_at; 211527_x_at	VEGF-A
210449_x_at; 211561_x_at	p38alpha (MAPK14)	x	x	211962_s_at	TISB
210449_x_at; 211561_x_at	p38alpha (MAPK14)	x	x	212073_at	Casein kinase II, alpha chain (CSNK2A1)
210449_x_at; 211561_x_at	p38alpha (MAPK14)	x	x	213746_s_at; 214752_x_at	Filamin A
210449_x_at; 211561_x_at	p38alpha (MAPK14)	x	x	218506_x_at; 221628_s_at	NP60
210449_x_at; 211561_x_at	p38alpha (MAPK14)	x	x	226440_at	JKAP
210449_x_at; 211561_x_at	p38alpha (MAPK14)	x	x	226906_s_at	ARHGAP9
210449_x_at; 211561_x_at	p38alpha (MAPK14)	x	x	228486_at	SLC44A1
210449_x_at; 211561_x_at	p38alpha (MAPK14)	x	x	228938_at	Myelin basic protein
210787_s_at	CaMKK2	x	x	1555677_s_at; 201589_at	SMC1
210787_s_at	CaMKK2	x	x	1555829_at	FAM62B
210787_s_at	CaMKK2	x	x	209615_s_at	PAK1
210787_s_at	CaMKK2	x	x	210449_x_at;	p38alpha (MAPK14)

IDs in active data set	Object name	miR-128 tgts_miRanda	Transcription Factor	IDs of corresponding network object from GeneGo network	Corresponding network object name
1552703_s_at; 206011_at; 209970_x_at; 211366_x_at; 211367_s_at; 211368_s_at	Caspase-1	x		1552701_a_at; 1552703_s_at	Pseudo-ICE
				211561_x_at	
210787_s_at	CaMKK2	x	x	213007_at	FANCI (KIAA1794)
210787_s_at	CaMKK2	x	x	213746_s_at; 214752_x_at	Filamin A
210787_s_at	CaMKK2	x	x	218832_x_at	Beta-arrestin1
210787_s_at	CaMKK2	x	x	227892_at; 240349_at	AMPK alpha 2 subunit
210787_s_at	CaMKK2	x	x	235412_at	BETA-PIX
211271_x_at	PTBP1			1553346_a_at; 224705_s_at	TNRC6A
211271_x_at	PTBP1			1558111_at; 235879_at	MBNL1
211271_x_at	PTBP1			1558561_at	Impas 1
211271_x_at	PTBP1			1565483_at	EGFR
211271_x_at	PTBP1			1565717_s_at	FUS
211271_x_at	PTBP1			1567223_at; 1567224_at	HMGA2
211271_x_at	PTBP1			200952_s_at; 200953_s_at	Cyclin D2
211271_x_at	PTBP1			201087_at; 211823_s_at	Paxillin
211271_x_at	PTBP1			202370_s_at	CBF beta
211271_x_at	PTBP1			203275_at	IRF2
211271_x_at	PTBP1			203529_at; 225426_at; 225429_at	PP6C
211271_x_at	PTBP1			203831_at	R3HDM2
211271_x_at	PTBP1			204083_s_at; 212654_at	Tropomyosin-2
211271_x_at	PTBP1			204455_at	BPAG1
211271_x_at	PTBP1			204760_s_at; 31637_s_at	REV-ERBalpha
211271_x_at	PTBP1			205436_s_at; 213344_s_at	Histone H2AX
211271_x_at	PTBP1			206967_at	Cyclin T1
211271_x_at	PTBP1			208819_at	Rab-8
211271_x_at	PTBP1			209339_at	SIAH2
211271_x_at	PTBP1			210282_at	ZNF198
211271_x_at	PTBP1			210512_s_at;	VEGF-A

IDs in active data set	Object name	miR-128 tgts_miRanda	Transcription Factor	IDs of corresponding network object from GeneGo network	Corresponding network object name
1552703_s_at; 206011_at; 209970_x_at; 211366_x_at; 211367_s_at; 211368_s_at	Caspase-1	x		1552701_a_at; 1552703_s_at	Pseudo-ICE
				211527_x_at	
211271_x_at	PTBP1			211994_at	WNK1
211271_x_at	PTBP1			213517_at	PCBP-2
211271_x_at	PTBP1			214073_at	Cortactin
211271_x_at	PTBP1			215959_at	Liprin-beta 2
211271_x_at	PTBP1			216215_s_at	RBM9
211271_x_at	PTBP1			218039_at	NUSAP1
211271_x_at	PTBP1			218683_at	PTB2
211271_x_at	PTBP1			221264_s_at	TARDBP (TDP43)
211271_x_at	PTBP1			225878_at	KIF1B
211271_x_at	PTBP1			227394_at	NCAM1
211271_x_at	PTBP1			244653_at	SET7
213136_at; 241622_at; 241623_at; 241983_at	PTPN2			1565483_at	EGFR
213136_at; 241622_at; 241623_at; 241983_at	PTPN2			203010_at	STAT5A
213136_at; 241622_at; 241623_at; 241983_at	PTPN2			203065_s_at; 212097_at	Caveolin-1
213136_at; 241622_at; 241623_at; 241983_at	PTPN2			204254_s_at; 204255_s_at; 213692_s_at	VDR
213136_at; 241622_at; 241623_at; 241983_at	PTPN2			205810_s_at	N-WASP
213136_at; 241622_at; 241623_at; 241983_at	PTPN2			205812_s_at	TMED9
213136_at; 241622_at; 241623_at;	PTPN2			208991_at; 208992_s_at; 225289_at	STAT3

IDs in active data set	Object name	miR-128 tgts_miRanda	Transcription Factor	IDs of corresponding network object from GeneGo network	Corresponding network object name
1552703_s_at; 206011_at; 209970_x_at; 211366_x_at; 211367_s_at; 211368_s_at	Caspase-1	x		1552701_a_at; 1552703_s_at	Pseudo-ICE
241983_at					
213136_at; 241622_at; 241623_at; 241983_at	PTPN2			212549_at	STAT5B
213136_at; 241622_at; 241623_at; 241983_at	PTPN2			213003_s_at	C/EBPdelta
213136_at; 241622_at; 241623_at; 241983_at	PTPN2			213324_at	c-Src
213226_at	Cyclin A2		x	1558233_s_at; 1565269_s_at	ATF-1
213226_at	Cyclin A2		x	200711_s_at; 200719_at	SKP1
213226_at	Cyclin A2		x	201549_x_at	PLU-1
213226_at	Cyclin A2		x	201897_s_at	CKS1
213226_at	Cyclin A2		x	202580_x_at	FOXM1
213226_at	Cyclin A2		x	202672_s_at	ATF-3
213226_at	Cyclin A2		x	203975_s_at; 229808_at	ChAF1 subunit A
213226_at	Cyclin A2		x	204109_s_at; 228433_at	NFYA
213226_at	Cyclin A2		x	204170_s_at	CKS2
213226_at	Cyclin A2		x	204767_s_at; 204768_s_at	FEN1
213226_at	Cyclin A2		x	205167_s_at; 216914_at; 217010_s_at	CDC25C
213226_at	Cyclin A2		x	209715_at; 212126_at	HP1 alpha
213226_at	Cyclin A2		x	209741_x_at; 216399_s_at	ZNF291
213226_at	Cyclin A2		x	213348_at	p57
213226_at	Cyclin A2		x	214881_s_at	UBF
213226_at	Cyclin A2		x	226554_at	FBI-1 (Pokemon)
213324_at	c-Src			1320_at;	PTPD1

IDs in active data set	Object name	miR-128 tgts_miRanda	Transcription Factor	IDs of corresponding network object from GeneGo network	Corresponding network object name
1552703_s_at; 206011_at; 209970_x_at; 211366_x_at; 211367_s_at; 211368_s_at	Caspase-1	x		1552701_a_at; 1552703_s_at	Pseudo-ICE
				205438_at; 226380_at; 40524_at	
213324_at	c-Src			1554168_a_at; 223082_at; 235692_at	CIN85
213324_at	c-Src			1555480_a_at; 225258_at	Migfilin
213324_at	c-Src			1555832_s_at	KLF6
213324_at	c-Src			1555960_at	HINT
213324_at	c-Src			1557458_s_at; 204657_s_at; 230459_s_at	SHB
213324_at	c-Src			1563466_at; 224823_at	MYLK1
213324_at	c-Src			201087_at; 211823_s_at	Paxillin
213324_at	c-Src			201502_s_at	NFKBIA
213324_at	c-Src			201693_s_at; 201694_s_at; 227404_s_at	EGR1
213324_at	c-Src			201874_at; 201875_s_at	MPZL1
213324_at	c-Src			202668_at; 202669_s_at	Ephrin-B2 (CTF2)
213324_at	c-Src			202668_at; 202669_s_at	Ephrin-B2
213324_at	c-Src			202677_at	p120GAP
213324_at	c-Src			203010_at	STAT5A
213324_at	c-Src			203036_s_at; 203037_s_at; 210359_at; 210360_s_at	MTSS1
213324_at	c-Src			203065_s_at; 212097_at	Caveolin-1
213324_at	c-Src			203431_s_at	p200RhoGAP
213324_at	c-Src			204254_s_at; 204255_s_at; 213692_s_at	VDR

IDs in active data set	Object name	miR-128 tgts_miRanda	Transcription Factor	IDs of corresponding network object from GeneGo network	Corresponding network object name
1552703_s_at; 206011_at; 209970_x_at; 211366_x_at; 211367_s_at; 211368_s_at	Caspase-1	x		1552701_a_at; 1552703_s_at	Pseudo-ICE
213324_at	c-Src			204363_at	Tissue factor
213324_at	c-Src			204466_s_at; 207827_x_at; 211546_x_at	Alpha-synuclein
213324_at	c-Src			204626_s_at; 204627_s_at; 204628_s_at; 216261_at	ITGB3
213324_at	c-Src			204760_s_at; 31637_s_at	TR-alpha
213324_at	c-Src			205167_s_at; 216914_at; 217010_s_at	CDC25C
213324_at	c-Src			205603_s_at; 205726_at; 217246_s_at	HDIA2
213324_at	c-Src			205880_at	PKC-mu
213324_at	c-Src			205945_at	IL6RA
213324_at	c-Src			206114_at; 227449_at; 228948_at; 229374_at	Ephrin-A receptor 4
213324_at	c-Src			206170_at	Beta-2 adrenergic receptor
213324_at	c-Src			206412_at	Fer
213324_at	c-Src			206492_at	FHIT
213324_at	c-Src			207112_s_at; 225998_at	GAB1
213324_at	c-Src			208430_s_at; 210736_x_at; 211493_x_at; 227084_at	Dystrobrevin alpha
213324_at	c-Src			208877_at; 208878_s_at	PAK2
213324_at	c-Src			209090_s_at	Endophilin B1
213324_at	c-Src			209140_x_at	HLAB
213324_at	c-Src			209341_s_at	IKK-beta
213324_at	c-Src			209589_s_at	Ephrin-B receptor 2
213324_at	c-Src			209682_at	CBL-B

IDs in active data set	Object name	miR-128 tgts_miRanda	Transcription Factor	IDs of corresponding network object from GeneGo network	Corresponding network object name
1552703_s_at; 206011_at; 209970_x_at; 211366_x_at; 211367_s_at; 211368_s_at	Caspase-1	x		1552701_a_at; 1552703_s_at	Pseudo-ICE
213324_at	c-Src			209691_s_at	IRS5(DOK4)
213324_at	c-Src			209925_at	Occludin
213324_at	c-Src			210145_at	PA24A
213324_at	c-Src			210449_x_at; 211561_x_at	p38alpha (MAPK14)
213324_at	c-Src			210512_s_at; 211527_x_at	VEGF-A
213324_at	c-Src			210684_s_at	PSD-95
213324_at	c-Src			210753_s_at; 211898_s_at; 230425_at	Ephrin-B receptor 1
213324_at	c-Src			211005_at	LAT
213324_at	c-Src			212167_s_at	BAF47
213324_at	c-Src			212549_at	STAT5B
213324_at	c-Src			213226_at	Cyclin A2
213324_at	c-Src			213795_s_at	PTPR-alpha
213324_at	c-Src			214073_at	Cortactin
213324_at	c-Src			218264_at	BCCIP
213324_at	c-Src			218832_x_at	Beta-arrestin1
213324_at	c-Src			218943_s_at	RIG-I
213324_at	c-Src			222635_s_at	EG1
213324_at	c-Src			223891_at; 244623_at	KCNQ5
213324_at	c-Src			224861_at	G-protein alpha-q
213324_at	c-Src			226020_s_at	DAB1
213324_at	c-Src			226660_at	p70 S6 kinase1
213324_at	c-Src			226666_at	DAAM1
213324_at	c-Src			227394_at	NCAM1
213324_at	c-Src			227884_at; 227891_s_at; 234168_at	TAF15
213324_at	c-Src			230190_at	NDFIP2
213517_at	PCBP-2			1552703_s_at; 206011_at; 209970_x_at; 211366_x_at; 211367_s_at; 211368_s_at	Caspase-1
213517_at	PCBP-2			1556499_s_at;	COL1A1

IDs in active data set	Object name	miR-128 tgts_miRanda	Transcription Factor	IDs of corresponding network object from GeneGo network	Corresponding network object name
1552703_s_at; 206011_at; 209970_x_at; 211366_x_at; 211367_s_at; 211368_s_at	Caspase-1	x		1552701_a_at; 1552703_s_at	Pseudo-ICE
				202310_s_at	
213517_at	PCBP-2			1559946_s_at; 201459_at	RUVBL2
213517_at	PCBP-2			200790_at	DCOR
213517_at	PCBP-2			202240_at	PLK1
213517_at	PCBP-2			205436_s_at; 213344_s_at	Histone H2AX
213517_at	PCBP-2			205552_s_at	OAS1
213517_at	PCBP-2			209077_at	MT-TRX
213517_at	PCBP-2			210684_s_at	PSD-95
213517_at	PCBP-2			213324_at	c-Src
213517_at	PCBP-2			213359_at; 221480_at	AUF1
213517_at	PCBP-2			213649_at	SFRS7 (9G8)
213517_at	PCBP-2			213746_s_at; 214752_x_at	Filamin A
213517_at	PCBP-2			214195_at	CLN2 (Tripeptidyl-peptidase I)
213517_at	PCBP-2			221264_s_at	TARDBP (TDP43)
213517_at	PCBP-2			226687_at	HYPA
213517_at	PCBP-2			237485_at	SFRS3
213517_at	PCBP-2			242260_at	Matrin-3
214073_at	Cortactin			1563466_at; 224823_at	MYLK1
214073_at	Cortactin			201615_x_at	Caldesmon
214073_at	Cortactin			201693_s_at; 201694_s_at; 227404_s_at	EGR1
214073_at	Cortactin			201841_s_at	HSP27
214073_at	Cortactin			202637_s_at; 202638_s_at; 215485_s_at	ICAM1
214073_at	Cortactin			202663_at; 202665_s_at	WaspIP
214073_at	Cortactin			203036_s_at; 203037_s_at; 210359_at; 210360_s_at	MTSS1
214073_at	Cortactin			205810_s_at	N-WASP

IDs in active data set	Object name	miR-128 tgts_miRanda	Transcription Factor	IDs of corresponding network object from GeneGo network	Corresponding network object name
1552703_s_at; 206011_at; 209970_x_at; 211366_x_at; 211367_s_at; 211368_s_at	Caspase-1	x		1552701_a_at; 1552703_s_at	Pseudo-ICE
214073_at	Cortactin			205880_at	PKC-mu
214073_at	Cortactin			206114_at; 227449_at; 228948_at; 229374_at	Ephrin-A receptor 4
214073_at	Cortactin			206412_at	Fer
214073_at	Cortactin			206846_s_at	HDAC6
214073_at	Cortactin			208877_at; 208878_s_at	PAK2
214073_at	Cortactin			213307_at; 243681_at	SHANK2
214073_at	Cortactin			214607_at	PAK3
214073_at	Cortactin			218683_at	PTB2
214073_at	Cortactin			220563_s_at	SHANK1
214073_at	Cortactin			227394_at	NCAM1
216277_at	BUB1	x		1553690_at	Sgo1
216277_at	BUB1	x		200711_s_at; 200719_at	SKP1
216277_at	BUB1	x		201458_s_at; 209974_s_at	BUB3
216277_at	BUB1	x		202240_at	PLK1
216277_at	BUB1	x		203213_at; 203214_x_at; 210559_s_at	CDK1 (p34)
216277_at	BUB1	x		203952_at	ATF-6 alpha
216277_at	BUB1	x		204026_s_at	HZwint-1
216277_at	BUB1	x		204162_at	HEC
216277_at	BUB1	x		208093_s_at	NUDEL
216277_at	BUB1	x		209891_at	SPBC25
216277_at	BUB1	x		231714_s_at; 231962_at; 236221_at	AP4B1
216277_at	BUB1	x		238068_at	ARIH2 (TRIAD1)
218117_at	RING-box protein 1	x		1555960_at	HINT
218117_at	RING-box protein 1	x		1567014_s_at; 1567015_at	NRF2
218117_at	RING-box protein 1	x		200085_s_at; 213877_x_at	Elongin B

IDs in active data set	Object name	miR-128 tgts_miRanda	Transcription Factor	IDs of corresponding network object from GeneGo network	Corresponding network object name
1552703_s_at; 206011_at; 209970_x_at; 211366_x_at; 211367_s_at; 211368_s_at	Caspase-1	x		1552701_a_at; 1552703_s_at	Pseudo-ICE
218117_at	RING-box protein 1	x		200711_s_at; 200719_at	SKP1
218117_at	RING-box protein 1	x		200952_s_at; 200953_s_at	Cyclin D2
218117_at	RING-box protein 1	x		201090_x_at; 212639_x_at; 213646_x_at	Tubulin alpha-1B
218117_at	RING-box protein 1	x		201316_at	PSMA2
218117_at	RING-box protein 1	x		201840_at	NEDD8
218117_at	RING-box protein 1	x		208838_at; 208839_s_at	TIP120A
218117_at	RING-box protein 1	x		211995_x_at; 212988_x_at	Actin cytoplasmic 2
218117_at	RING-box protein 1	x		213497_at	ABTB2
218117_at	RING-box protein 1	x		218284_at	SMAD3
218117_at	RING-box protein 1	x		218432_at; 229955_at	Fbxo3
218117_at	RING-box protein 1	x		223679_at	Beta-catenin
218117_at	RING-box protein 1	x		226660_at	p70 S6 kinase1
218117_at	RING-box protein 1	x		241747_s_at	Cullin7
218284_at	SMAD3		x	1552737_s_at	WWP2
218284_at	SMAD3		x	1553102_a_at; 212886_at	CCDC69
218284_at	SMAD3		x	1553533_at	JPH1
218284_at	SMAD3		x	1554481_a_at; 214530_x_at	EPB41
218284_at	SMAD3		x	1558199_at	Fibronectin
218284_at	SMAD3		x	1559548_at; 220028_at; 236126_at	ActRIIB
218284_at	SMAD3		x	1567223_at; 1567224_at	HMGA2

IDs in active data set	Object name	miR-128 tgts_miRanda	Transcription Factor	IDs of corresponding network object from GeneGo network	Corresponding network object name
1552703_s_at; 206011_at; 209970_x_at; 211366_x_at; 211367_s_at; 211368_s_at	Caspase-1	x		1552701_a_at; 1552703_s_at	Pseudo-ICE
218284_at	SMAD3		x	1568574_x_at	Osteopontin
218284_at	SMAD3		x	1568765_at; 202627_s_at; 202628_s_at	PAI1
218284_at	SMAD3		x	201042_at; 211003_x_at; 211573_x_at	TGM2
218284_at	SMAD3		x	201124_at	ITGB5
218284_at	SMAD3		x	201458_s_at; 209974_s_at	BUB3
218284_at	SMAD3		x	201666_at	TIMP1
218284_at	SMAD3		x	201693_s_at; 201694_s_at; 227404_s_at	EGR1
218284_at	SMAD3		x	201906_s_at	CTDSPL
218284_at	SMAD3		x	201908_at	DVL-3
218284_at	SMAD3		x	202149_at; 202150_s_at	CAS-L
218284_at	SMAD3		x	202267_at; 207517_at	LAMC2
218284_at	SMAD3		x	202672_s_at	ATF-3
218284_at	SMAD3		x	202704_at	Tob1
218284_at	SMAD3		x	203445_s_at	CTDSP2 (SCP2)
218284_at	SMAD3		x	203655_at	XRCC1
218284_at	SMAD3		x	204270_at	Ski
218284_at	SMAD3		x	204822_at	TTK
218284_at	SMAD3		x	204825_at	HPK38
218284_at	SMAD3		x	204933_s_at	Osteoprotegerin
218284_at	SMAD3		x	205014_at	HBP17
218284_at	SMAD3		x	205209_at; 208218_s_at; 208223_s_at; 213198_at	ALK-4
218284_at	SMAD3		x	205731_s_at; 205732_s_at	NCOA2 (GRIP1/TIF2)
218284_at	SMAD3		x	206649_s_at	TFE3
218284_at	SMAD3		x	206877_at; 228846_at	MAD
218284_at	SMAD3		x	206967_at	Cyclin T1

IDs in active data set	Object name	miR-128 tgts_miRanda	Transcription Factor	IDs of corresponding network object from GeneGo network	Corresponding network object name
1552703_s_at; 206011_at; 209970_x_at; 211366_x_at; 211367_s_at; 211368_s_at	Caspase-1	x		1552701_a_at; 1552703_s_at	Pseudo-ICE
218284_at	SMAD3		x	207836_s_at	RBPMS
218284_at	SMAD3		x	207980_s_at; 209357_at	CITED2
218284_at	SMAD3		x	208394_x_at	ESM-1
218284_at	SMAD3		x	208988_at; 208989_s_at	FBXL11
218284_at	SMAD3		x	209332_s_at	Max
218284_at	SMAD3		x	209892_at; 209893_s_at	FUT4
218284_at	SMAD3		x	209925_at	Occludin
218284_at	SMAD3		x	210282_at	ZNF198
218284_at	SMAD3		x	210477_x_at; 226048_at	JNK1(MAPK8)
218284_at	SMAD3		x	210512_s_at; 211527_x_at	VEGF-A
218284_at	SMAD3		x	211114_x_at; 211115_x_at	SIP1
218284_at	SMAD3		x	211711_s_at; 242622_x_at	PTEN
218284_at	SMAD3		x	211994_at	WNK1
218284_at	SMAD3		x	212213_x_at; 212214_at; 214306_at	OPA1
218284_at	SMAD3		x	212533_at	Wee1
218284_at	SMAD3		x	213003_s_at	C/EBPdelta
218284_at	SMAD3		x	213032_at; 213033_s_at	NFIB
218284_at	SMAD3		x	213746_s_at; 214752_x_at	Filamin A
218284_at	SMAD3		x	214856_at; 215918_s_at	Beta-fodrin
218284_at	SMAD3		x	215237_at	Zizimin 1
218284_at	SMAD3		x	216004_s_at	PREP1
218284_at	SMAD3		x	217542_at	MDM2
218284_at	SMAD3		x	217875_s_at; 222449_at; 222450_at	PMEPA1
218284_at	SMAD3		x	217941_s_at; 222473_s_at	ERBIN

IDs in active data set	Object name	miR-128 tgts_miRanda	Transcription Factor	IDs of corresponding network object from GeneGo network	Corresponding network object name
1552703_s_at; 206011_at; 209970_x_at; 211366_x_at; 211367_s_at; 211368_s_at	Caspase-1	x		1552701_a_at; 1552703_s_at	Pseudo-ICE
218284_at	SMAD3		x	218432_at; 229955_at	Fbxo3
218284_at	SMAD3		x	218667_at	Praja-1
218284_at	SMAD3		x	219213_at	JAM2
218284_at	SMAD3		x	219249_s_at	FKBP10
218284_at	SMAD3		x	220266_s_at	KLF4
218284_at	SMAD3		x	220938_s_at	GMEB1
218284_at	SMAD3		x	221744_at; 224730_at; 224748_at	HAN11
218284_at	SMAD3		x	222430_s_at	YTHDF2
218284_at	SMAD3		x	223713_at	RSHL2
218284_at	SMAD3		x	224164_at	TPM3
218284_at	SMAD3		x	225066_at	PPP2R2D
218284_at	SMAD3		x	226066_at	MITF
218284_at	SMAD3		x	226563_at; 235598_at	SMAD2
218284_at	SMAD3		x	226795_at; 235012_at	LRCH1
218284_at	SMAD3		x	228942_s_at	AIP1 (DAB2ip)
218284_at	SMAD3		x	229399_at	C10orf118
218284_at	SMAD3		x	236814_at	MDM4
218284_at	SMAD3		x	241922_at	LMO4
218284_at	SMAD3		x	243589_at	KIAA1267
220671_at	Nocturnin			204980_at	CLOCK
220671_at	Nocturnin			208991_at; 208992_s_at; 225289_at	STAT3
220671_at	Nocturnin			209674_at	CRY1
220671_at	Nocturnin			211962_s_at	TISB
220671_at	Nocturnin			212695_at	CRY2
220671_at	Nocturnin			213003_s_at	C/EBPdelta
220671_at	Nocturnin			225565_at; 225572_at	CREB1
222040_at	hnRNP A1	x		1554408_a_at	TK1
222040_at	hnRNP A1	x		1556499_s_at; 202310_s_at	COL1A1
222040_at	hnRNP A1	x		1557278_s_at;	Karyopherin beta 2

IDs in active data set	Object name	miR-128 tgts_miRanda	Transcription Factor	IDs of corresponding network object from GeneGo network	Corresponding network object name
1552703_s_at; 206011_at; 209970_x_at; 211366_x_at; 211367_s_at; 211368_s_at	Caspase-1	x		1552701_a_at; 1552703_s_at	Pseudo-ICE
				212635_at; 225765_at	
222040_at	hnRNP A1	x		200072_s_at	hnRNP M
222040_at	hnRNP A1	x		200952_s_at; 200953_s_at	Cyclin D2
222040_at	hnRNP A1	x		201502_s_at	NFKBIA
222040_at	hnRNP A1	x		203213_at; 203214_x_at; 210559_s_at	CDK1 (p34)
222040_at	hnRNP A1	x		204739_at	CENP-C
222040_at	hnRNP A1	x		204767_s_at; 204768_s_at	FEN1
222040_at	hnRNP A1	x		205192_at	NIK(MAP3K14)
222040_at	hnRNP A1	x		205436_s_at; 213344_s_at	Histone H2AX
222040_at	hnRNP A1	x		205731_s_at; 205732_s_at	NCOA2 (GRIP1/TIF2)
222040_at	hnRNP A1	x		206967_at	Cyclin T1
222040_at	hnRNP A1	x		208151_x_at; 208719_s_at; 213998_s_at	DDX17
222040_at	hnRNP A1	x		208801_at	SRP72
222040_at	hnRNP A1	x		209068_at	hnRNP D-like
222040_at	hnRNP A1	x		212427_at	ECM29
222040_at	hnRNP A1	x		212756_s_at	UBR2
222040_at	hnRNP A1	x		214639_s_at	HOXA1
222040_at	hnRNP A1	x		218542_at	Cep55
222040_at	hnRNP A1	x		218832_x_at	Beta-arrestin1
222040_at	hnRNP A1	x		220052_s_at; 223776_x_at	TIN-2
222040_at	hnRNP A1	x		220613_s_at	SYTL2
222040_at	hnRNP A1	x		221264_s_at	TARDBP (TDP43)
222040_at	hnRNP A1	x		222430_s_at	YTHDF2
222040_at	hnRNP A1	x		222749_at	SUFU
222040_at	hnRNP A1	x		223679_at	Beta-catenin
222040_at	hnRNP A1	x		225310_at	hnRNP-G
222040_at	hnRNP A1	x		226404_at	CAPER
222040_at	hnRNP A1	x		226921_at	UBR1

IDs in active data set	Object name	miR-128 tgts_miRanda	Transcription Factor	IDs of corresponding network object from GeneGo network	Corresponding network object name
1552703_s_at; 206011_at; 209970_x_at; 211366_x_at; 211367_s_at; 211368_s_at	Caspase-1	x		1552701_a_at; 1552703_s_at	Pseudo-ICE
223679_at	Beta-catenin			1553764_a_at; 225806_at	Ajuba
223679_at	Beta-catenin		x	1554168_a_at; 223082_at; 235692_at	CIN85
223679_at	Beta-catenin		x	1557141_at	INMT
223679_at	Beta-catenin		x	1559946_s_at; 201459_at	RUVBL2
223679_at	Beta-catenin		x	1565034_s_at	MLL1 (HRX)
223679_at	Beta-catenin		x	1565717_s_at	LXR-alpha
223679_at	Beta-catenin		x	1565717_s_at	FUS
223679_at	Beta-catenin		x	200072_s_at	hnRNP M
223679_at	Beta-catenin		x	200632_s_at	NDRG1
223679_at	Beta-catenin		x	200711_s_at; 200719_at	SKP1
223679_at	Beta-catenin		x	200952_s_at; 200953_s_at	Cyclin D2
223679_at	Beta-catenin		x	201087_at; 211823_s_at	Paxillin
223679_at	Beta-catenin		x	201693_s_at; 201694_s_at; 227404_s_at	EGR1
223679_at	Beta-catenin		x	201841_s_at	HSP27
223679_at	Beta-catenin		x	201908_at	DVL-3
223679_at	Beta-catenin		x	202094_at; 202095_s_at	Survivin
223679_at	Beta-catenin		x	202240_at	PLK1
223679_at	Beta-catenin		x	202313_at	PPP2R2A
223679_at	Beta-		x	202360_at	MAML1

IDs in active data set	Object name	miR-128 tgts_miRanda	Transcription Factor	IDs of corresponding network object from GeneGo network	Corresponding network object name
1552703_s_at; 206011_at; 209970_x_at; 211366_x_at; 211367_s_at; 211368_s_at	Caspase-1	x		1552701_a_at; 1552703_s_at	Pseudo-ICE
	catenin				
223679_at	Beta-catenin		x	202580_x_at	FOXM1
223679_at	Beta-catenin		x	203065_s_at; 212097_at	Caveolin-1
223679_at	Beta-catenin		x	203220_s_at; 203222_s_at	TLE1
223679_at	Beta-catenin		x	203408_s_at	SATB1
223679_at	Beta-catenin		x	203431_s_at	p200RhoGAP
223679_at	Beta-catenin		x	204254_s_at; 204255_s_at; 213692_s_at	VDR
223679_at	Beta-catenin		x	204466_s_at; 207827_x_at; 211546_x_at	Alpha-synuclein
223679_at	Beta-catenin		x	204609_at	DIPA
223679_at	Beta-catenin		x	204641_at; 211080_s_at	Nek2A
223679_at	Beta-catenin		x	204654_s_at	AP-2A
223679_at	Beta-catenin		x	204760_s_at; 31637_s_at	TR-alpha
223679_at	Beta-catenin		x	204920_at; 217564_s_at	CPSM
223679_at	Beta-catenin		x	204933_s_at	Osteoprotegerin
223679_at	Beta-catenin		x	205731_s_at; 205732_s_at	NCOA2 (GRIP1/TIF2)
223679_at	Beta-catenin		x	205880_at	PKC-mu
223679_at	Beta-catenin		x	206412_at	Fer
223679_at	Beta-catenin		x	206492_at	FHIT
223679_at	Beta-catenin		x	206846_s_at	HDAC6

IDs in active data set	Object name	miR-128 tgts_miRanda	Transcription Factor	IDs of corresponding network object from GeneGo network	Corresponding network object name
1552703_s_at; 206011_at; 209970_x_at; 211366_x_at; 211367_s_at; 211368_s_at	Caspase-1	x		1552701_a_at; 1552703_s_at	Pseudo-ICE
223679_at	Beta-catenin		x	208865_at; 208866_at; 208867_s_at	Casein kinase I alpha
223679_at	Beta-catenin		x	209002_s_at	CALCOCO1
223679_at	Beta-catenin		x	209311_at	Bcl-W
223679_at	Beta-catenin		x	209341_s_at	IKK-beta
223679_at	Beta-catenin		x	209466_x_at; 211737_x_at	Pleiotrophin (OSF1)
223679_at	Beta-catenin		x	209753_s_at	TMPOB
223679_at	Beta-catenin		x	209938_at; 210537_s_at	ADA2
223679_at	Beta-catenin		x	210173_at; 214137_at; 227396_at	DEP-1
223679_at	Beta-catenin		x	210248_at	WNT7A
223679_at	Beta-catenin		x	210426_x_at; 210479_s_at; 226682_at; 235567_at; 236266_at	ROR-alpha
223679_at	Beta-catenin		x	210477_x_at; 226048_at	JNK1(MAPK8)
223679_at	Beta-catenin		x	210512_s_at; 211527_x_at	VEGF-A
223679_at	Beta-catenin		x	211711_s_at; 242622_x_at	PTEN
223679_at	Beta-catenin		x	212037_at	Pinin
223679_at	Beta-catenin		x	212287_at	SUZ12
223679_at	Beta-catenin		x	212556_at	SCRIB
223679_at	Beta-catenin		x	213005_s_at	ANKRD15

IDs in active data set	Object name	miR-128 tgts_miRanda	Transcription Factor	IDs of corresponding network object from GeneGo network	Corresponding network object name
1552703_s_at; 206011_at; 209970_x_at; 211366_x_at; 211367_s_at; 211368_s_at	Caspase-1	x		1552701_a_at; 1552703_s_at	Pseudo-ICE
223679_at	Beta-catenin		x	213324_at	c-Src
223679_at	Beta-catenin		x	214881_s_at	UBF
223679_at	Beta-catenin		x	215273_s_at	ADA3-like protein
223679_at	Beta-catenin		x	215520_at	PYGO1
223679_at	Beta-catenin		x	217941_s_at; 222473_s_at	ERBIN
223679_at	Beta-catenin		x	218264_at	BCCIP
223679_at	Beta-catenin		x	218284_at	SMAD3
223679_at	Beta-catenin		x	219179_at	DACT1
223679_at	Beta-catenin		x	219908_at	DKK2
223679_at	Beta-catenin		x	220266_s_at	KLF4
223679_at	Beta-catenin		x	222749_at	SUFU
223679_at	Beta-catenin		x	223111_x_at	ARID4B
223679_at	Beta-catenin		x	223376_s_at	Brain protein I3
223679_at	Beta-catenin		x	224013_s_at	SOX7
223679_at	Beta-catenin		x	224889_at; 231548_at	FOXO3A
223679_at	Beta-catenin		x	224994_at	CaMK II delta
223679_at	Beta-catenin		x	225310_at	hnRNP-G
223679_at	Beta-catenin		x	225712_at	Gemin5
223679_at	Beta-catenin		x	226066_at	MITF
223679_at	Beta-		x	226115_at	ELYS

IDs in active data set	Object name	miR-128 tgts_miRanda	Transcription Factor	IDs of corresponding network object from GeneGo network	Corresponding network object name
1552703_s_at; 206011_at; 209970_x_at; 211366_x_at; 211367_s_at; 211368_s_at	Caspase-1	x		1552701_a_at; 1552703_s_at	Pseudo-ICE
	catenin				
223679_at	Beta-catenin		x	226404_at	CAPER
223679_at	Beta-catenin		x	226563_at; 235598_at	SMAD2
223679_at	Beta-catenin		x	227395_at; 231997_at	TBCEL
223679_at	Beta-catenin		x	227892_at; 240349_at	AMPK alpha 2 subunit
223679_at	Beta-catenin		x	228065_at	BCL9-2
223679_at	Beta-catenin		x	229500_at	HUEL
223679_at	Beta-catenin		x	230229_at	SAP97 (DLG1)
223679_at	Beta-catenin		x	230821_at	ZNF148
223679_at	Beta-catenin		x	230943_at	SOX17
223679_at	Beta-catenin		x	233633_at	TBLR1 (DC42)
223679_at	Beta-catenin		x	238285_at	SOX5
224889_at; 231548_at	FOXO3A		x	1555832_s_at	KLF6
224889_at; 231548_at	FOXO3A		x	1565717_s_at	LXR-alpha
224889_at; 231548_at	FOXO3A		x	1568765_at; 202627_s_at; 202628_s_at	PAI1
224889_at; 231548_at	FOXO3A		x	1570352_at	ATM
224889_at; 231548_at	FOXO3A		x	200894_s_at; 200895_s_at	FKBP4
224889_at; 231548_at	FOXO3A		x	201008_s_at; 201009_s_at; 201010_s_at	TXNIP (VDUP1)
224889_at; 231548_at	FOXO3A		x	202240_at	PLK1

IDs in active data set	Object name	miR-128 tgts_miRanda	Transcription Factor	IDs of corresponding network object from GeneGo network	Corresponding network object name
1552703_s_at; 206011_at; 209970_x_at; 211366_x_at; 211367_s_at; 211368_s_at	Caspase-1	x		1552701_a_at; 1552703_s_at	Pseudo-ICE
224889_at; 231548_at	FOXO3A		x	202580_x_at	FOXM1
224889_at; 231548_at	FOXO3A		x	203010_at	STAT5A
224889_at; 231548_at	FOXO3A		x	203065_s_at; 212097_at	Caveolin-1
224889_at; 231548_at	FOXO3A		x	203220_s_at; 203222_s_at	TLE1
224889_at; 231548_at	FOXO3A		x	206649_s_at	TFE3
224889_at; 231548_at	FOXO3A		x	207181_s_at	Caspase-7
224889_at; 231548_at	FOXO3A		x	207980_s_at; 209357_at	CITED2
224889_at; 231548_at	FOXO3A		x	208763_s_at	DSIPI (GILZ)
224889_at; 231548_at	FOXO3A		x	208786_s_at	MAP1LC3B
224889_at; 231548_at	FOXO3A		x	208997_s_at	UCP2
224889_at; 231548_at	FOXO3A		x	209077_at	MT-TRX
224889_at; 231548_at	FOXO3A		x	209341_s_at	IKK-beta
224889_at; 231548_at	FOXO3A		x	212426_s_at	14-3-3 theta
224889_at; 231548_at	FOXO3A		x	212549_at	STAT5B
224889_at; 231548_at	FOXO3A		x	212607_at; 212609_s_at; 219393_s_at; 222880_at	AKT3
224889_at; 231548_at	FOXO3A		x	213376_at	ZBTB1
224889_at; 231548_at	FOXO3A		x	217542_at	MDM2
224889_at; 231548_at	FOXO3A		x	218284_at	SMAD3
224889_at;	FOXO3A		x	220266_s_at	KLF4

IDs in active data set	Object name	miR-128 tgts_miRanda	Transcription Factor	IDs of corresponding network object from GeneGo network	Corresponding network object name
1552703_s_at; 206011_at; 209970_x_at; 211366_x_at; 211367_s_at; 211368_s_at	Caspase-1	x		1552701_a_at; 1552703_s_at	Pseudo-ICE
231548_at					
224889_at; 231548_at	FOXO3A		x	221478_at	NIX
224889_at; 231548_at	FOXO3A		x	221562_s_at	Sirtuin3
224889_at; 231548_at	FOXO3A		x	221986_s_at; 226158_at; 242088_at	KLHL24
224889_at; 231548_at	FOXO3A		x	225878_at	KIF1B
224889_at; 231548_at	FOXO3A		x	226563_at; 235598_at	SMAD2
224917_at	microRNA 21			1558532_at	Tropomyosin-1
224917_at	microRNA 21			200787_s_at; 200788_s_at	PEA15
224917_at	microRNA 21			201147_s_at; 201149_s_at; 201150_s_at	TIMP3
224917_at	microRNA 21			201489_at; 201490_s_at	Ppif
224917_at	microRNA 21			201669_s_at; 201670_s_at; 213002_at; 225897_at	MARCKS
224917_at	microRNA 21			201862_s_at	LRRFIP1
224917_at	microRNA 21			202677_at	p120GAP
224917_at	microRNA 21			203796_s_at	BCL7A
224917_at	microRNA 21			204011_at	SPRY2
224917_at	microRNA 21			204270_at	Ski
224917_at	microRNA 21			207828_s_at; 209172_s_at	CENP-F
224917_at	microRNA 21			208991_at; 208992_s_at;	STAT3

IDs in active data set	Object name	miR-128 tgts_miRanda	Transcription Factor	IDs of corresponding network object from GeneGo network	Corresponding network object name
1552703_s_at; 206011_at; 209970_x_at; 211366_x_at; 211367_s_at; 211368_s_at	Caspase-1	x		1552701_a_at; 1552703_s_at	Pseudo-ICE
				225289_at	
224917_at	microRNA 21			211711_s_at; 242622_x_at	PTEN
224917_at	microRNA 21			212594_at	PDCD4
224917_at	microRNA 21			212888_at; 213229_at	Dicer
224917_at	microRNA 21			213032_at; 213033_s_at	NFIB
224917_at	microRNA 21			213348_at	p57
224917_at	microRNA 21			218039_at	NUSAP1
224917_at	microRNA 21			218284_at	SMAD3
224917_at	microRNA 21			218662_s_at	CAP-G
224917_at	microRNA 21			218883_s_at	CENP-50
224917_at	microRNA 21			224889_at; 231548_at	FOXO3A
224986_s_at	PDK (PDPK1)			202458_at; 226279_at; 229441_at	PRSS23
224986_s_at	PDK (PDPK1)			202670_at	MEK1(MAP2K1)
224986_s_at	PDK (PDPK1)			203065_s_at; 212097_at	Caveolin-1
224986_s_at	PDK (PDPK1)			206204_at	GRB14
224986_s_at	PDK (PDPK1)			208010_s_at	70Z-PEP
224986_s_at	PDK (PDPK1)			209341_s_at	IKK-beta
224986_s_at	PDK (PDPK1)			210173_at; 214137_at; 227396_at	DEP-1
224986_s_at	PDK (PDPK1)			210477_x_at; 226048_at	JNK1(MAPK8)

IDs in active data set	Object name	miR-128 tgts_miRanda	Transcription Factor	IDs of corresponding network object from GeneGo network	Corresponding network object name
1552703_s_at; 206011_at; 209970_x_at; 211366_x_at; 211367_s_at; 211368_s_at	Caspase-1	x		1552701_a_at; 1552703_s_at	Pseudo-ICE
224986_s_at	PDK (PDPK1)			210538_s_at	c-IAP2
224986_s_at	PDK (PDPK1)			211995_x_at; 212988_x_at	Actin cytoplasmic 2
224986_s_at	PDK (PDPK1)			212037_at	Pinin
224986_s_at	PDK (PDPK1)			212426_s_at	14-3-3 theta
224986_s_at	PDK (PDPK1)			212685_s_at	TBL2
224986_s_at	PDK (PDPK1)			212724_at	RhoE
224986_s_at	PDK (PDPK1)			213324_at	c-Src
224986_s_at	PDK (PDPK1)			218284_at	SMAD3
224986_s_at	PDK (PDPK1)			218373_at; 223894_s_at	FTS
224986_s_at	PDK (PDPK1)			220038_at	SGK3
224986_s_at	PDK (PDPK1)			224889_at; 231548_at	FOXO3A
224986_s_at	PDK (PDPK1)			226563_at; 235598_at	SMAD2
224986_s_at	PDK (PDPK1)			226660_at	p70 S6 kinase1
225198_at	VAPA			1565483_at	EGFR
225198_at	VAPA			201626_at	INSIG1
225198_at	VAPA			201693_s_at; 201694_s_at; 227404_s_at	EGR1
225198_at	VAPA			202395_at	NSF
225198_at	VAPA			203952_at	ATF-6 alpha
225198_at	VAPA			204466_s_at; 207827_x_at; 211546_x_at	Alpha-synuclein
225198_at	VAPA			208078_s_at	SIK
225198_at	VAPA			209925_at	Occludin
225198_at	VAPA			213326_at	VAMP1

IDs in active data set	Object name	miR-128 tgts_miRanda	Transcription Factor	IDs of corresponding network object from GeneGo network	Corresponding network object name
1552703_s_at; 206011_at; 209970_x_at; 211366_x_at; 211367_s_at; 211368_s_at	Caspase-1	x		1552701_a_at; 1552703_s_at	Pseudo-ICE
225198_at	VAPA			225218_at	Protrudin
225198_at	VAPA			225565_at; 225572_at	CREB1
225399_at	Sen15			1566151_at; 204370_at	Clp1
225399_at	Sen15			218132_s_at	Sen34
225399_at	Sen15			218837_s_at	UBE2D4
225399_at	Sen15			241402_at	Sen54
225399_at	Sen15			241922_at	LMO4
225565_at; 225572_at	CREB1		x	1553402_a_at	HLA-H
225565_at; 225572_at	CREB1		x	1554503_a_at	OSCAR
225565_at; 225572_at	CREB1		x	1555279_at; 1555281_x_at; 219094_at	ARMC8
225565_at; 225572_at	CREB1		x	1558111_at; 235879_at	MBNL1
225565_at; 225572_at	CREB1		x	1558199_at	Fibronectin
225565_at; 225572_at	CREB1		x	1558233_s_at; 1565269_s_at	ATF-1
225565_at; 225572_at	CREB1		x	1559548_at; 220028_at; 236126_at	ActRIIB
225565_at; 225572_at	CREB1		x	1560916_a_at; 212792_at	DPY19L1
225565_at; 225572_at	CREB1		x	1568574_x_at	Osteopontin
225565_at; 225572_at	CREB1		x	1568765_at; 202627_s_at; 202628_s_at	PAI1
225565_at; 225572_at	CREB1		x	1568856_at; 1568857_a_at; 201383_s_at; 201384_s_at	NBR1
225565_at; 225572_at	CREB1		x	1570352_at	ATM
225565_at;	CREB1		x	200726_at	PP1-cat gamma

IDs in active data set	Object name	miR-128 tgts_miRanda	Transcription Factor	IDs of corresponding network object from GeneGo network	Corresponding network object name
1552703_s_at; 206011_at; 209970_x_at; 211366_x_at; 211367_s_at; 211368_s_at	Caspase-1	x		1552701_a_at; 1552703_s_at	Pseudo-ICE
225572_at					
225565_at; 225572_at	CREB1		x	200787_s_at; 200788_s_at	PEA15
225565_at; 225572_at	CREB1		x	200790_at	DCOR
225565_at; 225572_at	CREB1		x	200952_s_at; 200953_s_at	Cyclin D2
225565_at; 225572_at	CREB1		x	201147_s_at; 201149_s_at; 201150_s_at	TIMP3
225565_at; 225572_at	CREB1		x	201313_at	ENO2
225565_at; 225572_at	CREB1		x	201487_at; 225647_s_at	Cathepsin C
225565_at; 225572_at	CREB1		x	201693_s_at; 201694_s_at; 227404_s_at	EGR1
225565_at; 225572_at	CREB1		x	201882_x_at; 201883_s_at; 211631_x_at; 216627_s_at; 229403_at	B4GT1
225565_at; 225572_at	CREB1		x	202068_s_at	LDLR
225565_at; 225572_at	CREB1		x	202130_at	RIOK3
225565_at; 225572_at	CREB1		x	202149_at; 202150_s_at	CAS-L
225565_at; 225572_at	CREB1		x	202241_at; 235641_at	C8FW (GIG2)
225565_at; 225572_at	CREB1		x	202260_s_at	MUNC18
225565_at; 225572_at	CREB1		x	202314_at	CYP51A1
225565_at; 225572_at	CREB1		x	202360_at	MAML1
225565_at; 225572_at	CREB1		x	202380_s_at	NKTR
225565_at;	CREB1		x	202519_at;	MLXIP

IDs in active data set	Object name	miR-128 tgts_miRanda	Transcription Factor	IDs of corresponding network object from GeneGo network	Corresponding network object name
1552703_s_at; 206011_at; 209970_x_at; 211366_x_at; 211367_s_at; 211368_s_at	Caspase-1	x		1552701_a_at; 1552703_s_at	Pseudo-ICE
225572_at				225157_at	
225565_at; 225572_at	CREB1		x	202637_s_at; 202638_s_at; 215485_s_at	ICAM1
225565_at; 225572_at	CREB1		x	202672_s_at	ATF-3
225565_at; 225572_at	CREB1		x	202704_at	Tob1
225565_at; 225572_at	CREB1		x	202728_s_at	LTBP1
225565_at; 225572_at	CREB1		x	203010_at	STAT5A
225565_at; 225572_at	CREB1		x	203146_s_at; 238569_at	GBR1
225565_at; 225572_at	CREB1		x	203455_s_at; 210592_s_at; 213988_s_at	SSAT
225565_at; 225572_at	CREB1		x	203502_at	PMGE
225565_at; 225572_at	CREB1		x	203504_s_at; 203505_at; 216066_at	ABCA1
225565_at; 225572_at	CREB1		x	203655_at	XRCC1
225565_at; 225572_at	CREB1		x	203856_at	VRK1
225565_at; 225572_at	CREB1		x	203952_at	ATF-6 alpha
225565_at; 225572_at	CREB1		x	204162_at	HEC
225565_at; 225572_at	CREB1		x	204170_s_at	CKS2
225565_at; 225572_at	CREB1		x	204184_s_at	GRK3
225565_at; 225572_at	CREB1		x	204254_s_at; 204255_s_at; 213692_s_at	VDR
225565_at; 225572_at	CREB1		x	204270_at	Ski

IDs in active data set	Object name	miR-128 tgts_miRanda	Transcription Factor	IDs of corresponding network object from GeneGo network	Corresponding network object name
1552703_s_at; 206011_at; 209970_x_at; 211366_x_at; 211367_s_at; 211368_s_at	Caspase-1	x		1552701_a_at; 1552703_s_at	Pseudo-ICE
225565_at; 225572_at	CREB1		x	204279_at	PSMB9
225565_at; 225572_at	CREB1		x	204318_s_at	GTSE1
225565_at; 225572_at	CREB1		x	204654_s_at	AP-2A
225565_at; 225572_at	CREB1		x	204732_s_at; 210994_x_at; 210995_s_at	TRIM23
225565_at; 225572_at	CREB1		x	204752_x_at; 215773_x_at	PARP-2
225565_at; 225572_at	CREB1		x	204760_s_at; 31637_s_at	TR-alpha
225565_at; 225572_at	CREB1		x	204783_at	MLF1
225565_at; 225572_at	CREB1		x	204920_at; 217564_s_at	CPSM
225565_at; 225572_at	CREB1		x	204926_at; 210511_s_at	Activin beta A
225565_at; 225572_at	CREB1		x	204933_s_at	Osteoprotegerin
225565_at; 225572_at	CREB1		x	205067_at; 39402_at	IL-1 beta
225565_at; 225572_at	CREB1		x	205235_s_at	KRMP1
225565_at; 225572_at	CREB1		x	205353_s_at; 210825_s_at; 211941_s_at	RKIP
225565_at; 225572_at	CREB1		x	205579_at; 205580_s_at	Histamine H1 receptor
225565_at; 225572_at	CREB1		x	205715_at	BST1
225565_at; 225572_at	CREB1		x	205805_s_at	ROR1
225565_at; 225572_at	CREB1		x	205857_at	VMAT2
225565_at; 225572_at	CREB1		x	205880_at	PKC-mu
225565_at;	CREB1		x	206170_at	Beta-2 adrenergic

IDs in active data set	Object name	miR-128 tgts_miRanda	Transcription Factor	IDs of corresponding network object from GeneGo network	Corresponding network object name
1552703_s_at; 206011_at; 209970_x_at; 211366_x_at; 211367_s_at; 211368_s_at	Caspase-1	x		1552701_a_at; 1552703_s_at	Pseudo-ICE
225572_at					receptor
225565_at; 225572_at	CREB1		x	206382_s_at; 239367_at	BDNF
225565_at; 225572_at	CREB1		x	206870_at; 223438_s_at; 244689_at	PPAR-alpha
225565_at; 225572_at	CREB1		x	206925_at	SIAT8D
225565_at; 225572_at	CREB1		x	207543_s_at	P4HA1
225565_at; 225572_at	CREB1		x	207606_s_at	ARHGAP12
225565_at; 225572_at	CREB1		x	208078_s_at	SIK
225565_at; 225572_at	CREB1		x	208423_s_at; 211887_x_at	08p22/MSR1(CD204)
225565_at; 225572_at	CREB1		x	208518_s_at	PER2
225565_at; 225572_at	CREB1		x	208591_s_at	PDE3B
225565_at; 225572_at	CREB1		x	208725_at	eIF2S2
225565_at; 225572_at	CREB1		x	208763_s_at	DSIPI (GILZ)
225565_at; 225572_at	CREB1		x	208812_x_at; 214459_x_at	HLA-Cw3
225565_at; 225572_at	CREB1		x	208832_at; 208833_s_at	Ataxin-10
225565_at; 225572_at	CREB1		x	208991_at; 208992_s_at; 225289_at	STAT3
225565_at; 225572_at	CREB1		x	208997_s_at	UCP2
225565_at; 225572_at	CREB1		x	209318_x_at	Zac1
225565_at; 225572_at	CREB1		x	209436_at; 209437_s_at; 213994_s_at	F-spondin
225565_at;	CREB1		x	209732_at	CLECSF2

IDs in active data set	Object name	miR-128 tgts_miRanda	Transcription Factor	IDs of corresponding network object from GeneGo network	Corresponding network object name
1552703_s_at; 206011_at; 209970_x_at; 211366_x_at; 211367_s_at; 211368_s_at	Caspase-1	x		1552701_a_at; 1552703_s_at	Pseudo-ICE
225572_at					
225565_at; 225572_at	CREB1		x	209822_s_at	VLDLR
225565_at; 225572_at	CREB1		x	210113_s_at	CARD7
225565_at; 225572_at	CREB1		x	210393_at	LGR5
225565_at; 225572_at	CREB1		x	210449_x_at; 211561_x_at	p38alpha (MAPK14)
225565_at; 225572_at	CREB1		x	210512_s_at; 211527_x_at	VEGF-A
225565_at; 225572_at	CREB1		x	210538_s_at	c-IAP2
225565_at; 225572_at	CREB1		x	211380_s_at	Protein kinase G1
225565_at; 225572_at	CREB1		x	211506_s_at	IL-8
225565_at; 225572_at	CREB1		x	211711_s_at; 242622_x_at	PTEN
225565_at; 225572_at	CREB1		x	211962_s_at	TISB
225565_at; 225572_at	CREB1		x	211994_at	WNK1
225565_at; 225572_at	CREB1		x	212218_s_at	FASN
225565_at; 225572_at	CREB1		x	212362_at	Ca-ATPase2
225565_at; 225572_at	CREB1		x	212372_at; 213067_at	MYH10
225565_at; 225572_at	CREB1		x	212607_at; 212609_s_at; 219393_s_at; 222880_at	AKT3
225565_at; 225572_at	CREB1		x	212724_at	RhoE
225565_at; 225572_at	CREB1		x	212739_s_at	NDPK D (mitochondrial)
225565_at; 225572_at	CREB1		x	213003_s_at	C/EBPdelta

IDs in active data set	Object name	miR-128 tgts_miRanda	Transcription Factor	IDs of corresponding network object from GeneGo network	Corresponding network object name
1552703_s_at; 206011_at; 209970_x_at; 211366_x_at; 211367_s_at; 211368_s_at	Caspase-1	x		1552701_a_at; 1552703_s_at	Pseudo-ICE
225565_at; 225572_at	CREB1		x	213226_at	Cyclin A2
225565_at; 225572_at	CREB1		x	213326_at	VAMP1
225565_at; 225572_at	CREB1		x	213348_at	p57
225565_at; 225572_at	CREB1		x	213596_at	Caspase-4
225565_at; 225572_at	CREB1		x	214462_at; 227542_at	SOCS6
225565_at; 225572_at	CREB1		x	214970_s_at; 214971_s_at	SIAT1
225565_at; 225572_at	CREB1		x	214972_at	MGEA5 (GLCNACase)
225565_at; 225572_at	CREB1		x	215346_at; 222292_at	CD40(TNFRSF5)
225565_at; 225572_at	CREB1		x	217542_at	MDM2
225565_at; 225572_at	CREB1		x	217871_s_at	MIF
225565_at; 225572_at	CREB1		x	218178_s_at	CHMP1B
225565_at; 225572_at	CREB1		x	218264_at	BCCIP
225565_at; 225572_at	CREB1		x	218457_s_at	DNMT3A
225565_at; 225572_at	CREB1		x	218495_at	ART-27
225565_at; 225572_at	CREB1		x	218832_x_at	Beta-arrestin1
225565_at; 225572_at	CREB1		x	219174_at; 61732_r_at	IFT74
225565_at; 225572_at	CREB1		x	219249_s_at	FKBP10
225565_at; 225572_at	CREB1		x	220038_at	SGK3
225565_at; 225572_at	CREB1		x	220239_at	KLHL7
225565_at;	CREB1		x	220266_s_at	KLF4

IDs in active data set	Object name	miR-128 tgts_miRanda	Transcription Factor	IDs of corresponding network object from GeneGo network	Corresponding network object name
1552703_s_at; 206011_at; 209970_x_at; 211366_x_at; 211367_s_at; 211368_s_at	Caspase-1	x		1552701_a_at; 1552703_s_at	Pseudo-ICE
225572_at					
225565_at; 225572_at	CREB1		x	221031_s_at	APOLD1
225565_at; 225572_at	CREB1		x	221085_at	TL1A(TNFSF15)
225565_at; 225572_at	CREB1		x	221436_s_at	Tome-1
225565_at; 225572_at	CREB1		x	221478_at	NIX
225565_at; 225572_at	CREB1		x	223254_s_at	KIAA1333
225565_at; 225572_at	CREB1		x	223261_at	DNA polymerase kappa
225565_at; 225572_at	CREB1		x	223474_at	C14orf4
225565_at; 225572_at	CREB1		x	223679_at	Beta-catenin
225565_at; 225572_at	CREB1		x	224505_s_at	PLCD4
225565_at; 225572_at	CREB1		x	225396_at; 237333_at; 244872_at	RBBP4 (RbAp48)
225565_at; 225572_at	CREB1		x	226066_at	MITF
225565_at; 225572_at	CREB1		x	226660_at	p70 S6 kinase1
225565_at; 225572_at	CREB1		x	227100_at	B3GALTL
225565_at; 225572_at	CREB1		x	228328_at; 235727_at	BTBD5
225565_at; 225572_at	CREB1		x	228521_s_at	RAB4B
225565_at; 225572_at	CREB1		x	228855_at	Nudt7
225565_at; 225572_at	CREB1		x	229877_at	Neogenin
225565_at; 225572_at	CREB1		x	231801_at	NF-AT1(NFATC2)
225565_at;	CREB1		x	237485_at	SFRS3

IDs in active data set	Object name	miR-128 tgts_miRanda	Transcription Factor	IDs of corresponding network object from GeneGo network	Corresponding network object name
1552703_s_at; 206011_at; 209970_x_at; 211366_x_at; 211367_s_at; 211368_s_at	Caspase-1	x		1552701_a_at; 1552703_s_at	Pseudo-ICE
225572_at					
225565_at; 225572_at	CREB1		x	241922_at	LMO4
225565_at; 225572_at	CREB1		x	242482_at	PRKAR1A
225565_at; 225572_at	CREB1		x	242930_at	OSGEP
225565_at; 225572_at	CREB1		x	244581_at	ZNF288
225565_at; 225572_at	CREB1		x	36711_at	MafF
226563_at; 235598_at	SMAD2		x	1552656_s_at; 227740_at; 235003_at	KIS
226563_at; 235598_at	SMAD2		x	1552737_s_at	WWP2
226563_at; 235598_at	SMAD2		x	1556053_at	Tpr2
226563_at; 235598_at	SMAD2		x	1556499_s_at; 202310_s_at	COL1A1
226563_at; 235598_at	SMAD2		x	1567223_at; 1567224_at	HMGA2
226563_at; 235598_at	SMAD2		x	1568713_a_at; 1569566_at	TBC1D1
226563_at; 235598_at	SMAD2		x	1568765_at; 202627_s_at; 202628_s_at	PAI1
226563_at; 235598_at	SMAD2		x	201042_at; 211003_x_at; 211573_x_at	TGM2
226563_at; 235598_at	SMAD2		x	201124_at	ITGB5
226563_at; 235598_at	SMAD2		x	201147_s_at; 201149_s_at; 201150_s_at	TIMP3
226563_at; 235598_at	SMAD2		x	201458_s_at; 209974_s_at	BUB3
226563_at; 235598_at	SMAD2		x	201906_s_at	CTDSPL

IDs in active data set	Object name	miR-128 tgts_miRanda	Transcription Factor	IDs of corresponding network object from GeneGo network	Corresponding network object name
1552703_s_at; 206011_at; 209970_x_at; 211366_x_at; 211367_s_at; 211368_s_at	Caspase-1	x		1552701_a_at; 1552703_s_at	Pseudo-ICE
226563_at; 235598_at	SMAD2		x	202122_s_at	TIP47
226563_at; 235598_at	SMAD2		x	202149_at; 202150_s_at	CAS-L
226563_at; 235598_at	SMAD2		x	202704_at	Tob1
226563_at; 235598_at	SMAD2		x	203445_s_at	CTDSP2 (SCP2)
226563_at; 235598_at	SMAD2		x	204270_at	Ski
226563_at; 235598_at	SMAD2		x	204822_at	TTK
226563_at; 235598_at	SMAD2		x	204825_at	HPK38
226563_at; 235598_at	SMAD2		x	204933_s_at	Osteoprotegerin
226563_at; 235598_at	SMAD2		x	205014_at	HBP17
226563_at; 235598_at	SMAD2		x	205209_at; 208218_s_at; 208223_s_at; 213198_at	ALK-4
226563_at; 235598_at	SMAD2		x	207836_s_at	RBPMS
226563_at; 235598_at	SMAD2		x	207980_s_at; 209357_at	CITED2
226563_at; 235598_at	SMAD2		x	208361_s_at	POLR3D
226563_at; 235598_at	SMAD2		x	208666_s_at; 208667_s_at	ST13 (Hip)
226563_at; 235598_at	SMAD2		x	208920_at	Sorcin
226563_at; 235598_at	SMAD2		x	209377_s_at	HMGN3
226563_at; 235598_at	SMAD2		x	210512_s_at; 211527_x_at	VEGF-A
226563_at; 235598_at	SMAD2		x	211114_x_at; 211115_x_at	SIP1
226563_at;	SMAD2		x	211711_s_at;	PTEN

IDs in active data set	Object name	miR-128 tgts_miRanda	Transcription Factor	IDs of corresponding network object from GeneGo network	Corresponding network object name
1552703_s_at; 206011_at; 209970_x_at; 211366_x_at; 211367_s_at; 211368_s_at	Caspase-1	x		1552701_a_at; 1552703_s_at	Pseudo-ICE
235598_at				242622_x_at	
226563_at; 235598_at	SMAD2		x	211994_at	WNK1
226563_at; 235598_at	SMAD2		x	212213_x_at; 212214_at; 214306_at	OPA1
226563_at; 235598_at	SMAD2		x	213032_at; 213033_s_at	NFIB
226563_at; 235598_at	SMAD2		x	213746_s_at; 214752_x_at	Filamin A
226563_at; 235598_at	SMAD2		x	215237_at	Zizimin 1
226563_at; 235598_at	SMAD2		x	215606_s_at	ELKS
226563_at; 235598_at	SMAD2		x	216004_s_at	PREP1
226563_at; 235598_at	SMAD2		x	217875_s_at; 222449_at; 222450_at	PMEPA1
226563_at; 235598_at	SMAD2		x	217941_s_at; 222473_s_at	ERBIN
226563_at; 235598_at	SMAD2		x	217963_s_at	NADE(NGFRAP1)
226563_at; 235598_at	SMAD2		x	218432_at; 229955_at	Fbxo3
226563_at; 235598_at	SMAD2		x	218932_at	ZNHIT6
226563_at; 235598_at	SMAD2		x	219409_at	SNIP1
226563_at; 235598_at	SMAD2		x	220266_s_at	KLF4
226563_at; 235598_at	SMAD2		x	221744_at; 224730_at; 224748_at	HAN11
226563_at; 235598_at	SMAD2		x	222407_s_at	ZFP106
226563_at; 235598_at	SMAD2		x	222489_s_at	WRNIP1
226563_at;	SMAD2		x	224710_at	Rab-34

IDs in active data set	Object name	miR-128 tgts_miRanda	Transcription Factor	IDs of corresponding network object from GeneGo network	Corresponding network object name
1552703_s_at; 206011_at; 209970_x_at; 211366_x_at; 211367_s_at; 211368_s_at	Caspase-1	x		1552701_a_at; 1552703_s_at	Pseudo-ICE
235598_at					
226563_at; 235598_at	SMAD2		x	225038_s_at	SURF6
226563_at; 235598_at	SMAD2		x	225066_at	PPP2R2D
226563_at; 235598_at	SMAD2		x	226975_at	U11/U12-65K
226563_at; 235598_at	SMAD2		x	227606_s_at; 227607_at	AMSH-2
226563_at; 235598_at	SMAD2		x	228942_s_at	AIP1 (DAB2ip)
226563_at; 235598_at	SMAD2		x	230775_s_at	SPG20
226563_at; 235598_at	SMAD2		x	235154_at	TAF3
226563_at; 235598_at	SMAD2		x	241922_at	LMO4
226563_at; 235598_at	SMAD2		x	242482_at	PRKAR1A
226660_at	p70 S6 kinase1	x		200726_at	PP1-cat gamma
226660_at	p70 S6 kinase1	x		201693_s_at; 201694_s_at; 227404_s_at	EGR1
226660_at	p70 S6 kinase1	x		202313_at	PPP2R2A
226660_at	p70 S6 kinase1	x		206686_at; 226452_at	PDK1
226660_at	p70 S6 kinase1	x		206877_at; 228846_at	MAD
226660_at	p70 S6 kinase1	x		208725_at	eIF2S2
226660_at	p70 S6 kinase1	x		210449_x_at; 211561_x_at	p38alpha (MAPK14)
226660_at	p70 S6 kinase1	x		210477_x_at; 226048_at	JNK1(MAPK8)
226660_at	p70 S6 kinase1	x		212073_at	Casein kinase II, alpha chain (CSNK2A1)

IDs in active data set	Object name	miR-128 tgts_miRanda	Transcription Factor	IDs of corresponding network object from GeneGo network	Corresponding network object name
1552703_s_at; 206011_at; 209970_x_at; 211366_x_at; 211367_s_at; 211368_s_at	Caspase-1	x		1552701_a_at; 1552703_s_at	Pseudo-ICE
226660_at	p70 S6 kinase1	x		212100_s_at; 215357_s_at	PDIP46
226660_at	p70 S6 kinase1	x		212594_at	PDCD4
226660_at	p70 S6 kinase1	x		217542_at	MDM2
226660_at	p70 S6 kinase1	x		223158_s_at	NEK6
226660_at	p70 S6 kinase1	x		231966_at	Neurabin-1
226660_at	p70 S6 kinase1	x		242550_at	eIF3S9
227703_s_at	SYTL4	x		202260_s_at	MUNC18
227703_s_at	SYTL4	x		204609_at	DIPA
227703_s_at	SYTL4	x		207018_s_at	Rab-27B
227703_s_at	SYTL4	x		207346_at	Epimorphin
227703_s_at	SYTL4	x		208819_at	Rab-8
227703_s_at	SYTL4	x		209515_s_at	Rab-27A
227703_s_at	SYTL4	x		216985_s_at	Syntaxin 3
235412_at	BETA-PIX			1554168_a_at; 223082_at; 235692_at	CIN85
235412_at	BETA-PIX			202154_x_at; 213476_x_at	Tubulin beta 3
235412_at	BETA-PIX			202395_at	NSF
235412_at	BETA-PIX			203856_at	VRK1
235412_at	BETA-PIX			208877_at; 208878_s_at	PAK2
235412_at	BETA-PIX			209682_at	CBL-B
235412_at	BETA-PIX			211208_s_at	CASK
235412_at	BETA-PIX			212372_at; 213067_at	MYH10
235412_at	BETA-PIX			212556_at	SCRIB
235412_at	BETA-PIX			213307_at; 243681_at	SHANK2
235412_at	BETA-PIX			214607_at	PAK3
235412_at	BETA-PIX			218284_at	SMAD3
235412_at	BETA-PIX			220563_s_at	SHANK1
235412_at	BETA-PIX			221725_at;	WASF2

IDs in active data set	Object name	miR-128 tgts_miRanda	Transcription Factor	IDs of corresponding network object from GeneGo network	Corresponding network object name
1552703_s_at; 206011_at; 209970_x_at; 211366_x_at; 211367_s_at; 211368_s_at	Caspase-1	x		1552701_a_at; 1552703_s_at	Pseudo-ICE
				224562_at; 224563_at	
235412_at	BETA-PIX			224681_at	G-protein alpha-12
235412_at	BETA-PIX			224861_at	G-protein alpha-q
235412_at	BETA-PIX			226563_at; 235598_at	SMAD2
235412_at	BETA-PIX			237856_at	Rap1GDS1
235473_at	MED6		x	1553993_s_at	MED25
235473_at	MED6		x	204254_s_at; 204255_s_at; 213692_s_at	VDR
235473_at	MED6		x	204535_s_at	NRSF
235473_at	MED6		x	204760_s_at; 31637_s_at	TR-alpha
235473_at	MED6		x	209363_s_at	SRB7
235473_at	MED6		x	209938_at; 210537_s_at	ADA2
235473_at	MED6		x	212897_at	CDK11
235473_at	MED6		x	215167_at	TRAP170
235473_at	MED6		x	222635_s_at	EG1
235473_at	MED6		x	224871_at	TPRG1L
235473_at	MED6		x	226563_at; 235598_at	SMAD2
235473_at	MED6		x	242707_at	DRIP130
242141_at	HDAC2		x	1552758_at; 1552760_at; 234393_at	HDAC9
242141_at	HDAC2		x	1553764_a_at; 225806_at	Ajuba
242141_at	HDAC2		x	1556499_s_at; 202310_s_at	COL1A1
242141_at	HDAC2		x	1565483_at	EGFR
242141_at	HDAC2		x	201320_at	BAF170
242141_at	HDAC2		x	201458_s_at; 209974_s_at	BUB3
242141_at	HDAC2		x	201693_s_at; 201694_s_at; 227404_s_at	EGR1
242141_at	HDAC2		x	202240_at	PLK1

IDs in active data set	Object name	miR-128 tgts_miRanda	Transcription Factor	IDs of corresponding network object from GeneGo network	Corresponding network object name
1552703_s_at; 206011_at; 209970_x_at; 211366_x_at; 211367_s_at; 211368_s_at	Caspase-1	x		1552701_a_at; 1552703_s_at	Pseudo-ICE
242141_at	HDAC2		x	202599_s_at; 202600_s_at	RIP140
242141_at	HDAC2		x	202728_s_at	LTBP1
242141_at	HDAC2		x	203298_s_at	JARID2
242141_at	HDAC2		x	203408_s_at	SATB1
242141_at	HDAC2		x	203520_s_at	TZF
242141_at	HDAC2		x	204363_at	Tissue factor
242141_at	HDAC2		x	204535_s_at	NRSF
242141_at	HDAC2		x	204760_s_at; 31637_s_at	TR-alpha
242141_at	HDAC2		x	205014_at	HBP17
242141_at	HDAC2		x	206382_s_at; 239367_at	BDNF
242141_at	HDAC2		x	206877_at; 228846_at	MAD
242141_at	HDAC2		x	207836_s_at	RBPMS
242141_at	HDAC2		x	208151_x_at; 208719_s_at; 213998_s_at	DDX17
242141_at	HDAC2		x	208991_at; 208992_s_at; 225289_at	STAT3
242141_at	HDAC2		x	209332_s_at	Max
242141_at	HDAC2		x	209715_at; 212126_at	HP1 alpha
242141_at	HDAC2		x	210282_at	ZNF198
242141_at	HDAC2		x	210684_s_at	PSD-95
242141_at	HDAC2		x	210719_s_at	HMG20B
242141_at	HDAC2		x	212057_at	KIAA0182
242141_at	HDAC2		x	212073_at	Casein kinase II, alpha chain (CSNK2A1)
242141_at	HDAC2		x	212118_at	RFP
242141_at	HDAC2		x	212167_s_at	BAF47
242141_at	HDAC2		x	212287_at	SUZ12
242141_at	HDAC2		x	213348_at	p57
242141_at	HDAC2		x	217192_s_at; 228964_at	BLIMP1 (PRDI-BF1)
242141_at	HDAC2		x	218457_s_at	DNMT3A

IDs in active data set	Object name	miR-128 tgts_miRanda	Transcription Factor	IDs of corresponding network object from GeneGo network	Corresponding network object name
1552703_s_at; 206011_at; 209970_x_at; 211366_x_at; 211367_s_at; 211368_s_at	Caspase-1	x		1552701_a_at; 1552703_s_at	Pseudo-ICE
242141_at	HDAC2		x	218619_s_at	Suv39H1
242141_at	HDAC2		x	219497_s_at; 219498_s_at; 222891_s_at	CTIP1
242141_at	HDAC2		x	219682_s_at; 225544_at	TBX3
242141_at	HDAC2		x	220085_at; 223556_at; 227349_at	HELLS (Lsh)
242141_at	HDAC2		x	220266_s_at	KLF4
242141_at	HDAC2		x	221676_s_at	CORO1C
242141_at	HDAC2		x	222895_s_at	CTIP2
242141_at	HDAC2		x	223111_x_at	ARID4B
242141_at	HDAC2		x	223392_s_at	ZNF537
242141_at	HDAC2		x	223679_at	Beta-catenin
242141_at	HDAC2		x	224974_at	SDS3
242141_at	HDAC2		x	225396_at; 237333_at; 244872_at	RBBP4 (RbAp48)
242141_at	HDAC2		x	225565_at; 225572_at	CREB1
242141_at	HDAC2		x	226554_at	FBI-1 (Pokemon)
242141_at	HDAC2		x	226563_at; 235598_at	SMAD2
242141_at	HDAC2		x	226648_at	FIH-1
242141_at	HDAC2		x	227660_at	ATR/TEM8
242141_at	HDAC2		x	231292_at	EID3
242141_at	HDAC2		x	235588_at	ESCO2
242141_at	HDAC2		x	241813_at	MBD1
242141_at	HDAC2		x	241922_at	LMO4

Supplementary Table 9. Differentially expressed genes that are targeted by NF-κB or IL-1 Beta. Differentially expressed genes following miR-7 transfection in HEY cells that are directly regulated by NF-κB, by IL-1 beta, or by both are listed. IL-1 beta (IL1B) is a transcriptional target of NF-κB and both are significantly down-regulated following miR-7 transfection. The genes that are only targeted by IL-1 beta may be differentially expressed because RELA/NF-κB is a target of miR-7 and in turn regulates IL-1 beta.

NF-κB only		IL1-β only	Common targets
ACO2	KCTD12	CASP1	ASS1
ADAM19	LIFR	DPP4	BID
ASPH	LITAF	IGFBP3	BMP2
ATXN3	MAT2A	IL1RAP	CXCL1
BACE1	MATR3	IL7	CXCL2
CALM1	NQO1	IRS1	DDIT3
CALM3	OGT	LAMC1	FN1
CBS	PAX6	ME1	HBEGF
CCND2	PCK2	MMP14	ICAM1
CCRL2	PDPN	NFKBIZ	IL8
CDK6	PHLDA1	NR1H3	MIR21
CDKN1B	PMEPA1	NRP1	NUPR1
CSNK1A1	PSME3	NRP2	PTX3
DDIT4	RRM2	PAPPA	RUNX2
DDX21	S100A6	PRLR	SAA1
DLG1	SH2B3	SERPINH1	SAA2
DTNA	SLC11A2	SLC38A2	SERPINE2
EHD1	SLC44A1	SLC7A2	SOD2
EIF4A1	ST6GAL1	SOCS7	SPP1
ERAP1	ST8SIA1	THBD	TGFA
GAS5	TFRC	THBS1	TGFB2
GFPT2	TGM2	TIMP3	TGFBR2
GLO1	TNIP1		VEGFA
GREM1	TXNIP		VEGFC
HSPA9	VIM		XIAP
IL1B	ZBED4		
IL7R			

Supplementary Table 10. Direct downstream targets of Caveolin-1 and SMAD2. Caveolin-1 and SMAD2 are both identified as 'hub genes' among the genes differentially expressed after miR-128 transfection. CAV1 is a predicted target of miR-128 but SMAD2 is not. By targeting CAV1, miR-128 indirectly regulates SMAD2 and triggers the differential expression of genes regulated by SMAD2 as well. Differentially expressed genes that are direct targets of either Caveolin-1 or SMAD2 are listed. The relative fold change of these genes following miR-128 transfection into HEY cells are also provided.

Caveolin-1 and its direct targets		SMAD2 and its direct targets	
**Gene Symbol**	**Fold change**	**Gene Symbol**	**Fold change**
CAV1	-2.44	BUB3	-1.65
CD40	2.25	COL1A1	2.40
CFL1	-1.70	DAB2IP	2.06
ENO2	1.78	DCAF7	-1.61
EPHB1	-2.66	DOCK9	1.57
FLNA	-2.09	ERC1	1.80
FOLR1	-1.86	FGFBP1	-1.55
GNAQ	1.53	HMGA2	-1.64
ITGA2	2.00	HMGN3	-1.49
ITPR1	2.09	ITGB5	-1.60
MAPK14	-1.83	KLF4	-1.42
NEDD8	-1.57	NEDD9	1.97
NGFRAP1	-1.79	NFIB	-1.54
NT5E	-1.73	OPA1	-2.03
PDE3B	-1.59	PKNOX1	1.58
PTEN	-2.74	PLIN3	-1.54
PTGS1	4.12	PRKAR1A	2.84
SEPT7	-1.98	SERPINE1	2.23
SLC9A7	1.71	SMAD2	-1.59
SMAD2	-1.59	ST13	-1.95
SPRY4	1.71	SURF6	1.55
STMN1	-1.72	TAF3	1.53
TNFRSF10A	1.70	TIMP3	-2.99
		TNFRSF11B	1.47
		TOB1	-1.69
		VEGFA	2.15
		WRNIP1	1.48
		WWP2	1.73
		ZFP106	2.10
		ZNHIT6	-1.63

Supplementary Table 11. Significantly enriched GeneGo pathway maps among the differentially expressed genes following miR-7 transfection into HEY cells. GeneGo pathway maps significantly enriched (FDR <0.05) among the differentially expressed genes after miR-7 transfection into HEY cells are listed. Also shown are the hypergeometric distribution p-values of enrichments from GeneGo and the ratio of number of genes actually present in the dataset to the number of genes in the Affymetrix array that are in each pathway. Pathways are sorted based on p-values and the most frequent pathways are highlighted using colors with pathways within a particular process marked with the same shade.

Maps	p-value	ratio
Cell adhesion_Chemokines and adhesion	1.31E-07	15/100
Cell cycle_Regulation of G1/S transition (part 1)	8.68E-07	9/38
Cell adhesion_Ephrin signaling	3.96E-06	9/45
Development_EGFR signaling pathway	1.02E-05	10/63
Development_ERBB-family signaling	1.13E-05	8/39
Development_WNT signaling pathway. Part 1. Degradation of beta-catenin in the absence WNT signaling	1.42E-05	6/20
Development_VEGF-family signaling	1.68E-05	8/41
Cytoskeleton remodeling_Cytoskeleton remodeling	3.13E-05	12/102
Neurophysiological process_Receptor-mediated axon growth repulsion	3.43E-05	8/45
Proteolysis_Putative ubiquitin pathway	3.44E-05	6/23
Development_TGF-beta-dependent induction of EMT via RhoA, PI3K and ILK.	4.05E-05	8/46
Cell adhesion_Plasmin signaling	4.85E-05	7/35
Development_TGF-beta-dependent induction of EMT via SMADs	4.85E-05	7/35
Transport_RAB5A regulation pathway	5.75E-05	6/25
Cytoskeleton remodeling_TGF, WNT and cytoskeletal remodeling	7.29E-05	12/111
Cell cycle_Regulation of G1/S transition (part 2)	7.30E-05	6/26
Apoptosis and survival_HTR1A signaling	7.54E-05	8/50
Development_Regulation of epithelial-to-mesenchymal transition (EMT)	7.67E-05	9/64
Translation _Regulation of EIF2 activity	1.01E-04	7/39
Cell adhesion_ECM remodeling	1.01E-04	8/52
Translation _Regulation of EIF4F activity	1.16E-04	8/53
Translation_Non-genomic (rapid) action of Androgen Receptor	1.19E-04	7/40
Apoptosis and survival_NO synthesis and signaling	1.51E-04	8/55

Maps	p-value	ratio
Immune response_Neurotensin-induced activation of IL-8 in colonocytes	1.64E-04	7/42
Apoptosis and survival_TNFR1 signaling pathway	1.91E-04	7/43
Role of alpha-6/beta-4 integrins in carcinoma progression	2.57E-04	7/45
Development_EPO-induced MAPK pathway	2.57E-04	7/45
dCTP/dUTP metabolism	2.66E-04	9/75
Development_GDNF family signaling	2.95E-04	7/46
Signal transduction_PTEN pathway	2.95E-04	7/46
Cytoskeleton remodeling_CDC42 in cellular processes	3.24E-04	5/22
Development_TGF-beta-dependent induction of EMT via MAPK	3.39E-04	7/47
Development_Gastrin in cell growth and proliferation	3.54E-04	8/62
Proteolysis_Role of Parkin in the Ubiquitin-Proteasomal Pathway	4.99E-04	5/24
Development_Dopamine D2 receptor transactivation of EGFR	4.99E-04	5/24
Development_TGF-beta receptor signaling	5.00E-04	7/50
Transport_RAB3 regulation pathway	5.20E-04	4/14
Development_IGF-1 receptor signaling	5.66E-04	7/51
Membrane-bound ESR1: interaction with G-proteins signaling	5.66E-04	7/51
Immune response_Role of integrins in NK cells cytotoxicity	6.57E-04	6/38
Development_WNT signaling pathway. Part 2	7.18E-04	7/53
Neurophysiological process_Dopamine D2 receptor transactivation of PDGFR in CNS	7.36E-04	5/26
Immune response_Gastrin in inflammatory response	7.37E-04	8/69
Transcription_Receptor-mediated HIF regulation	7.58E-04	6/39
Regulation of degradation of deltaF508 CFTR in CF	8.83E-04	5/27
PGE2 pathways in cancer	9.00E-04	7/55
Regulation of lipid metabolism_Stimulation of Arachidonic acid production by ACM receptors	9.80E-04	8/72
Development_Mu-type opioid receptor regulation of proliferation	1.05E-03	5/28
Cytoskeleton remodeling_FAK signaling	1.12E-03	7/57
Apoptosis and survival_BAD phosphorylation	1.14E-03	6/42
Development_Role of IL-8 in angiogenesis	1.24E-03	7/58
Development_VEGF signaling and activation	1.29E-03	6/43
Regulation of degradation of wt-CFTR	1.45E-03	4/18
Immune response_IL-4 - antiapoptotic action	1.45E-03	5/30
dATP/dITP metabolism	1.64E-03	9/96
Cytoskeleton remodeling_Fibronectin-binding integrins in cell motility	1.69E-03	5/31
Cytoskeleton remodeling_Reverse signaling by ephrin B	1.69E-03	5/31
Development_Alpha-2 adrenergic receptor activation of ERK	1.84E-03	7/62
Development_Endothelin-1/EDNRA transactivation of EGFR	1.84E-03	6/46
Development_PDGF signaling via STATs and NF-kB	1.96E-03	5/32

Maps	p-value	ratio
Development_EGFR signaling via small GTPases	1.96E-03	5/32
Transport_Macropinocytosis regulation by growth factors	2.02E-03	7/63
Development_FGF2-dependent induction of EMT	2.20E-03	4/20
Immune response_CD40 signaling	2.22E-03	7/64
Development_Angiotensin activation of ERK	2.26E-03	5/33
Development_A3 receptor signaling	2.56E-03	6/49
Development_PEDF signaling	2.56E-03	6/49
G-protein signaling_G-Protein beta/gamma signaling cascades	2.59E-03	5/34
Cell cycle_Cell cycle (generic schema)	2.66E-03	4/21
Development_EPO-induced Jak-STAT pathway	2.95E-03	5/35
Development_EGFR signaling via PIP3	3.76E-03	4/23
Development_Beta-adrenergic receptors transactivation of EGFR	3.78E-03	5/37
Development_FGFR signaling pathway	3.82E-03	6/53
Cell cycle_Influence of Ras and Rho proteins on G1/S Transition	3.82E-03	6/53
Regulation of lipid metabolism_Regulation of lipid metabolism via LXR, NF-Y and SREBP	4.25E-03	5/38
Regulation of lipid metabolism_Insulin regulation of glycogen metabolism	4.61E-03	6/55
Cell adhesion_PLAU signaling	4.77E-03	5/39
G-protein signaling_K-RAS regulation pathway	5.13E-03	4/25
Cytoskeleton remodeling_Neurofilaments	5.13E-03	4/25
Development_Prolactin receptor signaling	5.99E-03	6/58
Regulation of CFTR activity (norm and CF)	5.99E-03	6/58
Translation_Insulin regulation of translation	6.57E-03	5/42
Immune response_Signaling pathway mediated by IL-6 and IL-1	6.81E-03	4/27
Immune response_IL-17 signaling pathways	7.07E-03	6/60
Development_ACM2 and ACM4 activation of ERK	7.27E-03	5/43
Signal transduction_AKT signaling	7.27E-03	5/43
Immune response_IL-4 signaling pathway	8.01E-03	5/44
Development_Flt3 signaling	8.01E-03	5/44
Development_Activation of Erk by ACM1, ACM3 and ACM5	8.01E-03	5/44
Development_Ligand-independent activation of ESR1 and ESR2	8.01E-03	5/44
Transcription_CREB pathway	8.01E-03	5/44

Supplementary Table 12. Significantly enriched GeneGo pathway maps among the differentially expressed genes following miR-128 transfection into HEY cells. GeneGo pathway maps significantly enriched (FDR <0.05) among the differentially expressed genes after miR-128 transfection into HEY cells are listed. Also shown are the hypergeometric distribution p-values of enrichments from GeneGo and the ratio of number of genes actually present in the dataset to the number of genes in the Affymetrix array that are in each pathway. Pathways are sorted based on p-values and the most frequent pathways are highlighted using colors with those within a particular process marked with the same shade.

Maps	p-value	Ratio
Cell cycle_The metaphase checkpoint	1.01E-11	18/36
Cytoskeleton remodeling_TGF, WNT and cytoskeletal remodeling	1.29E-09	29/111
Cell cycle_Role of Nek in cell cycle regulation	1.77E-09	15/32
Transport_Clathrin-coated vesicle cycle	4.15E-09	22/71
Cell cycle_Initiation of mitosis	4.37E-09	13/25
Immune response_Histamine H1 receptor signaling in immune response	1.78E-07	16/48
Cell cycle_Role of APC in cell cycle regulation	1.79E-07	13/32
Cell cycle_Spindle assembly and chromosome separation	2.75E-07	13/33
Cytoskeleton remodeling_Cytoskeleton remodeling	2.98E-07	24/102
Proteolysis_Role of Parkin in the Ubiquitin-Proteasomal Pathway	3.68E-07	11/24
Cytoskeleton remodeling_Neurofilaments	6.14E-07	11/25
Cell cycle_Chromosome condensation in prometaphase	8.18E-07	10/21
Immune response_Gastrin in inflammatory response	1.85E-06	18/69
Neurophysiological process_Receptor-mediated axon growth repulsion	2.69E-06	14/45
Translation_Non-genomic (rapid) action of Androgen Receptor	3.55E-06	13/40
Development_PIP3 signaling in cardiac myocytes	4.80E-06	14/47
Cytoskeleton remodeling_Role of Activin A in cytoskeleton remodeling	5.34E-06	9/20
Apoptosis and survival_Apoptotic Activin A signaling	5.76E-06	10/25
Apoptosis and survival_BAD phosphorylation	6.54E-06	13/42
Immune response_Fc epsilon RI pathway	7.40E-06	15/55
Cytoskeleton remodeling_Reverse signaling by ephrin B	7.70E-06	11/31
Development_Gastrin in cell growth and proliferation	8.04E-06	16/62
Immune response_Function of MEF2 in T lymphocytes	1.07E-05	14/50
Development_Activation of Erk by ACM1, ACM3 and ACM5	1.16E-05	13/44

Maps	p-value	Ratio
Immune response_CD40 signaling	1.25E-05	16/64
Cell cycle_Role of 14-3-3 proteins in cell cycle regulation	1.38E-05	9/22
Cell adhesion_Histamine H1 receptor signaling in the interruption of cell barrier integrity	1.52E-05	13/45
Signal transduction_Calcium signaling	1.52E-05	13/45
Development_TGF-beta-dependent induction of EMT via RhoA, PI3K and ILK.	1.98E-05	13/46
Proteolysis_Putative ubiquitin pathway	2.12E-05	9/23
Cell cycle_Influence of Ras and Rho proteins on G1/S Transition	2.22E-05	14/53
Cell cycle_Role of SCF complex in cell cycle regulation	2.69E-05	10/29
Development_Role of HDAC and calcium/calmodulin-dependent kinase (CaMK) in control of skeletal myogenesis	2.79E-05	14/54
Development_Angiopoietin - Tie2 signaling	2.88E-05	11/35
Neurophysiological process_EphB receptors in dendritic spine morphogenesis and synaptogenesis	2.88E-05	11/35
Development_Slit-Robo signaling	3.76E-05	10/30
Cell adhesion_Chemokines and adhesion	3.83E-05	20/100
Signal transduction_AKT signaling	4.73E-05	12/43
Development_A2A receptor signaling	4.73E-05	12/43
Development_Regulation of epithelial-to-mesenchymal transition (EMT)	5.28E-05	15/64
Cytoskeleton remodeling_FAK signaling	5.37E-05	14/57
Cell adhesion_Cadherin-mediated cell adhesion	6.61E-05	9/26
Development_Role of IL-8 in angiogenesis	6.61E-05	14/58
Role of alpha-6/beta-4 integrins in carcinoma progression	7.72E-05	12/45
Development_Endothelin-1/EDNRA transactivation of EGFR	9.75E-05	12/46
Cytoskeleton remodeling_CDC42 in cellular processes	1.14E-04	8/22
Development_Leptin signaling via PI3K-dependent pathway	1.22E-04	12/47
Immune response_CD28 signaling	1.23E-04	13/54
Cell adhesion_Alpha-4 integrins in cell migration and adhesion	1.25E-04	10/34
G-protein signaling_G-Protein alpha-q signaling cascades	1.25E-04	10/34
Development_Role of CDK5 in neuronal development	1.25E-04	10/34
Immune response_IL-15 signaling via JAK-STAT cascade	1.63E-04	8/23
Cytoskeleton remodeling_Regulation of actin cytoskeleton by Rho GTPases	1.63E-04	8/23
Delta508-CFTR traffic / ER-to-Golgi in CF	1.82E-04	6/13
Normal wtCFTR traffic / ER-to-Golgi	1.82E-04	6/13
Immune response_Neurotensin-induced activation of IL-8 in colonocytes	1.83E-04	11/42
Immune response_TREM1 signaling pathway	1.83E-04	13/56
Immune response_IL-2 activation and signaling pathway	1.88E-04	12/49
Signal transduction_IP3 signaling	1.88E-04	12/49

Maps	p-value	Ratio
Immune response_IL-15 signaling	2.05E-04	14/64
Immune response_IL-7 signaling in B lymphocytes	2.29E-04	11/43
Apoptosis and survival_Anti-apoptotic action of Gastrin	2.29E-04	11/43
Development_A2B receptor: action via G-protein alpha s	2.31E-04	12/50
Immune response_CCR5 signaling in macrophages and T lymphocytes	2.66E-04	13/58
wtCFTR and delta508 traffic / Clathrin coated vesicles formation (norm and CF)	2.82E-04	7/19
Development_IGF-1 receptor signaling	2.82E-04	12/51
Mucin expression in CF via TLRs, EGFR signaling pathways	2.82E-04	12/51
Development_Flt3 signaling	2.86E-04	11/44
Transcription_CREB pathway	2.86E-04	11/44
Cytoskeleton remodeling_Fibronectin-binding integrins in cell motility	3.04E-04	9/31
Development_Leptin signaling via JAK/STAT and MAPK cascades	3.15E-04	8/25
Signal transduction_Activation of PKC via G-Protein coupled receptor	3.42E-04	12/52
Immune response_Human NKG2D signaling	3.43E-04	10/38
Regulation of lipid metabolism_Regulation of lipid metabolism via LXR, NF-Y and SREBP	3.43E-04	10/38
Cell cycle_Regulation of G1/S transition (part 1)	3.43E-04	10/38
Development_Angiotensin signaling via STATs	3.96E-04	9/32
Development_FGFR signaling pathway	4.13E-04	12/53
Development_A1 receptor signaling	4.13E-04	12/53
Development_Cross-talk between VEGF and Angiopoietin 1 signaling pathways	4.26E-04	8/26
Immune response_IL-10 signaling pathway	4.26E-04	8/26
Development_GDNF family signaling	4.33E-04	11/46
Immune response_MIF - the neuroendocrine-macrophage connector	4.33E-04	11/46
Signal transduction_PTEN pathway	4.33E-04	11/46
Immune response _IFN gamma signaling pathway	4.96E-04	12/54
Immune response_BCR pathway	4.96E-04	12/54
Signal transduction_Activin A signaling regulation	5.08E-04	9/33
Neurophysiological process_Circadian rhythm	5.28E-04	11/47
Cytoskeleton remodeling_Role of PKA in cytoskeleton reorganization	5.37E-04	10/40
Reproduction_Progesterone-mediated oocyte maturation	5.37E-04	10/40
Apoptosis and survival_Anti-apoptotic TNFs/NF-kB/IAP pathway	5.66E-04	8/27
PGE2 pathways in cancer	5.92E-04	12/55
Apoptosis and survival_NO synthesis and signaling	5.92E-04	12/55

Maps	p-value	Ratio
Development_EGFR signaling pathway	6.26E-04	13/63
Transport_Macropinocytosis regulation by growth factors	6.26E-04	13/63
Cell adhesion_Integrin-mediated cell adhesion and migration	6.40E-04	11/48
Development_NOTCH1-mediated pathway for NF-KB activity modulation	6.46E-04	9/34
Transport_ACM3 in salivary glands	6.64E-04	10/41
Muscle contraction_ACM regulation of smooth muscle contraction	7.03E-04	12/56
Development_A3 receptor signaling	7.71E-04	11/49
Cell cycle_Sister chromatid cohesion	7.83E-04	7/22
Transcription_Role of heterochromatin protein 1 (HP1) family in transcriptional silencing	7.83E-04	7/22
Development_EPO-induced Jak-STAT pathway	8.14E-04	9/35
Immune response_PIP3 signaling in B lymphocytes	8.14E-04	10/42
Development_TGF-beta receptor signaling	9.23E-04	11/50
Regulation of CFTR activity (norm and CF)	9.79E-04	12/58
Development_VEGF signaling and activation	9.92E-04	10/43
Cytoskeleton remodeling_Keratin filaments	1.01E-03	9/36
Development_EGFR signaling via PIP3	1.05E-03	7/23
Muscle contraction_ GPCRs in the regulation of smooth muscle tone	1.07E-03	15/83
Immune response_NFAT in immune response	1.10E-03	11/51
Transport_Macropinocytosis	1.15E-03	5/12
Immune response_IL-1 signaling pathway	1.20E-03	10/44
Muscle contraction_EDG5-mediated smooth muscle contraction	1.22E-03	8/30
Cell adhesion_Gap junctions	1.22E-03	8/30
G-protein signaling_G-Protein alpha-12 signaling pathway	1.25E-03	9/37
Cell adhesion_Role of tetraspanins in the integrin-mediated cell adhesion	1.25E-03	9/37
Development_Hedgehog and PTH signaling pathways in bone and cartilage development	1.25E-03	9/37
Development_Glucocorticoid receptor signaling	1.39E-03	7/24
Immune response_Inhibitory action of Lipoxins on pro-inflammatory TNF-alpha signaling	1.44E-03	10/45
Development_VEGF signaling via VEGFR2 - generic cascades	1.44E-03	10/45
Immune response _CCR3 signaling in eosinophils	1.46E-03	14/77
Development_WNT signaling pathway. Part 2	1.54E-03	11/53
Development_Gastrin in differentiation of the gastric mucosa	1.54E-03	9/38
Development_Angiotensin activation of Akt	1.72E-03	10/46
Immune response_ICOS pathway in T-helper cell	1.72E-03	10/46
Development_WNT5A signaling	1.72E-03	10/46
Transcription_Transcription regulation of aminoacid metabolism	1.81E-03	7/25

Maps	p-value	Ratio
Translation _Regulation of EIF2 activity	1.87E-03	9/39
G-protein signaling_Regulation of p38 and JNK signaling mediated by G-proteins	1.87E-03	9/39
Development_TGF-beta-dependent induction of EMT via MAPK	2.04E-03	10/47
Neurophysiological process_NMDA-dependent postsynaptic long-term potentiation in CA1 hippocampal neurons	2.14E-03	14/80
Development_Role of Activin A in cell differentiation and proliferation	2.25E-03	9/40
Immune response_MIF in innate immunity response	2.25E-03	9/40
Regulation of lipid metabolism_Stimulation of Arachidonic acid production by ACM receptors	2.28E-03	13/72
DNA damage_ATM / ATR regulation of G2 / M checkpoint	2.32E-03	7/26
Development_EDG5 and EDG3 in cell proliferation and differentiation	2.32E-03	7/26
Normal and pathological TGF-beta-mediated regulation of cell proliferation	2.38E-03	8/33
Development_Keratinocyte differentiation	2.45E-03	11/56
Development_WNT signaling pathway. Part 1. Degradation of beta-catenin in the absence WNT signaling	2.62E-03	6/20
Development_FGF2-dependent induction of EMT	2.62E-03	6/20
Neurophysiological process_Netrin-1 in regulation of axon guidance	2.70E-03	9/41
Development_Melanocyte development and pigmentation	2.83E-03	10/49
Development_PEDF signaling	2.83E-03	10/49
Signal transduction_Erk Interactions: Inhibition of Erk	2.91E-03	8/34
G-protein signaling_RhoA regulation pathway	2.91E-03	8/34
Neurophysiological process_GABA-A receptor life cycle	2.94E-03	7/27
Regulation of degradation of deltaF508 CFTR in CF	2.94E-03	7/27
Development_Prolactin receptor signaling	3.27E-03	11/58
Development_EDNRB signaling	3.31E-03	10/50
Apoptosis and survival_HTR1A signaling	3.31E-03	10/50
Immune response_Histamine signaling in dendritic cells	3.31E-03	10/50
Development_GM-CSF signaling	3.31E-03	10/50
Blood coagulation_GPIb-IX-V-dependent platelet activation	3.32E-03	13/75
Cell cycle_Cell cycle (generic schema)	3.45E-03	6/21
Atherosclerosis_Role of ZNF202 in regulation of expression of genes involved in Atherosclerosis	3.45E-03	6/21
Apoptosis and survival_Anti-apoptotic action of membrane-bound ESR1	3.53E-03	8/35
Development_Regulation of telomere length and cellular immortalization	3.53E-03	8/35
Development_Growth hormone signaling via STATs and PLC/IP3	3.53E-03	8/35

Maps	p-value	Ratio
Development_EDG3 signaling pathway	3.80E-03	9/43
Immune response_HTR2A-induced activation of cPLA2	3.80E-03	9/43
Signal transduction_PKA signaling	3.85E-03	10/51
Membrane-bound ESR1: interaction with G-proteins signaling	3.85E-03	10/51
Chemotaxis_Inhibitory action of lipoxins on IL-8- and Leukotriene B4-induced neutrophil migration	3.85E-03	10/51
Some pathways of EMT in cancer cells	3.85E-03	10/51
Immune response_IL-17 signaling pathways	4.29E-03	11/60
Apoptosis and survival_nAChR in apoptosis inhibition and cell cycle progression	4.53E-03	7/29
NGF activation of NF-kB	4.53E-03	7/29
Immune response_CD137 signaling in immune cell	4.53E-03	7/29
Cytoskeleton remodeling_ACM3 and ACM4 in keratinocyte migration	5.08E-03	8/37
Development_Delta-type opioid receptor mediated cardioprotection	5.08E-03	8/37
Development_Beta-adrenergic receptors transactivation of EGFR	5.08E-03	8/37
Development_Endothelin-1/EDNRA signaling	5.14E-03	10/53
Neurophysiological process_Glutamate regulation of Dopamine D1A receptor signaling	5.23E-03	9/45
Transcription_Androgen Receptor nuclear signaling	5.23E-03	9/45
Cell adhesion_Ephrin signaling	5.23E-03	9/45
Development_Thrombopoietin-regulated cell processes	5.23E-03	9/45
Cytoskeleton remodeling_RalA regulation pathway	5.54E-03	7/30
Immune response_Role of DAP12 receptors in NK cells	5.90E-03	10/54
Immune response_Role of integrins in NK cells cytotoxicity	6.02E-03	8/38
Immune response_IL-7 signaling in T lymphocytes	6.02E-03	8/38
Development_Mu-type opioid receptor signaling	6.02E-03	8/38
Chemotaxis_Lipoxin inhibitory action on fMLP-induced neutrophil chemotaxis	6.09E-03	9/46
Neurophysiological process_ACM regulation of nerve impulse	6.09E-03	9/46
Reproduction_GnRH signaling	6.49E-03	12/72
Transcription_Transcription factor Tubby signaling pathways	6.59E-03	5/17
Development_HGF signaling pathway	7.04E-03	9/47
Development_Dopamine D2 receptor transactivation of EGFR	7.06E-03	6/24
Apoptosis and survival_NO signaling in survival	7.06E-03	6/24
HIV-1 signaling via CCR5 in macrophages and T lymphocytes	7.09E-03	8/39
Development_ERBB-family signaling	7.09E-03	8/39
Cell adhesion_PLAU signaling	7.09E-03	8/39
Muscle contraction_Regulation of eNOS activity in endothelial cells	7.11E-03	11/64
Cell adhesion_Integrin inside-out signaling	7.67E-03	10/56

Maps	p-value	Ratio
Development_PDGF signaling via STATs and NF-kB	8.05E-03	7/32
Development_Neurotrophin family signaling	8.30E-03	8/40
Inhibitory action of Lipoxins on neutrophil migration	8.70E-03	10/57
Immune response_IL-23 signaling pathway	8.71E-03	6/25
Development_Angiotensin signaling via beta-Arrestin	8.71E-03	6/25
Cytoskeleton remodeling_Integrin outside-in signaling	9.30E-03	9/49
Immune response_Bacterial infections in normal airways	9.30E-03	9/49
Development_Angiotensin activation of ERK	9.57E-03	7/33
Development_Activation of astroglial cells proliferation by ACM3	9.57E-03	7/33
Development_Signaling of Beta-adrenergic receptors via Beta-arrestins	1.06E-02	6/26
Cytoskeleton remodeling_Alpha-1A adrenergic receptor-dependent inhibition of PI3K	1.09E-02	5/19
G-protein signaling_G-Protein beta/gamma signaling cascades	1.13E-02	7/34
wtCFTR and deltaF508 traffic / Membrane expression (norm and CF)	1.13E-02	7/34
Muscle contraction_Oxytocin signaling in uterus and mammary gland	1.24E-02	10/60
Immune response_CD16 signaling in NK cells	1.24E-02	11/69
G-protein signaling_G-Protein alpha-i signaling cascades	1.28E-02	6/27
Development_ACM2 and ACM4 activation of ERK	1.29E-02	8/43
Development_Angiotensin signaling via PYK2	1.29E-02	8/43
Cell adhesion_Plasmin signaling	1.32E-02	7/35
Development_TGF-beta-dependent induction of EMT via SMADs	1.32E-02	7/35
G-protein signaling_EDG5 signaling	1.32E-02	7/35
ENaC regulation in airways (normal and CF)	1.37E-02	9/52
Immune response_T cell receptor signaling pathway	1.37E-02	9/52
G-protein signaling_Proinsulin C-peptide signaling	1.37E-02	9/52
Cytoskeleton remodeling_ESR1 action on cytoskeleton remodeling and cell migration	1.37E-02	5/20
Translation_IL-2 regulation of translation	1.37E-02	5/20
Regulation of lipid metabolism_Alpha-1 adrenergic receptors signaling via arachidonic acid	1.38E-02	11/70
Development_Ligand-independent activation of ESR1 and ESR2	1.47E-02	8/44
Immune response_IL-5 signalling	1.47E-02	8/44
Blood coagulation_GPCRs in platelet aggregation	1.53E-02	11/71
Development_Delta-type opioid receptor signaling via G-protein alpha-14	1.53E-02	6/28
Translation _Regulation of EIF4F activity	1.54E-02	9/53
Immune response_IL-9 signaling pathway	1.54E-02	7/36
Cell adhesion_Tight junctions	1.54E-02	7/36
G-protein signaling_Regulation of RAC1 activity	1.54E-02	7/36

Maps	p-value	Ratio
G-protein signaling_RAC1 in cellular process	1.54E-02	7/36
Development_Alpha-2 adrenergic receptor activation of ERK	1.55E-02	10/62
Cell cycle_Nucleocytoplasmic transport of CDK/Cyclins	1.67E-02	4/14
Immune response_Delta-type opioid receptor signaling in T-cells	1.81E-02	6/29

Supplementary Table 13. Significantly enriched GeneGo pathway maps among the down-regulated genes following miR-7 transfection into HEY cells. Significantly enriched (FDR <0.05) GeneGo pathway maps among the down-regulated genes after miR-7 transfection into HEY cells are listed. Also shown are the hypergeometric distribution p-values of enrichments from GeneGo and the ratio of number of genes actually present in the dataset to the number of genes in the Affymetrix array that are in each pathway. Pathways are sorted based on enrichment p-values.

Maps	p-value	Ratio
Cell adhesion_Chemokines and adhesion	6.99E-08	14/100
Cell adhesion_Ephrin signaling	7.47E-07	9/45
Development_EGFR signaling pathway	1.70E-06	10/63
Development_ERBB-family signaling	2.57E-06	8/39
Development_VEGF-family signaling	3.83E-06	8/41
Cytoskeleton remodeling_Cytoskeleton remodeling	4.10E-06	12/102
Development_WNT signaling pathway. Part 1. Degradation of beta-catenin in the absence WNT signaling	4.47E-06	6/20
Neurophysiological process_Receptor-mediated axon growth repulsion	7.97E-06	8/45
Cytoskeleton remodeling_TGF, WNT and cytoskeletal remodeling	9.97E-06	12/111
Apoptosis and survival_HTR1A signaling	1.80E-05	8/50
Transport_RAB5A regulation pathway	1.85E-05	6/25
Cell cycle_Regulation of G1/S transition (part 1)	2.35E-05	7/38
Cell adhesion_ECM remodeling	2.42E-05	8/52
Translation _Regulation of EIF4F activity	2.79E-05	8/53
Translation _Regulation of EIF2 activity	2.81E-05	7/39
Translation_Non-genomic (rapid) action of Androgen Receptor	3.33E-05	7/40
Apoptosis and survival_NO synthesis and signaling	3.69E-05	8/55

Maps	p-value	Ratio
Immune response_Neurotensin-induced activation of IL-8 in colonocytes	4.64E-05	7/42
Apoptosis and survival_TNFR1 signaling pathway	5.43E-05	7/43
Role of alpha-6/beta-4 integrins in carcinoma progression	7.35E-05	7/45
Development_GDNF family signaling	8.50E-05	7/46
Signal transduction_PTEN pathway	8.50E-05	7/46
Development_Gastrin in cell growth and proliferation	8.90E-05	8/62
Development_Regulation of epithelial-to-mesenchymal transition (EMT)	1.12E-04	8/64
Cell adhesion_Plasmin signaling	1.40E-04	6/35
Development_TGF-beta receptor signaling	1.47E-04	7/50
Proteolysis_Putative ubiquitin pathway	1.59E-04	5/23
Membrane-bound ESR1: interaction with G-proteins signaling	1.67E-04	7/51
Immune response_Gastrin in inflammatory response	1.91E-04	8/69
Development_Dopamine D2 receptor transactivation of EGFR	1.97E-04	5/24
Development_WNT signaling pathway. Part 2	2.13E-04	7/53
Immune response_Role of integrins in NK cells cytotoxicity	2.24E-04	6/38
Regulation of lipid metabolism_Stimulation of Arachidonic acid production by ACM receptors	2.58E-04	8/72
PGE2 pathways in cancer	2.70E-04	7/55
Neurophysiological process_Dopamine D2 receptor transactivation of PDGFR in CNS	2.93E-04	5/26
Cell cycle_Regulation of G1/S transition (part 2)	2.93E-04	5/26
Cytoskeleton remodeling_FAK signaling	3.38E-04	7/57
Regulation of degradation of deltaF508 CFTR in CF	3.53E-04	5/27
Development_Role of IL-8 in angiogenesis	3.77E-04	7/58
Apoptosis and survival_BAD phosphorylation	3.94E-04	6/42
Development_Mu-type opioid receptor regulation of proliferation	4.22E-04	5/28
Development_VEGF signaling and activation	4.50E-04	6/43
Development_Alpha-2 adrenergic receptor activation of ERK	5.70E-04	7/62
Development_EPO-induced MAPK pathway	5.78E-04	6/45
Immune response_IL-4 - antiapoptotic action	5.89E-04	5/30
Transport_Macropinocytosis regulation by growth factors	6.28E-04	7/63
Development_TGF-beta-dependent induction of EMT via RhoA, PI3K and ILK.	6.52E-04	6/46
Development_Endothelin-1/EDNRA transactivation of EGFR	6.52E-04	6/46
Regulation of degradation of wt-CFTR	6.87E-04	4/18
Cytoskeleton remodeling_Fibronectin-binding integrins in cell motility	6.89E-04	5/31
Cytoskeleton remodeling_Reverse signaling by ephrin B	6.89E-04	5/31
Immune response_CD40 signaling	6.92E-04	7/64

Maps	p-value	Ratio
Development_EGFR signaling via small GTPases	8.01E-04	5/32
Development_PDGF signaling via STATs and NF-kB	8.01E-04	5/32
Development_PEDF signaling	9.18E-04	6/49
Development_Angiotensin activation of ERK	9.27E-04	5/33
Development_FGF2-dependent induction of EMT	1.05E-03	4/20
G-protein signaling_G-Protein beta/gamma signaling cascades	1.07E-03	5/34
Development_IGF-1 receptor signaling	1.14E-03	6/51
Development_EPO-induced Jak-STAT pathway	1.22E-03	5/35
Development_FGFR signaling pathway	1.40E-03	6/53
Cell cycle_Influence of Ras and Rho proteins on G1/S Transition	1.40E-03	6/53
Cytoskeleton remodeling_CDC42 in cellular processes	1.53E-03	4/22
Development_Beta-adrenergic receptors transactivation of EGFR	1.58E-03	5/37
Regulation of lipid metabolism_Insulin regulation of glycogen metabolism	1.70E-03	6/55
Regulation of lipid metabolism_Regulation of lipid metabolism via LXR, NF-Y and SREBP	1.78E-03	5/38
Development_EGFR signaling via PIP3	1.81E-03	4/23
Transcription_Receptor-mediated HIF regulation	2.01E-03	5/39
Cell adhesion_PLAU signaling	2.01E-03	5/39
Proteolysis_Role of Parkin in the Ubiquitin-Proteasomal Pathway	2.14E-03	4/24
Regulation of CFTR activity (norm and CF)	2.23E-03	6/58
Development_Prolactin receptor signaling	2.23E-03	6/58
Cytoskeleton remodeling_Neurofilaments	2.50E-03	4/25
Immune response_IL-17 signaling pathways	2.66E-03	6/60
Translation_Insulin regulation of translation	2.80E-03	5/42
Development_ACM2 and ACM4 activation of ERK	3.11E-03	5/43
Signal transduction_AKT signaling	3.11E-03	5/43
Immune response_Signaling pathway mediated by IL-6 and IL-1	3.34E-03	4/27
Immune response_IL-4 signaling pathway	3.45E-03	5/44
Transcription_CREB pathway	3.45E-03	5/44
Development_Flt3 signaling	3.45E-03	5/44
Development_Activation of Erk by ACM1, ACM3 and ACM5	3.45E-03	5/44
Development_Ligand-independent activation of ESR1 and ESR2	3.45E-03	5/44
Development_Thrombopoietin-regulated cell processes	3.80E-03	5/45
Development_PIP3 signaling in cardiac myocytes	4.60E-03	5/47
Development_TGF-beta-dependent induction of EMT via MAPK	4.60E-03	5/47
Immune response_Histamine H1 receptor signaling in immune response	5.04E-03	5/48
Muscle contraction_Relaxin signaling pathway	5.04E-03	5/48
Development_A3 receptor signaling	5.51E-03	5/49
Signal transduction_IP3 signaling	5.51E-03	5/49

Maps	p-value	Ratio
Cytoskeleton remodeling_Integrin outside-in signaling	5.51E-03	5/49
Apoptosis and survival_Role of IAP-proteins in apoptosis	5.57E-03	4/31
Immune response_Histamine signaling in dendritic cells	6.00E-03	5/50
Immune response_IL-13 signaling via PI3K-ERK	6.00E-03	5/50
Chemotaxis_Inhibitory action of lipoxins on IL-8- and Leukotriene B4-induced neutrophil migration	6.53E-03	5/51
Mucin expression in CF via TLRs, EGFR signaling pathways	6.53E-03	5/51
Apoptosis and survival_Caspase cascade	6.99E-03	4/33
G-protein signaling_Proinsulin C-peptide signaling	7.09E-03	5/52
Development_Endothelin-1/EDNRA signaling	7.69E-03	5/53
Cell adhesion_Alpha-4 integrins in cell migration and adhesion	7.77E-03	4/34
Development_CNTF receptor signaling	7.77E-03	4/34
G-protein signaling_G-Protein alpha-q signaling cascades	7.77E-03	4/34
Development_Angiopoietin - Tie2 signaling	8.62E-03	4/35
Development_TGF-beta-dependent induction of EMT via SMADs	8.62E-03	4/35
Development_FGF-family signaling	8.62E-03	4/35
Neurophysiological process_EphB receptors in dendritic spine morphogenesis and synaptogenesis	8.62E-03	4/35
Immune response_Fc epsilon RI pathway	8.98E-03	5/55
Immune response_IL-9 signaling pathway	9.52E-03	4/36
Muscle contraction_ACM regulation of smooth muscle contraction	9.67E-03	5/56
Immune response_TREM1 signaling pathway	9.67E-03	5/56
Inhibitory action of Lipoxins on neutrophil migration	1.04E-02	5/57
Development_Delta-type opioid receptor mediated cardioprotection	1.05E-02	4/37

Supplementary Table 14. Significantly enriched GeneGo pathway maps among the down-regulated genes following miR-128 transfection into HEY cells. Significantly enriched (FDR <0.05) GeneGo pathway maps among the down-regulated genes after miR-128 transfection into HEY cells are listed. Also shown are the hypergeometric distribution p-values of enrichments from GeneGo and the ratio of number of genes actually present in the dataset to the number of genes in the Affymetrix array that belong to each pathway. Pathways are sorted based on the enrichment p-values.

Maps	p-value	Ratio
Cell cycle_The metaphase checkpoint	2.30E-19	18/36
Cell cycle_Role of Nek in cell cycle regulation	8.80E-16	15/32
Cell cycle_Role of APC in cell cycle regulation	1.88E-11	12/32
Cell cycle_Spindle assembly and chromosome separation	2.88E-11	12/33
Cell cycle_Chromosome condensation in prometaphase	5.33E-11	10/21
Cell cycle_Initiation of mitosis	4.49E-10	10/25
Cytoskeleton remodeling_Neurofilaments	2.04E-07	8/25
Cytoskeleton remodeling_Regulation of actin cytoskeleton by Rho GTPases	1.79E-06	7/23
Proteolysis_Putative ubiquitin pathway	1.79E-06	7/23
Cytoskeleton remodeling_Cytoskeleton remodeling	3.78E-06	13/102
Cytoskeleton remodeling_Keratin filaments	4.40E-06	8/36
Cytoskeleton remodeling_TGF, WNT and cytoskeletal remodeling	9.76E-06	13/111
Cell cycle_Role of SCF complex in cell cycle regulation	9.89E-06	7/29
Cell cycle_Role of 14-3-3 proteins in cell cycle regulation	2.05E-05	6/22
Development_Role of IL-8 in angiogenesis	2.47E-05	9/58
Role of alpha-6/beta-4 integrins in carcinoma progression	2.53E-05	8/45
Development_PIP3 signaling in cardiac myocytes	3.52E-05	8/47
Proteolysis_Role of Parkin in the Ubiquitin-Proteasomal Pathway	3.53E-05	6/24
DNA damage_ATM / ATR regulation of G2 / M checkpoint	5.77E-05	6/26
Cell cycle_Influence of Ras and Rho proteins on G1/S Transition	8.61E-05	8/53
Apoptosis and survival_BAD phosphorylation	1.26E-04	7/42
Development_Slit-Robo signaling	1.36E-04	6/30
Cytoskeleton remodeling_RalA regulation pathway	1.36E-04	6/30
Cell adhesion_Gap junctions	1.36E-04	6/30
Signal transduction_AKT signaling	1.47E-04	7/43
Neurophysiological process_Receptor-mediated axon growth repulsion	1.98E-04	7/45

Maps	p-value	Ratio
Cell cycle_Cell cycle (generic schema)	2.10E-04	5/21
Transport_Macropinocytosis	2.29E-04	4/12
Immune response _CCR3 signaling in eosinophils	2.38E-04	9/77
Cell cycle_Sister chromatid cohesion	2.66E-04	5/22
Cell adhesion_Integrin-mediated cell adhesion and migration	2.99E-04	7/48
Development_EGFR signaling via PIP3	3.33E-04	5/23
Cell adhesion_Chemokines and adhesion	3.93E-04	10/100
Muscle contraction_ GPCRs in the regulation of smooth muscle tone	4.20E-04	9/83
Development_Beta-adrenergic receptors transactivation of EGFR	4.53E-04	6/37
Regulation of lipid metabolism_Regulation of lipid metabolism via LXR, NF-Y and SREBP	5.25E-04	6/38
Development_Role of HDAC and calcium/calmodulin-dependent kinase (CaMK) in control of skeletal myogenesis	6.27E-04	7/54
Immune response_BCR pathway	6.27E-04	7/54
Transport_Clathrin-coated vesicle cycle	6.77E-04	8/71
Reproduction_Progesterone-mediated oocyte maturation	6.98E-04	6/40
Cytoskeleton remodeling_Role of PKA in cytoskeleton reorganization	6.98E-04	6/40
Regulation of degradation of deltaF508 CFTR in CF	7.30E-04	5/27
Cell cycle_Transition and termination of DNA replication	8.69E-04	5/28
Transcription_CREB pathway	1.17E-03	6/44
Muscle contraction_EDG5-mediated smooth muscle contraction	1.20E-03	5/30
Apoptosis and survival_Granzyme A signaling	1.20E-03	5/30
Cell adhesion_Histamine H1 receptor signaling in the interruption of cell barrier integrity	1.32E-03	6/45
Cytoskeleton remodeling_Fibronectin-binding integrins in cell motility	1.40E-03	5/31
Cytoskeleton remodeling_Reverse signaling by ephrin B	1.40E-03	5/31
Chemotaxis_Lipoxin inhibitory action on fMLP-induced neutrophil chemotaxis	1.49E-03	6/46
Signal transduction_PTEN pathway	1.49E-03	6/46
wtCFTR and delta508 traffic / Clathrin coated vesicles formation (norm and CF)	1.54E-03	4/19
Cell cycle_Start of DNA replication in early S phase	1.63E-03	5/32
Cell adhesion_Alpha-4 integrins in cell migration and adhesion	2.15E-03	5/34
Chemotaxis_Inhibitory action of lipoxins on IL-8- and Leukotriene B4-induced neutrophil migration	2.55E-03	6/51
Transcription_Role of heterochromatin protein 1 (HP1) family in transcriptional silencing	2.73E-03	4/22
G-protein signaling_G-Protein alpha-12 signaling pathway	3.15E-03	5/37

Maps	p-value	Ratio
Cell adhesion_Role of tetraspanins in the integrin-mediated cell adhesion	3.15E-03	5/37
Development_MAG-dependent inhibition of neurite outgrowth	3.15E-03	5/37
Cell cycle_Regulation of G1/S transition (part 1)	3.55E-03	5/38
Translation _Regulation of EIF2 activity	3.98E-03	5/39
Muscle contraction_ACM regulation of smooth muscle contraction	4.10E-03	6/56
Apoptosis and survival_Apoptotic Activin A signaling	4.42E-03	4/25
Translation_Non-genomic (rapid) action of Androgen Receptor	4.45E-03	5/40
Inhibitory action of Lipoxins on neutrophil migration	4.48E-03	6/57
Immune response_IL-10 signaling pathway	5.11E-03	4/26
Development_EDG5 and EDG3 in cell proliferation and differentiation	5.11E-03	4/26
Cell cycle_Nucleocytoplasmic transport of CDK/Cyclins	5.91E-03	3/14
Development_Flt3 signaling	6.73E-03	5/44
Development_Gastrin in cell growth and proliferation	6.78E-03	6/62

Supplementary Table 15. Significantly enriched GeneGo pathway maps among the up-regulated genes following miR-7 transfection into HEY cells. Significantly enriched (FDR <0.05) GeneGo pathway maps among the up-regulated genes after miR-7 transfection into HEY cells are listed. Also shown are the hypergeometric distribution p-values of enrichments from GeneGo and the ratio of number of genes actually present in the dataset to the number of genes in the Affymetrix array that are in each pathway. In this case only 1 pathway was found to be significant.

Maps	p-value	Ratio
Transport_RAB3 regulation pathway	8.01E-07	4/14

Supplementary Table 16. Significantly enriched GeneGo pathway maps among the up-regulated genes following miR-128 transfection into HEY cells. Significantly enriched (FDR <0.05) GeneGo pathway maps among the up-regulated genes after miR-128 transfection into HEY cells are listed. Also shown are the hypergeometric distribution p-values of enrichments from GeneGo and the ratio of number of genes actually present in the dataset to the number of genes in the Affymetrix array that belong to each pathway. Pathways are sorted based on the enrichment p-values.

Maps	p-value	Ratio
Immune response_Histamine H1 receptor signaling in immune response	2.89E-07	13/48
Transport_Clathrin-coated vesicle cycle	1.18E-06	15/71
Immune response_Fc epsilon RI pathway	1.59E-06	13/55
Development_Activation of Erk by ACM1, ACM3 and ACM5	5.67E-06	11/44
Development_Regulation of epithelial-to-mesenchymal transition (EMT)	9.65E-06	13/64
Neurophysiological process_Circadian rhythm	1.13E-05	11/47
Cytoskeleton remodeling_FAK signaling	1.45E-05	12/57
Development_Angiotensin signaling via STATs	1.45E-05	9/32
Development_Leptin signaling via JAK/STAT and MAPK cascades	1.53E-05	8/25
Development_Cross-talk between VEGF and Angiopoietin 1 signaling pathways	2.12E-05	8/26
Cell adhesion_Cadherin-mediated cell adhesion	2.12E-05	8/26
Immune response_Gastrin in inflammatory response	2.27E-05	13/69
Cytoskeleton remodeling_TGF, WNT and cytoskeletal remodeling	2.32E-05	17/111
Immune response_Neurotensin-induced activation of IL-8 in colonocytes	2.42E-05	10/42
Cytoskeleton remodeling_Role of Activin A in cytoskeleton remodeling	2.74E-05	7/20
Neurophysiological process_EphB receptors in dendritic spine morphogenesis and synaptogenesis	3.20E-05	9/35
Development_Hedgehog and PTH signaling pathways in bone and cartilage development	5.17E-05	9/37
Development_TGF-beta-dependent induction of EMT via RhoA, PI3K and ILK.	5.62E-05	10/46
Immune response_Human NKG2D signaling	6.48E-05	9/38
Immune response_IL-15 signaling via JAK-STAT cascade	7.64E-05	7/23

Maps	p-value	Ratio
Development_A3 receptor signaling	9.94E-05	10/49
Signal transduction_IP3 signaling	9.94E-05	10/49
Translation_Non-genomic (rapid) action of Androgen Receptor	9.97E-05	9/40
Development_Glucocorticoid receptor signaling	1.03E-04	7/24
Immune response_Function of MEF2 in T lymphocytes	1.19E-04	10/50
Mucin expression in CF via TLRs, EGFR signaling pathways	1.42E-04	10/51
Development_A2A receptor signaling	1.81E-04	9/43
Development_A1 receptor signaling	1.98E-04	10/53
Development_Angiopoietin - Tie2 signaling	2.17E-04	8/35
Immune response_CD40 signaling	2.28E-04	11/64
Apoptosis and survival_Anti-apoptotic TNFs/NF-kB/IAP pathway	2.34E-04	7/27
Signal transduction_Calcium signaling	2.60E-04	9/45
Apoptosis and survival_NO synthesis and signaling	2.73E-04	10/55
Development_GDNF family signaling	3.10E-04	9/46
Development_Gastrin in differentiation of the gastric mucosa	3.96E-04	8/38
Immune response_CCR5 signaling in macrophages and T lymphocytes	4.27E-04	10/58
Development_Role of Activin A in cell differentiation and proliferation	5.72E-04	8/40
Cytoskeleton remodeling_Reverse signaling by ephrin B	5.84E-04	7/31
Development_EDNRB signaling	5.93E-04	9/50
Apoptosis and survival_HTR1A signaling	5.93E-04	9/50
Development_A2B receptor: action via G-protein alpha s	5.93E-04	9/50
Reproduction_GnRH signaling	6.54E-04	11/72
Transport_ACM3 in salivary glands	6.81E-04	8/41
Neurophysiological process_Netrin-1 in regulation of axon guidance	6.81E-04	8/41
Development_Gastrin in cell growth and proliferation	7.39E-04	10/62
Signal transduction_Activation of PKC via G-Protein coupled receptor	7.99E-04	9/52
Proteolysis_Role of Parkin in the Ubiquitin-Proteasomal Pathway	8.15E-04	6/24
Immune response_IL-7 signaling in B lymphocytes	9.49E-04	8/43
Development_Angiotensin signaling via PYK2	9.49E-04	8/43
Apoptosis and survival_Apoptotic Activin A signaling	1.03E-03	6/25
Development_NOTCH1-mediated pathway for NF-KB activity modulation	1.05E-03	7/34
G-protein signaling_G-Protein alpha-q signaling cascades	1.05E-03	7/34
Immune response _IFN gamma signaling pathway	1.06E-03	9/54
Immune response_CD28 signaling	1.06E-03	9/54
PGE2 pathways in cancer	1.21E-03	9/55
Development_EPO-induced Jak-STAT pathway	1.26E-03	7/35

Maps	p-value	Ratio
Cytoskeleton remodeling_Cytoskeleton remodeling	1.28E-03	13/102
Neurophysiological process_Receptor-mediated axon growth repulsion	1.30E-03	8/45
Neurophysiological process_Glutamate regulation of Dopamine D1A receptor signaling	1.30E-03	8/45
Cell adhesion_Histamine H1 receptor signaling in the interruption of cell barrier integrity	1.30E-03	8/45
Transcription_Androgen Receptor nuclear signaling	1.30E-03	8/45
Development_Keratinocyte differentiation	1.39E-03	9/56
Immune response_TREM1 signaling pathway	1.39E-03	9/56
Development_Endothelin-1/EDNRA transactivation of EGFR	1.50E-03	8/46
Immune response_MIF - the neuroendocrine-macrophage connector	1.50E-03	8/46
Development_WNT5A signaling	1.50E-03	8/46
Development_TGF-beta-dependent induction of EMT via MAPK	1.74E-03	8/47
Development_Leptin signaling via PI3K-dependent pathway	1.74E-03	8/47
Cytoskeleton remodeling_ACM3 and ACM4 in keratinocyte migration	1.78E-03	7/37
Development_Prolactin receptor signaling	1.79E-03	9/58
Development_Delta-type opioid receptor signaling via G-protein alpha-14	1.93E-03	6/28
Immune response_IL-7 signaling in T lymphocytes	2.09E-03	7/38
Cell cycle_Regulation of G1/S transition (part 1)	2.09E-03	7/38
Development_WNT signaling pathway. Part 1. Degradation of beta-catenin in the absence WNT signaling	2.25E-03	5/20
Development_Melanocyte development and pigmentation	2.29E-03	8/49
Immune response_IL-2 activation and signaling pathway	2.29E-03	8/49
Immune response_Bacterial infections in normal airways	2.29E-03	8/49
Development_PACAP signaling in neural cells	2.44E-03	7/39
Transcription_NF-kB signaling pathway	2.44E-03	7/39
G-protein signaling_Regulation of p38 and JNK signaling mediated by G-proteins	2.44E-03	7/39
Development_Thyroliberin signaling	2.56E-03	9/61
Immune response_Histamine signaling in dendritic cells	2.61E-03	8/50
Development_GM-CSF signaling	2.61E-03	8/50
Delta508-CFTR traffic / ER-to-Golgi in CF	2.78E-03	4/13
Normal wtCFTR traffic / ER-to-Golgi	2.78E-03	4/13
Development_IGF-1 receptor signaling	2.97E-03	8/51
Membrane-bound ESR1: interaction with G-proteins signaling	2.97E-03	8/51
Immune response_NFAT in immune response	2.97E-03	8/51
Some pathways of EMT in cancer cells	2.97E-03	8/51
Development_EGFR signaling pathway	3.21E-03	9/63

Maps	p-value	Ratio
Cell adhesion_Chemokines and adhesion	3.24E-03	12/100
Blood coagulation_GPIb-IX-V-dependent platelet activation	3.25E-03	10/75
Immune response_PGE2 common pathways	3.36E-03	8/52
Immune response_Antiviral actions of Interferons	3.36E-03	8/52
Cytoskeleton remodeling_CDC42 in cellular processes	3.53E-03	5/22
Muscle contraction_Regulation of eNOS activity in endothelial cells	3.58E-03	9/64
Immune response_PIP3 signaling in B lymphocytes	3.78E-03	7/42
Development_FGFR signaling pathway	3.79E-03	8/53
Development_Endothelin-1/EDNRA signaling	3.79E-03	8/53
Development_WNT signaling pathway. Part 2	3.79E-03	8/53
Development_PDGF signaling via STATs and NF-kB	3.95E-03	6/32
Immune response_Role of DAP12 receptors in NK cells	4.27E-03	8/54
Development_Role of HDAC and calcium/calmodulin-dependent kinase (CaMK) in control of skeletal myogenesis	4.27E-03	8/54
Development_ACM2 and ACM4 activation of ERK	4.33E-03	7/43
Development_VEGF signaling and activation	4.33E-03	7/43
Apoptosis and survival_Anti-apoptotic action of Gastrin	4.33E-03	7/43
Immune response_HTR2A-induced activation of cPLA2	4.33E-03	7/43
Development_Angiotensin activation of ERK	4.63E-03	6/33
Normal and pathological TGF-beta-mediated regulation of cell proliferation	4.63E-03	6/33
Transcription_CREB pathway	4.94E-03	7/44
Neurophysiological process_NMDA-dependent postsynaptic long-term potentiation in CA1 hippocampal neurons	5.20E-03	10/80
Signal transduction_Erk Interactions: Inhibition of Erk	5.40E-03	6/34
Development_Role of CDK5 in neuronal development	5.40E-03	6/34
Development_VEGF signaling via VEGFR2 - generic cascades	5.61E-03	7/45
Development_Growth hormone signaling via STATs and PLC/IP3	6.25E-03	6/35
IL-1 beta-dependent CFTR expression	6.31E-03	4/16
Transcription_Transcription regulation of aminoacid metabolism	6.31E-03	5/25
Development_G-Proteins mediated regulation MARK-ERK signaling	6.34E-03	7/46
Neurophysiological process_ACM regulation of nerve impulse	6.34E-03	7/46
Development_Hedgehog signaling	6.34E-03	7/46
Immune response_ICOS pathway in T-helper cell	6.34E-03	7/46
Bacterial infections in CF airways	6.64E-03	8/58
Regulation of CFTR activity (norm and CF)	6.64E-03	8/58
Development_PIP3 signaling in cardiac myocytes	7.15E-03	7/47
Cell adhesion_Tight junctions	7.20E-03	6/36
G-protein signaling_RAC1 in cellular process	7.20E-03	6/36

Maps	p-value	Ratio
Regulation of lipid metabolism_Stimulation of Arachidonic acid production by ACM receptors	7.86E-03	9/72
Transcription_Transcription factor Tubby signaling pathways	7.94E-03	4/17
Muscle contraction_Relaxin signaling pathway	8.03E-03	7/48
G-protein signaling_G-Protein alpha-i signaling cascades	8.85E-03	5/27
Muscle contraction_nNOS Signaling in Skeletal Muscle	8.85E-03	5/27
Neurophysiological process_Long-term depression in cerebellum	8.99E-03	7/49
Development_Mu-type opioid receptor signaling	9.41E-03	6/38
Immune response_Oncostatin M signaling via JAK-Stat in mouse cells	9.82E-03	4/18
Development_TGF-beta receptor signaling	1.00E-02	7/50
HIV-1 signaling via CCR5 in macrophages and T lymphocytes	1.07E-02	6/39
Development_ERBB-family signaling	1.07E-02	6/39
Transport_Macropinocytosis regulation by growth factors	1.09E-02	8/63
Signal transduction_PKA signaling	1.12E-02	7/51
Immune response_IL-15 signaling	1.19E-02	8/64
wtCFTR and delta508 traffic / Clathrin coated vesicles formation (norm and CF)	1.20E-02	4/19
Immune response_Delta-type opioid receptor signaling in T-cells	1.20E-02	5/29
Immune response_CD137 signaling in immune cell	1.20E-02	5/29
Immune response_MIF in innate immunity response	1.21E-02	6/40
Immune response_T cell receptor signaling pathway	1.24E-02	7/52
